# An interspecific assessment of Bergmann’s rule in 22 mammalian families

**DOI:** 10.1186/s12862-016-0778-x

**Published:** 2016-10-19

**Authors:** Jostein Gohli, Kjetil L. Voje

**Affiliations:** 1 Centre for Biodiversity Dynamics, Department of Biology, Norwegian University of Science and Technology, P.O. Box 7800, N-5020 Trondheim, Norway; 2 Centre for Ecological and Evolutionary Synthesis, Department of Biosciences, University of Oslo, P.O. Box 1066, Blindern, N-0316 Oslo Norway

**Keywords:** Allen’s rule, Bergmann’s rule, Body mass, Mammals, Phylogenetic comparative methods

## Abstract

**Background:**

Bergmann’s rule proposes that animals in cold habitats will be larger than those in warm habitats. This prediction has been tested thoroughly at the intraspecific level, but few studies have investigated the hypothesis with interspecific data using phylogenetic comparative approaches. Many clades of mammals have representatives in numerous distinct biomes, making this order highly suitable for a large-scale interspecific assessment of Bergmann’s rule. Here, we evaluate Bergmann’s rule within 22 mammalian families—with a dataset that include ~35 % of all described species—using a phylogenetic comparative approach. The method is based on an Ornstein-Uhlenbeck model of evolution that allows for joint estimation of adaptation and constraints (phylogenetic inertia) in the evolution of a trait. We use this comparative method to investigate whether body mass evolves towards phenotypic optima that are functions of median latitude, maximum latitude or temperature. We also assess the closely related Allen’s rule in five families, by testing if relative forelimb length evolves as a function of temperature or latitude.

**Results:**

Among 22 mammalian families, there was weak support for Bergmann’s rule in one family: A decrease in temperature predicted increased body mass in Canidae (canids). We also found latitude and temperature to significantly predict body mass in Geomyidae (pocket gophers); however, the association went in the opposite direction of Bergmann’s predictions. Allen’s rule was supported in one of the five examined families (Pteropodidae; megabats), but only when forelimb length evolves towards an optimum that is a function of maximum latitude, not median latitude or temperature.

**Conclusions:**

Based on this exhaustive assessment of Bergmann’s rule, we conclude that factors other than latitude and temperature are the major drivers of body mass evolution at the family level in mammals.

**Electronic supplementary material:**

The online version of this article (doi:10.1186/s12862-016-0778-x) contains supplementary material, which is available to authorized users.

## Background

Biologists have always sought ecological and evolutionary generalizations that structure the rich and complex diversity of life. Bergmann’s rule is such an eco-evolutionary generalization stating that animals will be larger in cold climates and smaller in warm climates [1]. Given latitude’s association with temperature, Bergmann’s hypothesis predicts a positive association between body mass and latitude. One might think that such a simple idea—increased body size with latitude—would be easily confirmed or discarded. However, Bergmann’s hypothesis continues to receive a great deal of attention more than 150 years after it was originally proposed.

Bergmann proposed his rule as applying to closely related species within the same genus (see Blackburn et al. [2] for a review of definitions of Bergmann’s rule), and it seems that Bergmann’s rule can operate at both the intraspecific and interspecific level [3, 4]. However, it can be argued that the expected correlation between body size and temperature—given that Bergmann’s hypothesis is correct—may differ in strength among species or populations. For example, there might be few biological differences among populations of the same species at different latitudes. Thus, if temperature clines have an effect on body mass evolution, they may be readily detectable among populations, since most other variables are the same. Conversely, temperature may describe a comparatively smaller amount of interspecific variation among species, since (many) species are different in their general biology, and will thus differ in important constraints or selective pressures on body mass. Larger amounts of residual variation in interspecific studies of body mass and temperature might therefore be expected, but species specific median or maximum latitudes may still explain a substantial amount of variation in body mass.

If we consider only endotherms—which some deem appropriate [4]—Bergmann’s rule has been thoroughly evaluated on the intraspecific level: Studies have shown that temperature or latitude is associated with body mass within species of both mammals e.g., [5–7] and birds e.g., [6, 8]. These observed positive correlations between latitude and body size within species suggests that a similar pattern could also be found among species. However, we are only aware of a handful of studies that evaluate Bergmann’s rule interspecifically in endotherms, and their collective results are inconclusive: While some studies find strong evidence [9–11], others find no evidence for Bergmann’s rule across species [12, 13].

One reason for the conflicting evidence for Bergmann’s rule at the interspecific level may be due to the different statistical models used to investigate the hypothesis. Blackburn and Hawkins [10] and Rodríguez et al. [9] investigated Bergmann’s rule using standard linear regression models which assumes that all species constitute independent data points and that shared evolutionary history among species has no effect on the data. Kamilar et al. [13] and Blackburn and Gaston [11] used Felsenstein’s [14] independent contrasts approach in their evaluation of Bergmanns’s rule in Malagasy Strepsirhines, a method that controls for phylogenetic effects by assuming that any statistical influence of the phylogeny is the result of ancestry [15]. Another comparative study by Diniz-Filho and colleges [16] use what they call Phylogenetic Eigenvector Regression (PVR) to estimate how much variation in body mass that can be due to ecological factors. None of these tests of Bergmann’s rule assume an underlying model of adaptive evolution that can disentangle how much of the estimated phylogenetic effect that stems from shared ancestry and how much that is due to adaptive evolution of body mass towards niches that are placed non-randomly on the phylogeny [17].

With the recent accumulation of high quality phylogenies, the stage is set for a large scale interspecific assessment of Bergmann’s rule in mammals. Our hypothesis is that of Bergmann: that body mass will increase as an adaptation to increased absolute latitude, and increase in response to decreased temperature. We test the hypothesis for 22 mammalian families using a phylogenetic comparative approach that allows for the joint estimation of phylogenetic inertia and adaptation in body mass based on an Ornstein-Uhlenbeck process [18–20]. More specifically, we investigate whether body mass evolves as a consequence of changes in an optimum that is modeled as a function of various predictor variable (average temperature and median and max absolute latitude).

For five mammalian families, data also allowed us to test Allen’s rule [21], an ecogeographic pattern related to Bergmann’s rule, which states that the relative size of body extremities of endotherms (e.g., limbs, tails, ears etc.) should be smaller in colder environments in order to reduce thermoregulatory costs. The level of support for Allen’s rule in the literature is comparable to Bergmann’s: Allen’s rule has been found to hold for some intraspecific temperature clines in mammals e.g., [22, 23], poikiloterms [24] and birds [25, 26], while we are only aware of two interspecific analyses, on birds [27, 28], which both found support for the rule.

## Methods

**Fig. 1:**
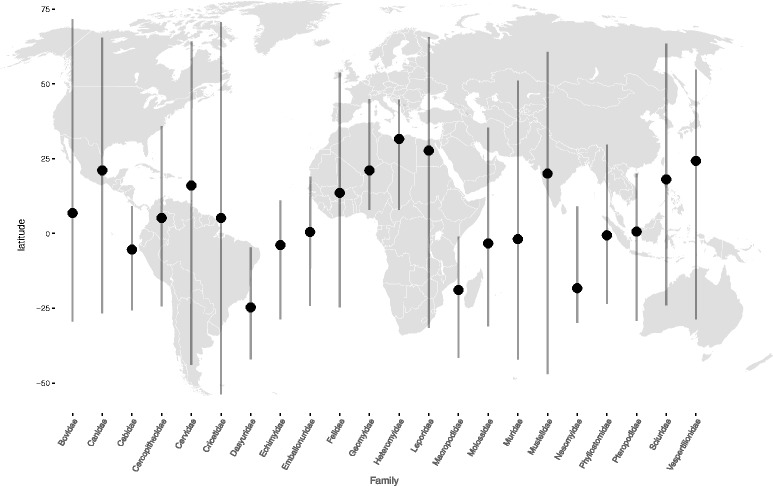
Distribution of median latitudes within each family. The dots are the within family median latitude, which are based on species median latitudes. Bars extend to minimum and maximum species specific median latitude. The families are ordered alphabetically; the figure does not contain information on longitudinal distributions. Note that absolute latitude values were used in the analyses

For testing Bergmann’s rule, we analyzed 22 mammalian families where we had data on a minimum of 25 species per family. The dataset included a total of 1871 species (listed in the SLOUCH input data files; Additional file 1), with a mean of 85 species per family. All included families showed considerable variation in their latitudinal distribution (Fig. 1), and only families who had phylogenies were the large majority of nodes where resolved were chosen for the analyses (these phylogenies are given in Additional file 1). The included families (Fig. 1; Table 1) cover the following mammalian orders: Artiodactyla, Carnivora, Chiroptera, Dasyuromorphia, Diprotodontia, Lagomorpha, Primates, Rodentia. We also performed tests of Allen’s rule, where relative forelimb length, rather than body mass, was the response variable. Here we analyzed 5 families, with a minimum of 34 and a mean of 62 species. Phylogenies for each family were extracted from Fabre et al. [29], Bibi [30], Agnarsson et al. [31] or Bininda-Emonds et al. [32]. Apart from the Agnarsson et al. phylogeny, all of the source-phylogenies were ultrametric. The length of all utrametric phylogenies was set to 1 prior to phylogenetic comparative analyses. Phylograms (non-ultrametric trees) were transformed so that the length from the root to the most distal tip equaled 1. The following data was extracted from the PanTHERIA database [33]: Median adult body mass (g), forelimb length (mm), median absolute latitude, max absolute latitude and temperature (mean monthly °C). Since a negative association between temperature and latitude is a fundamental part of Bergmann’s prediction, we performed regressions of median latitude on temperature for each family to quantify the strength of association between these two predictors.

**Table 1:** Results from regressing ln (body mass) on maximum-, median latitude, or temperature

Family	Predictor	*n*	Phylogentic half-life	Stationary variance	*r* ^2^	Optimal regression slope	AICc	AICc-*θ*AICc
	−		31.50	30.00	−	−	186.25	−
Bovidae	median latitude	81	32.50	29.00	0.030	0.983	186.11	−0.14
	max latitude		32.50	29.00	0.040	1.037	185.29	−0.96
	temperature		31.00	29.00	0.019	−1.180	186.99	0.73
Median latitude - temperature regression: *P*-value = <2e–16; Adjusted R-squared = 0.7038; estimate = −1.3470								
	−		2.41	1.40	−	−	52.12	−
Canidae	median latitude	25	11.61	5.30	0.076	0.390	53.06	0.94
	max latitude		4.41	2.10	0.085	0.103	52.72	0.60
	temperature		10.81	3.70	0.233	−0.935	48.27	−3.85
Median latitude - temperature regression: *P*-value = 4.42e–06 Adjusted R-squared = 0.5903; estimate = −1.1081								
	−		∞	200.10	−	−	15.20	−
Cebidae	median latitude	35	∞	180.10	0.040	23.736	16.49	1.29
	max latitude		∞	180.10	0.035	20.376	16.66	1.46
	temperature		∞	180.10	0.037	−56.352	16.58	1.38
Median latitude - temperature regression: *P*-value = 2.52e–12; Adjusted R-squared = 0.7714 estimate = −2.6744								
	−		∞	195.10	−	−	55.34	−
Cercopithecidae	median latitude	71	∞	104.10	0.023	8.854	55.94	0.61
	max latitude		∞	152.10	0.076	25.809	51.91	−3.42
	temperature		∞	136.10	0.039	−36.621	54.73	−0.60
Median latitude - temperature regression: *P*-value = 1.34e–14; Adjusted R-squared: 0.5731; estimate = −1.8854								
	−		0.41	0.80	−	−	62.38	−
Cervidae	median latitude	29	0.46	0.85	0.009	−0.005	64.90	2.51
	max latitude		0.31	0.70	0.031	0.007	64.41	2.02
	temperature		0.31	0.70	0.036	−0.020	64.26	1.88
Median latitude - temperature regression: *P*-value = 9.32e–08; Adjusted R-squared = 0.6457; estimate = −1.4761								
	−		2.30	1.45	−	−	616.80	−
Cricetidae	median latitude	415	2.70	1.70	0.000	−0.005	618.81	2.01
	max latitude		3.00	1.85	0.004	−0.024	617.22	0.42
	temperature		2.70	1.70	0.000	−0.002	618.89	2.08
Median latitude - temperature regression: *P*-value = <2e–16; Adjusted R-squared = 0.5502; estimate = −1.3965								
	−		1.15	1.80	−	−	136.22	−
Dasyuridae	median latitude	51	1.38	2.04	0.010	0.033	138.06	1.84
	max latitude		1.74	2.40	0.032	0.063	137.02	0.80
	temperature		1.29	1.86	0.041	−0.142	136.46	0.24
Median latitude - temperature regression: *P*-value = 9.49e–10; Adjusted R-squared = 0.5281; estimate = −1.6096								
	−		1.67	0.59	−	−	69.93	−
Echimyidae	median latitude	49	1.22	1.22	0.008	−0.029	71.98	2.05
	max latitude		3.68	1.15	0.010	0.076	71.91	1.98
	temperature		1.46	0.52	0.014	0.109	71.63	1.70
Median latitude - temperature regression: *P*-value = 2.65e–09; Adjusted R-squared = 0.523; estimate = −2.0384								
	−		43.40	12.10	−	−	55.29	−
Emballonuridae	median latitude	34	43.80	12.10	0.004	−0.587	57.72	2.44
	max latitude		44.60	12.10	0.019	0.964	57.19	1.91
	temperature		43.80	12.10	0.010	4.156	57.53	2.24
Median latitude - temperature regression: *P*-value = 0.0257; Adjusted R-squared = 0.1194; estimate = −1.9120								
	−		∞	1488.10	−	−	91.82	−
Felidae	median latitude	29	∞	1440.10	0.030	−43.326	93.71	1.88
	max latitude		∞	1320.10	0.055	53.462	92.99	1.17
	temperature		∞	1520.10	0.002	17.582	94.47	2.64
Median latitude - temperature regression: *P*-value = 6.69e–09; Adjusted R-squared = 0.7076; estimate = −1.5160								
	−		0.39	0.46	−	−	64.03	−
Geomyidae	median latitude	32	0.01	0.23	0.366	−0.035	54.29	−9.74
	max latitude		0.01	0.23	0.380	−0.030	53.55	−10.47
	temperature		0.01	0.29	0.237	0.055	60.21	−3.82
Median latitude - temperature regression: *P*-value = 1.02e–08; Adjusted R-squared = 0.6596; estimate = −1.5882								
	−		∞	390.10	−	−	70.63	−
Heteromyidae	median latitude	51	∞	375.10	0.009	−20.960	72.54	1.91
	max latitude		∞	360.10	0.050	−38.684	70.39	−0.24
	temperature		∞	345.10	0.045	71.262	70.65	0.02
Median latitude - temperature regression: *P*-value = <2e–16; Adjusted R-squared = 0.7576 estimate = −1.6277								
	−		1.36	0.55	−	−	57.47	−
Leporidae	median latitude	45	1.45	0.58	0.010	−0.014	59.42	1.95
	max latitude		1.81	0.66	0.037	−0.029	58.19	0.71
	temperature		1.21	0.50	0.011	0.024	59.41	1.94
Median latitude - temperature regression: *P*-value = 1.11e–13; Adjusted R-squared = 0.72; estimate = −1.4950								
	−		0.55	0.60	−	−	98.63	−
Macropodidae	median latitude	46	1.21	0.90	0.068	−0.068	98.59	−0.05
	max latitude		0.49	0.58	0.003	0.007	100.94	2.31
	temperature		0.97	0.74	0.071	0.098	98.01	−0.62
Median latitude - temperature regression: *P*-value = 5.36e–08; Adjusted R-squared = 0.4817; estimate = −1.7153								
	−		0.01	0.50	−	−	94.02	−
Molossidae	median latitude	41	0.01	0.50	0.016	−0.009	95.86	1.83
	max latitude		0.01	0.50	0.017	−0.009	95.80	1.78
	temperature		0.01	0.50	0.017	0.033	95.80	1.78
Median latitude - temperature regression: *P*-value = 9.92e–09; Adjusted R-squared = 0.5627; estimate = −2.5360								
	−		0.75	1.60	−	−	786.97	−
Muridae	median latitude	324	0.80	1.70	0.000	0.002	789.02	2.05
	max latitude		0.80	1.70	0.000	0.005	788.90	1.93
	temperature		0.80	1.70	0.001	0.016	788.71	1.75
Median latitude - temperature regression: *P*-value = <2e–16; Adjusted R-squared = 0.3136; estimate = −1.3391								
	−		∞	990.70	−	−	112.15	−
Mustelidae	median latitude	42	∞	975.70	0.002	−3.630	114.53	2.38
	max latitude		∞	975.70	0.001	2.613	114.55	2.41
	temperature		∞	975.70	0.003	9.162	114.48	2.33
Median latitude - temperature regression: *P*-value = <2e–16; Adjusted R-squared = 0.8448; estimate = −1.6665								
	−		∞	945.70	−	−	97.62	−
Nesomyidae	median latitude	32	∞	900.70	0.004	−22.286	100.15	2.52
	max latitude		∞	850.70	0.048	−51.197	98.82	1.19
	temperature		∞	925.70	0.007	−42.047	100.04	2.42
Median latitude - temperature regression: *P*-value = 0.54933; Adjusted R-squared = −0.0209; estimate = −0.1836								
	−		1.71	1.20	−	−	184.97	−
Phyllostomidae	median latitude	101	1.66	1.15	0.000	0.006	187.13	2.15
	max latitude		1.96	1.30	0.013	−0.050	185.82	0.85
	temperature		1.66	1.15	0.009	−0.157	186.23	1.25
Median latitude - temperature regression: *P*-value = 4.17e–08; Adjusted R-squared = 0.2556; estimate = −2.1340								
	−		0.15	1.18	−	−	134.12	−
Pteropodidae	median latitude	52	0.13	1.02	0.069	0.025	136.36	2.24
	max latitude		0.16	0.96	0.143	0.034	132.27	−1.85
	temperature		0.13	1.02	0.065	−0.126	136.71	2.59
Median latitude - temperature regression: *P*-value = 3.04e–05; Adjusted R-squared = 0.2821; estimate = −2.4749								
	−		1.41	2.50	−	−	418.47	−
Sciuridae	median latitude	196	1.21	2.20	0.005	0.018	419.71	1.24
	max latitude		1.21	2.20	0.000	0.001	420.58	2.11
	temperature		1.61	2.80	0.000	0.002	420.58	2.11
Median latitude - temperature regression: *P*-value = <2e–16; Adjusted R-squared = 0.8577; estimate = −1.8349								
	−		0.16	0.45	−	−	155.53	−
Vespertilionidae	median latitude	91	0.16	0.45	0.041	0.002	158.77	3.24
	max latitude		0.16	0.45	0.042	−0.003	158.66	3.13
	temperature		0.16	0.45	0.042	−0.006	158.72	3.20
Median latitude-temperature regression: *P*-value = <2e–16; Adjusted R-squared = 0.8307; estimate = −2.0254								

Phylogenetic half-life estimates indicate the level of phylogenetic dependency in models where no predictors were included. In models with predictors, half-life gives the average time (in lengths of the phylogeny) it takes to move half the way from an ancestral state to the optimal state, i.e. rate of adaptation. All phylogenies are scaled to a total length of 1. Stationary variance gives the residual variance when the model has reaches a stochastic equilibrium, and *r* ^2^ gives the amount of variance explained by the optimal regression. Optimal regression slope is the slope for which SLOUCH has removed the effect of phylogenetic inertia (slope expected under immediate and unconstrained adaptation). AICc values are compared to the intercept-only model (AICc-*θ*AICc) where larger negative values indicate the most improvement from the model without predictors. Also included are results from regressing median latitude on temperature for each family

We investigate whether log transformed body mass (Bergmann’s rule) and relative forelimb length (forelimb length controlled for body mass; Allen’s rule) have evolved towards optima that are influenced by latitude or temperature within different families of mammals. We did this using a phylogenetic comparative approach implemented in the R program SLOUCH, designed to study adaptive evolution of a trait along a phylogenetic tree [17–20, 34]. The output of the model can be summarized by a regression, which includes information on whether the analyzed trait is evolving towards the estimated optima, how fast (or slow) the trait approaches the optimum, and how much of the trait variation is explained by adaptation towards the optimum. The model of evolution in SLOUCH is based on an Ornstein–Uhlenbeck model and assumes that the trait (body mass and relative forelimb length in our case) has a tendency to evolve towards a ‘primary’ optimum *Θ*, defined as the average optimal state that species will reach in a given environment when ancestral constraints have disappeared [18]. The primary optimum is modeled as a linear function of the predictor variable, which evolves as if by a Brownian-motion process. Lag in adaptation towards primary optima is quantified by a half-life parameter, *t*_*1/2*_ = ln (2)/*α*, which can be interpreted as the average time it takes a species to evolve half the distance from the ancestral phenotype towards the predicted optimal phenotype. A half-life of zero means there is no evolutionary lag, while a half-life above zero indicates that adaptation is not immediate.

The SLOUCH model returns an “optimal regression”, which is the best fit of the estimated primary optimum on the response variable (e.g. ln body mass). In tests of Bergmann’s rule, this optimal regression describes the expected relationship between ln (body mass) and the predictor in the model (e.g. latitude) if there were no constraints on the evolution of body mass towards the optimal state (instantaneous adaptation). If Bergmann’s rule applies, the optimal regression coefficient would be positive in models of log body mass and the two latitude variables, while it will be negative for the optimal regression of log body mass and temperature. The optimal regression is contrasted with an “evolutionary regression”, which is the best fit of the predictor variable on the response variable. The evolutionary regression represents the observed relationship between the variables and is shallower than the optimal regression whenever there is a lag in adaptation (i.e. when the half-life is not zero). The model of evolution implemented in SLOUCH also includes a stochastic component with standard deviation *σ,* which can be interpreted as evolutionary changes in body mass due to unmeasured selective forces and genetic drift. This component of the model is reported as v_y_ = *σ* ^2^/2*α*, which signify the expected residual variance when adaptation and stochastic changes have come to an equilibrium. Generalized least squares is used for estimation of the regression parameters (i.e., the influence of the predictor on the primary optimum) and maximum likelihood for estimation of *α* and *σ* ^2^ in an iterative procedure. For a full description of the model implemented in SLOUCH, see Hansen et al. [20].

Models that include a predictor variable are referred to as adaptation models since we in these models test whether body mass (in tests of Bergmann’s rule) or relative forelimb length (in tests of Allen’s rule) evolves towards optima influenced by latitude or temperature. The adaptation models are contrasted with an intercept-only model without a predictor variable. The half-life parameter in such intercept-only models is a measure of the phylogenetic effect in the response variable, which is an estimate of how well the phylogeny explains the distribution of body mass or relative forelimb length in the investigated family. A half-life of zero in such a model means the response variable is not phylogenetically structured, while a half-life > 0 indicates that there exists an influence of phylogeny on the data. A half-life value larger than 30 times the length of the phylogeny is reported as infinity, as the OU model reduces to a Brownian motion when the half-life is very large. A phylogenetic effect can be due to slowness of adaptation, adaptation towards phylogenetically structured optima, or a combination of both. The adaptation models can determine what proportion of the phylogenetic effect within each family that can be accounted for by adaptation towards optima influenced by latitude and temperature, respectively. Adaptation models are compared to the intercept-only models using the small sample-size corrected version of Akaike information criterion (AICc); adaption models that have AICc scores that are two or more units lower than their respective intercept-only models are considered substantially better [35]. To judge whether better models (according to the AICc score) support Bergmann’s rule, we interpret the slope of the optimal regression together with the amount of variation in body mass that the optimal regression explains. All statistical analyses were done in R v3.1.3 [36].

## Results

### Tests of Bergmann’s rule

The phylogenetic effect in body mass varied among the examined families, but were generally large (Table 1). 18 out of 22 families had very strong phylogenetic effects in body mass (half-life > 0.5). We found median latitude and temperature to be strongly negatively correlated in all 22 families, except for Nesomyidae (Table 1).

**Fig. 2:**
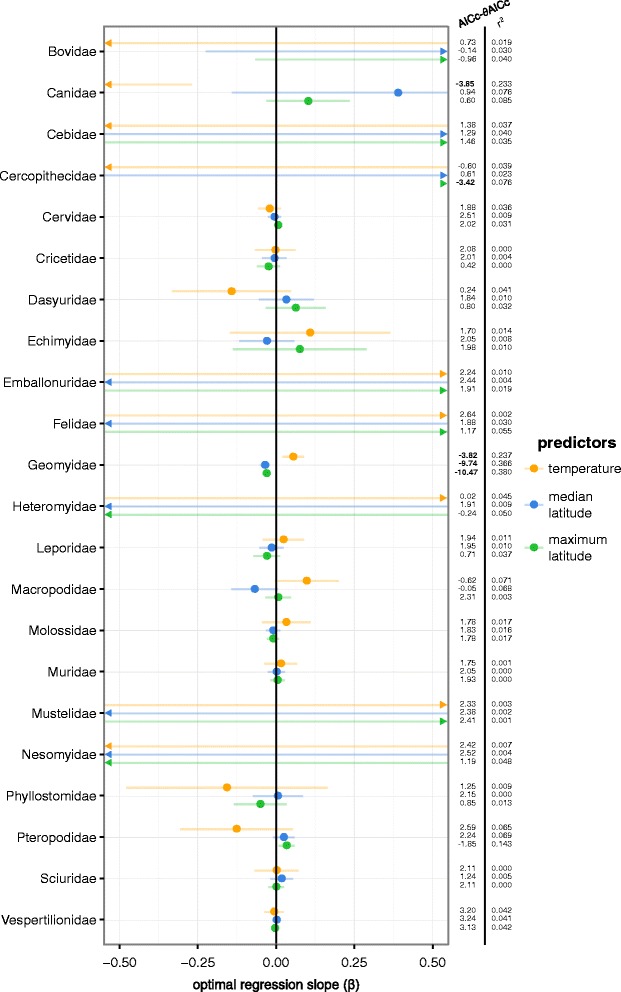
Optimal regression slopes for all body mass models. The dots give the steepness and direction of slopes (β) for all models in Table 1, where Bergmann’s rule is tested. The lines show the 95 % confidence intervals of the β estimates. When a β estimate lies outside the range of the plot, this is indicated by an arrowhead. AICc-*θ*AICc denotes the difference in AIC between the respective adaption model and the intercept-only model, and *r* ^2^ gives the amount of variation in body mass explained by the optimal regression. AICc-*θ*AICc < −2 are bolded. Each family was analyzed with three separate models, with one predictor variable in each

**Fig. 3:**
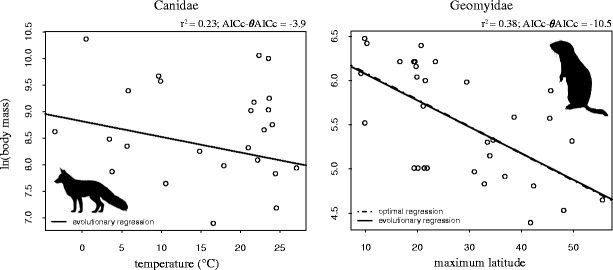
Log transformed body mass regressed on maximum latitude or temperature. The figure shows the two families where we found an association between body mass and latitude or temperature, and where these models explained a substantial proportion of the variation in body mass. Only the strongest predictor, out of temperature, maximum or median latitude, is shown for Geomyidae. AICc-*θ*AICc denotes the difference in AIC between the respective adaption model and the intercept-only model, and *r* ^2^ gives the amount of variation in body mass explained by the optimal regression. Evolutionary regressions (solid lines) represent the observed relationship between the predictor and the response variable, whereas optimal regressions are adjusted for the effect of phylogenetic inertia. The optimal regression is not shown for Canidae, due to its very high half-life estimation which produced an extremely steep slope with little biological interpretability

Generally, there were few models that gave any support to Bergmann’s rule; only five of the 66 models that used temperature, median or maximum latitude as predictors had an AICc at least two units better than the model without a predictor (Fig. 2; Table 1). The slopes of all optimal regressions, along with the associated 95 % CI, *r* ^2^, and AICc-*θ*AICc values are given in Fig. 2. Many models had steep slopes due to very large half-life values, which means body mass shows no tendency to evolve as a response to changes in the predictor in these models and that the slope estimate is not meaningful. Also, the majority of these steep slopes have a 95 % CI covering zero due to large standard errors. The negative association between temperature and body mass in Canidae (canines) is the strongest evidence in favor of Bergmann’s rule of all the models we tested (*r* ^2^ = 0.23; AICc-*θ*AICc = −3.9; Fig. 3; Fig. 2; Table 1). The half-life estimate of this model was more than ten times the length of the phylogeny, which means there is no tendency for body mass to evolve towards the optimum and that the model residuals changes similar to a Brownian motion, possibly with a trend [18, 37]. Body mass in Cercopithecidae (Old World monkeys) also showed a positive relationship with maximum latitude, but the coefficient of determination was small (*r* ^2^ = 0.08), which means that maximum latitude has marginal predictive power on body size variation within this family. The strongest relationship between body mass and a predictor variable was found for Geomyidae (pocket gophers); the model using maximum latitude as a predictor explained 39 % of the variation in body mass (AICc-*θ*AICc = −10.5; Fig. 3; Table 1), and the model using median latitude as predictor performed qualitatively similar. Importantly, Geomyidae exhibited a negative association between body mass and latitude. Half-life estimates were low for both models (<0.06), indicating rapid adaptation in body mass towards the optimum. The optimal regression slope of Geomyidae’s maximum latitude model was − 0.030, which predicts a 3 % decrease in body mass for every increase of one latitudinal degree.

### Tests of Allen’s rule

**Fig. 4:**
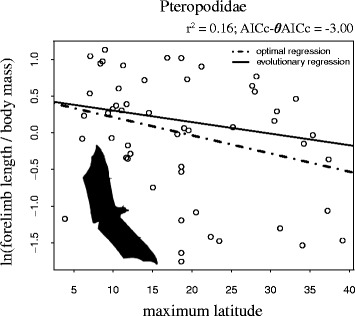
Log transformed relative forelimb length regressed on maximum latitude. The figure shows the association between relative forelimb length and latitude in Pteropodidae. AICc-*θ*AICc denotes the difference in AIC between the shown adaption model and the intercept-only model, and *r* ^2^ denotes the amount of variation in relative limb length explained by maximum latitude (optimal regression). The evolutionary regression (solid line) represents the observed relationship between relative forelimb length and maximum latitude, whereas the optimal regression is adjusted for the effect of phylogenetic inertia

We also tested Allen’s rule for five families by running models using log transformed relative forelimb length as response variable and using maximum-, median latitude, and temperature as predictor variables (Table 2). Only one of the 15 models including a predictor variable had a better AICc score than the model without any predictors: Relative forelimb length in Pteropodidae (megabats) showed a negative association with maximum latitude (*r* ^2^ = 0.16; AICc-*θ*AICc = −3.0; Fig. 4; Table 2). Half-life estimates indicate that adaptation of forelimb length as a function of maximum latitude was relatively rapid in Pteropodidae (half-life = 0.15). For each increase of one latitudinal degree, the model predicted a decrease of ~2.5 % in forelimb length. In all the five datasets (families) where we tested Allen’s rule, median latitude and temperature was strongly and negatively correlated (Table 2).

**Table 2:** Results from regressing ln (relative forelimb length) on maximum-, median latitude, and temperature

Family	Predictor	*n*	Phylogentic half-life	Stationary variance	*r* ^2^	Optimal regression slope	AICc	AICc-*θ*AICc
	−		∞	168.10	−	−	38.64	−
Emballonuridae	median latitude	34	∞	160.10	0.004	9.693	41.09	2.45
	max latitude		∞	160.10	0.025	−18.349	40.36	1.71
	temperature		∞	160.10	0.015	−88.544	40.70	2.05
Median latitude - temperature regression: *P*-value = 0.0257; Adjusted R-squared = 0.1194; estimate = −1.9120								
	−		0.01	0.25	−	−	66.48	−
Molossidae	median latitude	40	0.01	0.25	0.020	0.008	68.13	1.65
	max latitude		0.01	0.25	0.082	0.014	65.52	−0.96
	temperature		0.01	0.25	0.048	−0.039	66.95	0.47
Median latitude - temperature regression: *P*-value = 1.31e–08; Adjusted R-squared = 0.5661; estimate = −2.5311								
	−		0.73	0.43	−	−	141.60	−
Phyllostomidae	median latitude	97	0.66	0.40	0.015	0.021	142.31	0.71
	max latitude		0.86	0.45	0.031	0.033	140.81	−0.79
	temperature		0.66	0.40	0.001	−0.017	143.72	2.12
Median latitude - temperature regression: *P*-value = 3.73e–08; Adjusted R-squared = 0.2665; estimate = −2.1620								
	−		0.13	0.50	−	−	93.80	−
Pteropodidae	median latitude	52	0.13	0.45	0.068	−0.012	95.95	2.15
	max latitude		0.15	0.40	0.163	−0.025	90.80	−3.00
	temperature		0.13	0.45	0.074	0.100	95.72	1.92
Median latitude - temperature regression: *P*-value = 3.04e–05; Adjusted R-squared = 0.2821; estimate = −2.4749								
	−		0.10	0.22	−	−	105.53	−
Vespertilionidae	median latitude	88	0.06	0.18	0.100	−0.004	105.80	0.27
	max latitude		0.08	0.20	0.070	0.001	106.63	1.11
	temperature		0.06	0.18	10.11	0.010	105.69	0.17
Median latitude - temperature regression: *P*-value = <2e–16; Adjusted R-squared = 0.8224; estimate = −1.9978								

Phylogenetic half-life estimates indicate the level of phylogenetic dependency in models where no predictors were included. In models with predictors, half-life gives the time (in lengths of the phylogeny) necessary to lose half the influence of the ancestral trait, i.e. rate of adaptation. All phylogenies are scaled to a total length of 1. Stationary variance gives the residual variance when the model has reaches a stochastic equilibrium, and *r* ^2^ gives the amount of variance explained by the optimal regression model. Optimal regression slope is the slope for which SLOUCH has removed the effect of phylogenetic inertia (slope expected under instant adaptation). AICc values are compared to the intercept-only model (AICc-*θ*AICc) where larger negative values indicate the most improvement from the model without predictors. Also included are results from regressing median latitude on temperature for each family

## Discussion

Here, we have performed tests of Bergmann’s rule in 22 mammal families. To do this, we used a phylogenetic comparative method to test if log body mass evolved as a response to changes in median latitude, maximum latitude or temperature. The dataset includes ~35 % of all described species and covers eight orders, which makes this a comprehensive interspecific test of Bergmann’s rule in mammals. The general pattern was one of very little support for Bergmann’s rule, i.e., of latitude or temperature being important predictors of body mass evolution. Most of the models in SLOUCH that included a predictor variable were not better than a model without a predictor according to their AICc score and most did not have a slope estimate that differed significantly from zero (Fig. 2). Also, most models explained very little variation in body mass (Fig. 2; Table 1). We also performed tests of Allen’s rule in 5 families, were we found a similar pattern of little statistical and biological significance in the tested predictor variables (Table 2).

Only one of the 22 families exhibited a somewhat clear negative relationship between temperature and body mass, thus supporting Bergmann’s rule. In this family, Canidae, the temperature model explained about 23 % of the variation in body mass, however, this adaption model was only 4 AICc units better than the model without any predictors. Furthermore, the estimated rate of adaptation in body mass was extremely slow, which indicates that changes in temperature are not necessarily followed by any actual change in body size. Furthermore, given that we ran 66 distinct regression analyses in our tests of Bergmann’s rule, this result may be the product of a type I error. Thus, we caution that further analyses (including all 34 species) of this relationship in Canidae should be conducted before concluding that species within this family follows the prediction from Bergmann’s rule.

We did find a strong association between body mass and latitude in one of the 22 families, namely Geomyidae. Interestingly, the relationship was in the opposite direction of Bergmann’s prediction. The negative association between body mass and latitude in Geomyidae is congruent with Medina et al. [12]: In their study of a rodent genus (*Ctenomys*), which is not included in our data set, body size decreased with increasing latitude. This congruence may be explained by the fact that both *Ctenomys* and Geomyidae are subterranean rodents; the burrowing lifestyle will probably involve some constraints on body mass not found in other rodents or mammals in general. Also, the importance of overland temperature is likely less for animals that spend a lot of time underground [38, 39]. A likely causal driver for the correlation between latitude and body mass in Geomyidae is soil humidity, which seem to affects burrowing; larger species are found in areas with dry, sandy, and brittle soil [12]. This fits the observed pattern since humidity correlates negatively with overland temperature in the latitudinal range of both Geomyidae and *Ctenomys*. However, resource availability is also a possible driver of body mass evolution in Geomyidae and *Ctenomys* [12], which could be a confounding variable in our analyses of body mass in Geomyidae.

While there was no reliable support for Bergmann’s rule among the 22 families (with the potential exception of Canidae), we did detect weak support for Allen’s rule in one family of bats—namely Pteropodidae (megabats; Fig. 4; Table 2). Bats are unique, being the only truly flying mammals, and while they may use crowding and rolling up into a spherical shape in order to limit heat loss while roosting [40], the way in which they are subjected to the environment when flying is fundamentally different to other mammals. Many mammals live underground or under vegetation, and even those that do not, benefit from the insulation of the ground, terrain or vegetation [41]. The wings of bats are excellent tools for dissipating excess heat during flight in warm habitats [42], but are inherently poor at retaining heat. This is in contrast to birds, whose wings are covered in highly insulating feathers. There is some support for Allen’s rule across bird species, but only for featherless limbs [27, 28]. In fact, migratory birds that breed further north/south are expected to have longer wings due to longer migration distance [43]. With their extreme exposure to the elements, and lack of insulation on their wings, and the fact that they have relatively much longer forelimbs than any other mammal group, it is not surprising that increased latitude (i.e., lower temperatures) would inflict a strong selective pressure on wing length in Pteropodidae.

Interestingly, we found no support for Allen’s rule in a second bat family included in our study (Emballonuridae; sac-winged bats), which suggests that the result found for Pteropodidae should be interpreted with care. With large species such as the flying fox (*Pteropus vampyrus*) most commonly representing Pteropodidae, one might intuitively think that Pteropodidae’s exceedingly large wingspan (1.7 m in *P. vampyrus*) is what sets it apart from other groups of bats. And while it is true that Pteropodidae has larger average forelimb length than Emballonuridae (96.6 mm, and 54.9 mm respectively), the average forelimb length to body mass ratio is actually larger in Emballonuridae (5.13, compared to 1.02 in Pteropodidae). The two families also have similar latitudinal distributions, so this variable offers no explanation for the lack of support for Allen’s rule in Emballonuridae. One major difference between the two is that Emballonuridae species are mainly insectivorous and hawk flying insects in flight. It may be that this foraging behavior inflicts strong constraints on wing morphology—that counteracts a selective pressure towards decreasing wing span with increased latitude—which is absent in the frugivorous Pteropodidae.

Bergmann’s original formulation of his rule was about how body size variation in a group of closely related species was related to temperature (see Blackburn et al. [2]). Our investigation of whether Bergmann’s rule applies at the family level may therefore be argued to be outside the taxonomic scope of how the rule was originally formulated. However, analyzing families separately allow us to investigate whether Bergmann’s rule holds true across mammals in general. Our results indicate that temperature and latitude are not universal drivers of body mass variation among mammals.

Our main result, that temperature and latitude do not represent important factors affecting the adaptive landscape of body mass or limb length evolution within families of mammals, comes with some caveats. Performing comparative analyses of interspecific data on species means is associated with several levels of uncertainty. The members of species may show substantial variation in phenotypes and ecology, which implies that the analyzed species median values may not be very representative for all populations of a given species. This could partly have been accounted for in our analyses if variance measures for all variables were available, but this was not the case. For latitude, this issue is largely remedied by the inclusion of both median and maximum latitude in separate tests. However, the issue remains for body mass and temperature.

There is also the issue of whether our data variables are appropriate and exhaustive. For instance, we did not account for altitude in this study. It is likely that altitude and latitude have similar effects on temperature, which would entail that species living only at high elevations experience a climate similar to that at higher latitudes and lower altitudes. The inclusion of temperature as a predictor of body mass acts to control for this issue. Another potential issue is the use of mean temperature, which isn’t necessarily the most relevant metric when investigating temperature’s effect on body mass evolution; perhaps the most extreme temperature experienced by species is the most essential factor affecting body mass evolution. However, there might be a correlation between the extreme and the mean temperatures within the geographic range of most species, which means that some of the relevant variation may be captured by the mean values. Also, extreme temperature values may to some extent be represented by the maximum latitude variable.

## Conclusion

In summation, we found no reliable support for Bergmann’s rule among the 22 examined mammalian families. Further, we found weak support for Allen’s rule in only one very atypical mammalian family, the megabats. We conclude that neither Bergmann’s rule nor Allen’s rule are important interspecific phenomena in mammals at the family level.

## Additional file

Additional file 1: All phylogenies used in analyses. R script for data extraction and analyses. Detailed results/raw output from SLOUCH. SLOUCH input data. Likelihood plots for all half-life estimations. (ZIP 2442 kb)
