# Supplementary figures and images for: An interspecific assessment of Bergmann’s rule in 22 mammalian families (part 2 of 2)
Source: BMC Evol Biol. 2016 Oct 19;16:222. doi: 10.1186/s12862-016-0778-x (PMC5069937; doi:10.1186/s12862-016-0778-x)

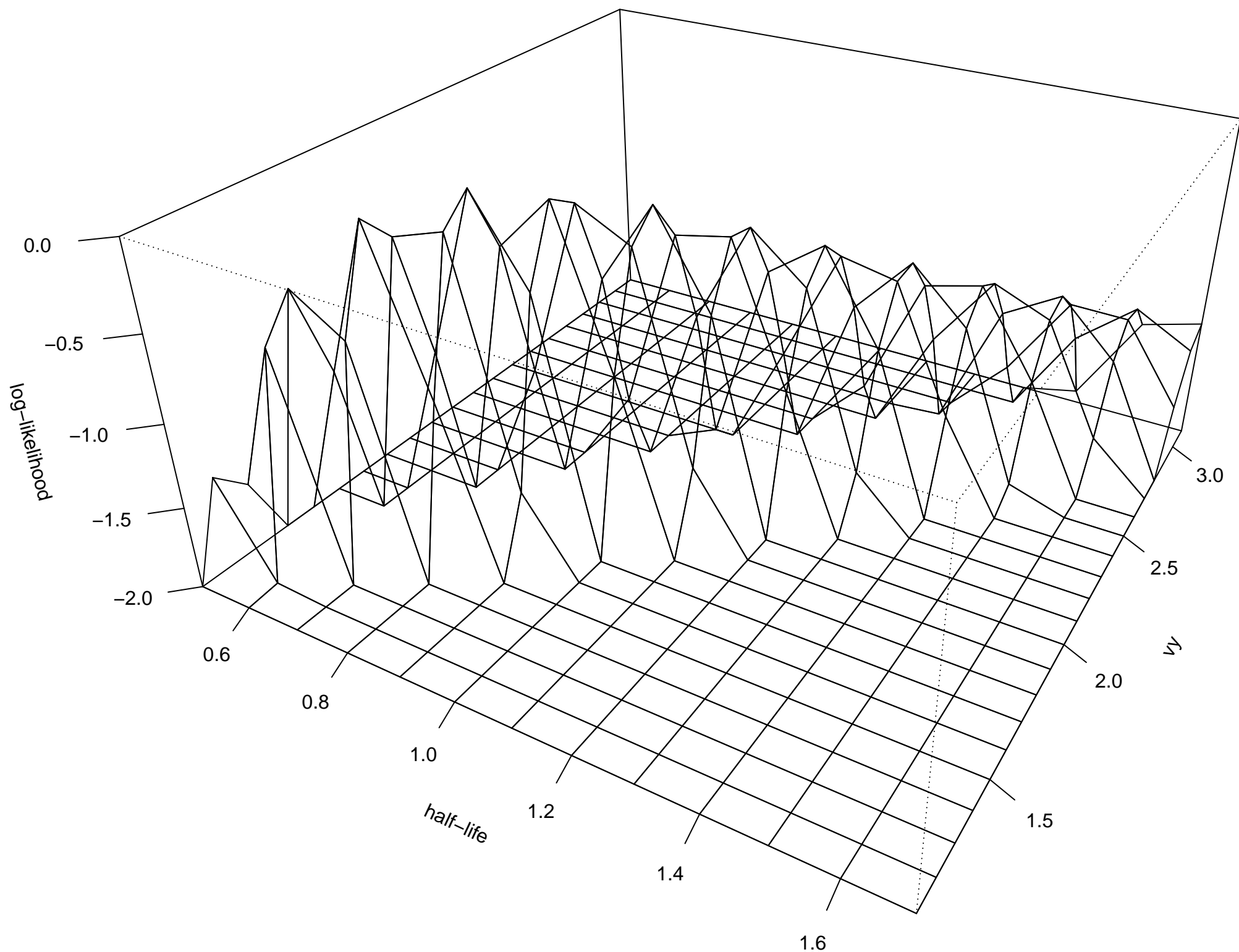

Supplement: Additional file 1: — All phylogenies used in analyses. R script for data extraction and analyses. Detailed results/raw output from SLOUCH. SLOUCH input data. Likelihood plots for all half-life estimations. (ZIP 2442 kb) [file 12862_2016_778_MOESM1_ESM.zip › Additional file 1/Results Bergman's rule - body mass/Muridae_BM_maxlat.pdf]

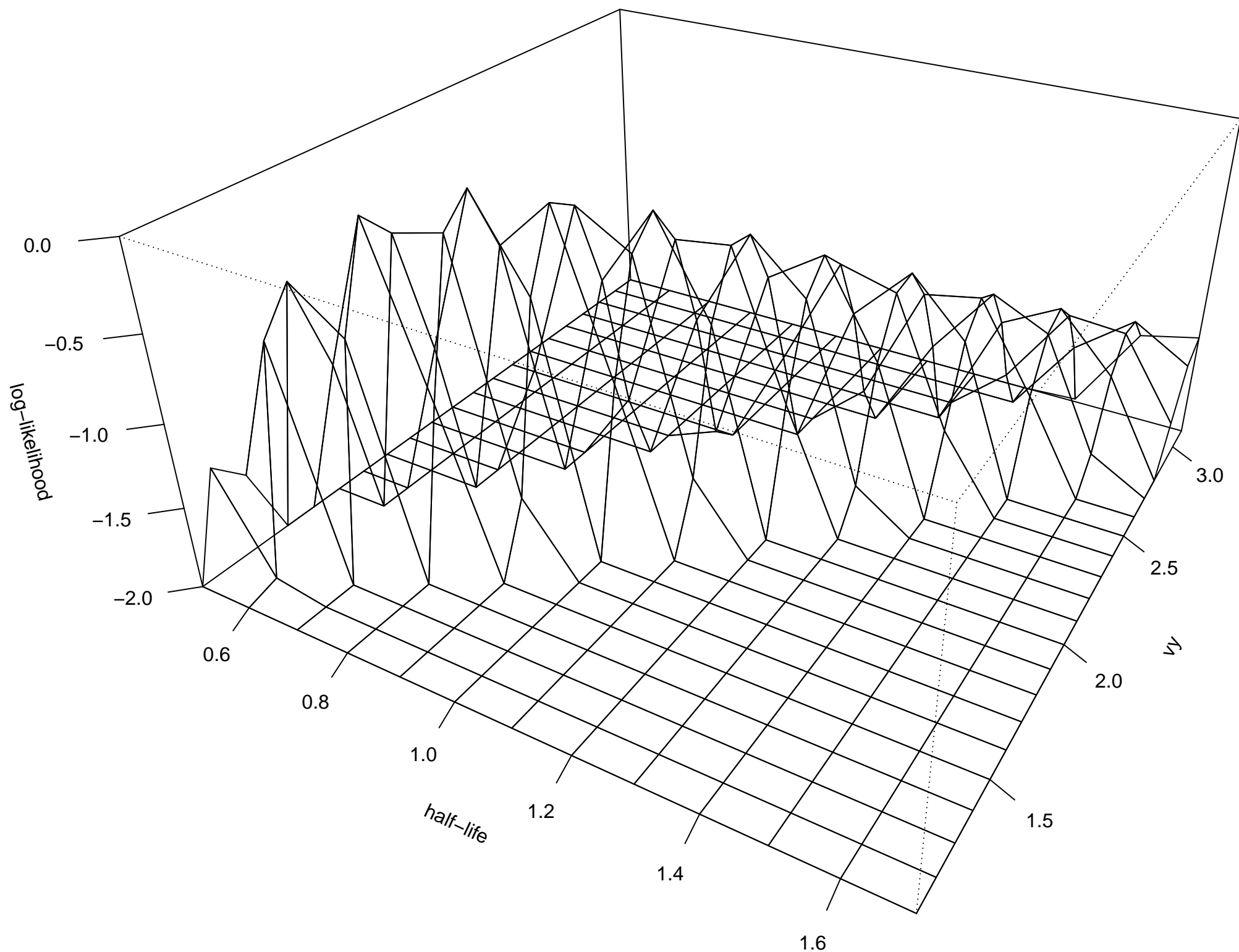

Supplement: Additional file 1: — All phylogenies used in analyses. R script for data extraction and analyses. Detailed results/raw output from SLOUCH. SLOUCH input data. Likelihood plots for all half-life estimations. (ZIP 2442 kb) [file 12862_2016_778_MOESM1_ESM.zip › Additional file 1/Results Bergman's rule - body mass/Muridae_BM_midlat.pdf]

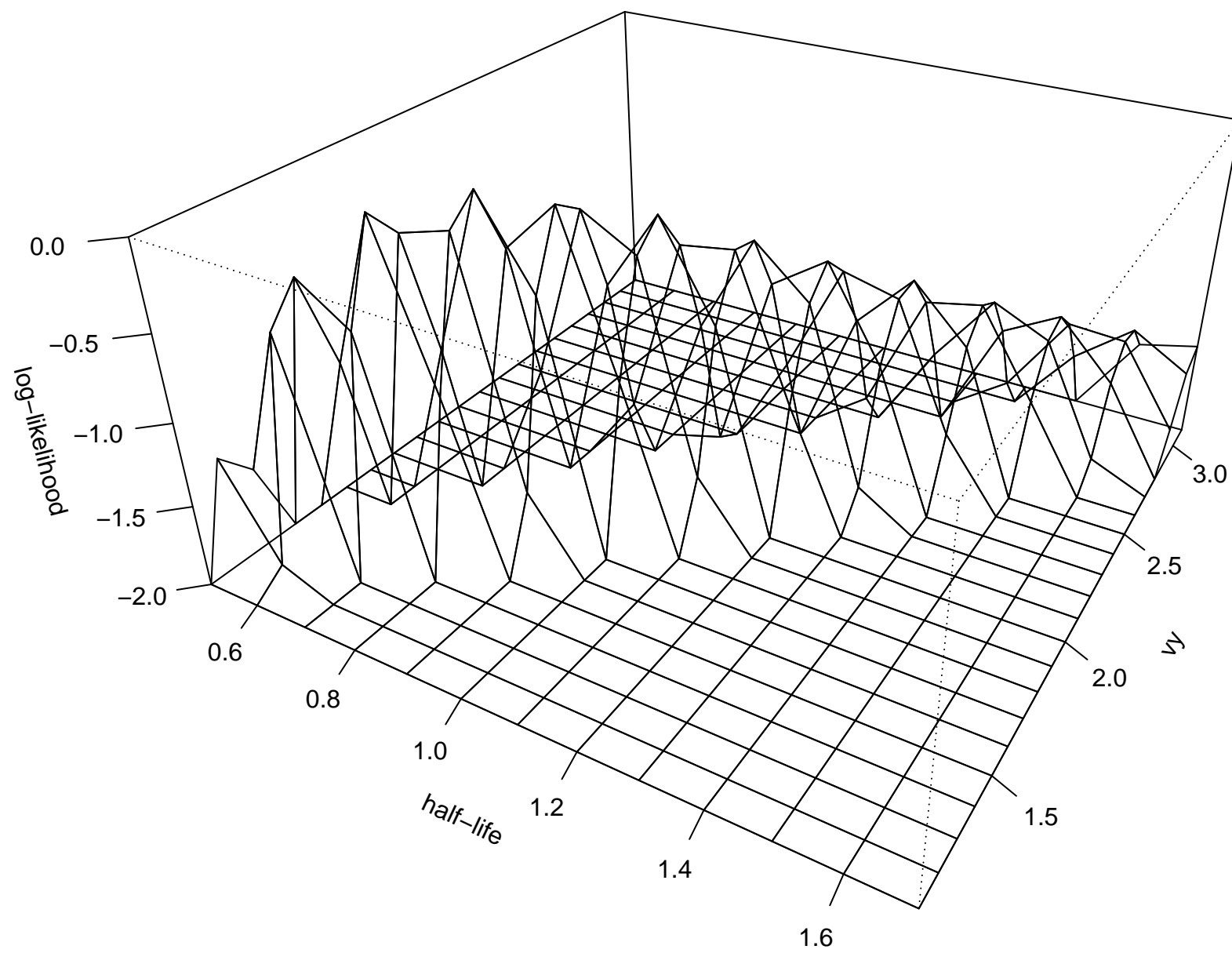

Supplement: Additional file 1: — All phylogenies used in analyses. R script for data extraction and analyses. Detailed results/raw output from SLOUCH. SLOUCH input data. Likelihood plots for all half-life estimations. (ZIP 2442 kb) [file 12862_2016_778_MOESM1_ESM.zip › Additional file 1/Results Bergman's rule - body mass/Muridae_BM_temp.pdf]

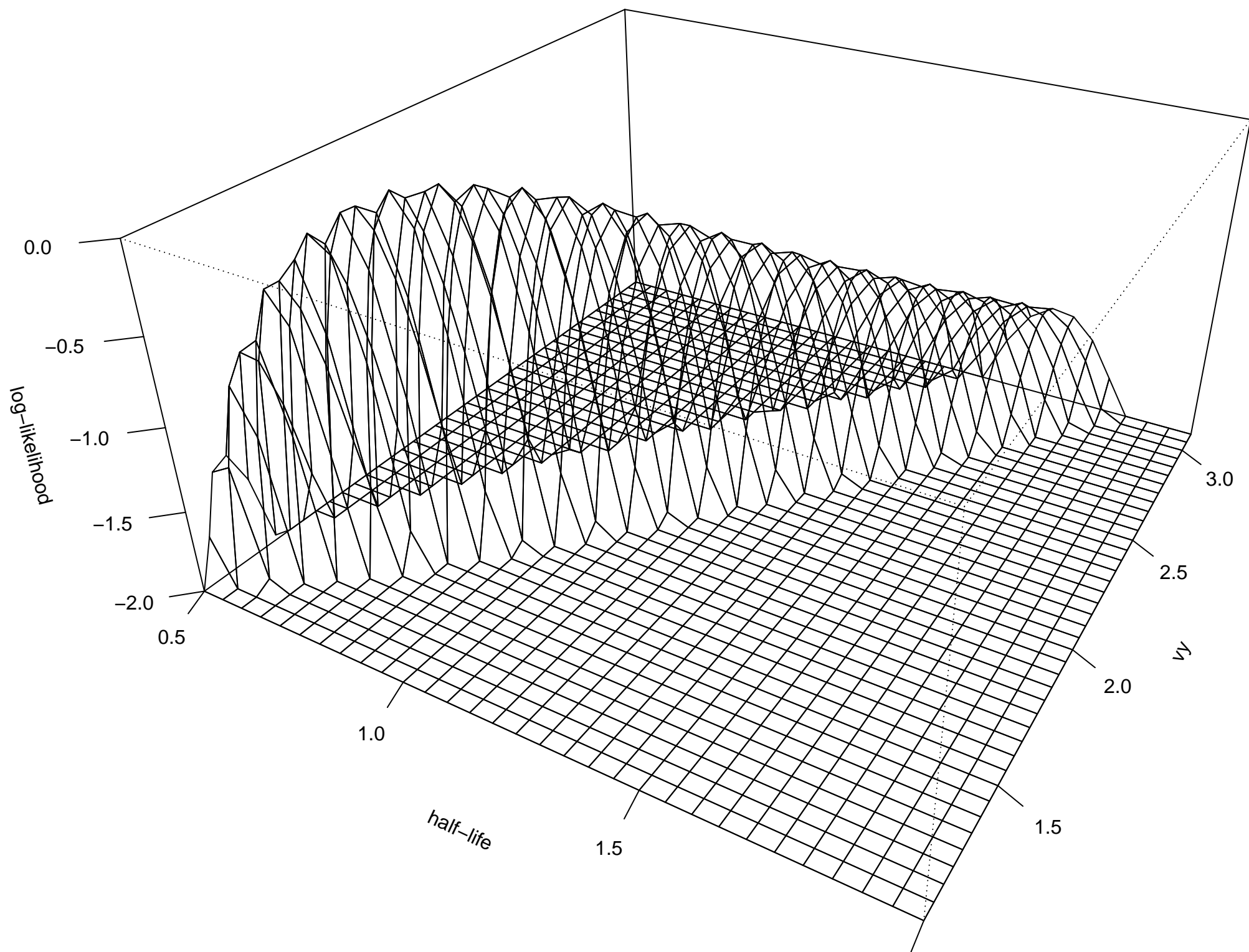

Supplement: Additional file 1: — All phylogenies used in analyses. R script for data extraction and analyses. Detailed results/raw output from SLOUCH. SLOUCH input data. Likelihood plots for all half-life estimations. (ZIP 2442 kb) [file 12862_2016_778_MOESM1_ESM.zip › Additional file 1/Results Bergman's rule - body mass/Muridae_phySig.pdf]

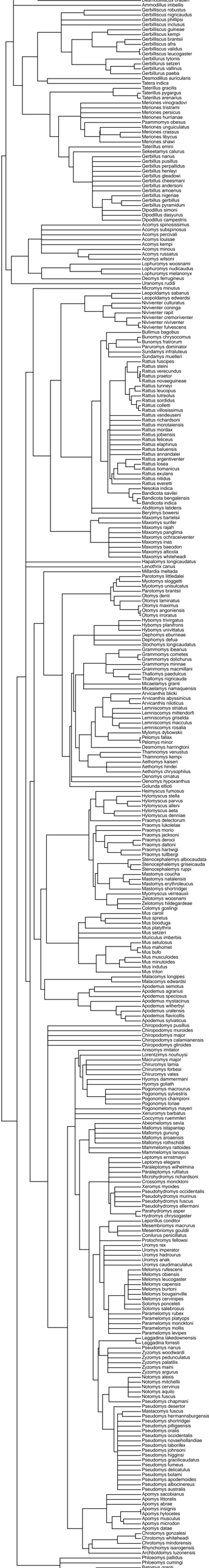

Supplement: Additional file 1: — All phylogenies used in analyses. R script for data extraction and analyses. Detailed results/raw output from SLOUCH. SLOUCH input data. Likelihood plots for all half-life estimations. (ZIP 2442 kb) [file 12862_2016_778_MOESM1_ESM.zip › Additional file 1/Results Bergman's rule - body mass/Muridae_tree.pdf]

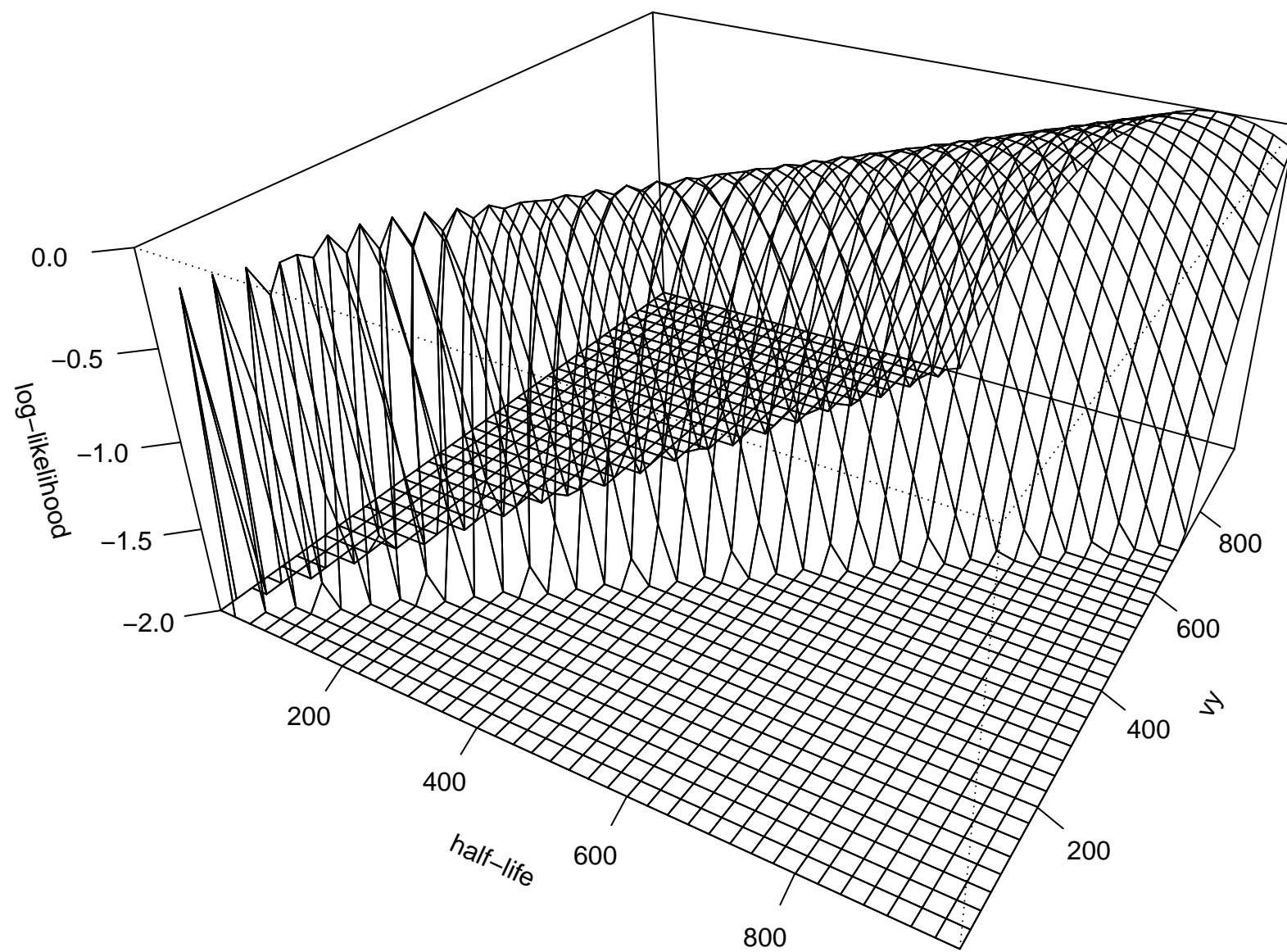

Supplement: Additional file 1: — All phylogenies used in analyses. R script for data extraction and analyses. Detailed results/raw output from SLOUCH. SLOUCH input data. Likelihood plots for all half-life estimations. (ZIP 2442 kb) [file 12862_2016_778_MOESM1_ESM.zip › Additional file 1/Results Bergman's rule - body mass/Mustelidae_BM_maxlat.pdf]

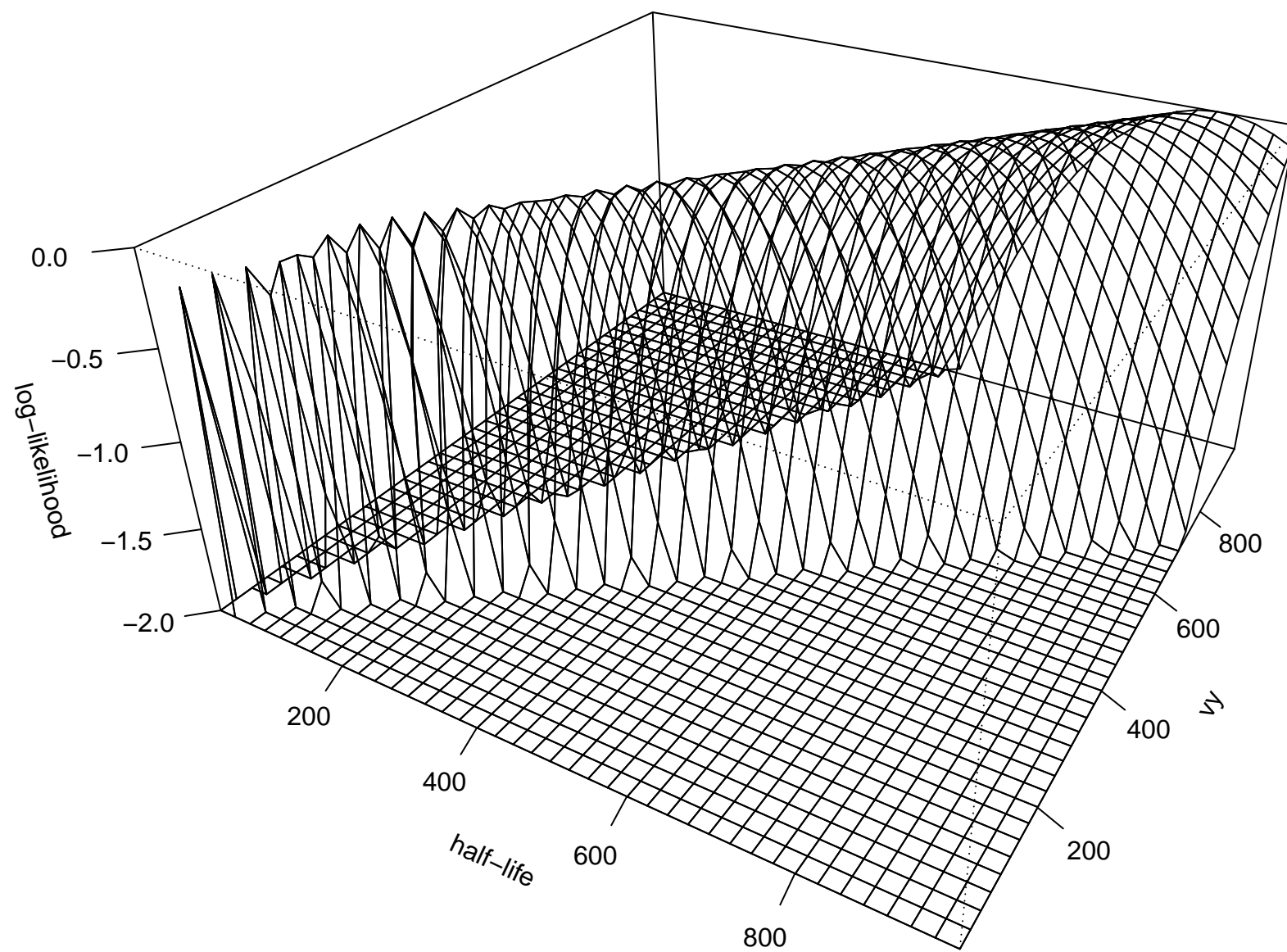

Supplement: Additional file 1: — All phylogenies used in analyses. R script for data extraction and analyses. Detailed results/raw output from SLOUCH. SLOUCH input data. Likelihood plots for all half-life estimations. (ZIP 2442 kb) [file 12862_2016_778_MOESM1_ESM.zip › Additional file 1/Results Bergman's rule - body mass/Mustelidae_BM_midlat.pdf]

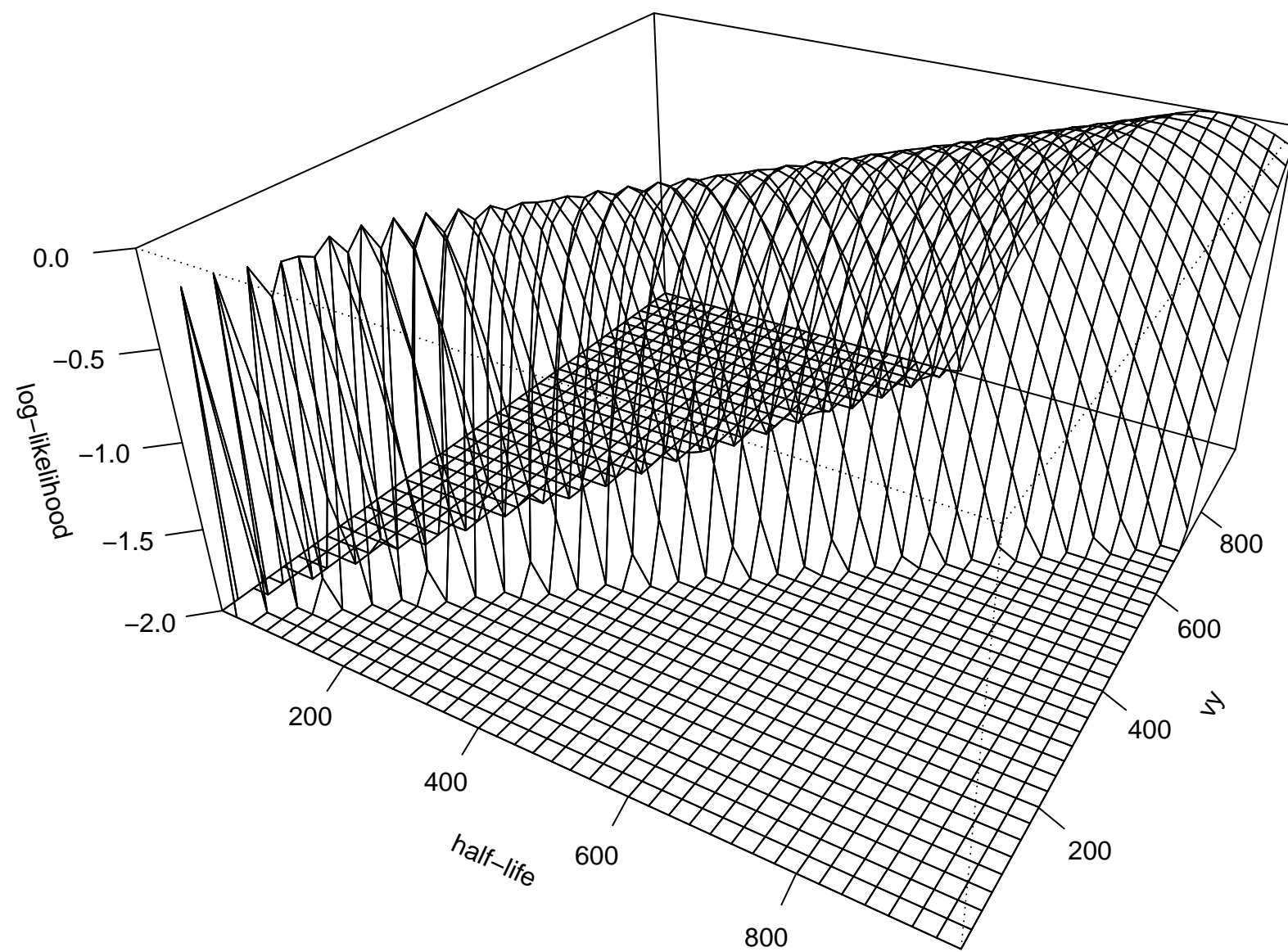

Supplement: Additional file 1: — All phylogenies used in analyses. R script for data extraction and analyses. Detailed results/raw output from SLOUCH. SLOUCH input data. Likelihood plots for all half-life estimations. (ZIP 2442 kb) [file 12862_2016_778_MOESM1_ESM.zip › Additional file 1/Results Bergman's rule - body mass/Mustelidae_BM_temp.pdf]

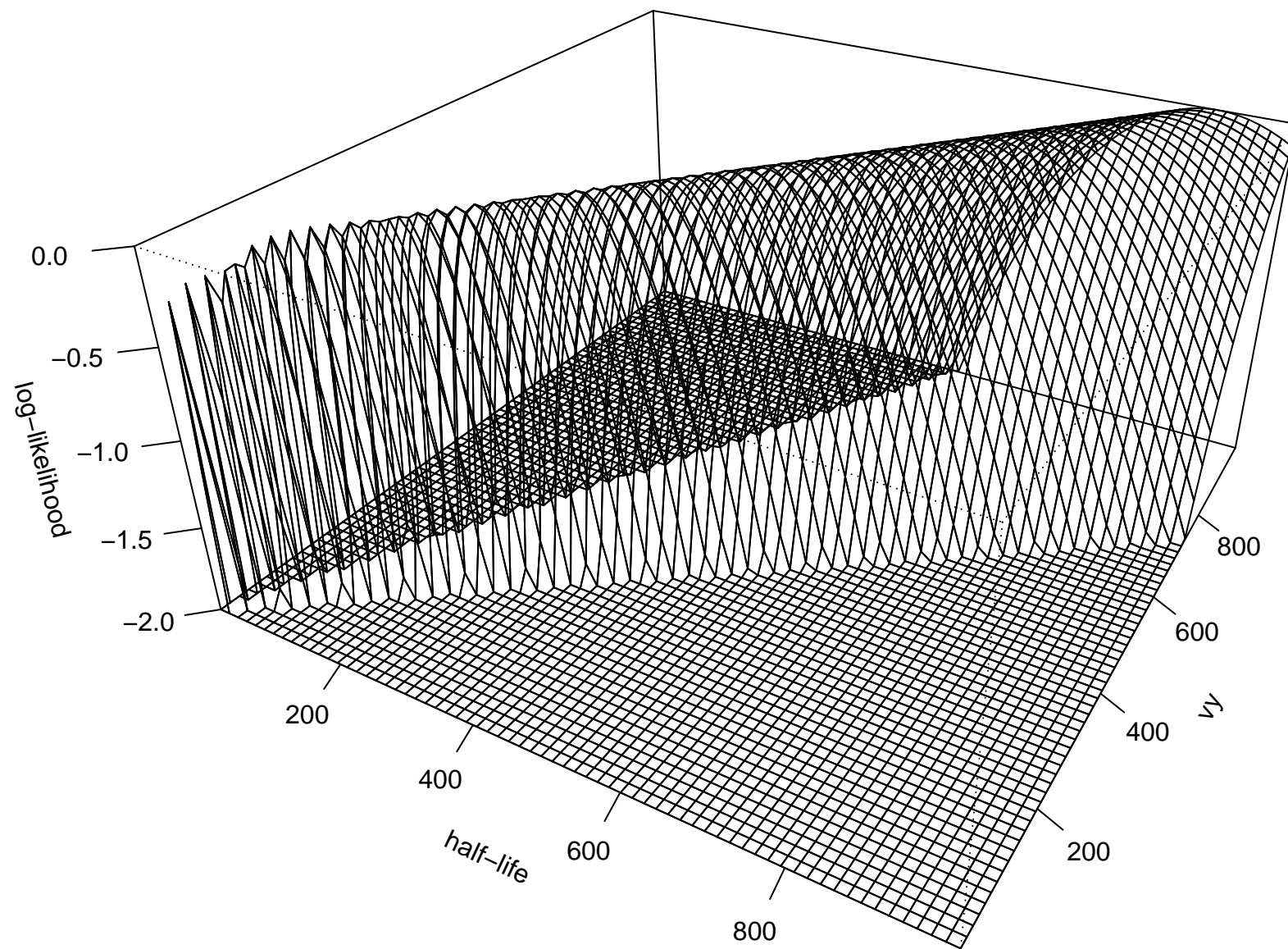

Supplement: Additional file 1: — All phylogenies used in analyses. R script for data extraction and analyses. Detailed results/raw output from SLOUCH. SLOUCH input data. Likelihood plots for all half-life estimations. (ZIP 2442 kb) [file 12862_2016_778_MOESM1_ESM.zip › Additional file 1/Results Bergman's rule - body mass/Mustelidae_phySig.pdf]

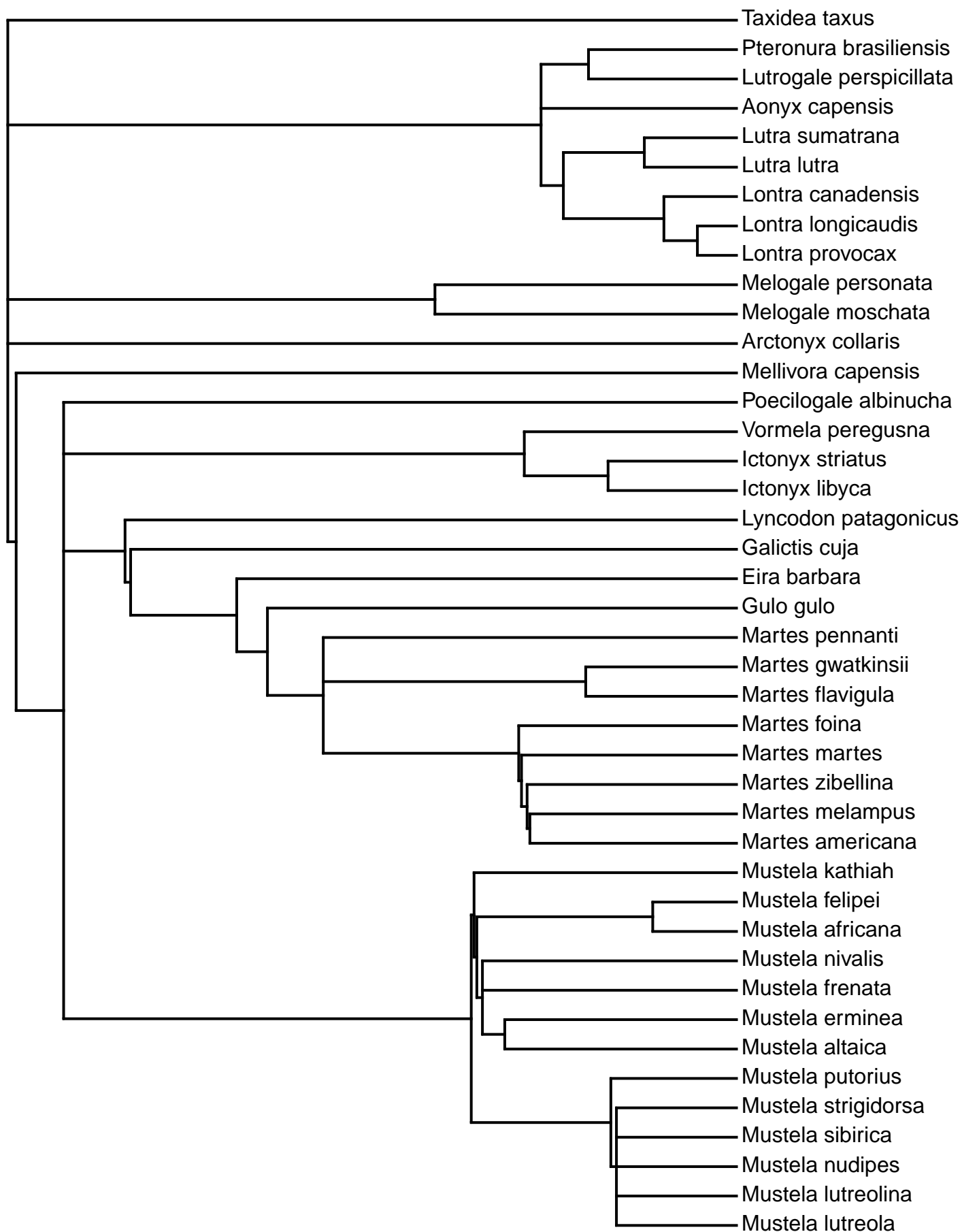

Supplement: Additional file 1: — All phylogenies used in analyses. R script for data extraction and analyses. Detailed results/raw output from SLOUCH. SLOUCH input data. Likelihood plots for all half-life estimations. (ZIP 2442 kb) [file 12862_2016_778_MOESM1_ESM.zip › Additional file 1/Results Bergman's rule - body mass/Mustelidae_tree.pdf]

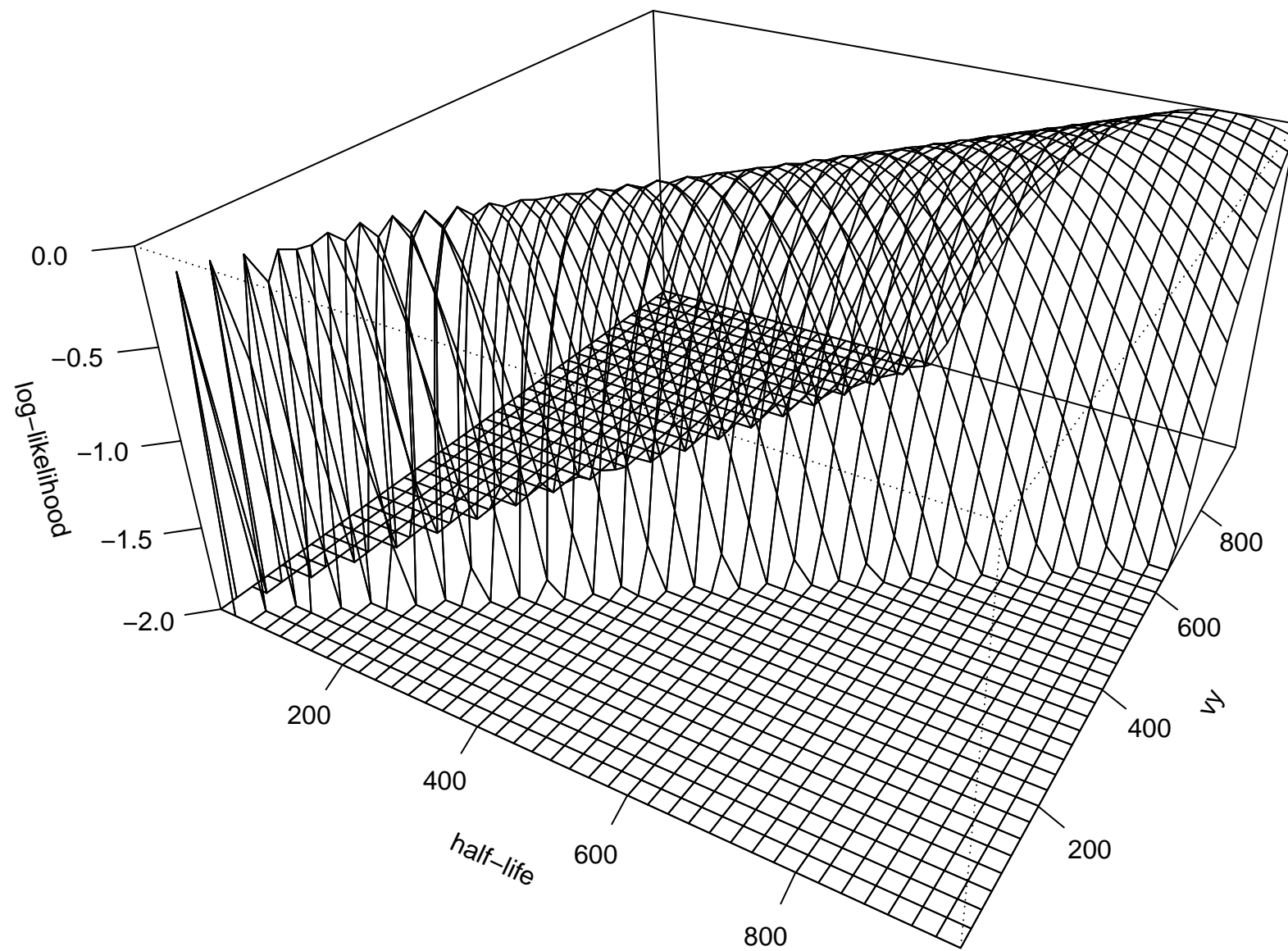

Supplement: Additional file 1: — All phylogenies used in analyses. R script for data extraction and analyses. Detailed results/raw output from SLOUCH. SLOUCH input data. Likelihood plots for all half-life estimations. (ZIP 2442 kb) [file 12862_2016_778_MOESM1_ESM.zip › Additional file 1/Results Bergman's rule - body mass/Nesomyidae_BM_maxlat.pdf]

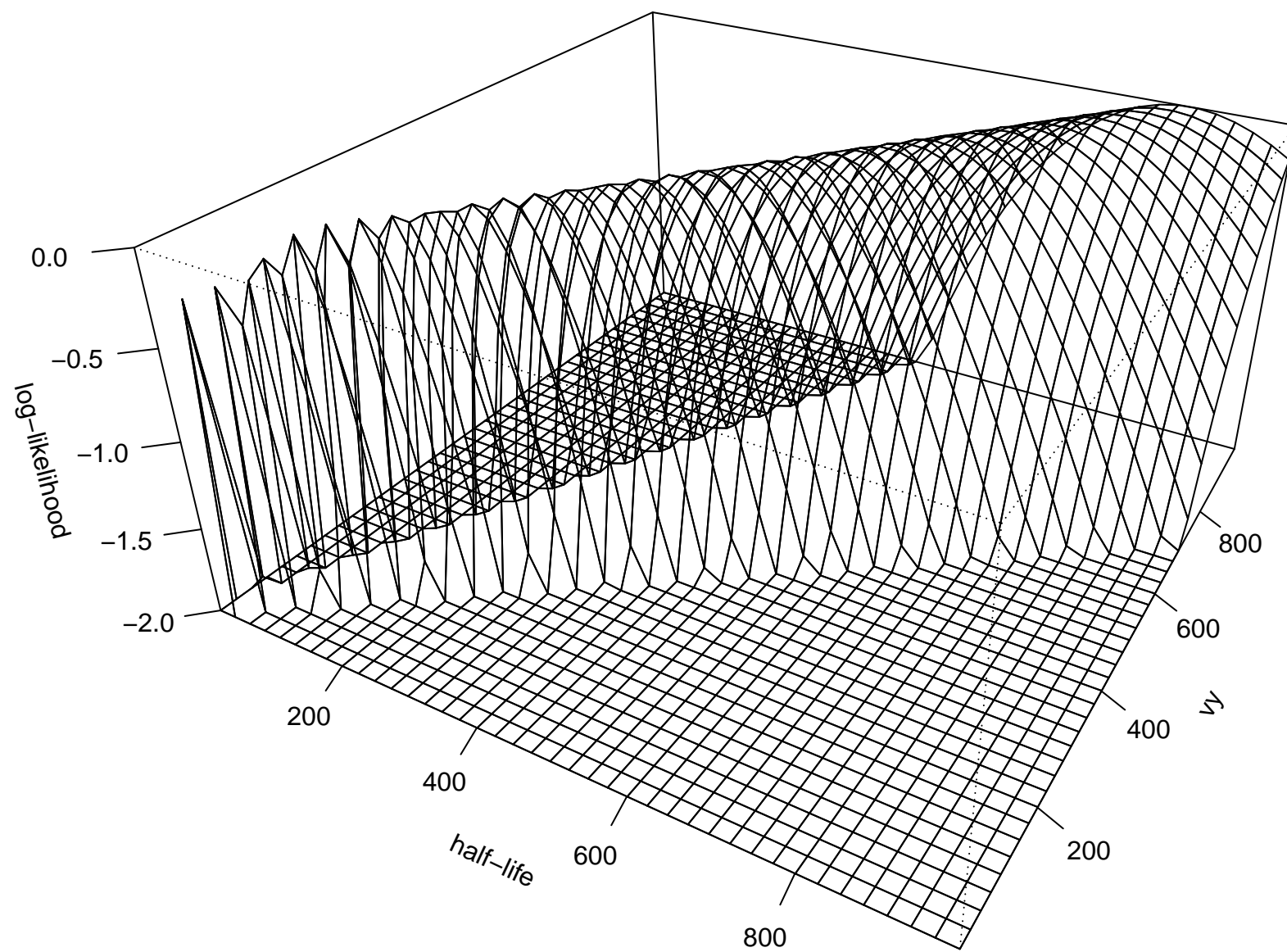

Supplement: Additional file 1: — All phylogenies used in analyses. R script for data extraction and analyses. Detailed results/raw output from SLOUCH. SLOUCH input data. Likelihood plots for all half-life estimations. (ZIP 2442 kb) [file 12862_2016_778_MOESM1_ESM.zip › Additional file 1/Results Bergman's rule - body mass/Nesomyidae_BM_midlat.pdf]

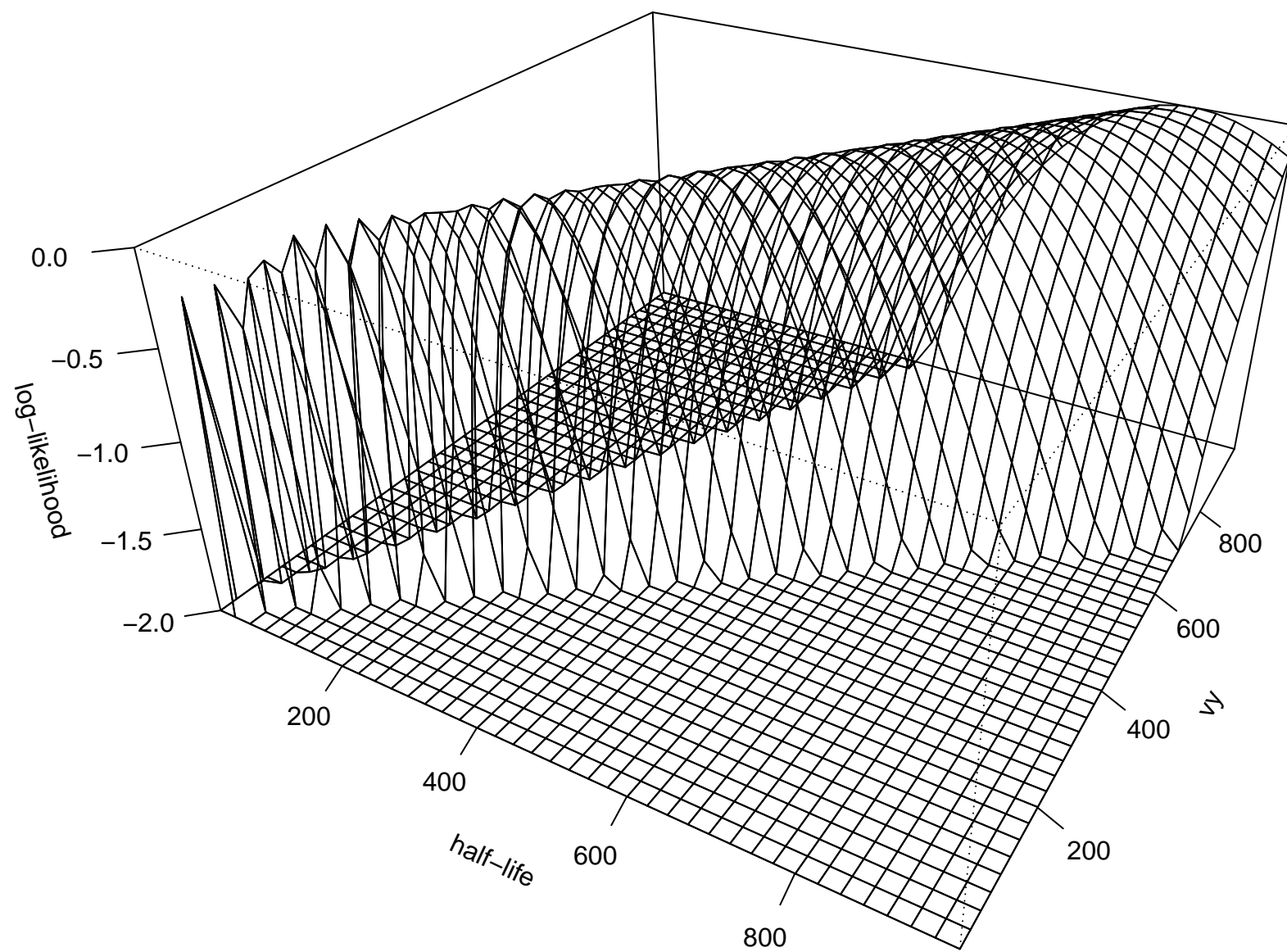

Supplement: Additional file 1: — All phylogenies used in analyses. R script for data extraction and analyses. Detailed results/raw output from SLOUCH. SLOUCH input data. Likelihood plots for all half-life estimations. (ZIP 2442 kb) [file 12862_2016_778_MOESM1_ESM.zip › Additional file 1/Results Bergman's rule - body mass/Nesomyidae_BM_temp.pdf]

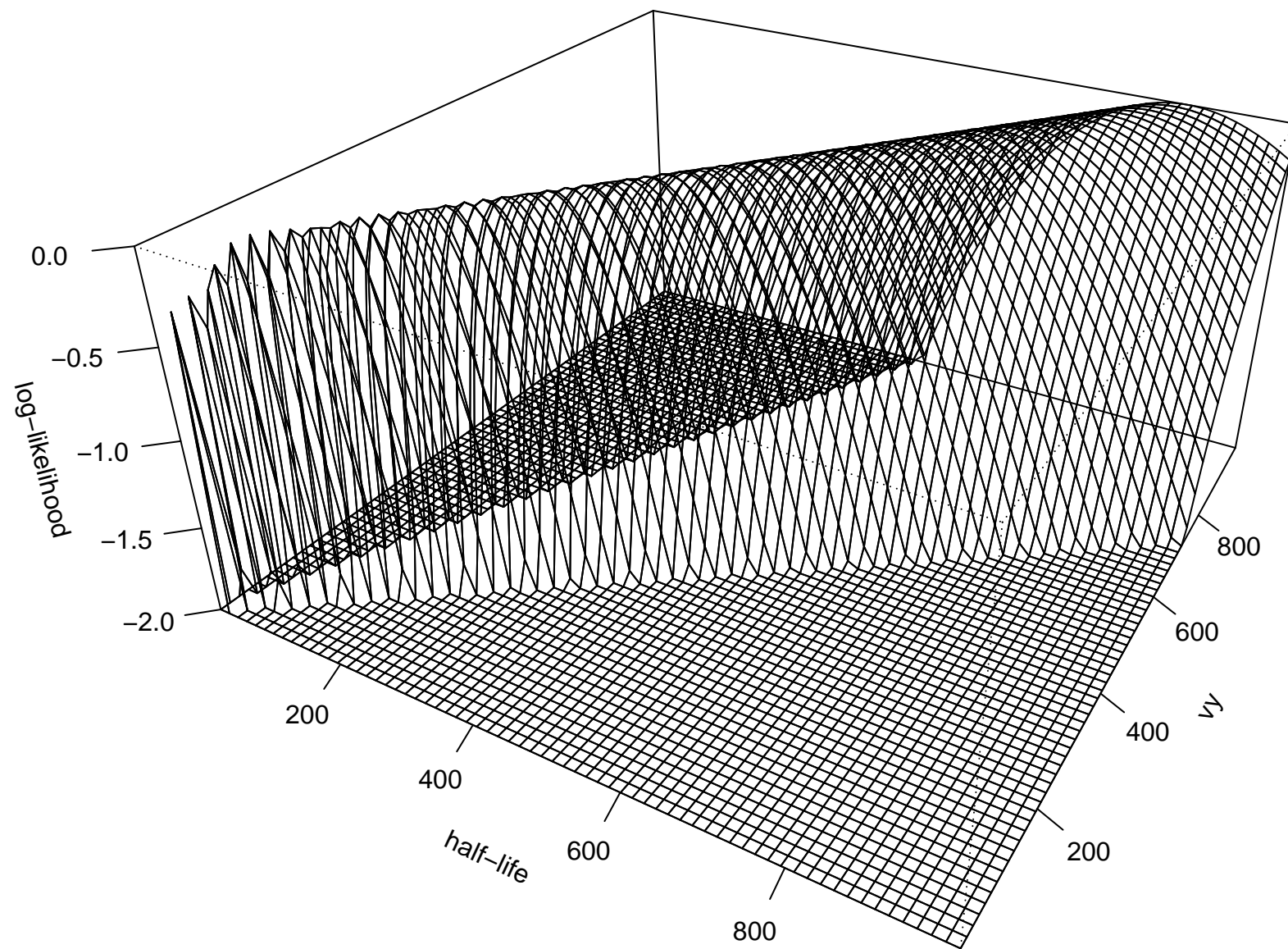

Supplement: Additional file 1: — All phylogenies used in analyses. R script for data extraction and analyses. Detailed results/raw output from SLOUCH. SLOUCH input data. Likelihood plots for all half-life estimations. (ZIP 2442 kb) [file 12862_2016_778_MOESM1_ESM.zip › Additional file 1/Results Bergman's rule - body mass/Nesomyidae_phySig.pdf]

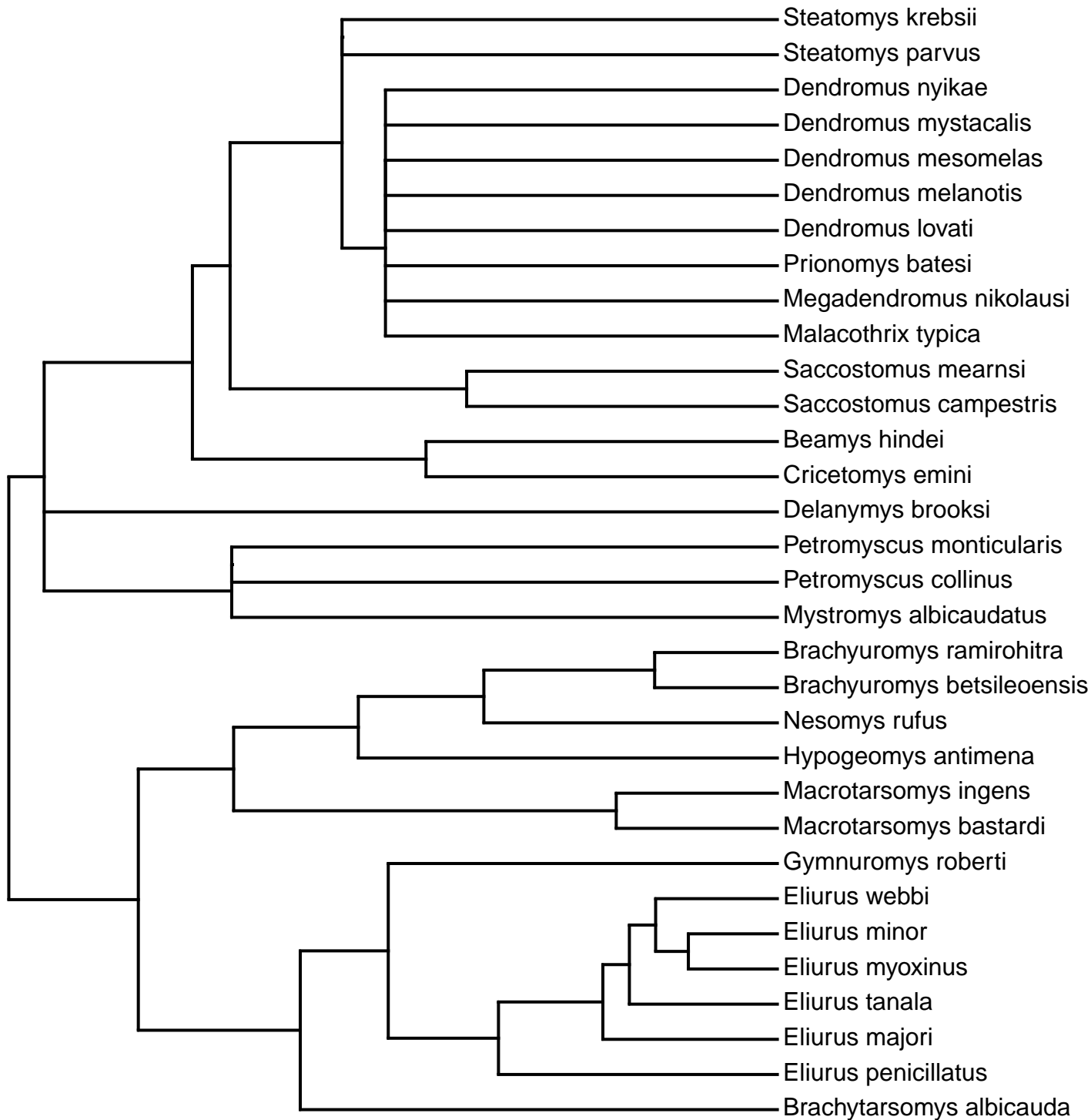

Supplement: Additional file 1: — All phylogenies used in analyses. R script for data extraction and analyses. Detailed results/raw output from SLOUCH. SLOUCH input data. Likelihood plots for all half-life estimations. (ZIP 2442 kb) [file 12862_2016_778_MOESM1_ESM.zip › Additional file 1/Results Bergman's rule - body mass/Nesomyidae_tree.pdf]

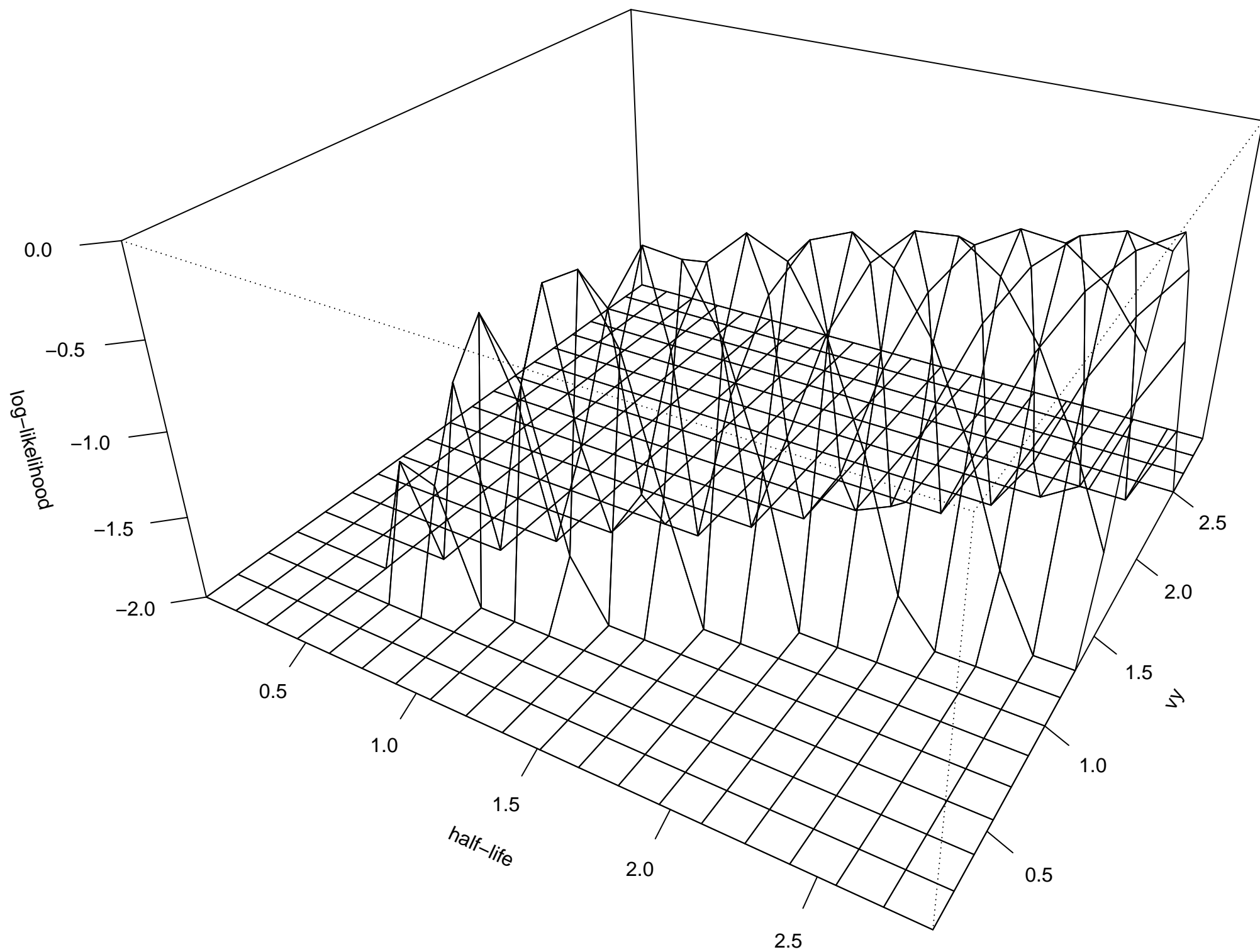

Supplement: Additional file 1: — All phylogenies used in analyses. R script for data extraction and analyses. Detailed results/raw output from SLOUCH. SLOUCH input data. Likelihood plots for all half-life estimations. (ZIP 2442 kb) [file 12862_2016_778_MOESM1_ESM.zip › Additional file 1/Results Bergman's rule - body mass/Phyllostomidae_BM_maxlat.pdf]

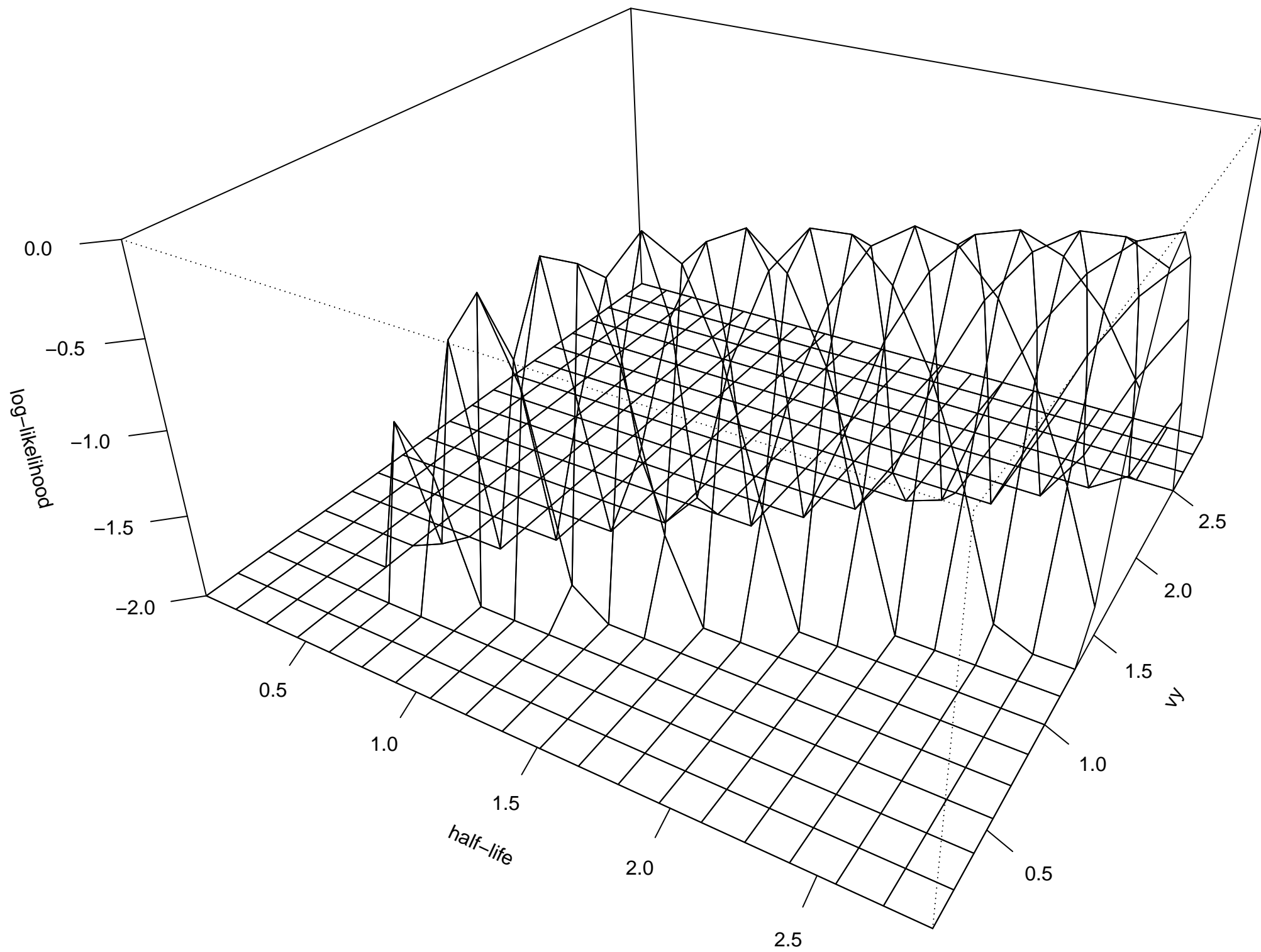

Supplement: Additional file 1: — All phylogenies used in analyses. R script for data extraction and analyses. Detailed results/raw output from SLOUCH. SLOUCH input data. Likelihood plots for all half-life estimations. (ZIP 2442 kb) [file 12862_2016_778_MOESM1_ESM.zip › Additional file 1/Results Bergman's rule - body mass/Phyllostomidae_BM_midlat.pdf]

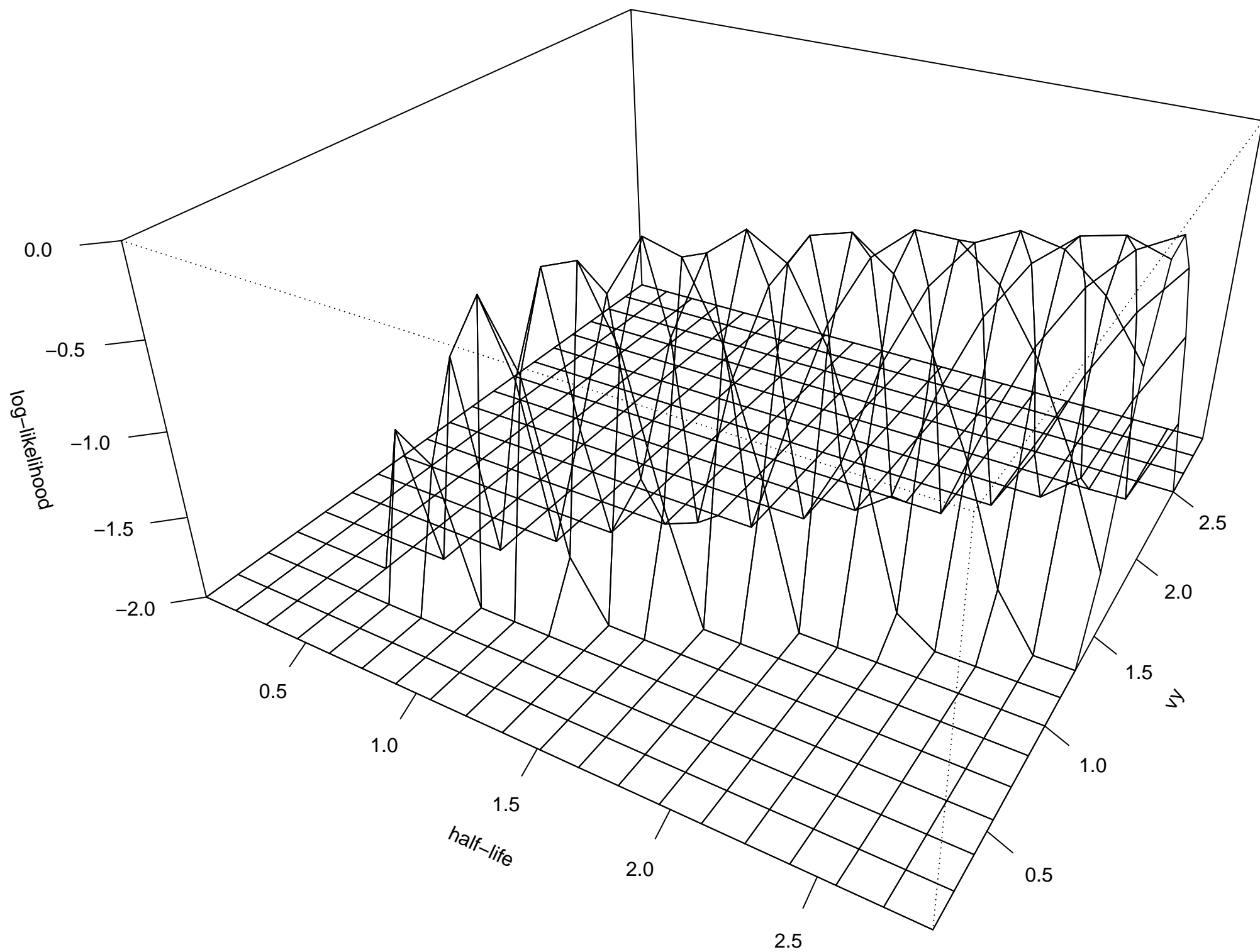

Supplement: Additional file 1: — All phylogenies used in analyses. R script for data extraction and analyses. Detailed results/raw output from SLOUCH. SLOUCH input data. Likelihood plots for all half-life estimations. (ZIP 2442 kb) [file 12862_2016_778_MOESM1_ESM.zip › Additional file 1/Results Bergman's rule - body mass/Phyllostomidae_BM_temp.pdf]

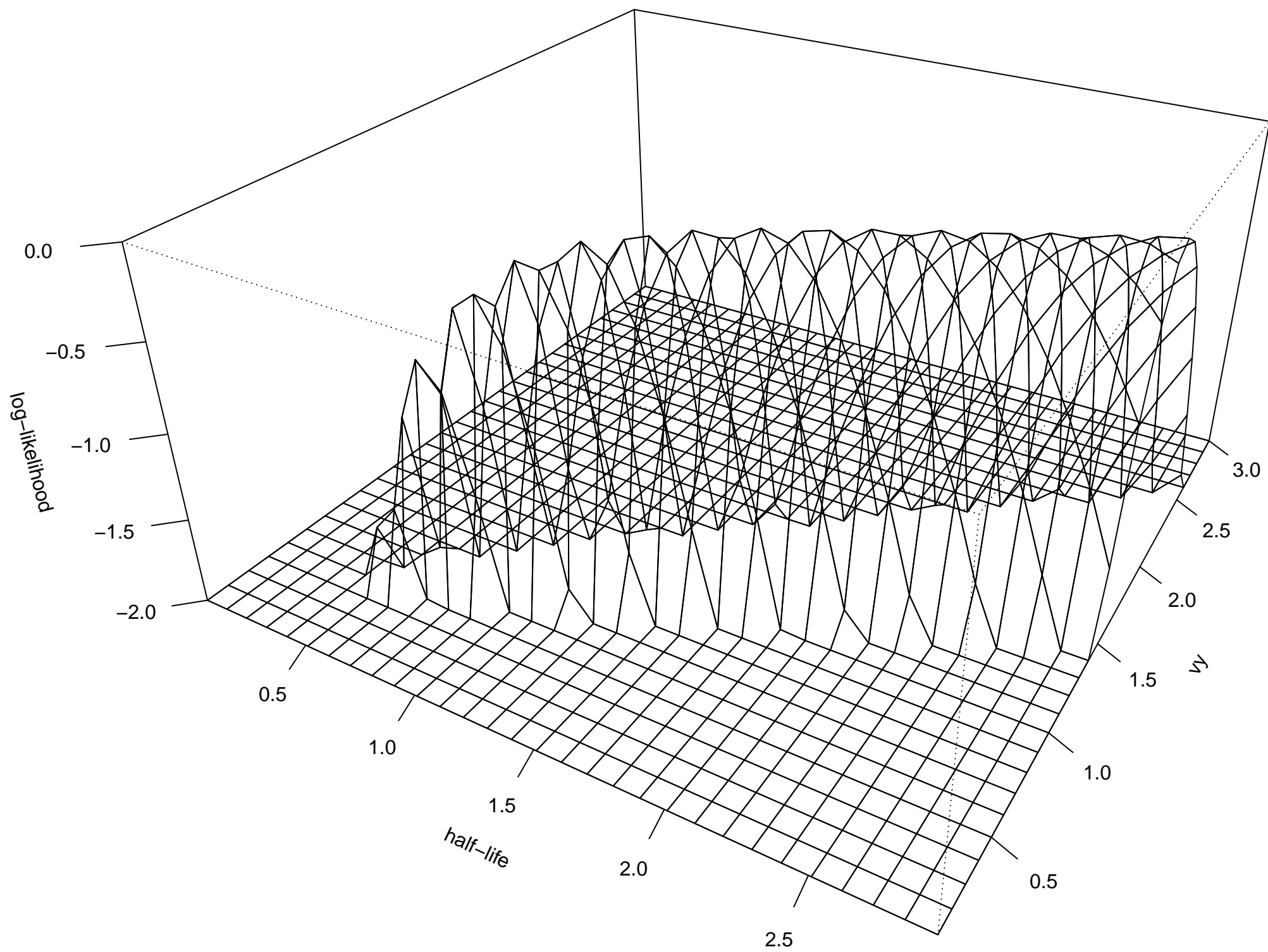

Supplement: Additional file 1: — All phylogenies used in analyses. R script for data extraction and analyses. Detailed results/raw output from SLOUCH. SLOUCH input data. Likelihood plots for all half-life estimations. (ZIP 2442 kb) [file 12862_2016_778_MOESM1_ESM.zip › Additional file 1/Results Bergman's rule - body mass/Phyllostomidae_phySig.pdf]

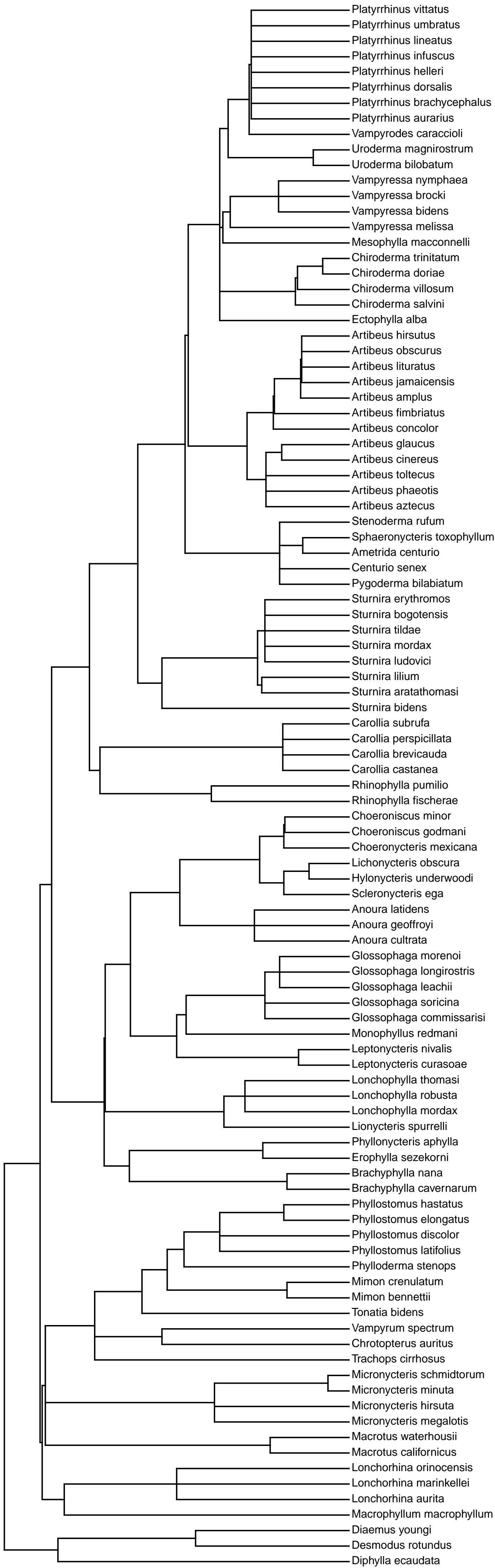

Supplement: Additional file 1: — All phylogenies used in analyses. R script for data extraction and analyses. Detailed results/raw output from SLOUCH. SLOUCH input data. Likelihood plots for all half-life estimations. (ZIP 2442 kb) [file 12862_2016_778_MOESM1_ESM.zip › Additional file 1/Results Bergman's rule - body mass/Phyllostomidae_tree.pdf]

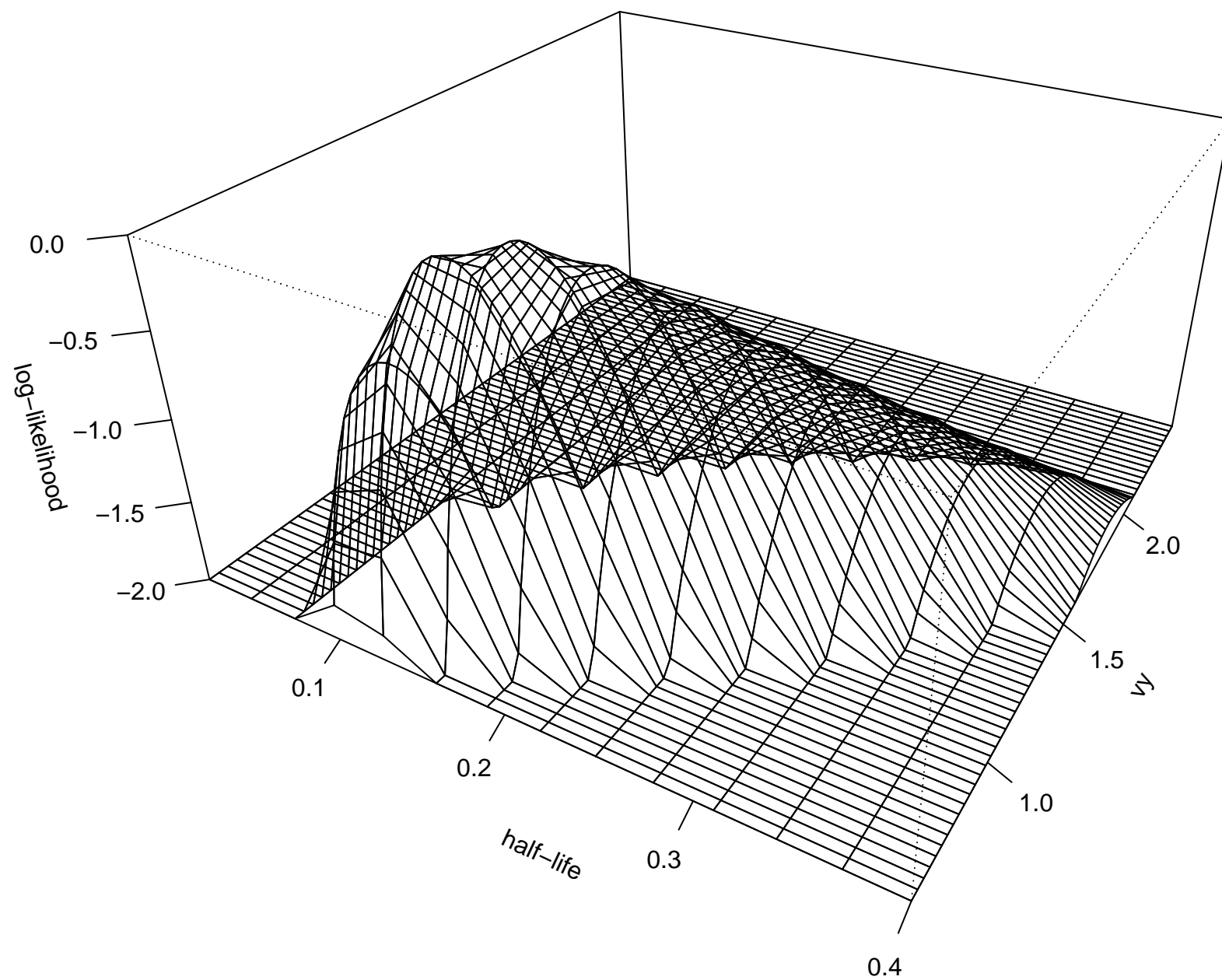

Supplement: Additional file 1: — All phylogenies used in analyses. R script for data extraction and analyses. Detailed results/raw output from SLOUCH. SLOUCH input data. Likelihood plots for all half-life estimations. (ZIP 2442 kb) [file 12862_2016_778_MOESM1_ESM.zip › Additional file 1/Results Bergman's rule - body mass/Pteropodidae_BM_maxlat.pdf]

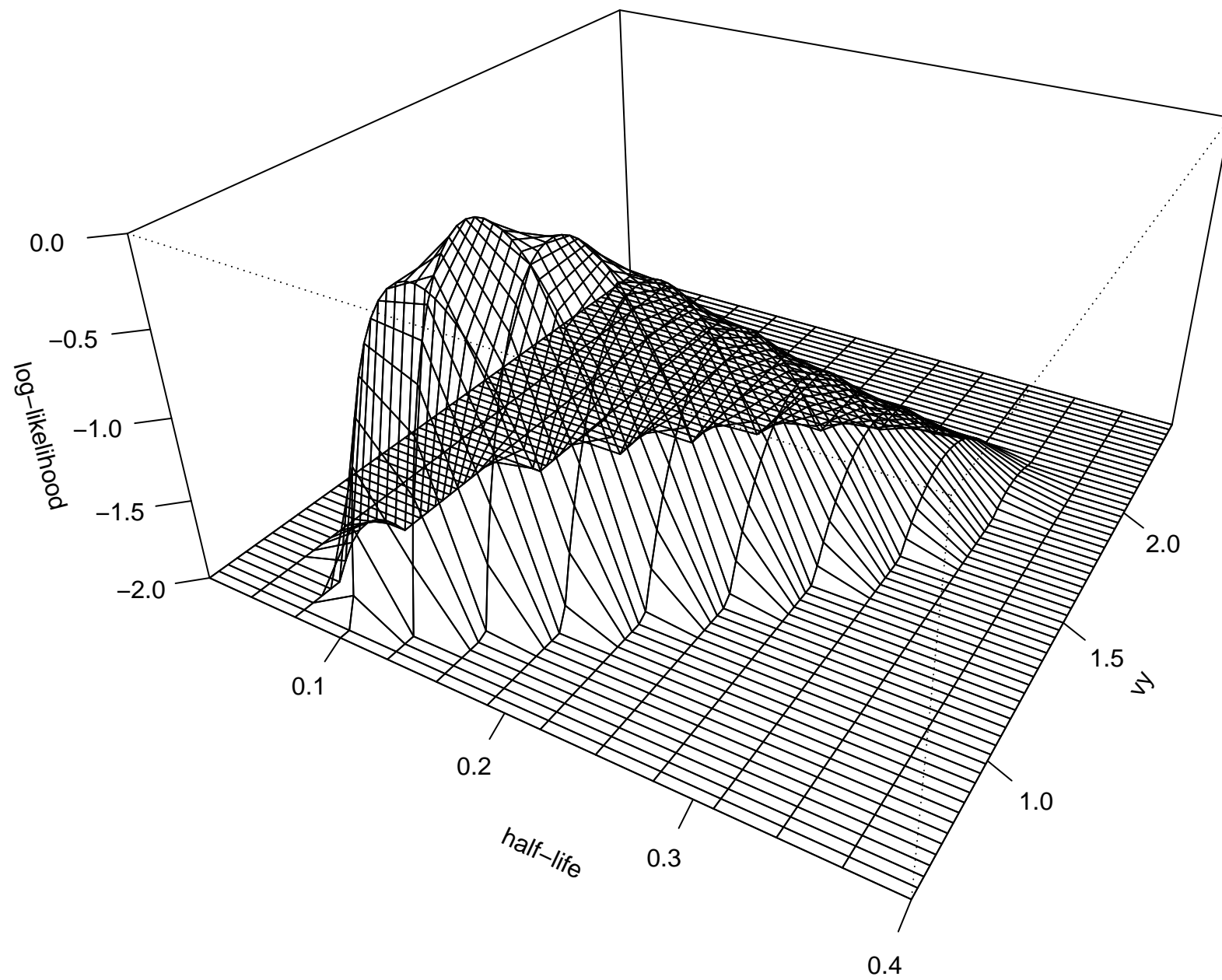

Supplement: Additional file 1: — All phylogenies used in analyses. R script for data extraction and analyses. Detailed results/raw output from SLOUCH. SLOUCH input data. Likelihood plots for all half-life estimations. (ZIP 2442 kb) [file 12862_2016_778_MOESM1_ESM.zip › Additional file 1/Results Bergman's rule - body mass/Pteropodidae_BM_midlat.pdf]

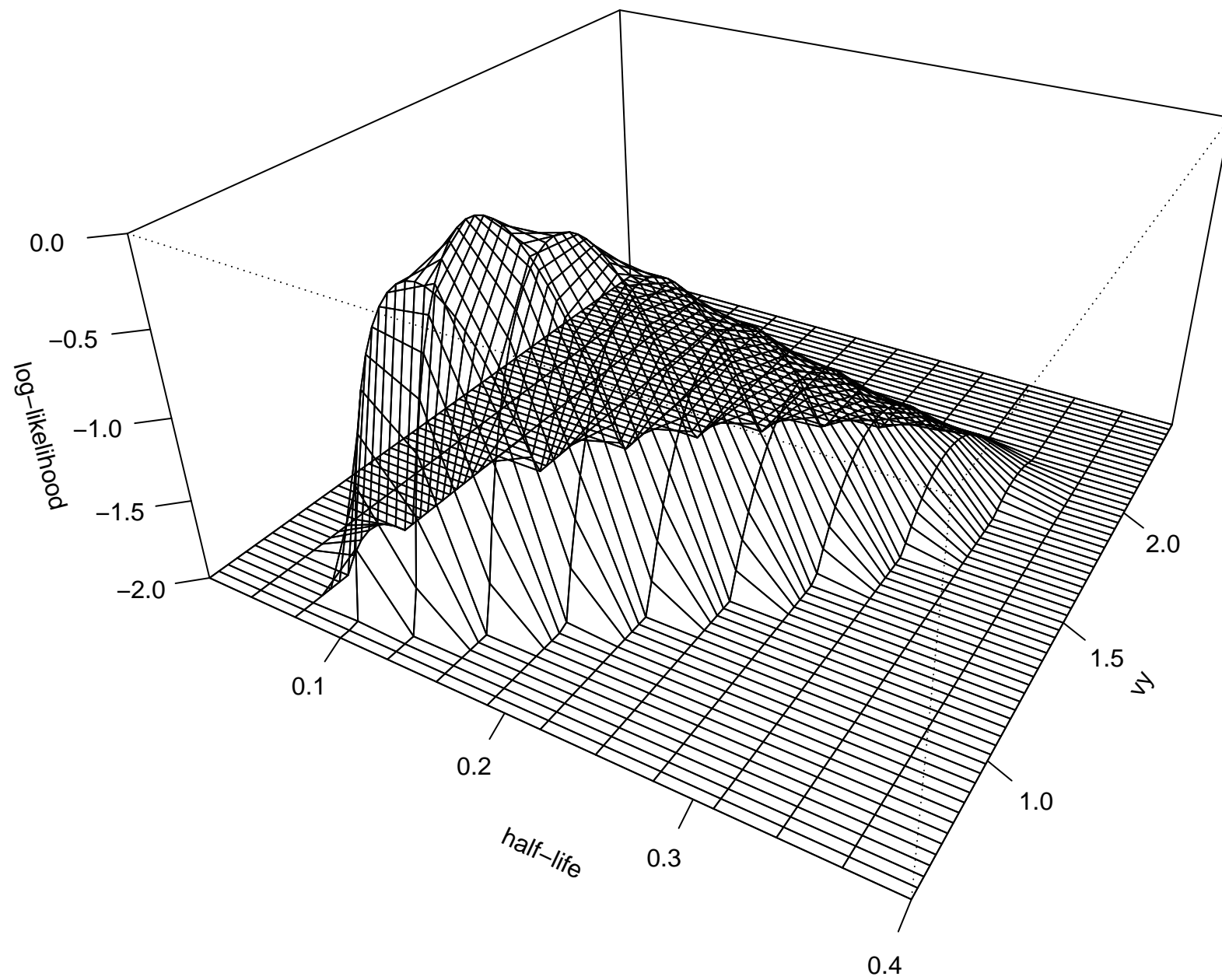

Supplement: Additional file 1: — All phylogenies used in analyses. R script for data extraction and analyses. Detailed results/raw output from SLOUCH. SLOUCH input data. Likelihood plots for all half-life estimations. (ZIP 2442 kb) [file 12862_2016_778_MOESM1_ESM.zip › Additional file 1/Results Bergman's rule - body mass/Pteropodidae_BM_temp.pdf]

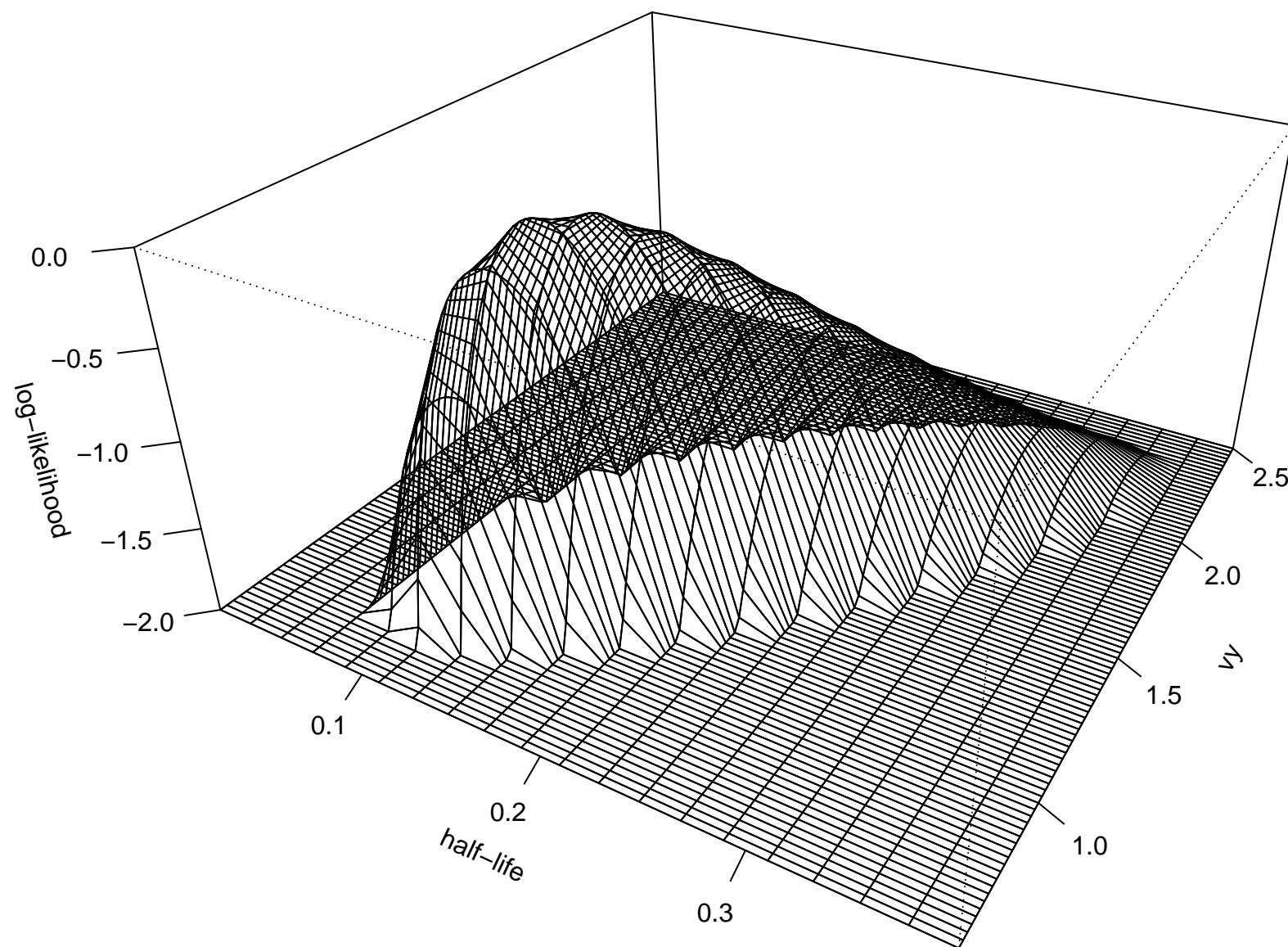

Supplement: Additional file 1: — All phylogenies used in analyses. R script for data extraction and analyses. Detailed results/raw output from SLOUCH. SLOUCH input data. Likelihood plots for all half-life estimations. (ZIP 2442 kb) [file 12862_2016_778_MOESM1_ESM.zip › Additional file 1/Results Bergman's rule - body mass/Pteropodidae_phySig.pdf]

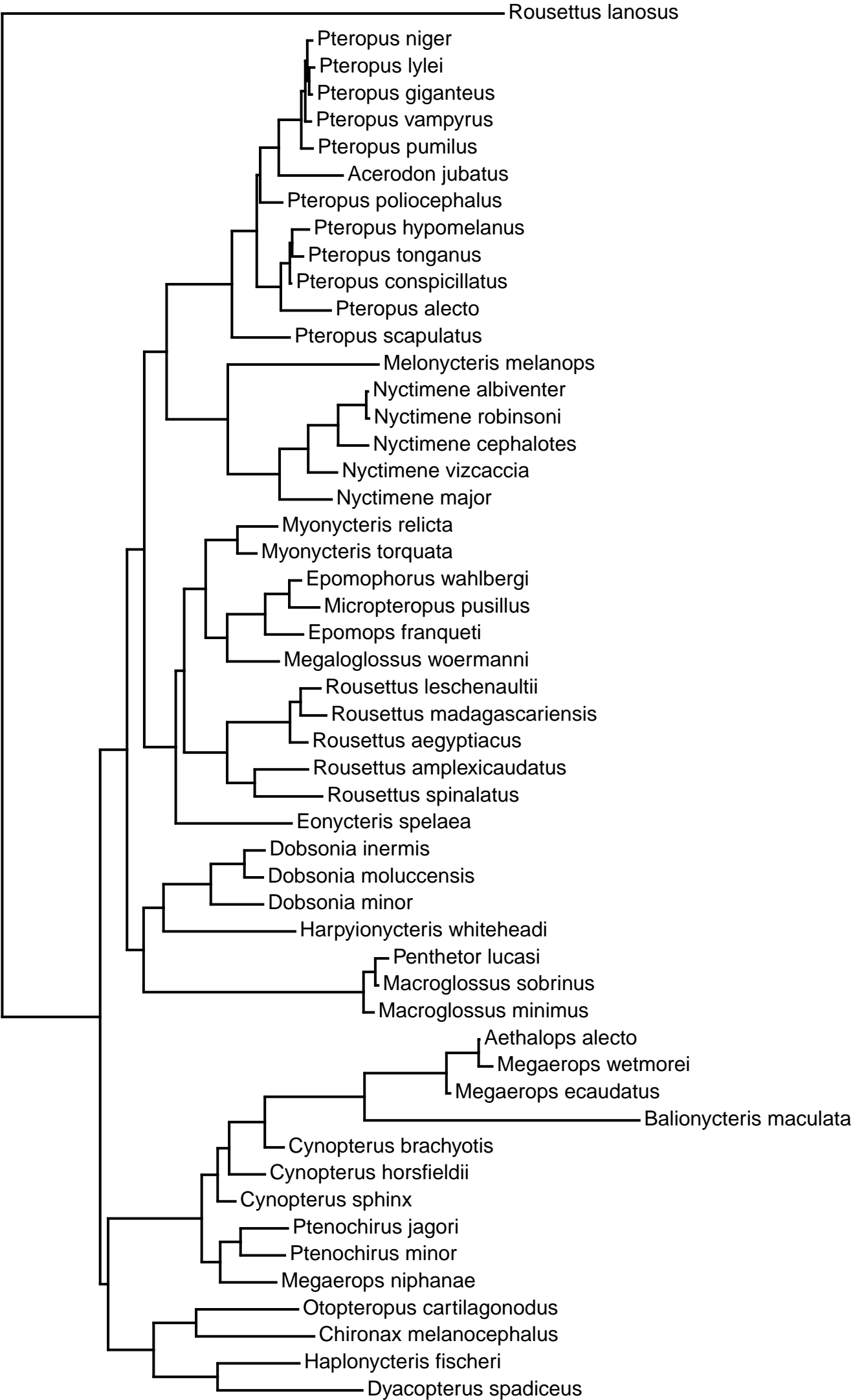

Supplement: Additional file 1: — All phylogenies used in analyses. R script for data extraction and analyses. Detailed results/raw output from SLOUCH. SLOUCH input data. Likelihood plots for all half-life estimations. (ZIP 2442 kb) [file 12862_2016_778_MOESM1_ESM.zip › Additional file 1/Results Bergman's rule - body mass/Pteropodidae_tree.pdf]

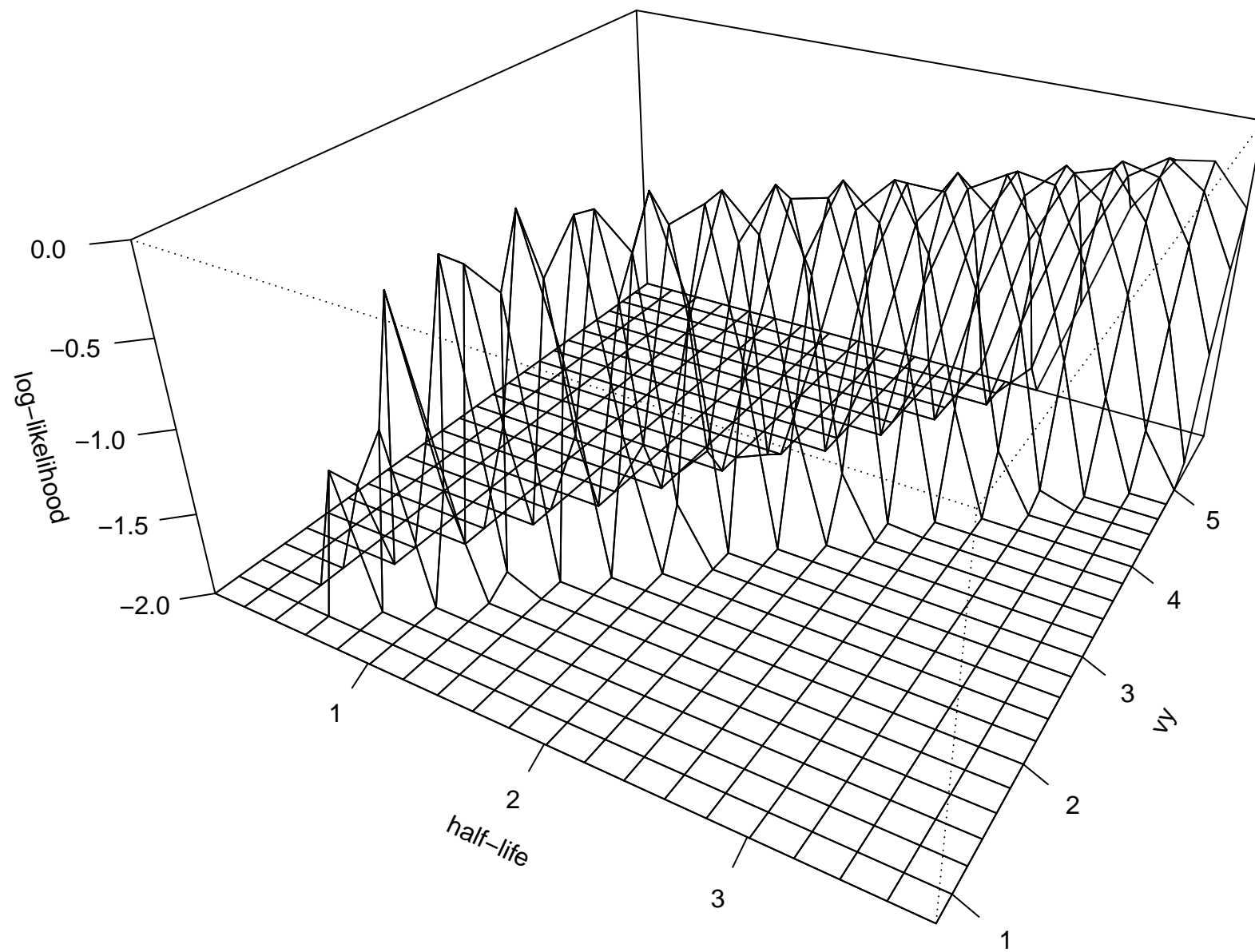

Supplement: Additional file 1: — All phylogenies used in analyses. R script for data extraction and analyses. Detailed results/raw output from SLOUCH. SLOUCH input data. Likelihood plots for all half-life estimations. (ZIP 2442 kb) [file 12862_2016_778_MOESM1_ESM.zip › Additional file 1/Results Bergman's rule - body mass/Sciuridae_BM_maxlat.pdf]

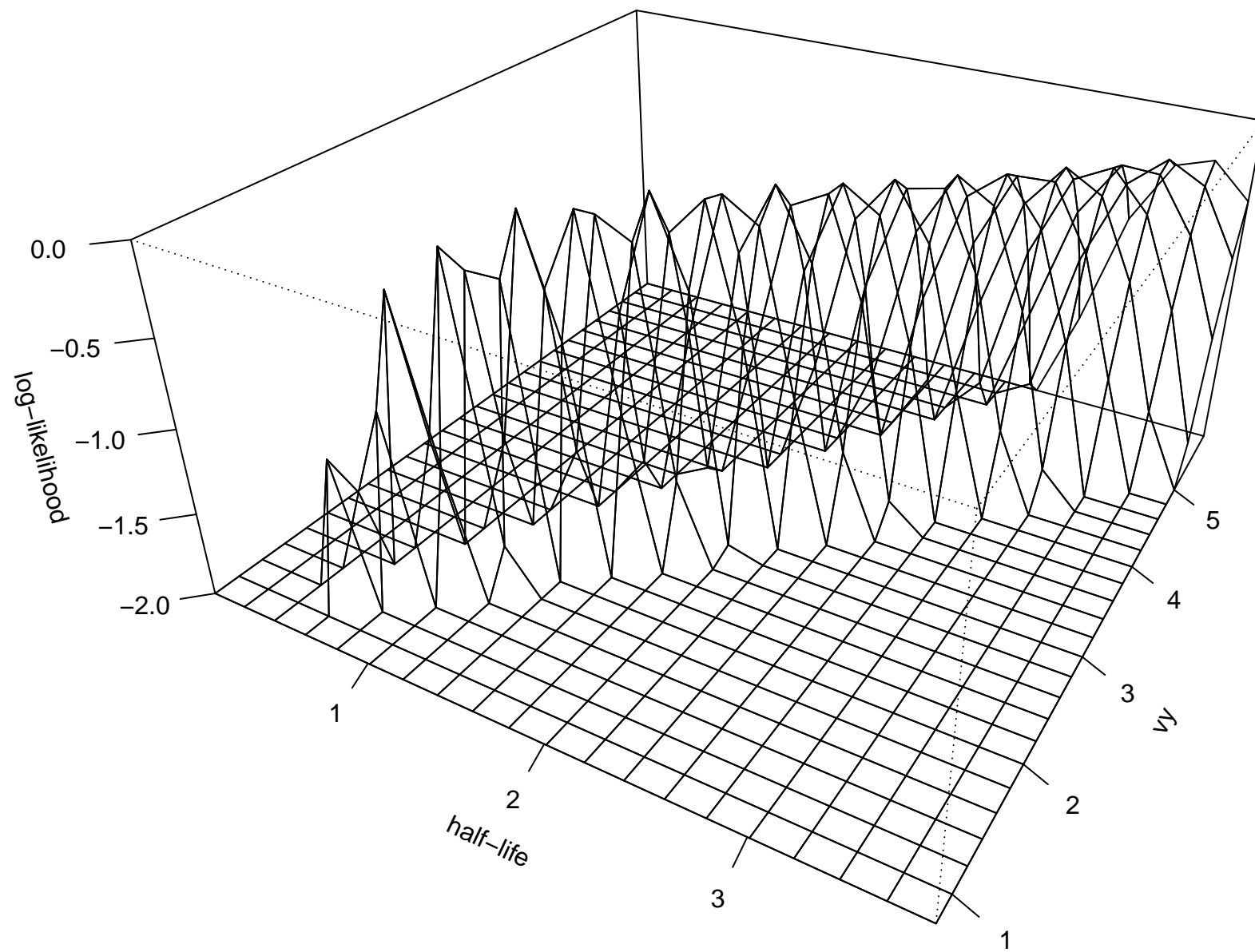

Supplement: Additional file 1: — All phylogenies used in analyses. R script for data extraction and analyses. Detailed results/raw output from SLOUCH. SLOUCH input data. Likelihood plots for all half-life estimations. (ZIP 2442 kb) [file 12862_2016_778_MOESM1_ESM.zip › Additional file 1/Results Bergman's rule - body mass/Sciuridae_BM_midlat.pdf]

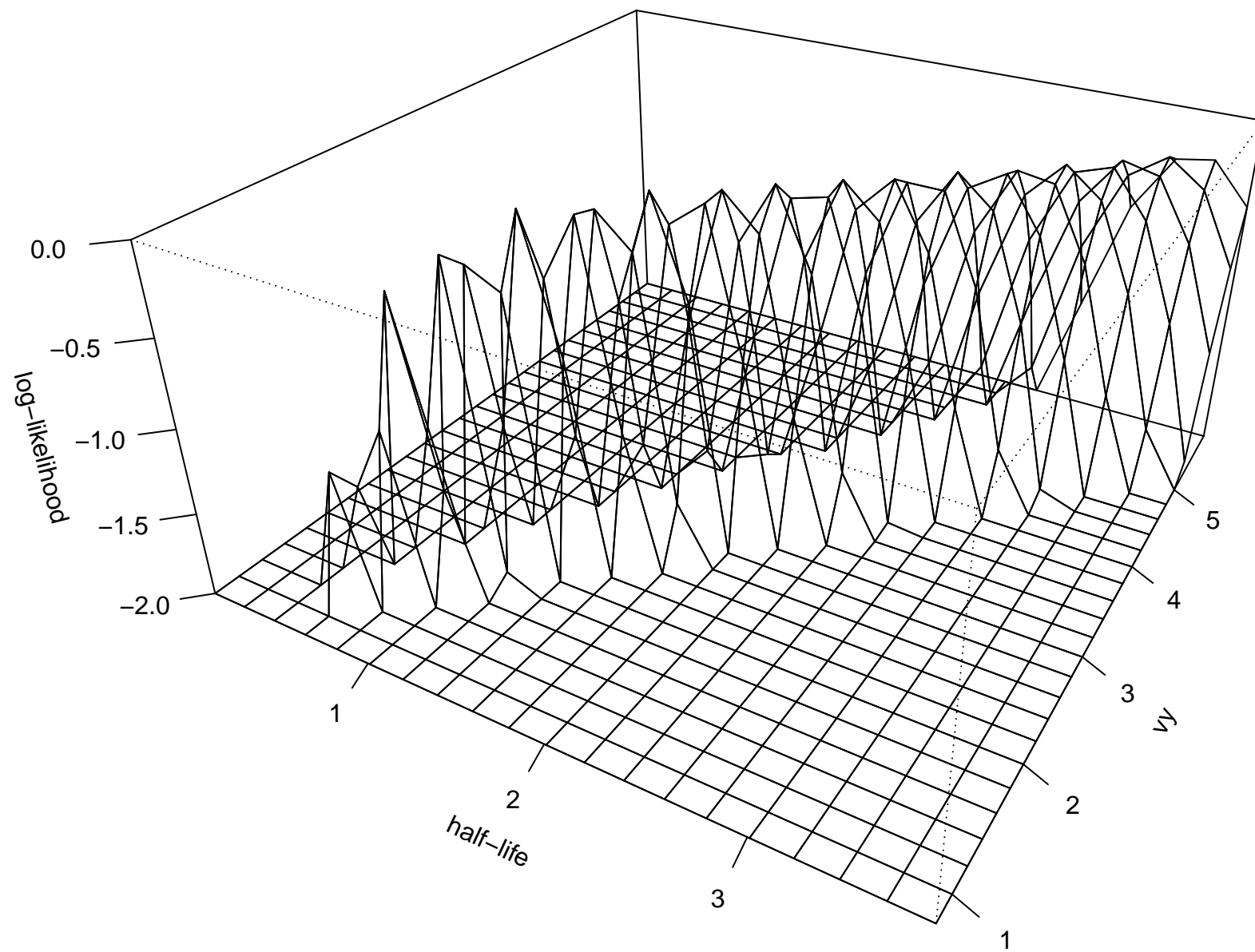

Supplement: Additional file 1: — All phylogenies used in analyses. R script for data extraction and analyses. Detailed results/raw output from SLOUCH. SLOUCH input data. Likelihood plots for all half-life estimations. (ZIP 2442 kb) [file 12862_2016_778_MOESM1_ESM.zip › Additional file 1/Results Bergman's rule - body mass/Sciuridae_BM_temp.pdf]

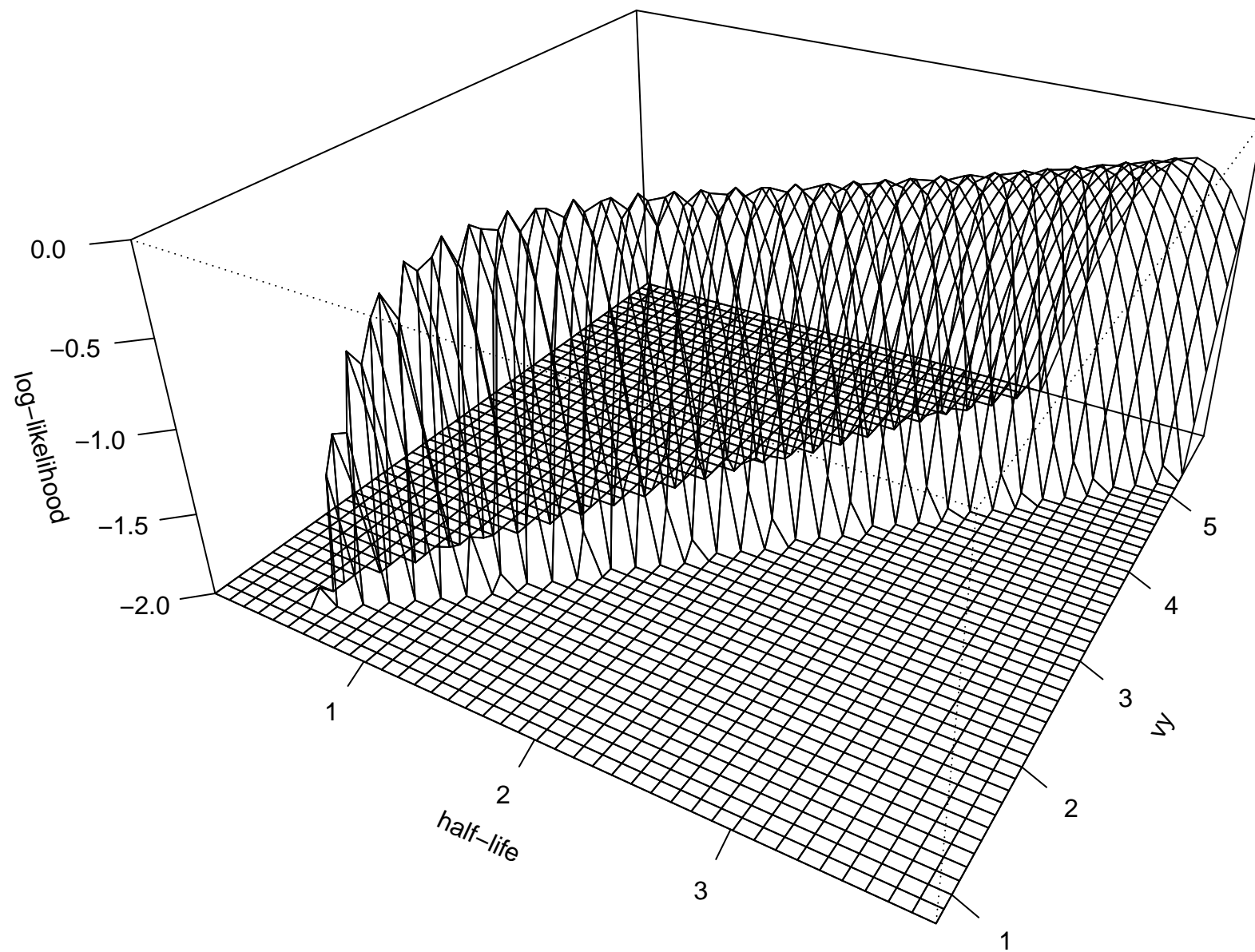

Supplement: Additional file 1: — All phylogenies used in analyses. R script for data extraction and analyses. Detailed results/raw output from SLOUCH. SLOUCH input data. Likelihood plots for all half-life estimations. (ZIP 2442 kb) [file 12862_2016_778_MOESM1_ESM.zip › Additional file 1/Results Bergman's rule - body mass/Sciuridae_phySig.pdf]

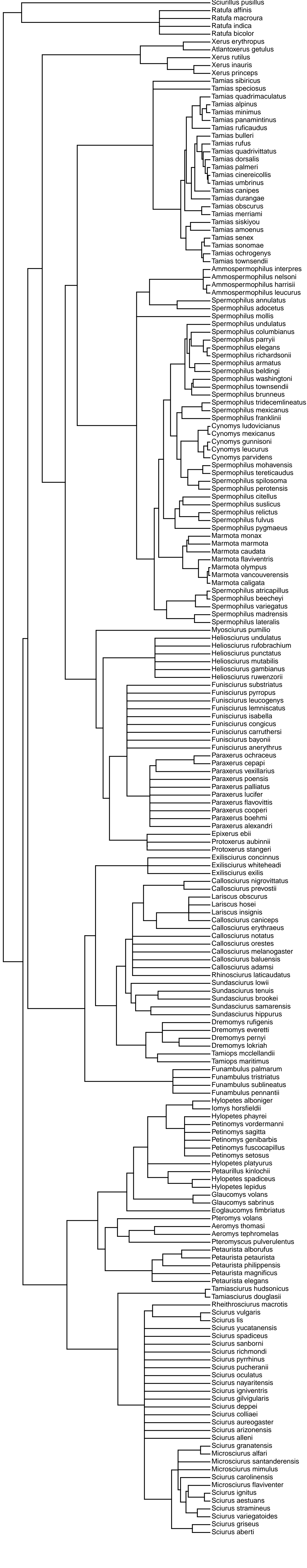

Supplement: Additional file 1: — All phylogenies used in analyses. R script for data extraction and analyses. Detailed results/raw output from SLOUCH. SLOUCH input data. Likelihood plots for all half-life estimations. (ZIP 2442 kb) [file 12862_2016_778_MOESM1_ESM.zip › Additional file 1/Results Bergman's rule - body mass/Sciuridae_tree.pdf]

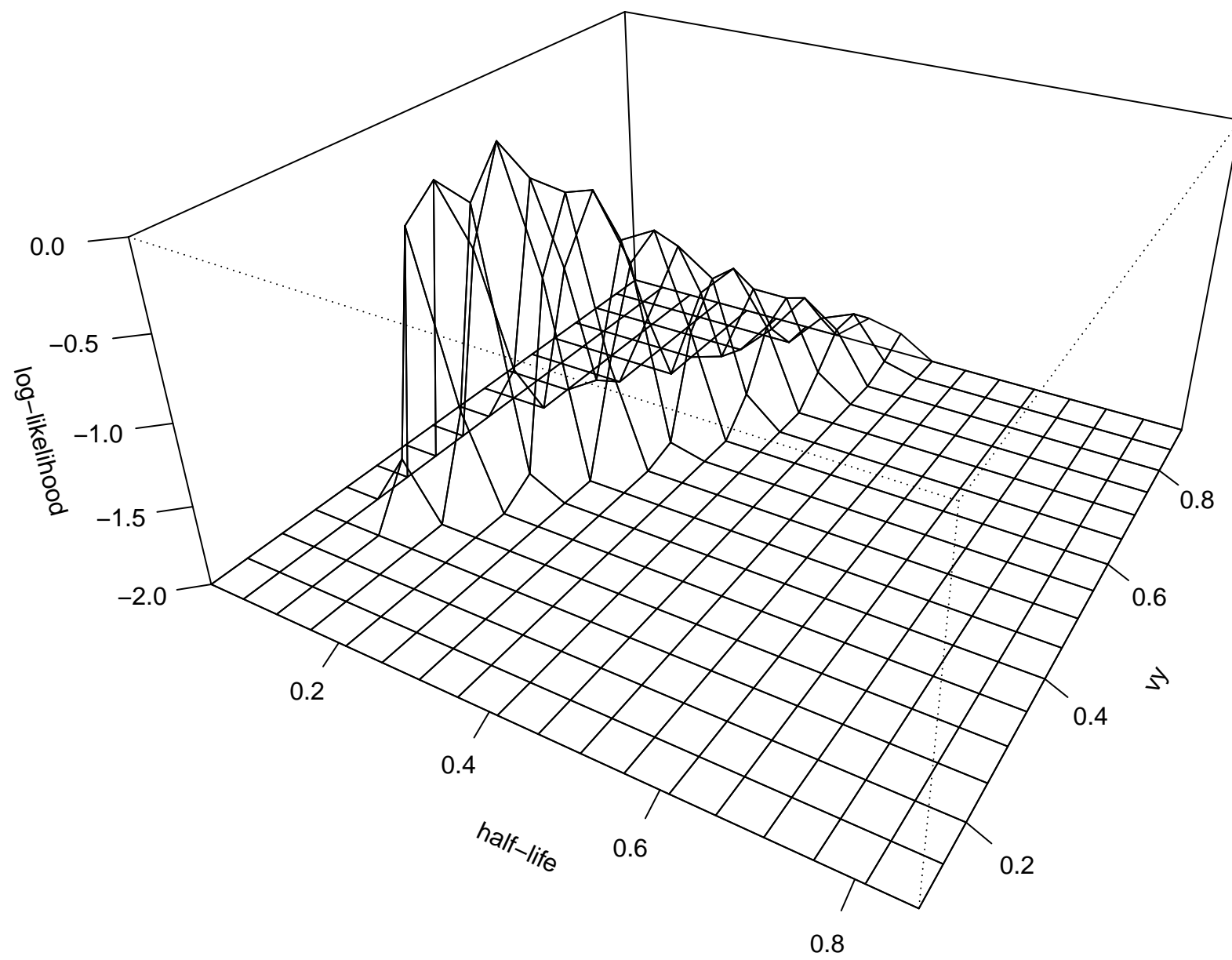

Supplement: Additional file 1: — All phylogenies used in analyses. R script for data extraction and analyses. Detailed results/raw output from SLOUCH. SLOUCH input data. Likelihood plots for all half-life estimations. (ZIP 2442 kb) [file 12862_2016_778_MOESM1_ESM.zip › Additional file 1/Results Bergman's rule - body mass/Vespertilionidae_BM_maxlat.pdf]

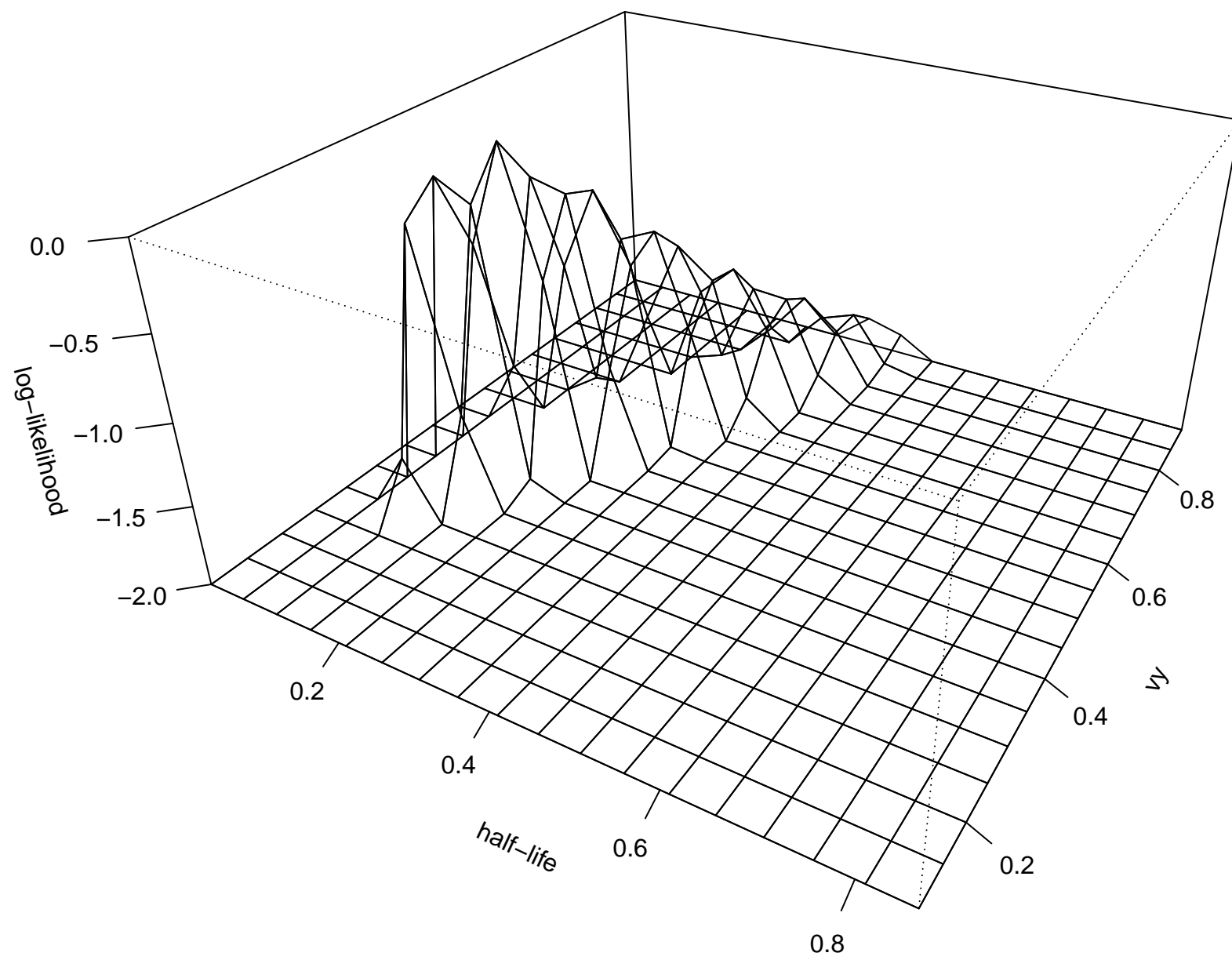

Supplement: Additional file 1: — All phylogenies used in analyses. R script for data extraction and analyses. Detailed results/raw output from SLOUCH. SLOUCH input data. Likelihood plots for all half-life estimations. (ZIP 2442 kb) [file 12862_2016_778_MOESM1_ESM.zip › Additional file 1/Results Bergman's rule - body mass/Vespertilionidae_BM_midlat.pdf]

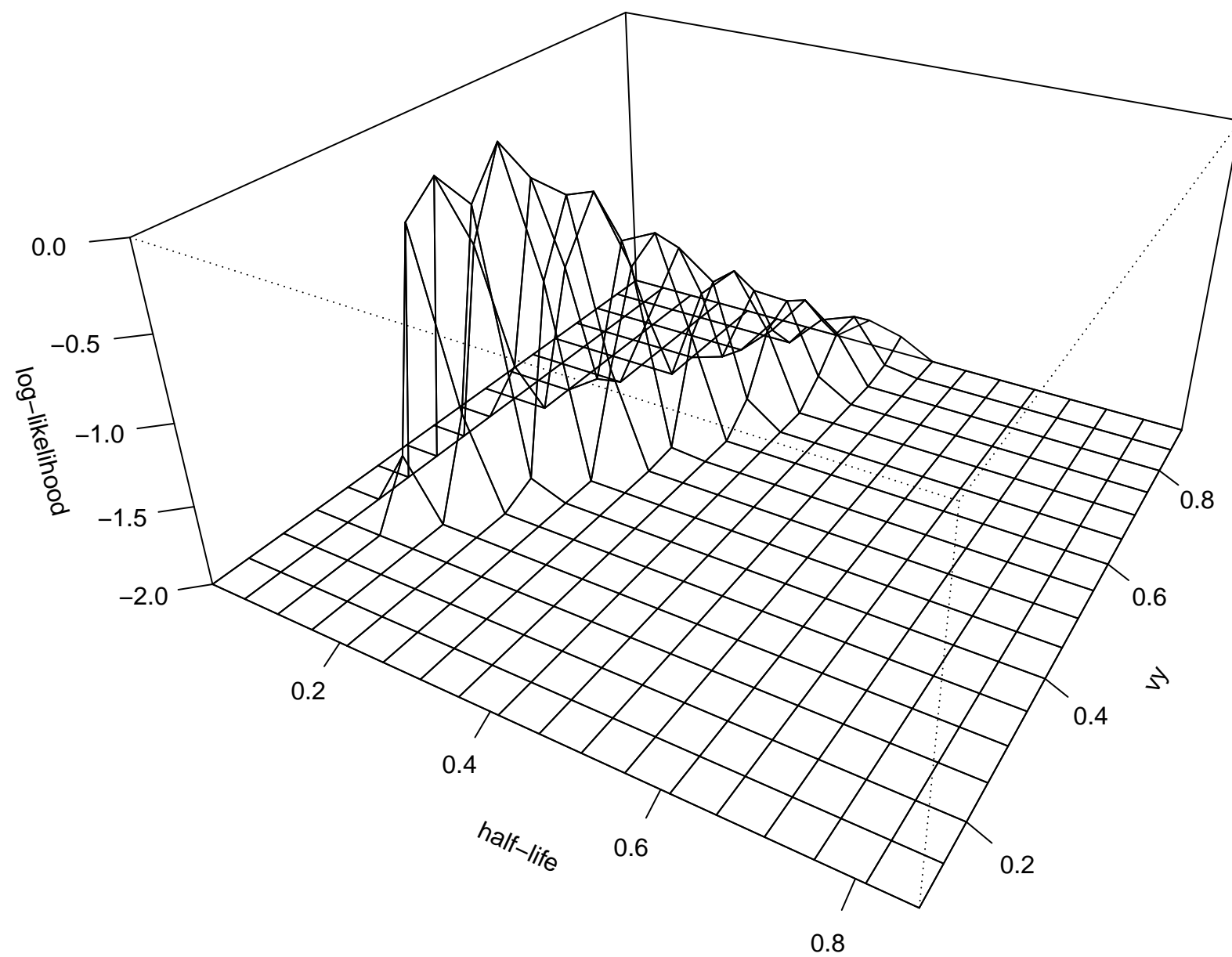

Supplement: Additional file 1: — All phylogenies used in analyses. R script for data extraction and analyses. Detailed results/raw output from SLOUCH. SLOUCH input data. Likelihood plots for all half-life estimations. (ZIP 2442 kb) [file 12862_2016_778_MOESM1_ESM.zip › Additional file 1/Results Bergman's rule - body mass/Vespertilionidae_BM_temp.pdf]

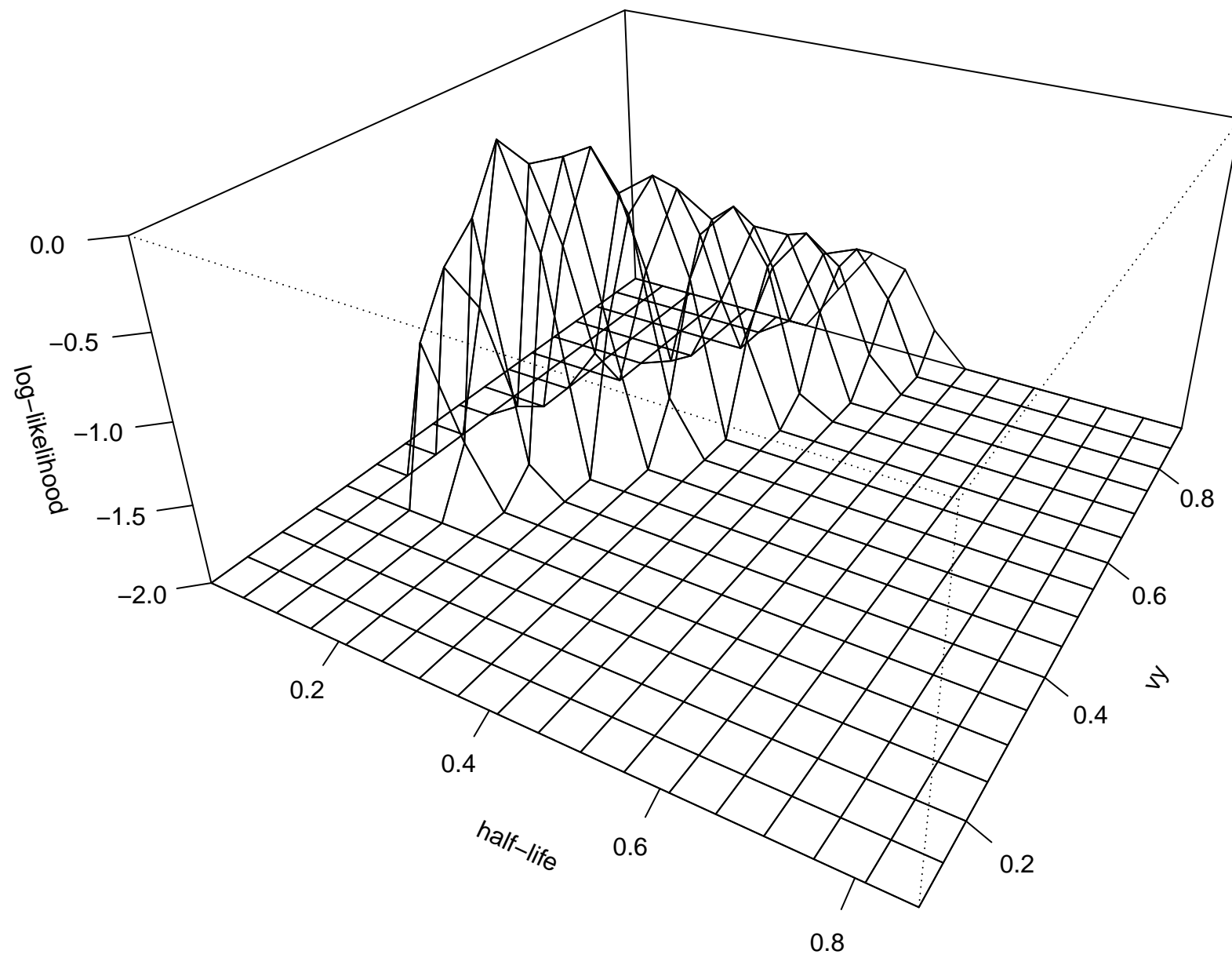

Supplement: Additional file 1: — All phylogenies used in analyses. R script for data extraction and analyses. Detailed results/raw output from SLOUCH. SLOUCH input data. Likelihood plots for all half-life estimations. (ZIP 2442 kb) [file 12862_2016_778_MOESM1_ESM.zip › Additional file 1/Results Bergman's rule - body mass/Vespertilionidae_phySig.pdf]

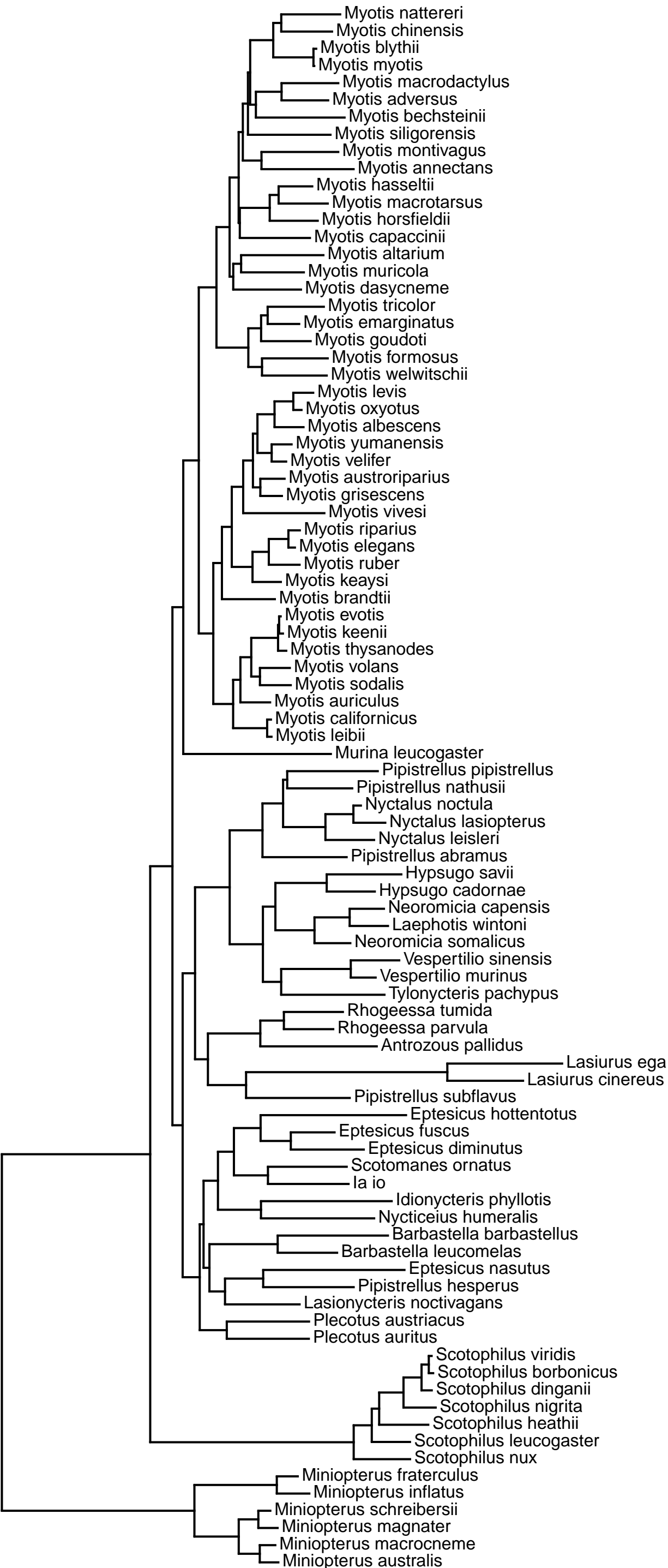

Supplement: Additional file 1: — All phylogenies used in analyses. R script for data extraction and analyses. Detailed results/raw output from SLOUCH. SLOUCH input data. Likelihood plots for all half-life estimations. (ZIP 2442 kb) [file 12862_2016_778_MOESM1_ESM.zip › Additional file 1/Results Bergman's rule - body mass/Vespertilionidae_tree.pdf]
