# Supplementary figures and images for: An interspecific assessment of Bergmann’s rule in 22 mammalian families (part 1 of 2)
Source: BMC Evol Biol. 2016 Oct 19;16:222. doi: 10.1186/s12862-016-0778-x (PMC5069937; doi:10.1186/s12862-016-0778-x)

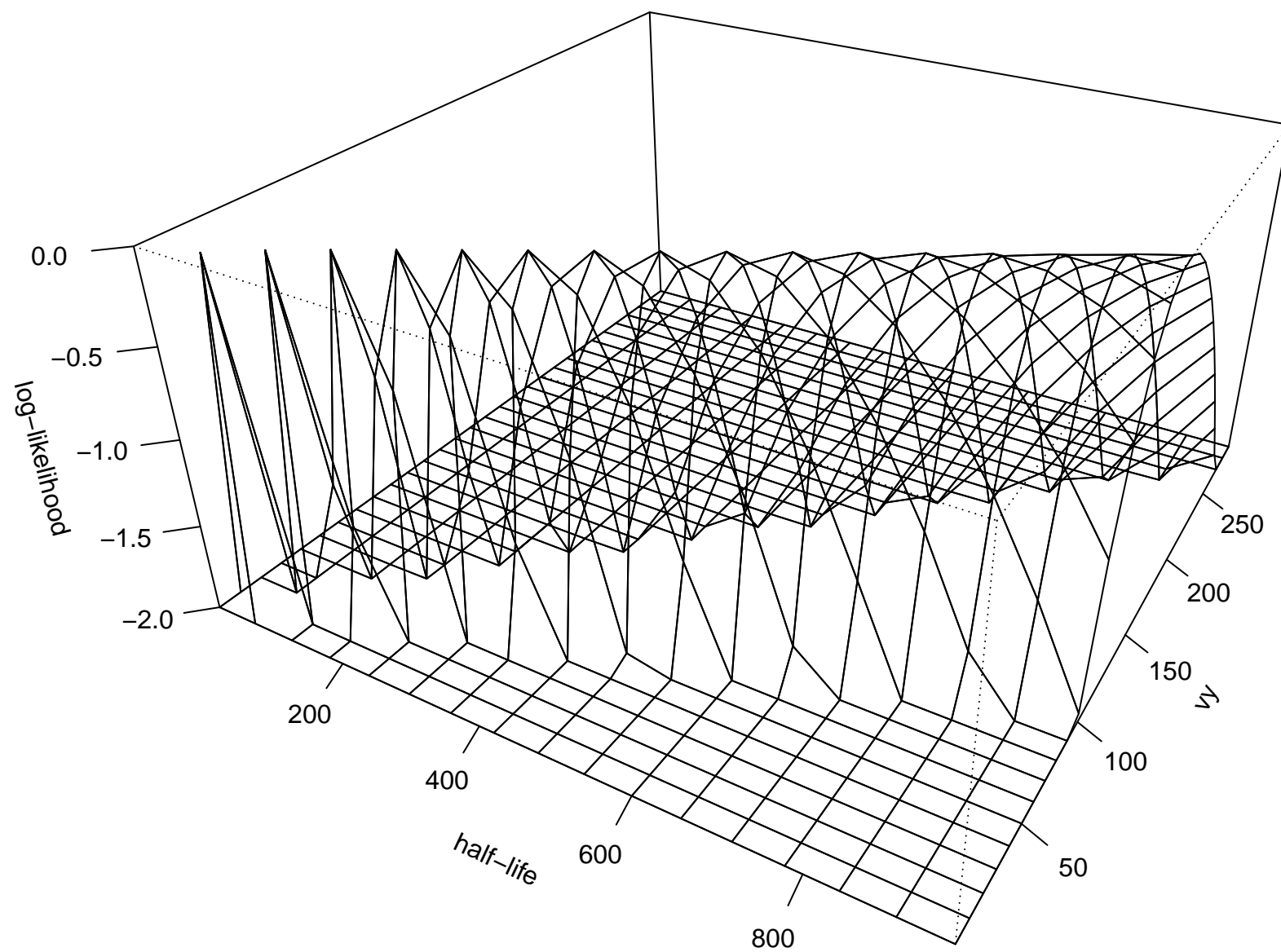

Supplement: Additional file 1: — All phylogenies used in analyses. R script for data extraction and analyses. Detailed results/raw output from SLOUCH. SLOUCH input data. Likelihood plots for all half-life estimations. (ZIP 2442 kb) [file 12862_2016_778_MOESM1_ESM.zip › Additional file 1/Results Allen's rule - forelimb length over body mass/Emballonuridae_LLBM_maxlat.pdf]

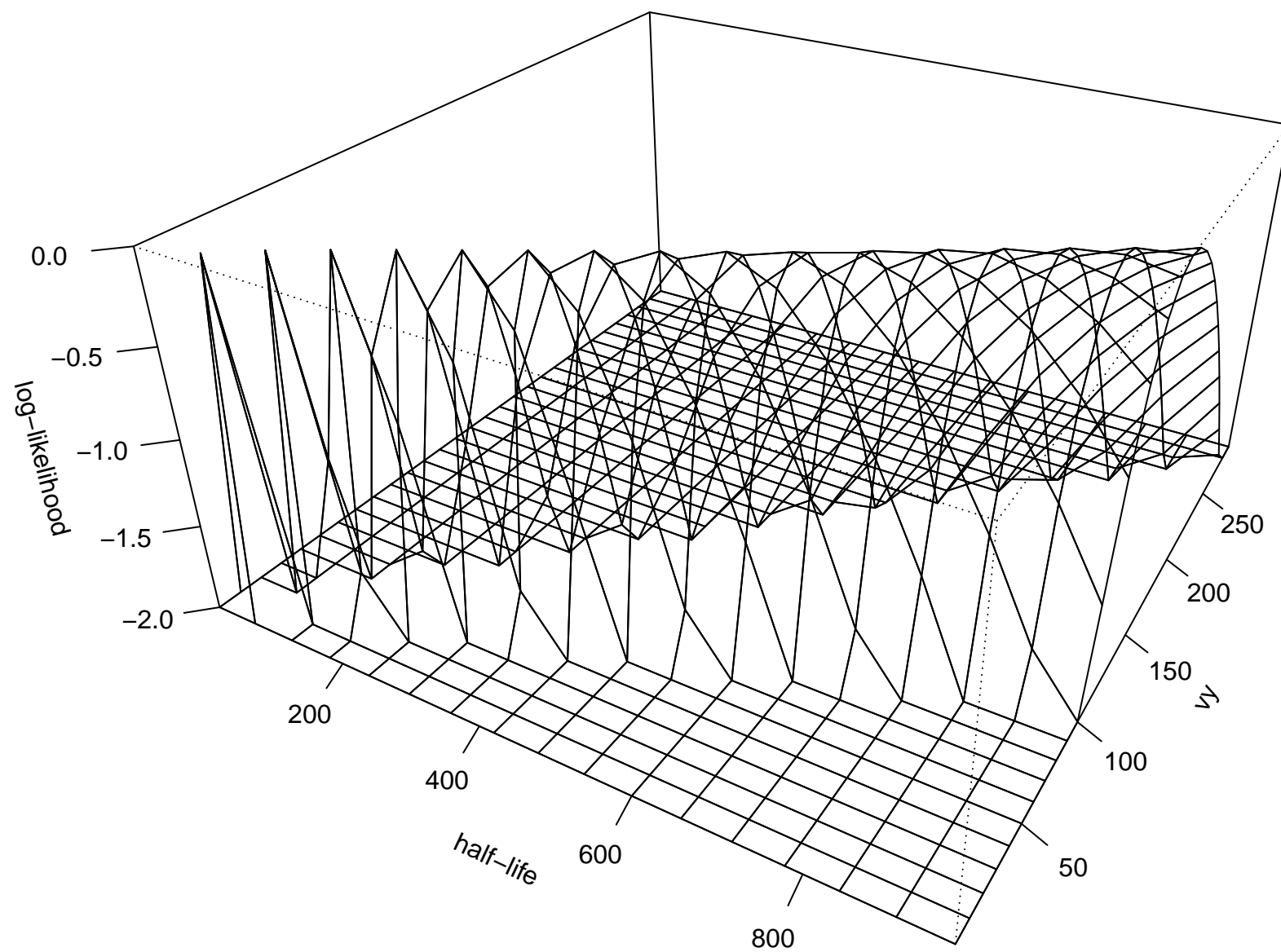

Supplement: Additional file 1: — All phylogenies used in analyses. R script for data extraction and analyses. Detailed results/raw output from SLOUCH. SLOUCH input data. Likelihood plots for all half-life estimations. (ZIP 2442 kb) [file 12862_2016_778_MOESM1_ESM.zip › Additional file 1/Results Allen's rule - forelimb length over body mass/Emballonuridae_LLBM_midlat.pdf]

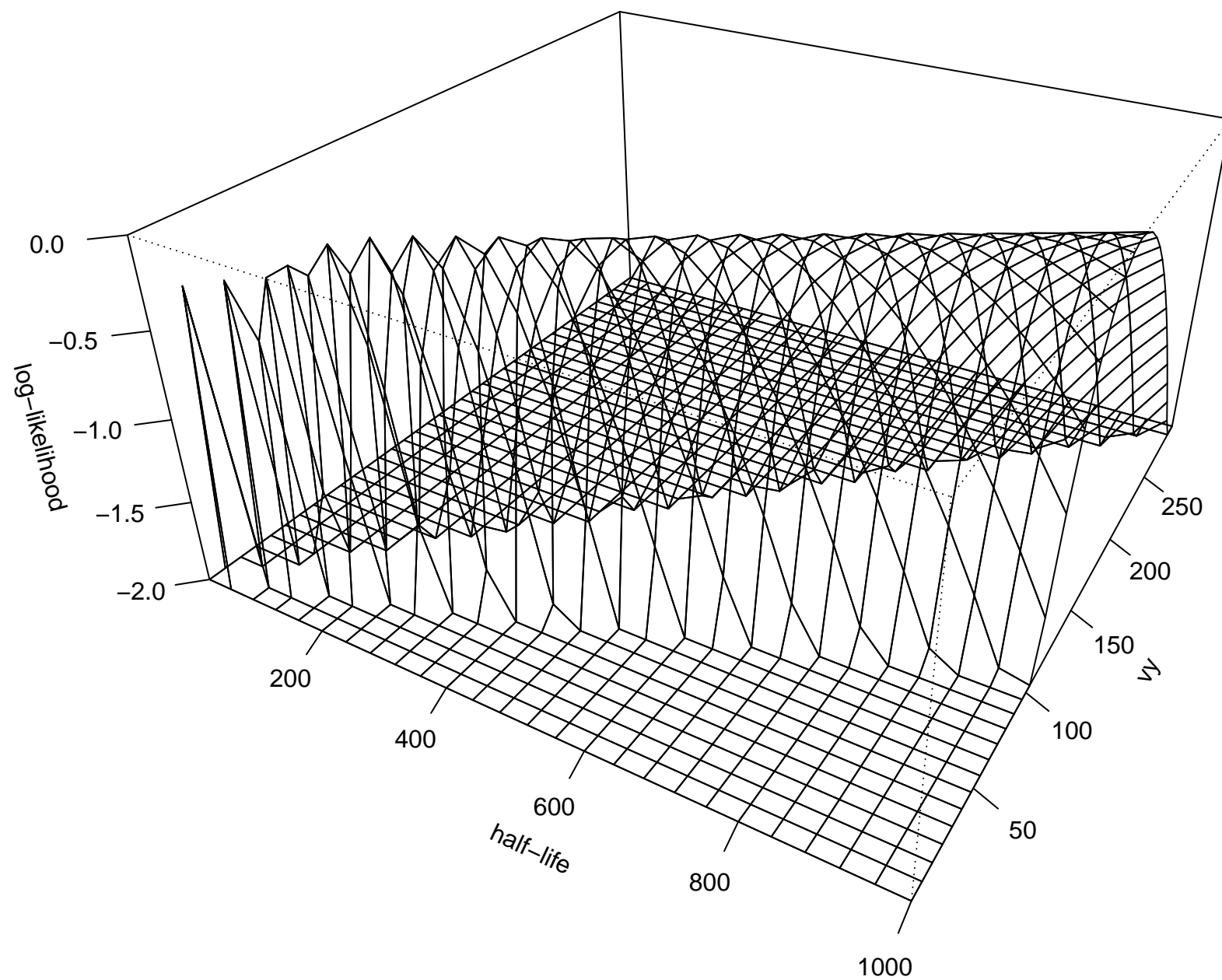

Supplement: Additional file 1: — All phylogenies used in analyses. R script for data extraction and analyses. Detailed results/raw output from SLOUCH. SLOUCH input data. Likelihood plots for all half-life estimations. (ZIP 2442 kb) [file 12862_2016_778_MOESM1_ESM.zip › Additional file 1/Results Allen's rule - forelimb length over body mass/Emballonuridae_LLBM_phySig.pdf]

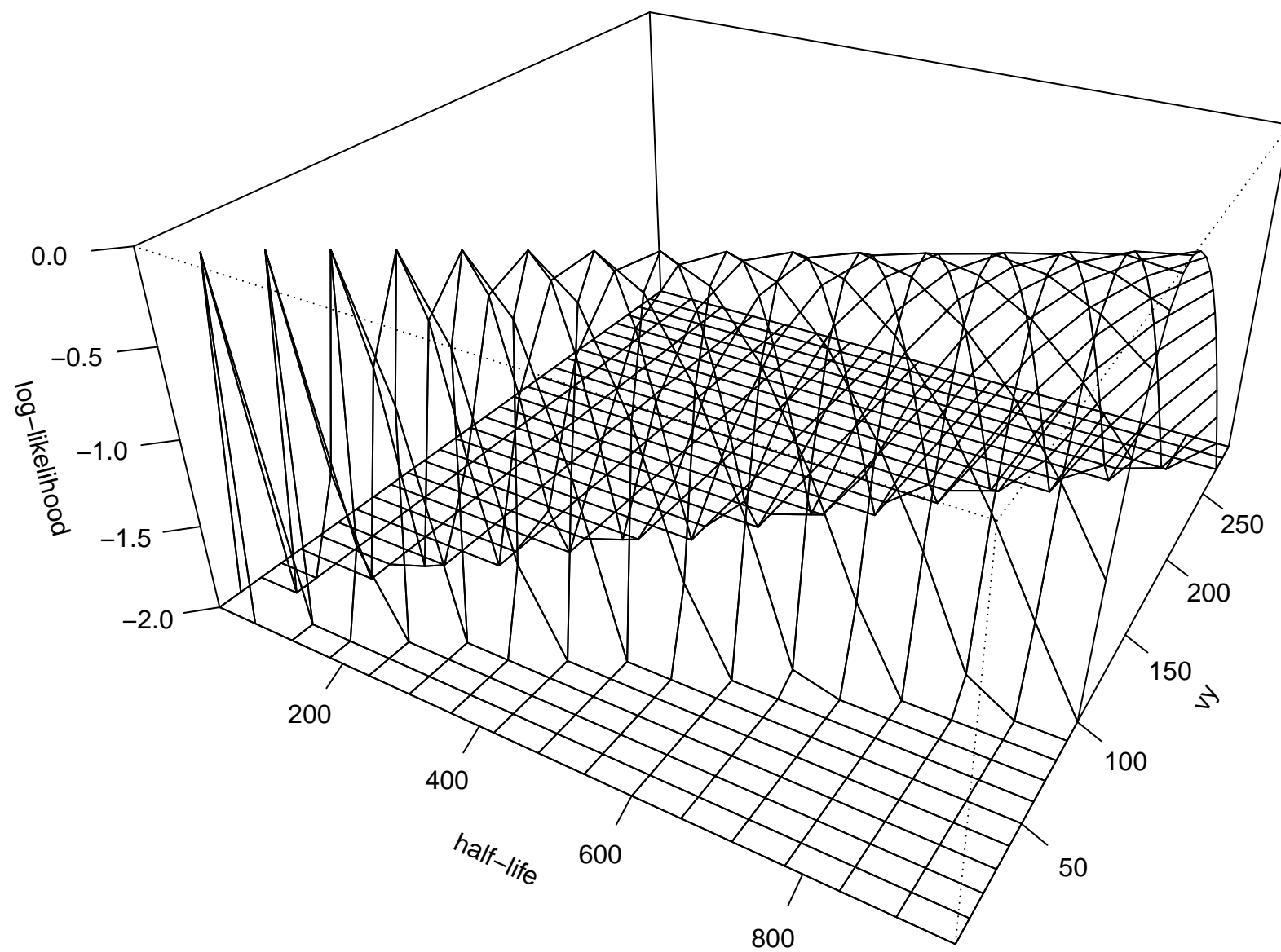

Supplement: Additional file 1: — All phylogenies used in analyses. R script for data extraction and analyses. Detailed results/raw output from SLOUCH. SLOUCH input data. Likelihood plots for all half-life estimations. (ZIP 2442 kb) [file 12862_2016_778_MOESM1_ESM.zip › Additional file 1/Results Allen's rule - forelimb length over body mass/Emballonuridae_LLBM_temp.pdf]

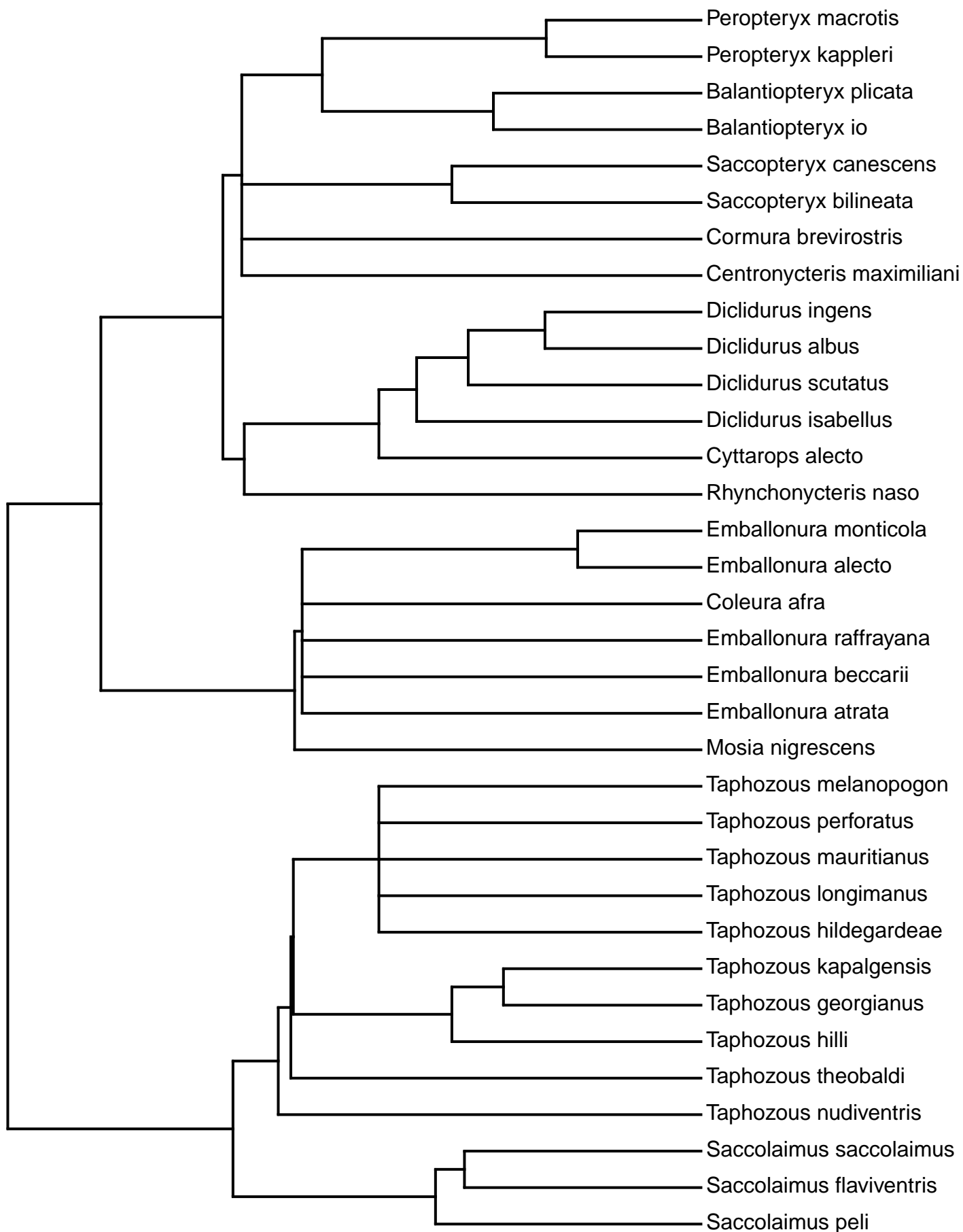

Supplement: Additional file 1: — All phylogenies used in analyses. R script for data extraction and analyses. Detailed results/raw output from SLOUCH. SLOUCH input data. Likelihood plots for all half-life estimations. (ZIP 2442 kb) [file 12862_2016_778_MOESM1_ESM.zip › Additional file 1/Results Allen's rule - forelimb length over body mass/Emballonuridae_tree.pdf]

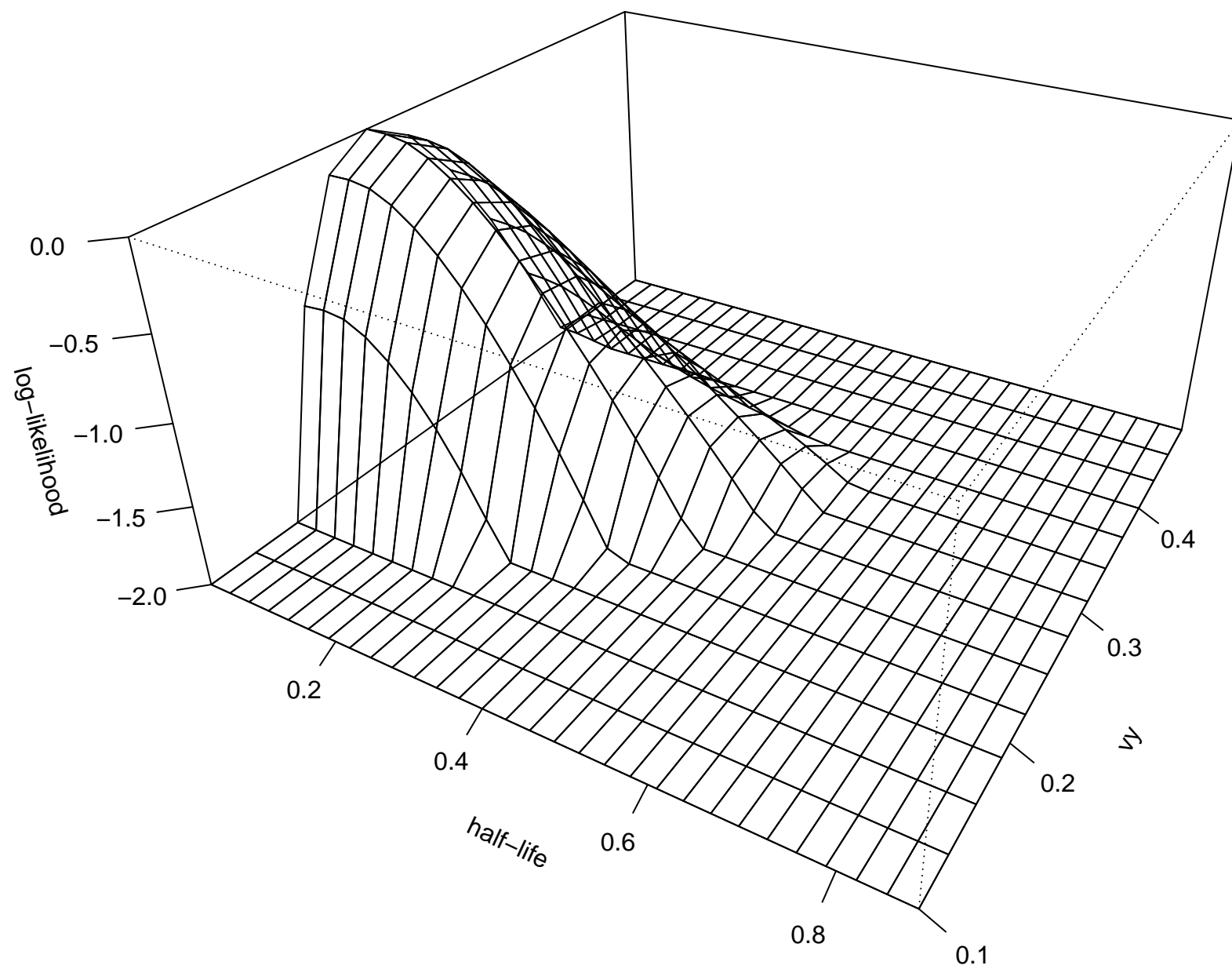

Supplement: Additional file 1: — All phylogenies used in analyses. R script for data extraction and analyses. Detailed results/raw output from SLOUCH. SLOUCH input data. Likelihood plots for all half-life estimations. (ZIP 2442 kb) [file 12862_2016_778_MOESM1_ESM.zip › Additional file 1/Results Allen's rule - forelimb length over body mass/Molossidae.pdf]

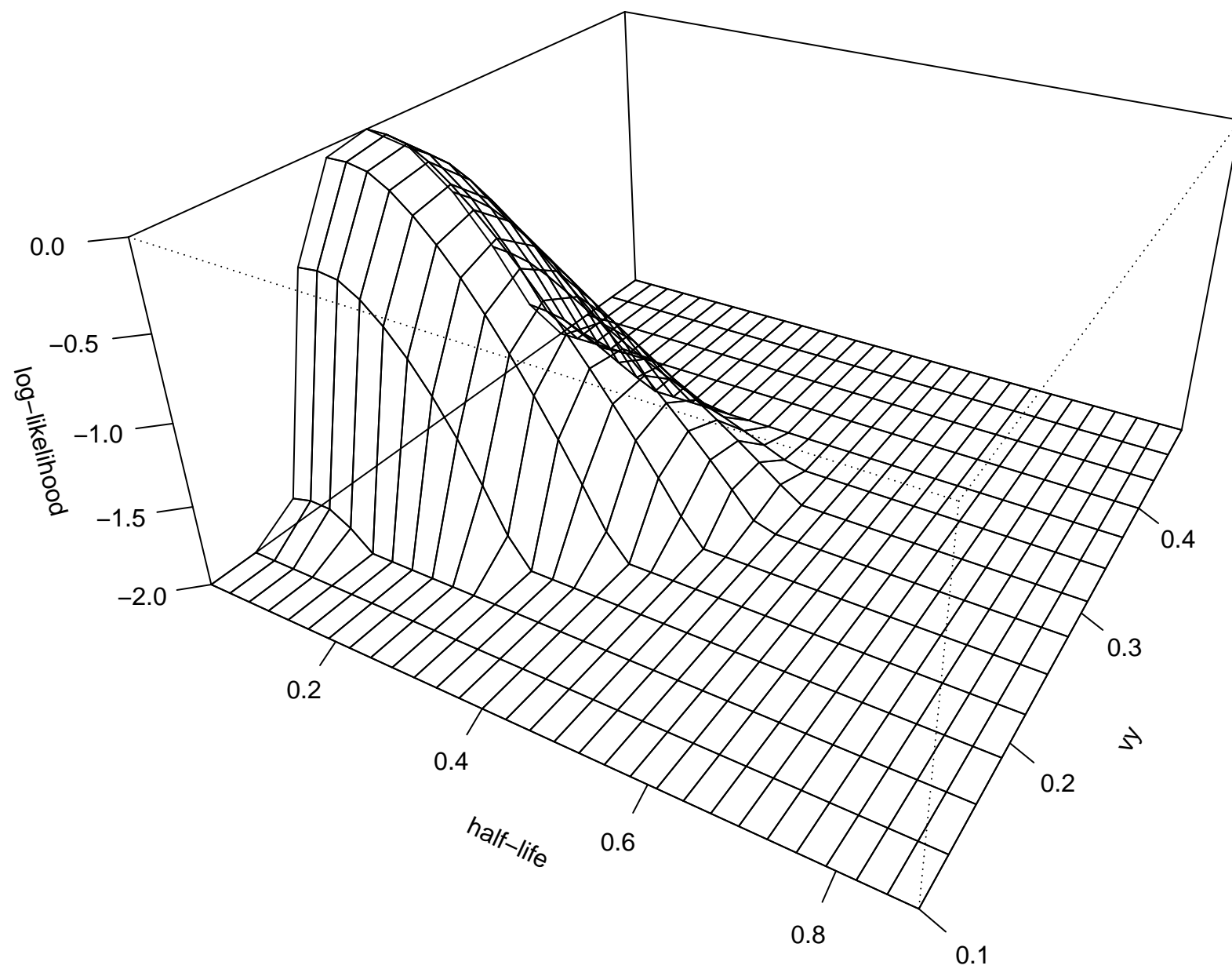

Supplement: Additional file 1: — All phylogenies used in analyses. R script for data extraction and analyses. Detailed results/raw output from SLOUCH. SLOUCH input data. Likelihood plots for all half-life estimations. (ZIP 2442 kb) [file 12862_2016_778_MOESM1_ESM.zip › Additional file 1/Results Allen's rule - forelimb length over body mass/Molossidae_LLBM_maxlat.pdf]

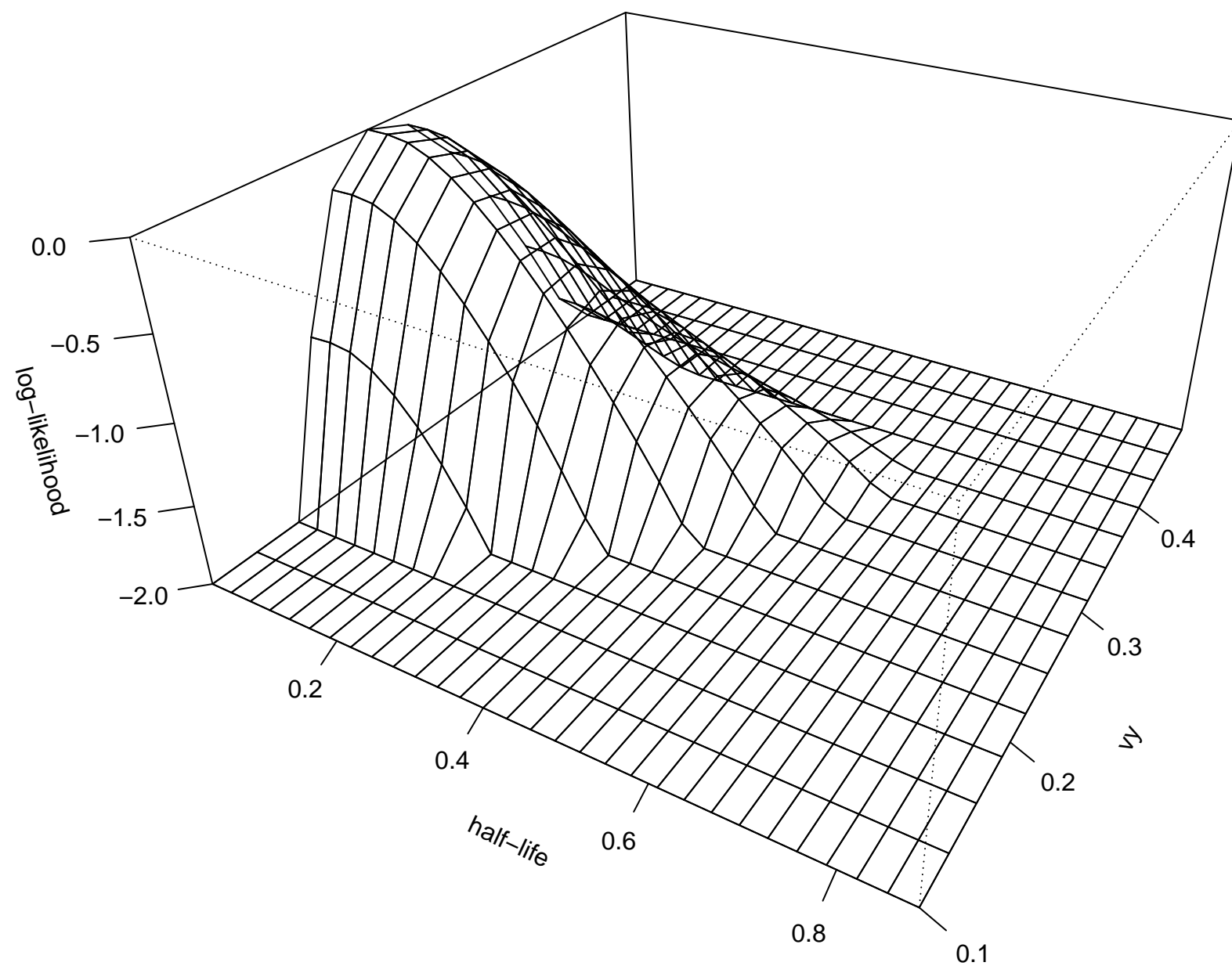

Supplement: Additional file 1: — All phylogenies used in analyses. R script for data extraction and analyses. Detailed results/raw output from SLOUCH. SLOUCH input data. Likelihood plots for all half-life estimations. (ZIP 2442 kb) [file 12862_2016_778_MOESM1_ESM.zip › Additional file 1/Results Allen's rule - forelimb length over body mass/Molossidae_LLBM_midlat.pdf]

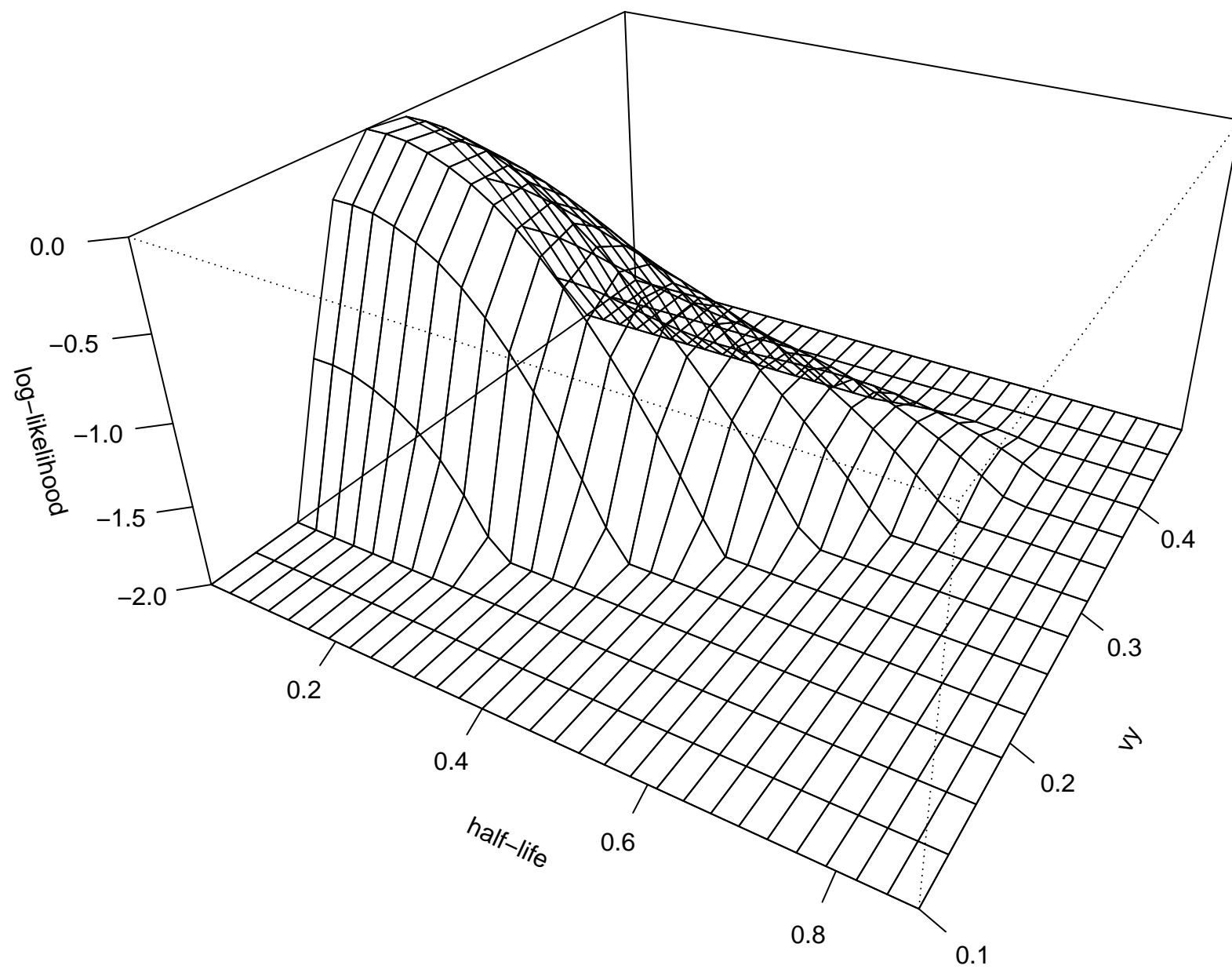

Supplement: Additional file 1: — All phylogenies used in analyses. R script for data extraction and analyses. Detailed results/raw output from SLOUCH. SLOUCH input data. Likelihood plots for all half-life estimations. (ZIP 2442 kb) [file 12862_2016_778_MOESM1_ESM.zip › Additional file 1/Results Allen's rule - forelimb length over body mass/Molossidae_LLBM_phySig.pdf]

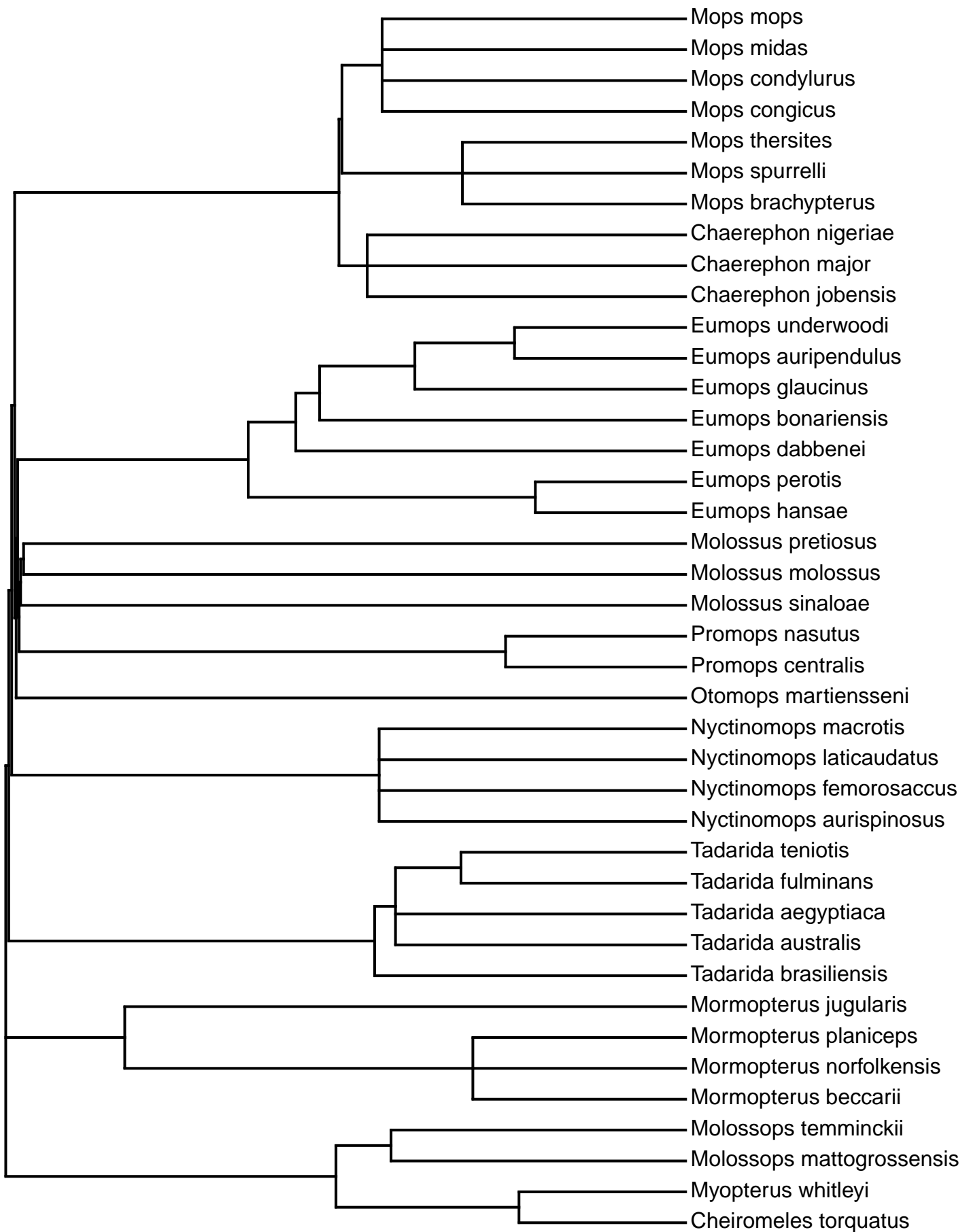

Supplement: Additional file 1: — All phylogenies used in analyses. R script for data extraction and analyses. Detailed results/raw output from SLOUCH. SLOUCH input data. Likelihood plots for all half-life estimations. (ZIP 2442 kb) [file 12862_2016_778_MOESM1_ESM.zip › Additional file 1/Results Allen's rule - forelimb length over body mass/Molossidae_tree.pdf]

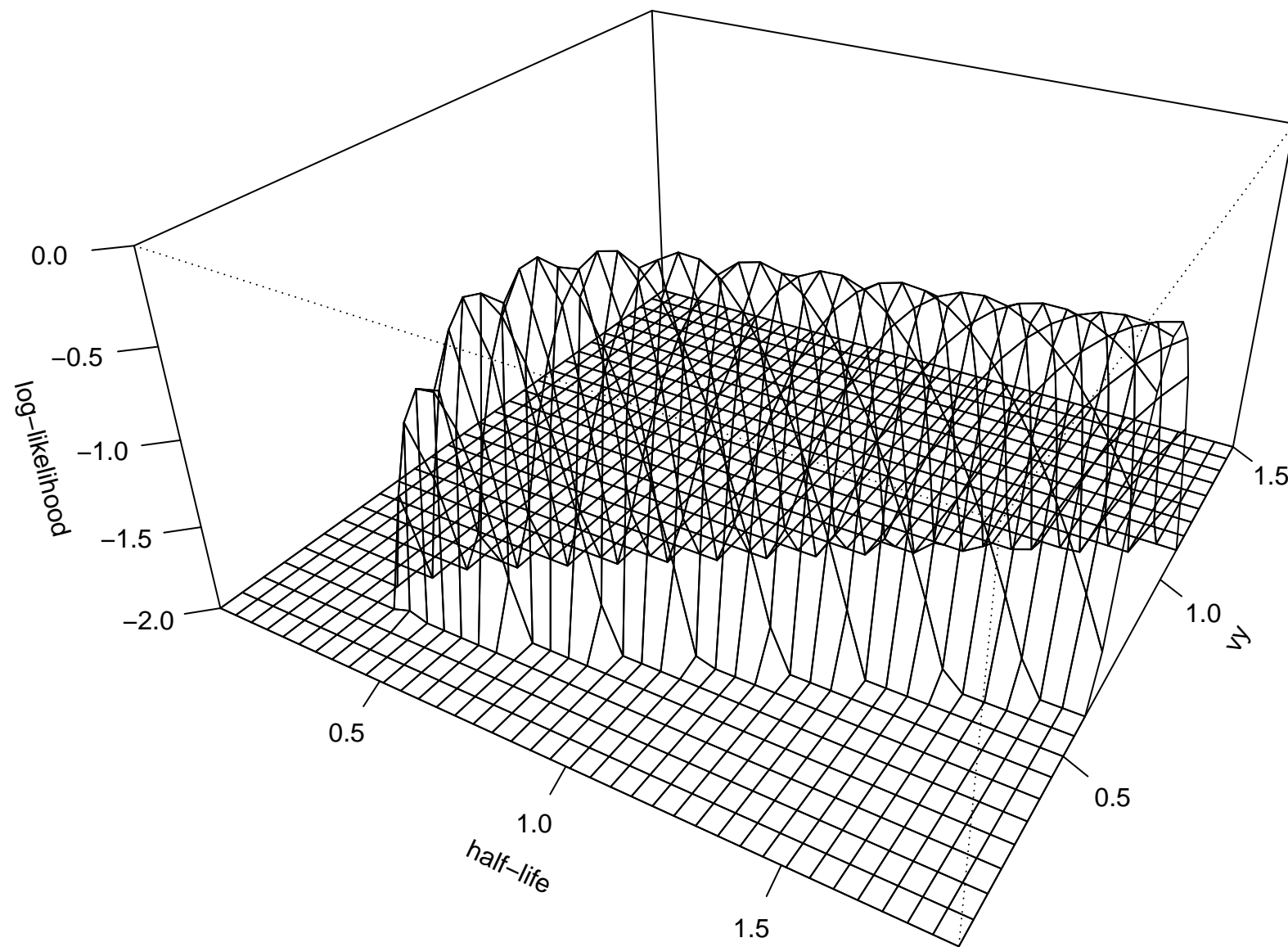

Supplement: Additional file 1: — All phylogenies used in analyses. R script for data extraction and analyses. Detailed results/raw output from SLOUCH. SLOUCH input data. Likelihood plots for all half-life estimations. (ZIP 2442 kb) [file 12862_2016_778_MOESM1_ESM.zip › Additional file 1/Results Allen's rule - forelimb length over body mass/Phyllostomidae_LLBM_maxlat.pdf]

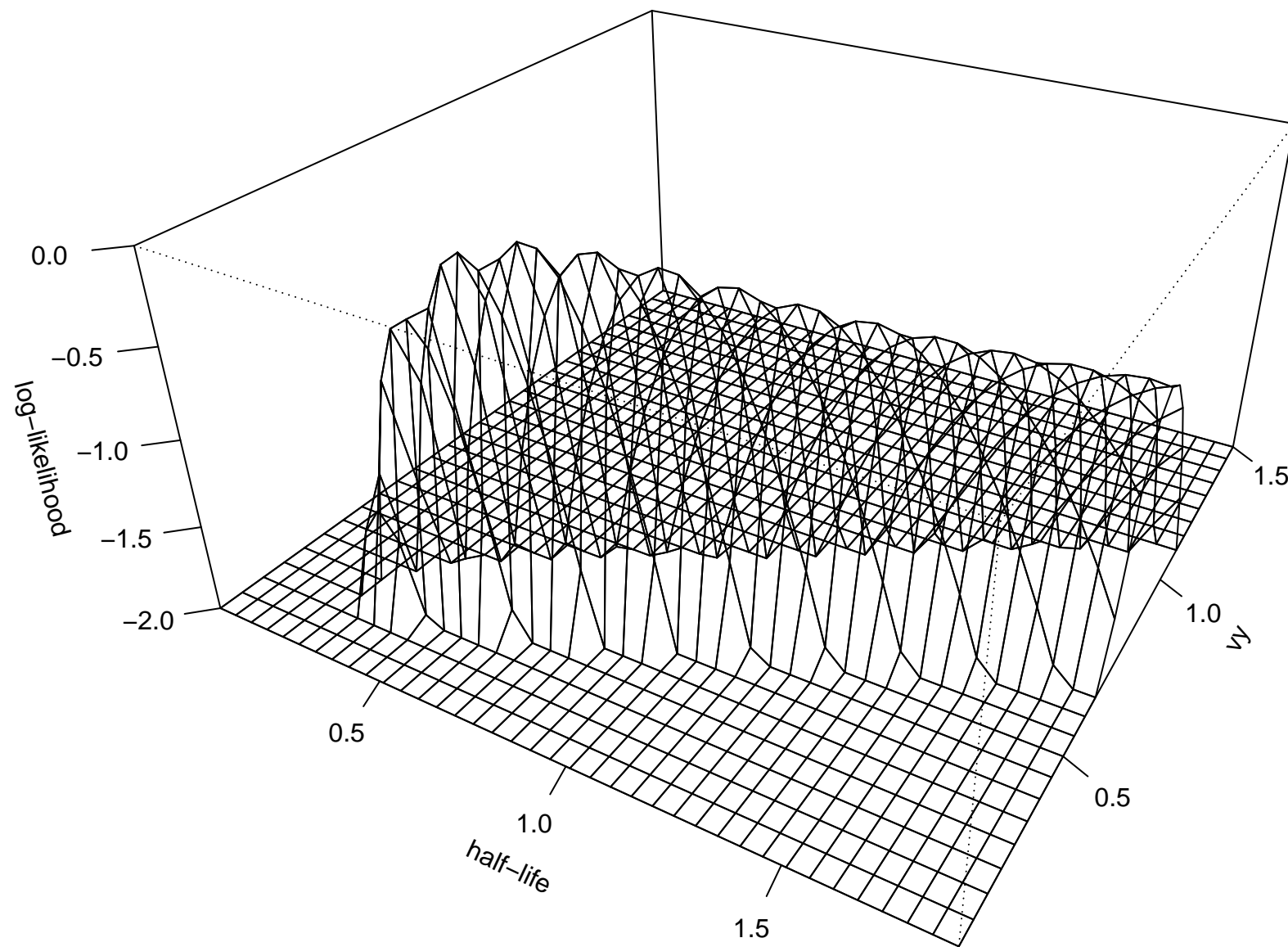

Supplement: Additional file 1: — All phylogenies used in analyses. R script for data extraction and analyses. Detailed results/raw output from SLOUCH. SLOUCH input data. Likelihood plots for all half-life estimations. (ZIP 2442 kb) [file 12862_2016_778_MOESM1_ESM.zip › Additional file 1/Results Allen's rule - forelimb length over body mass/Phyllostomidae_LLBM_midlat.pdf]

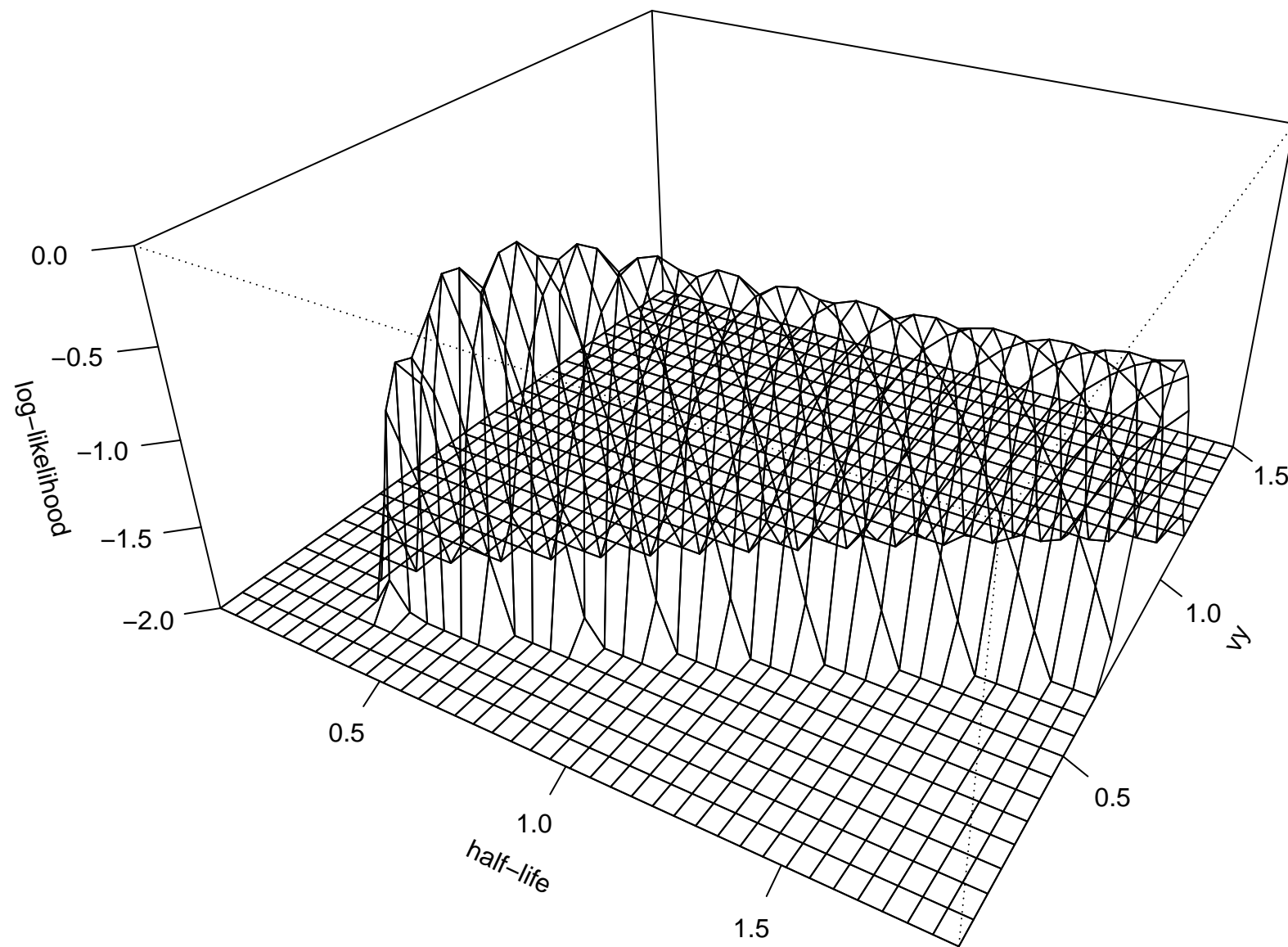

Supplement: Additional file 1: — All phylogenies used in analyses. R script for data extraction and analyses. Detailed results/raw output from SLOUCH. SLOUCH input data. Likelihood plots for all half-life estimations. (ZIP 2442 kb) [file 12862_2016_778_MOESM1_ESM.zip › Additional file 1/Results Allen's rule - forelimb length over body mass/Phyllostomidae_LLBM_temp.pdf]

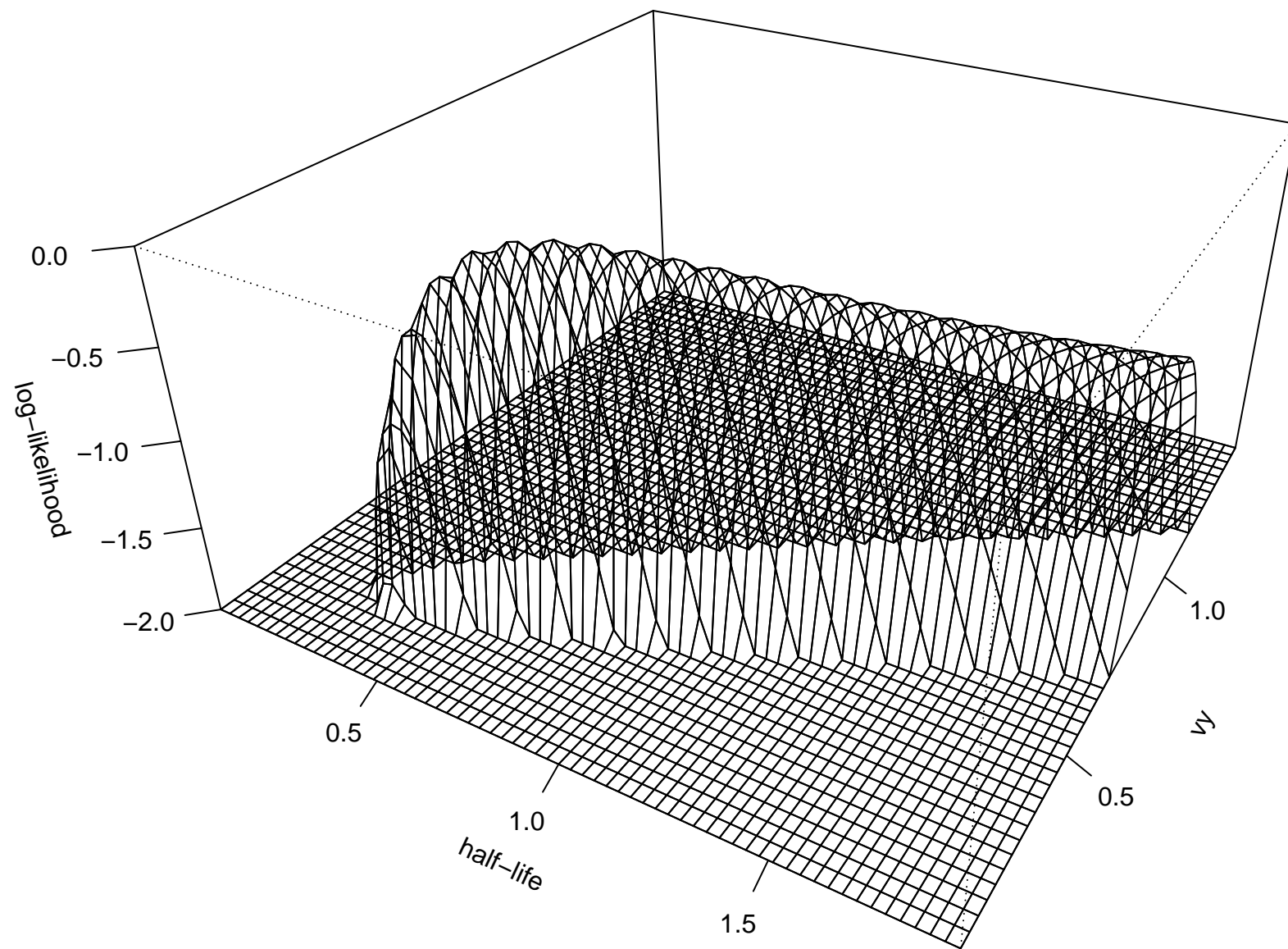

Supplement: Additional file 1: — All phylogenies used in analyses. R script for data extraction and analyses. Detailed results/raw output from SLOUCH. SLOUCH input data. Likelihood plots for all half-life estimations. (ZIP 2442 kb) [file 12862_2016_778_MOESM1_ESM.zip › Additional file 1/Results Allen's rule - forelimb length over body mass/Phyllostomidae_phySig.pdf]

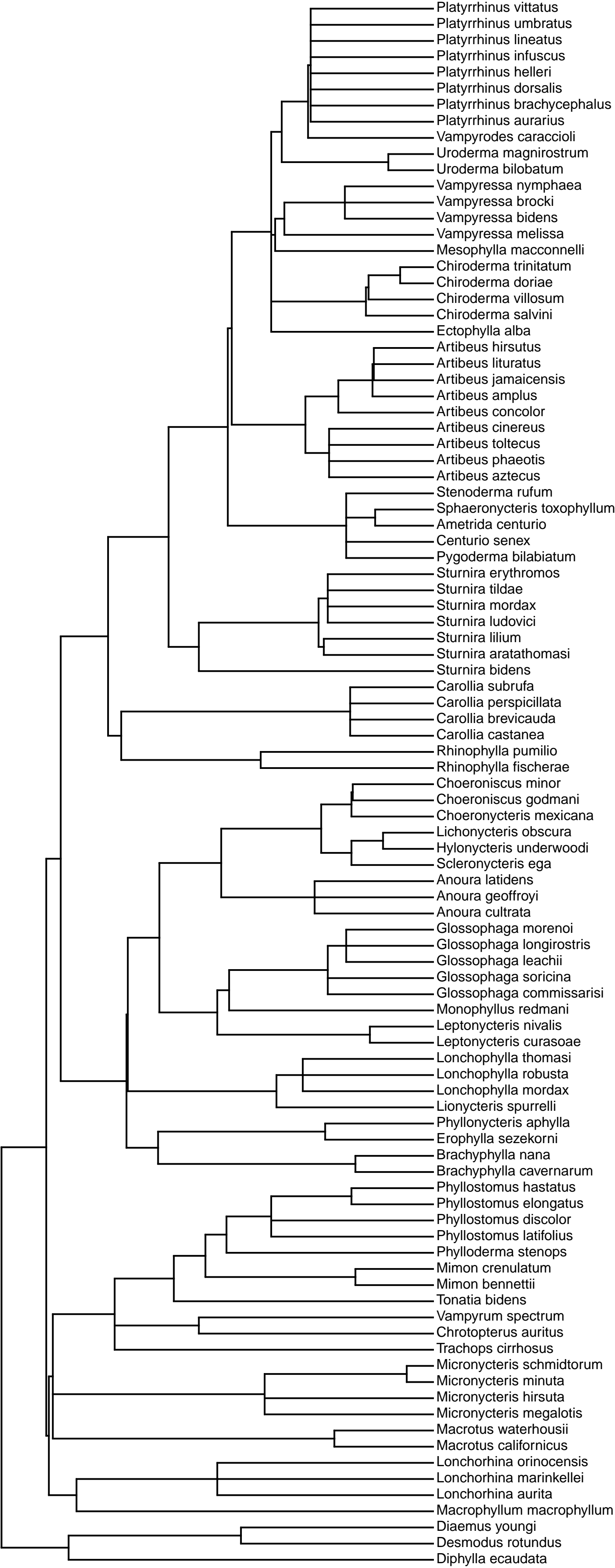

Supplement: Additional file 1: — All phylogenies used in analyses. R script for data extraction and analyses. Detailed results/raw output from SLOUCH. SLOUCH input data. Likelihood plots for all half-life estimations. (ZIP 2442 kb) [file 12862_2016_778_MOESM1_ESM.zip › Additional file 1/Results Allen's rule - forelimb length over body mass/Phyllostomidae_tree.pdf]

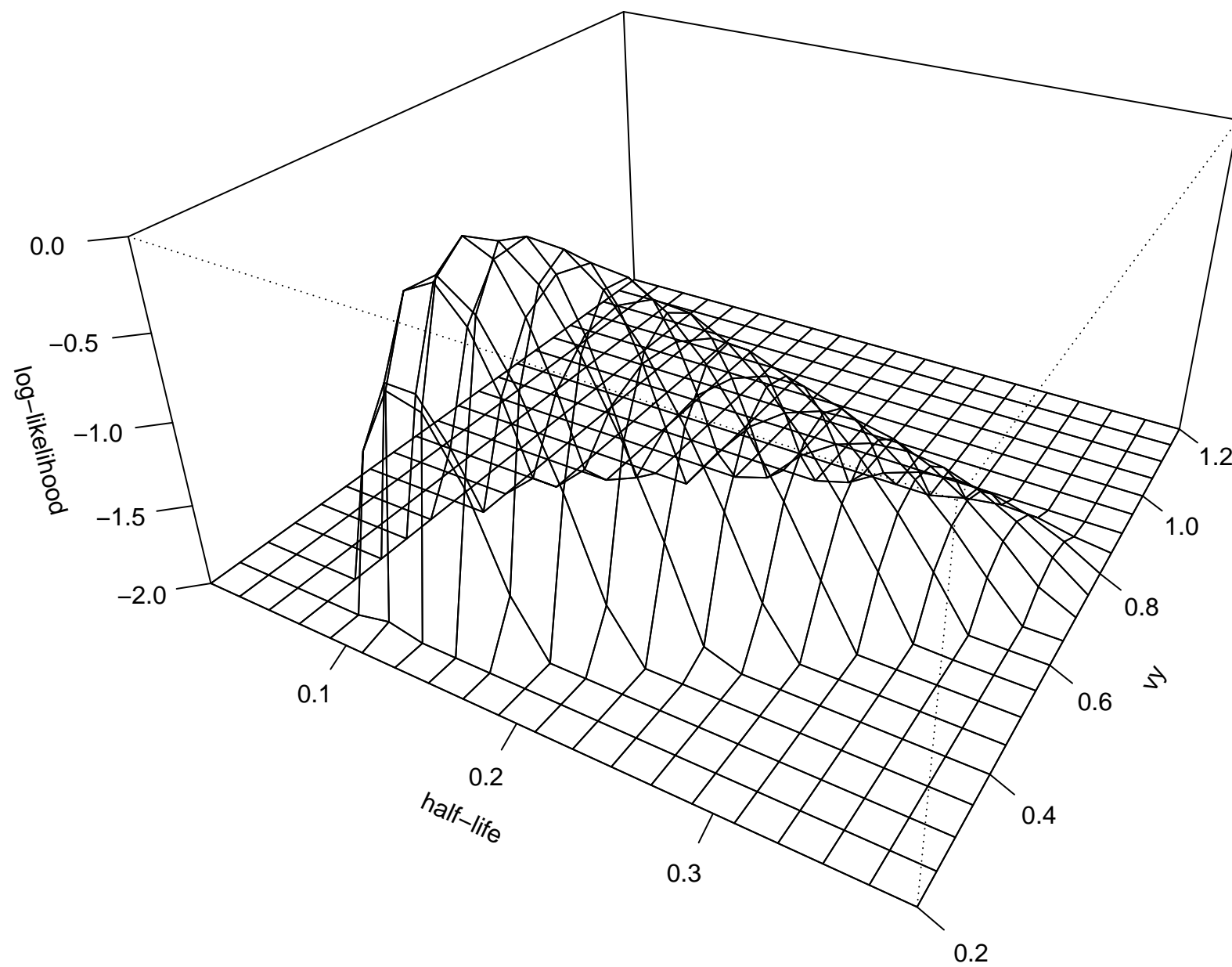

Supplement: Additional file 1: — All phylogenies used in analyses. R script for data extraction and analyses. Detailed results/raw output from SLOUCH. SLOUCH input data. Likelihood plots for all half-life estimations. (ZIP 2442 kb) [file 12862_2016_778_MOESM1_ESM.zip › Additional file 1/Results Allen's rule - forelimb length over body mass/Pteropodidae_LLBM_maxlat.pdf]

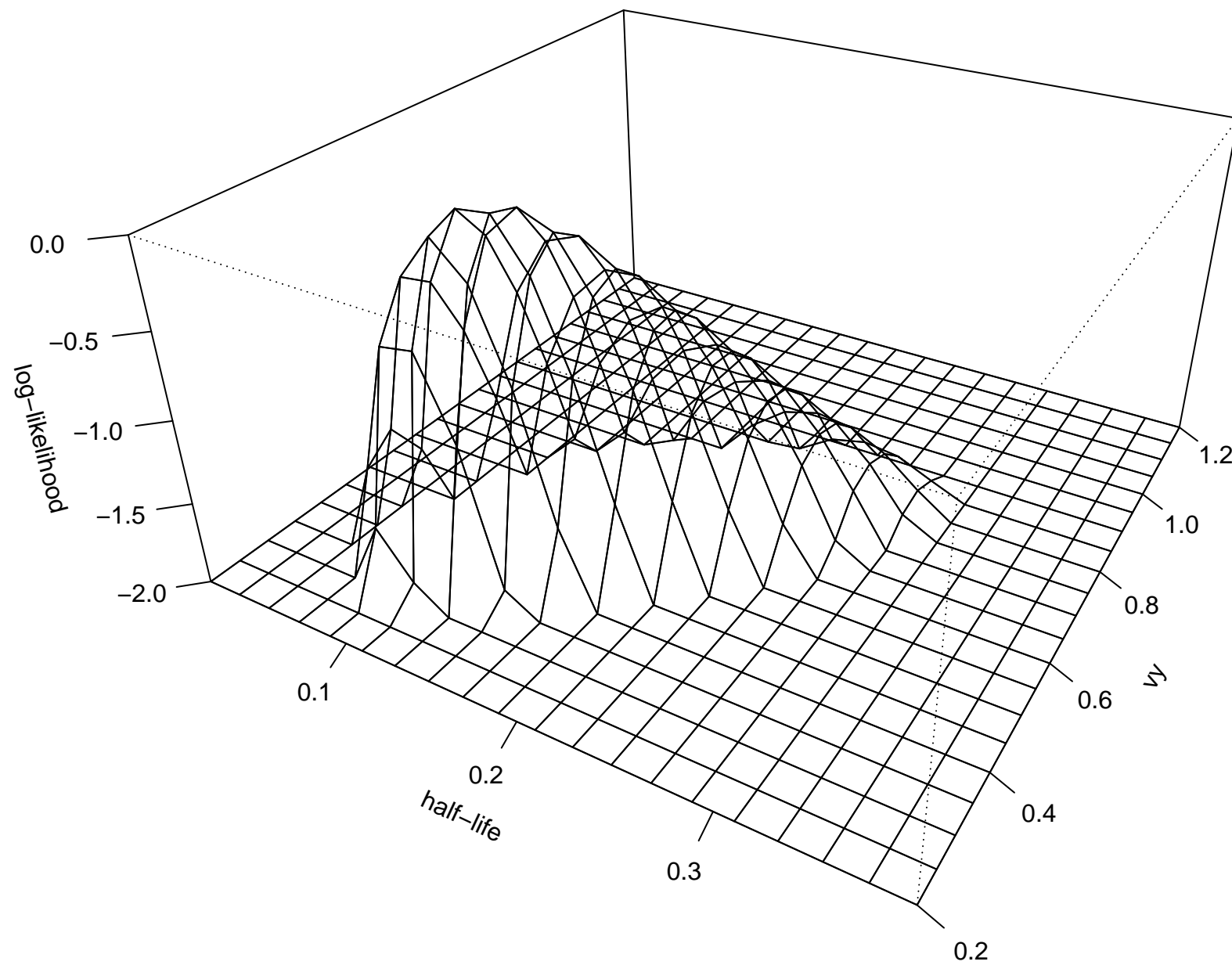

Supplement: Additional file 1: — All phylogenies used in analyses. R script for data extraction and analyses. Detailed results/raw output from SLOUCH. SLOUCH input data. Likelihood plots for all half-life estimations. (ZIP 2442 kb) [file 12862_2016_778_MOESM1_ESM.zip › Additional file 1/Results Allen's rule - forelimb length over body mass/Pteropodidae_LLBM_midlat.pdf]

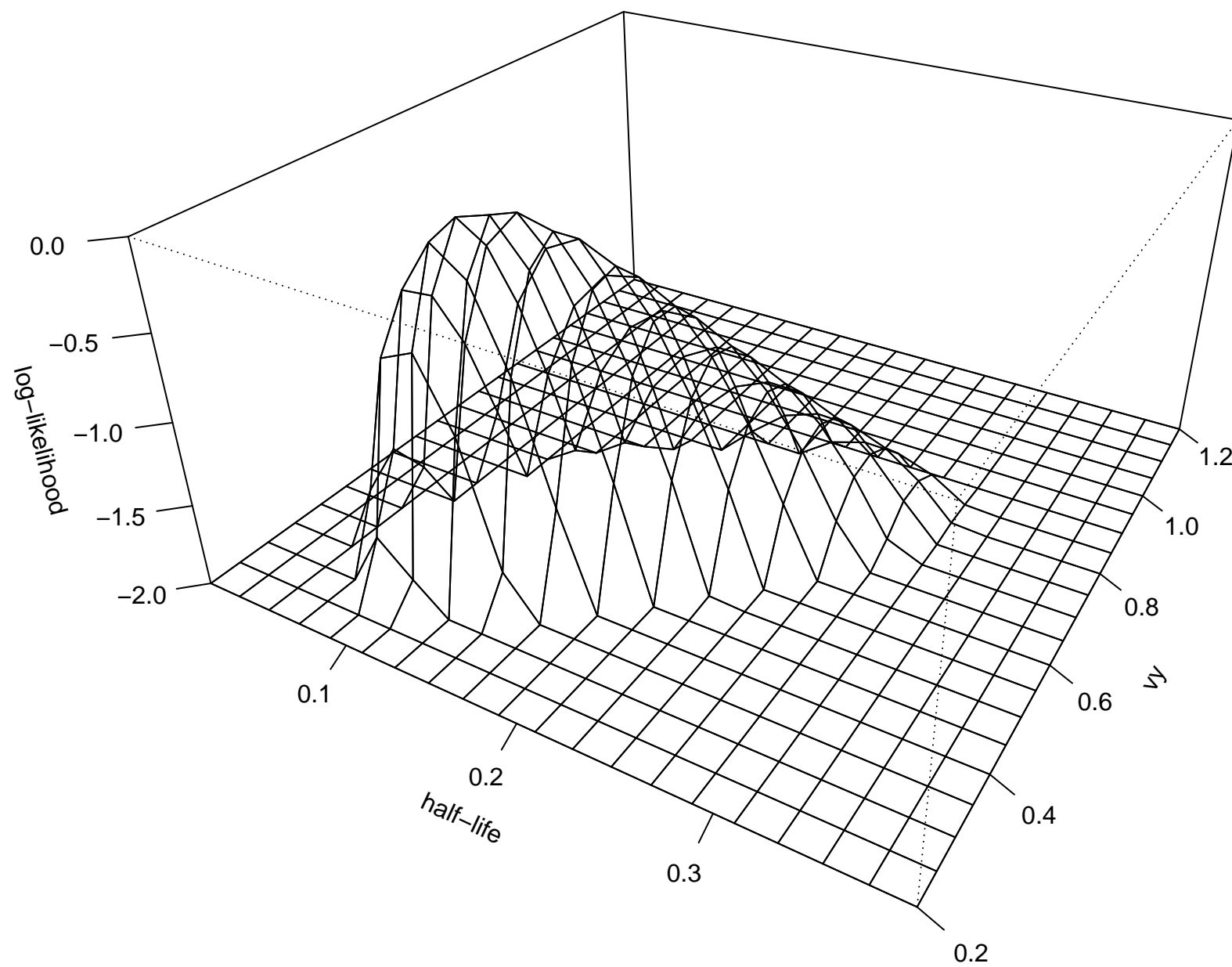

Supplement: Additional file 1: — All phylogenies used in analyses. R script for data extraction and analyses. Detailed results/raw output from SLOUCH. SLOUCH input data. Likelihood plots for all half-life estimations. (ZIP 2442 kb) [file 12862_2016_778_MOESM1_ESM.zip › Additional file 1/Results Allen's rule - forelimb length over body mass/Pteropodidae_LLBM_temp.pdf]

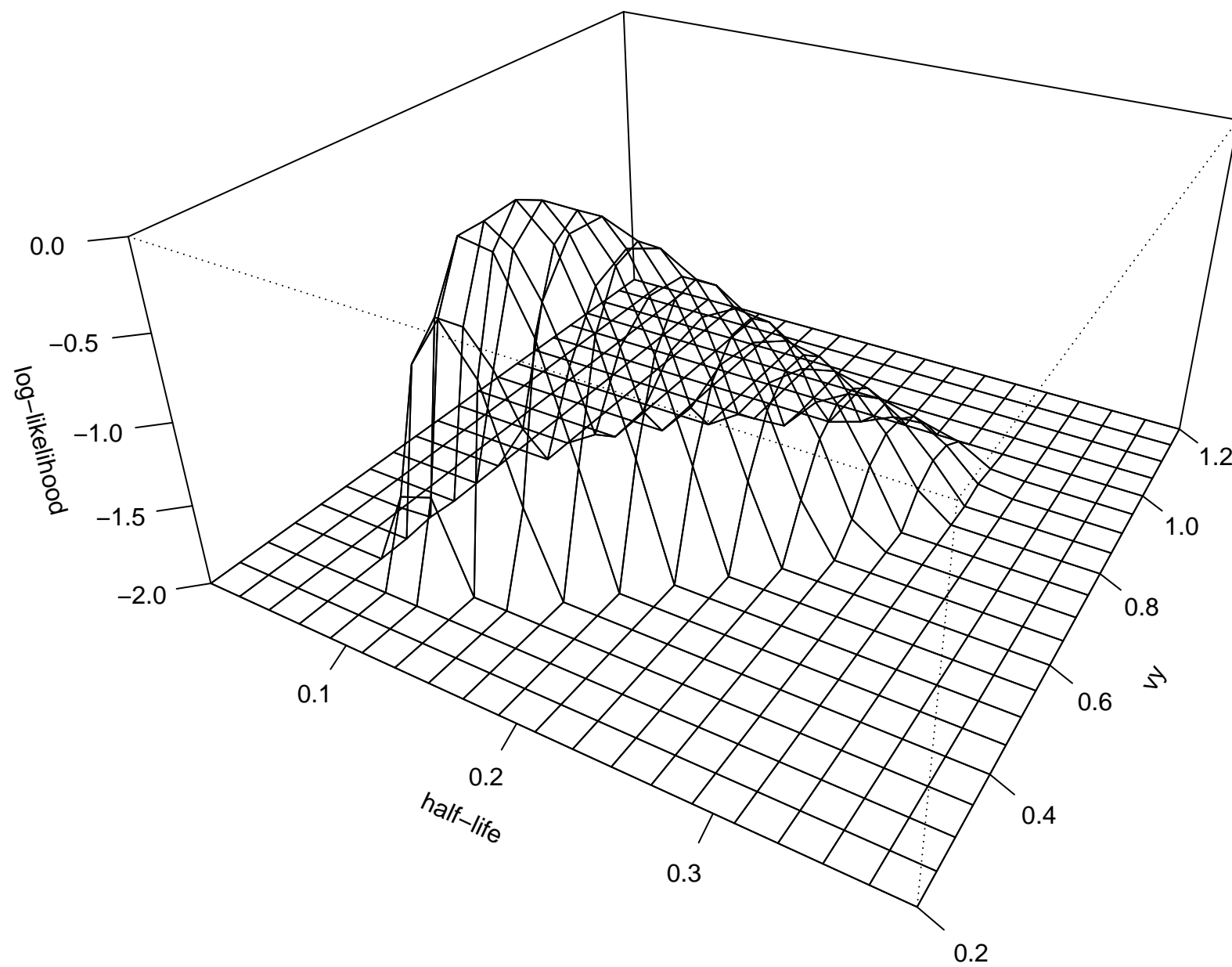

Supplement: Additional file 1: — All phylogenies used in analyses. R script for data extraction and analyses. Detailed results/raw output from SLOUCH. SLOUCH input data. Likelihood plots for all half-life estimations. (ZIP 2442 kb) [file 12862_2016_778_MOESM1_ESM.zip › Additional file 1/Results Allen's rule - forelimb length over body mass/Pteropodidae_phySig.pdf]

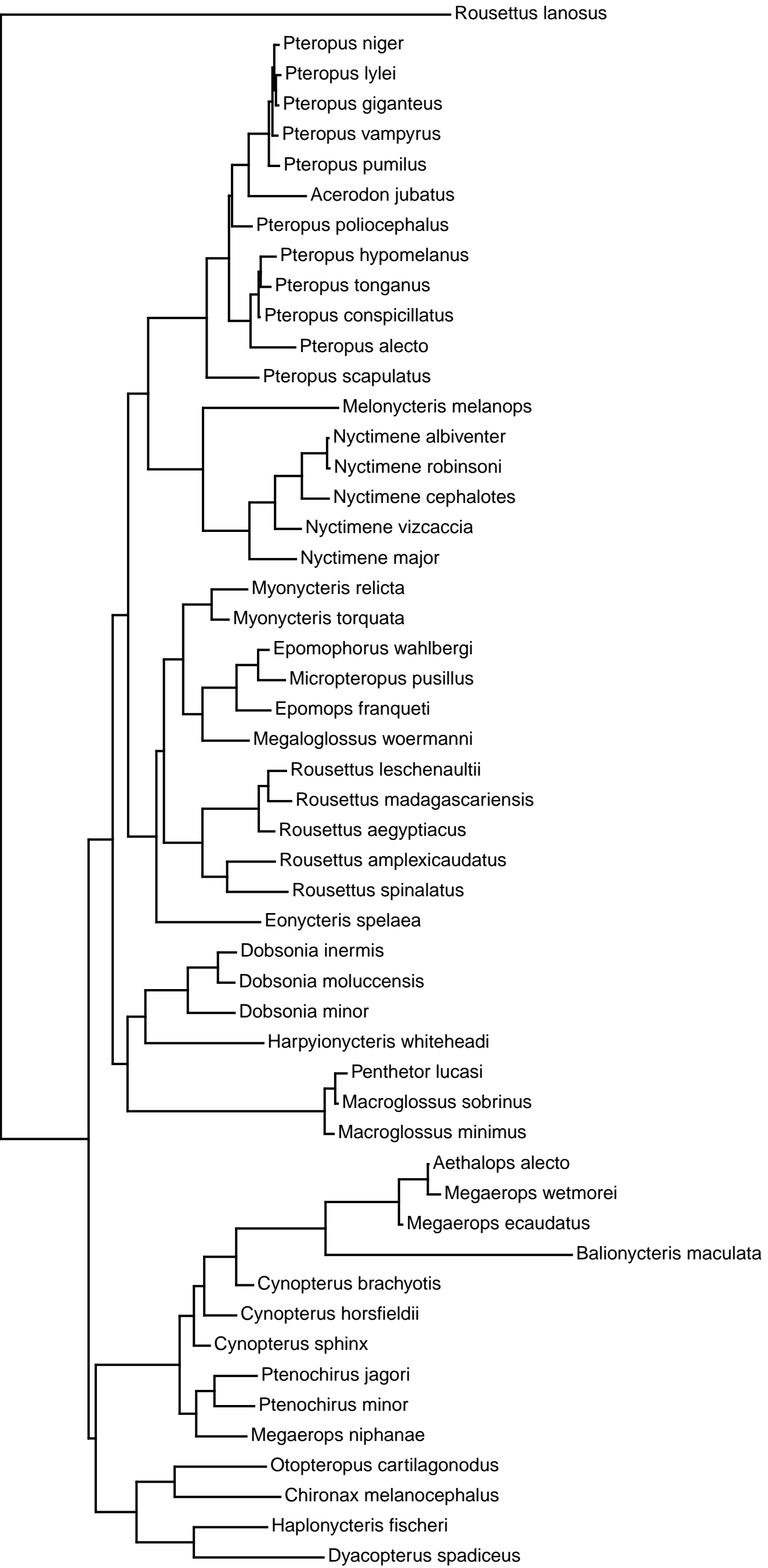

Supplement: Additional file 1: — All phylogenies used in analyses. R script for data extraction and analyses. Detailed results/raw output from SLOUCH. SLOUCH input data. Likelihood plots for all half-life estimations. (ZIP 2442 kb) [file 12862_2016_778_MOESM1_ESM.zip › Additional file 1/Results Allen's rule - forelimb length over body mass/Pteropodidae_tree.pdf]

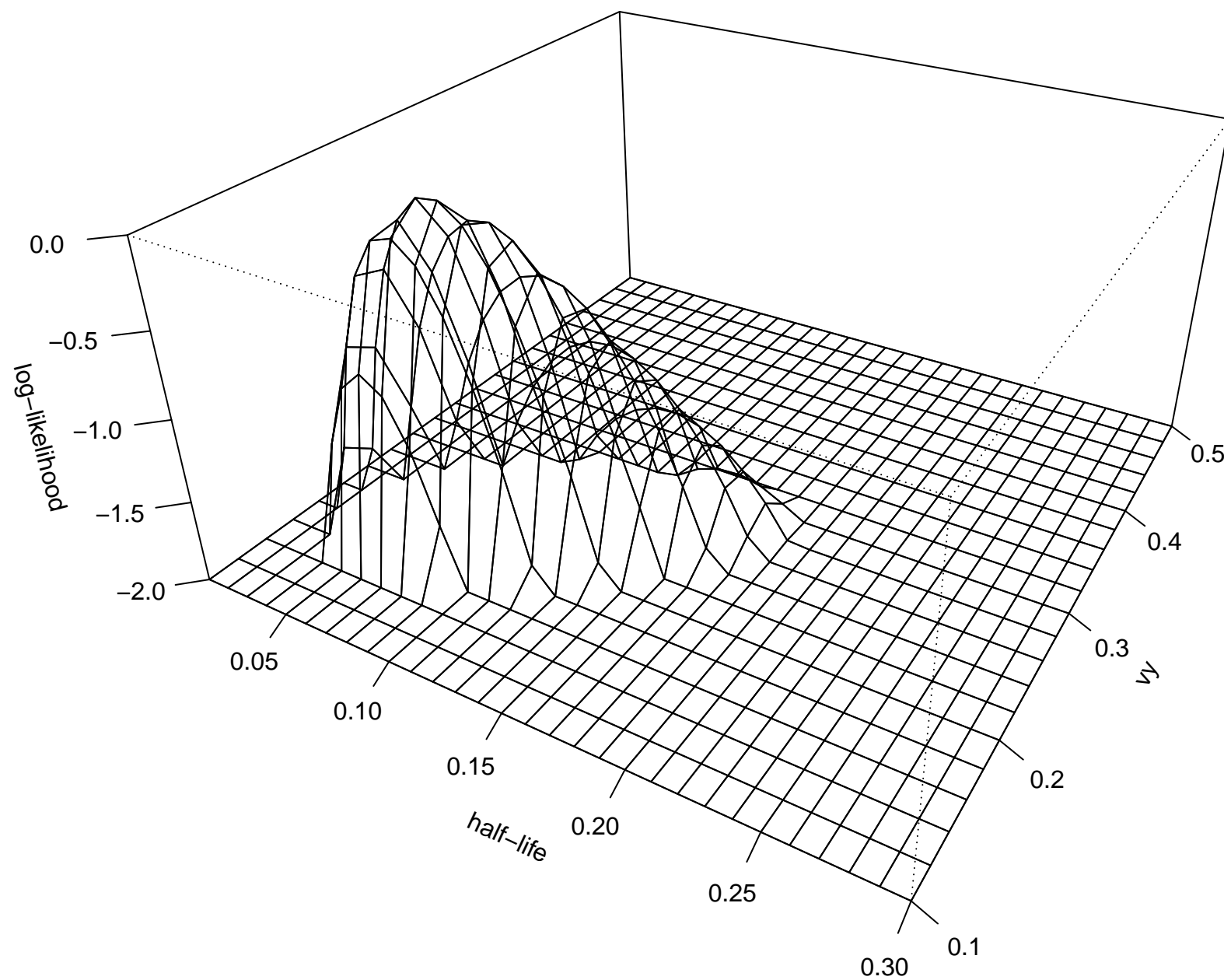

Supplement: Additional file 1: — All phylogenies used in analyses. R script for data extraction and analyses. Detailed results/raw output from SLOUCH. SLOUCH input data. Likelihood plots for all half-life estimations. (ZIP 2442 kb) [file 12862_2016_778_MOESM1_ESM.zip › Additional file 1/Results Allen's rule - forelimb length over body mass/Vespertilionidae_LLBM_maxlat.pdf]

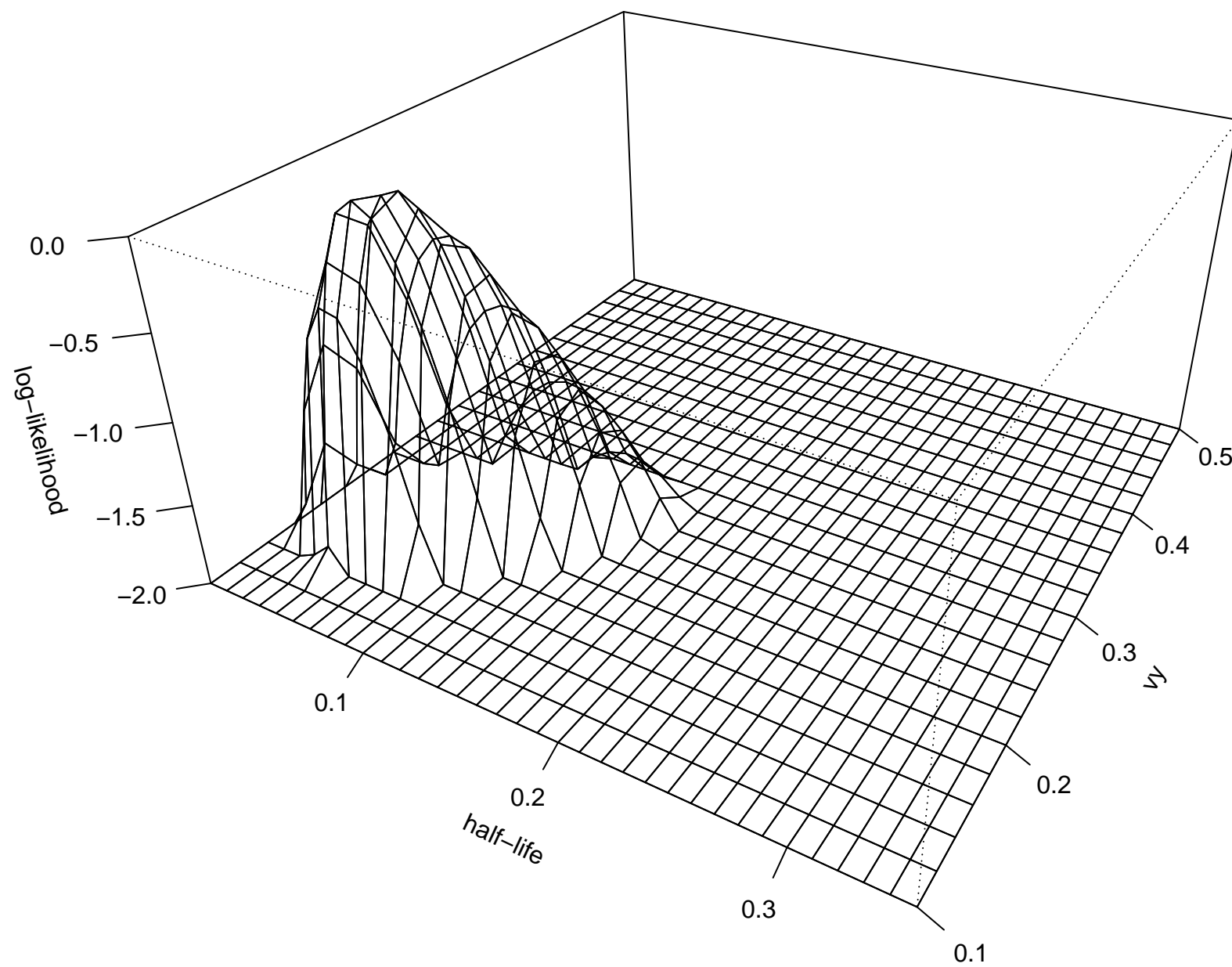

Supplement: Additional file 1: — All phylogenies used in analyses. R script for data extraction and analyses. Detailed results/raw output from SLOUCH. SLOUCH input data. Likelihood plots for all half-life estimations. (ZIP 2442 kb) [file 12862_2016_778_MOESM1_ESM.zip › Additional file 1/Results Allen's rule - forelimb length over body mass/Vespertilionidae_LLBM_midlat.pdf]

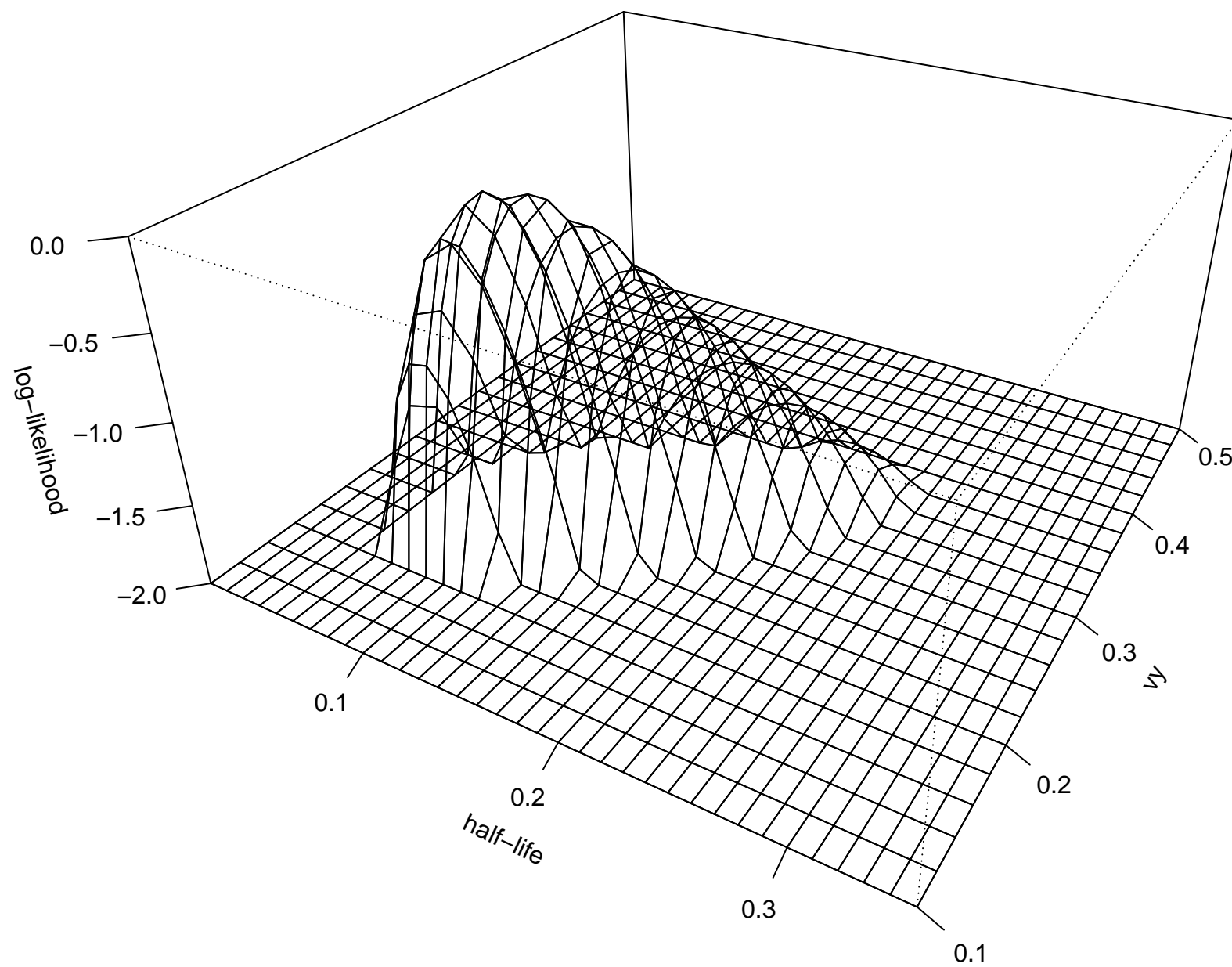

Supplement: Additional file 1: — All phylogenies used in analyses. R script for data extraction and analyses. Detailed results/raw output from SLOUCH. SLOUCH input data. Likelihood plots for all half-life estimations. (ZIP 2442 kb) [file 12862_2016_778_MOESM1_ESM.zip › Additional file 1/Results Allen's rule - forelimb length over body mass/Vespertilionidae_LLBM_phySig.pdf]

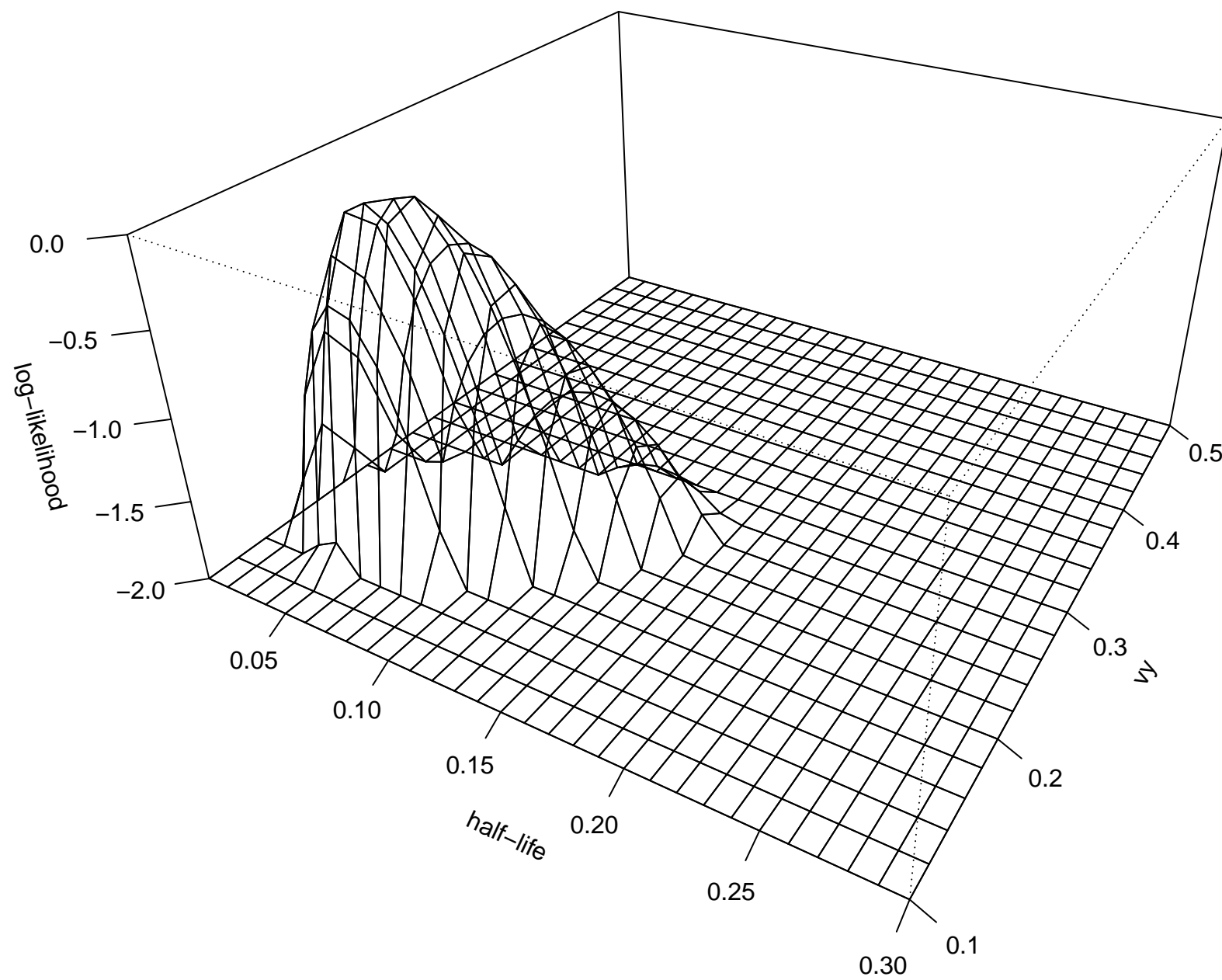

Supplement: Additional file 1: — All phylogenies used in analyses. R script for data extraction and analyses. Detailed results/raw output from SLOUCH. SLOUCH input data. Likelihood plots for all half-life estimations. (ZIP 2442 kb) [file 12862_2016_778_MOESM1_ESM.zip › Additional file 1/Results Allen's rule - forelimb length over body mass/Vespertilionidae_LLBM_temp.pdf]

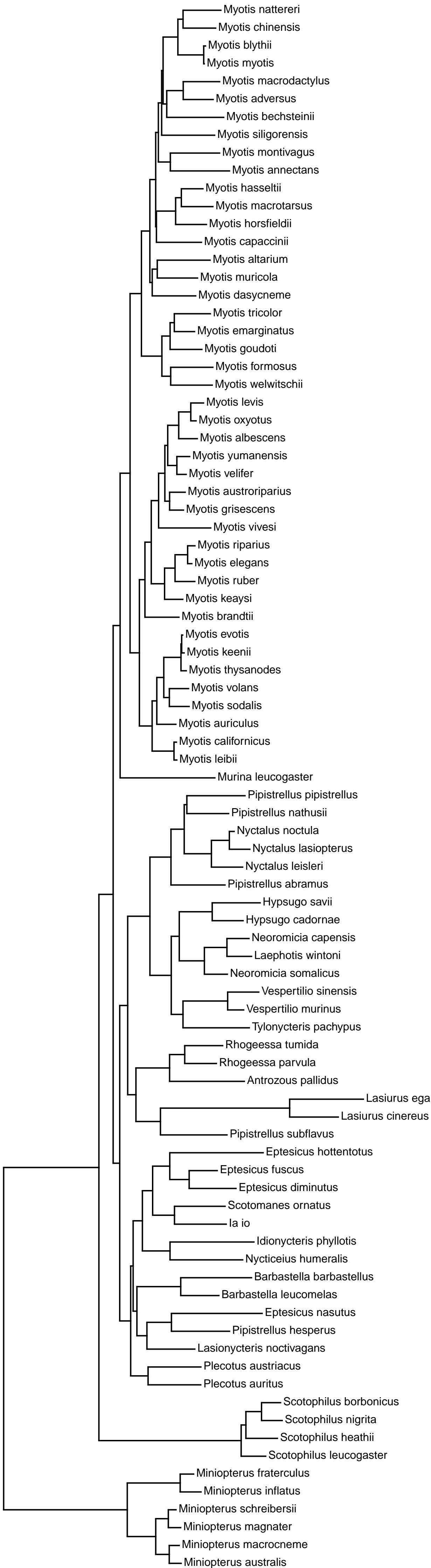

Supplement: Additional file 1: — All phylogenies used in analyses. R script for data extraction and analyses. Detailed results/raw output from SLOUCH. SLOUCH input data. Likelihood plots for all half-life estimations. (ZIP 2442 kb) [file 12862_2016_778_MOESM1_ESM.zip › Additional file 1/Results Allen's rule - forelimb length over body mass/Vespertilionidae_tree.pdf]

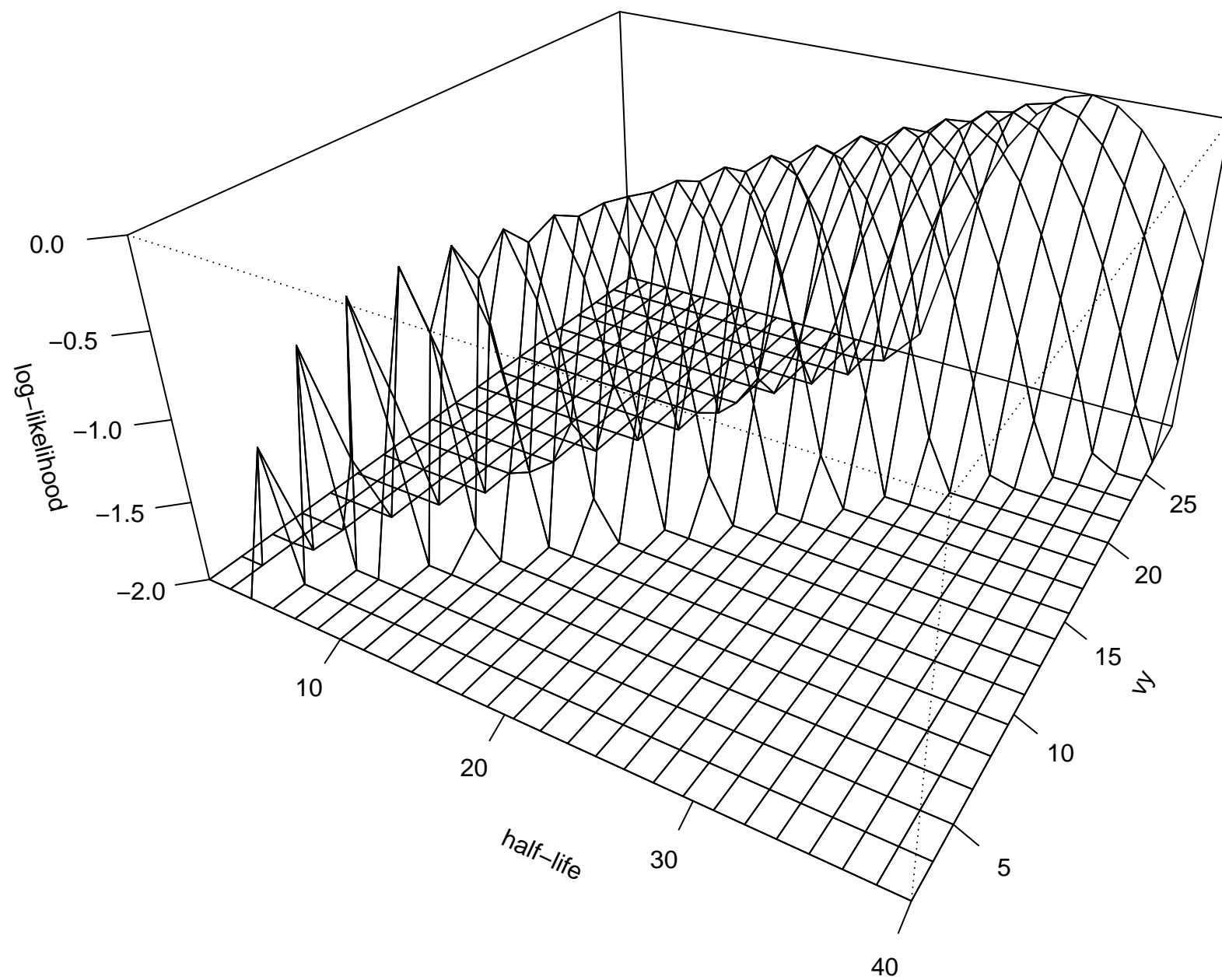

Supplement: Additional file 1: — All phylogenies used in analyses. R script for data extraction and analyses. Detailed results/raw output from SLOUCH. SLOUCH input data. Likelihood plots for all half-life estimations. (ZIP 2442 kb) [file 12862_2016_778_MOESM1_ESM.zip › Additional file 1/Results Bergman's rule - body mass/Bovidae_BM_maxlat.pdf]

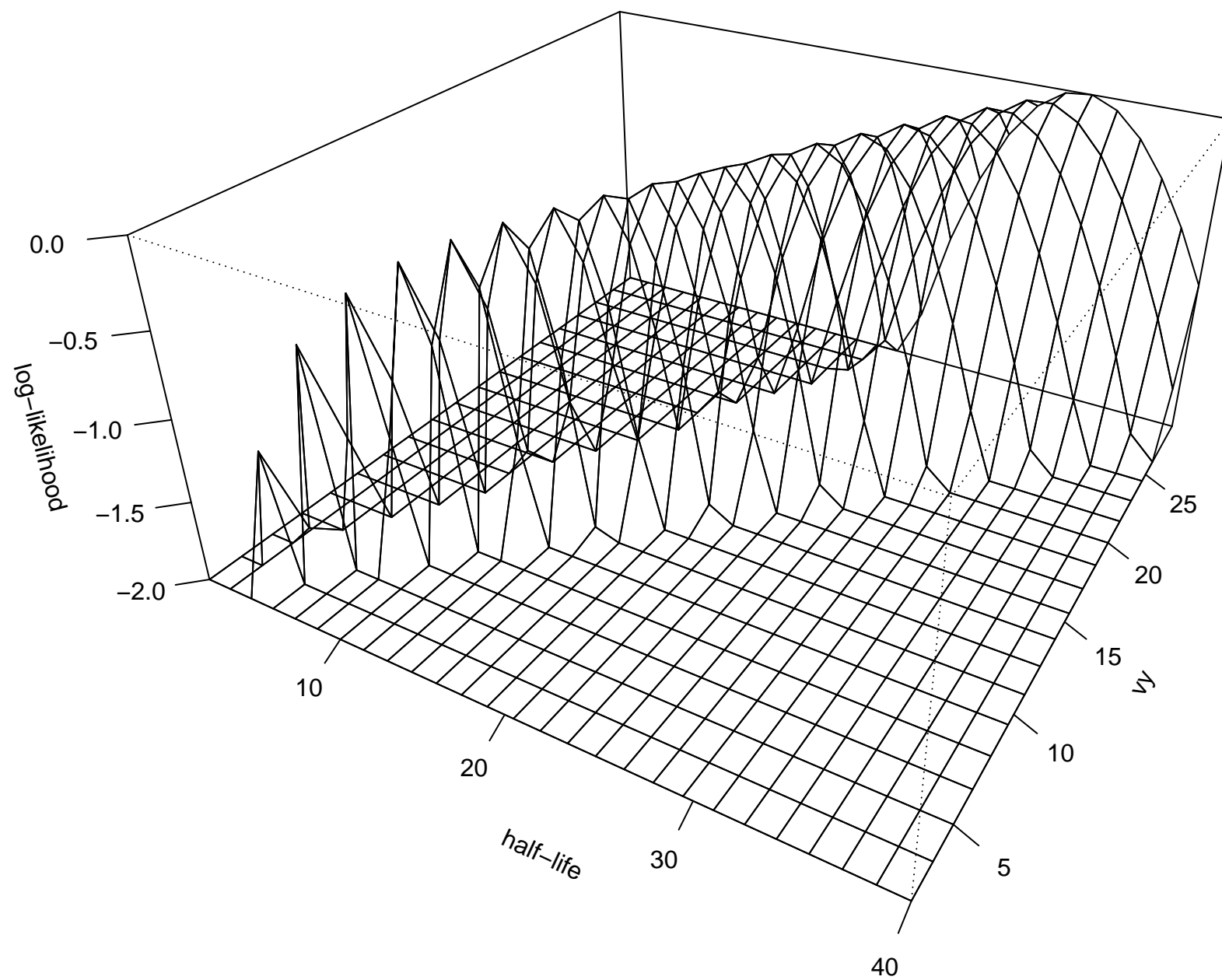

Supplement: Additional file 1: — All phylogenies used in analyses. R script for data extraction and analyses. Detailed results/raw output from SLOUCH. SLOUCH input data. Likelihood plots for all half-life estimations. (ZIP 2442 kb) [file 12862_2016_778_MOESM1_ESM.zip › Additional file 1/Results Bergman's rule - body mass/Bovidae_BM_midlat.pdf]

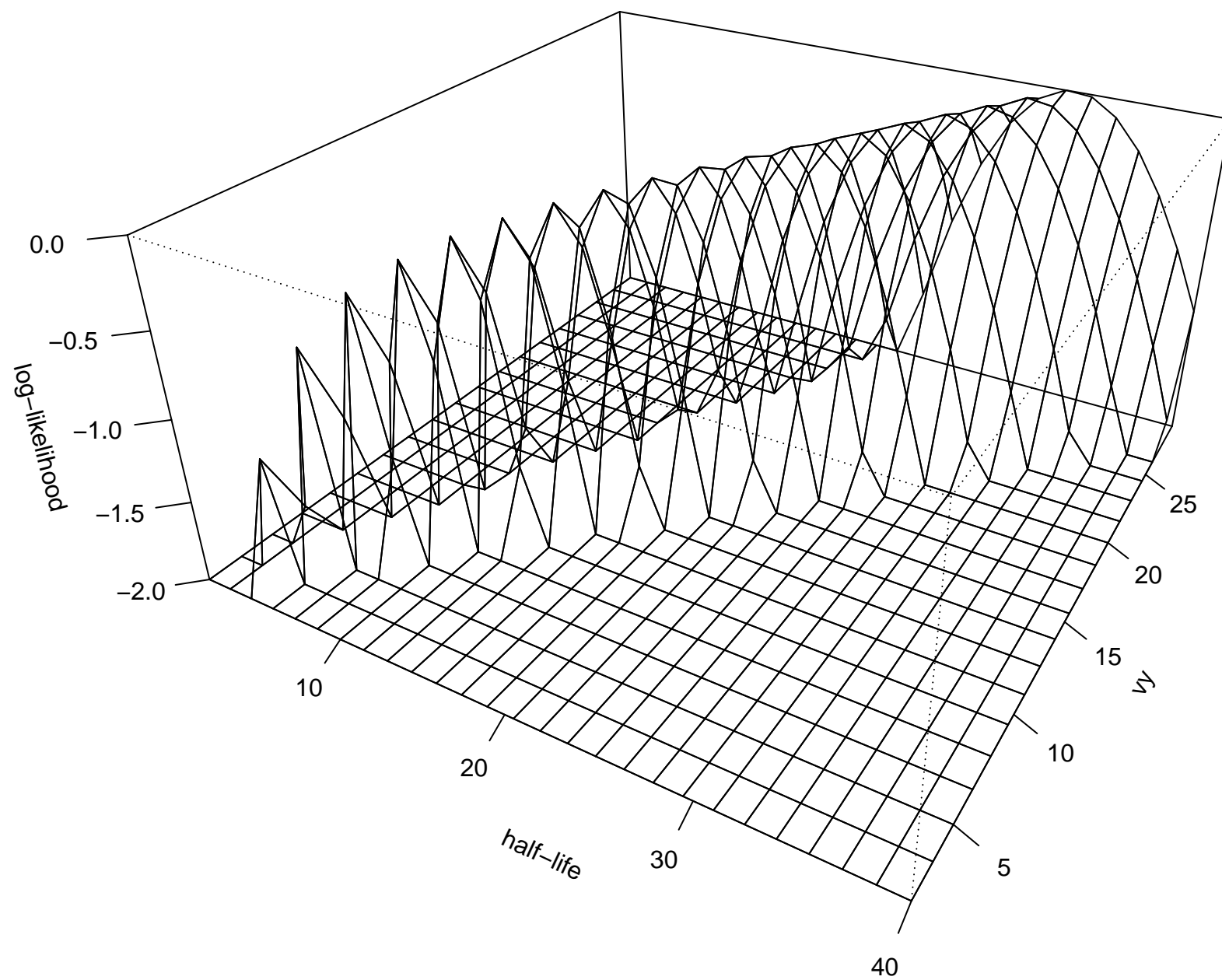

Supplement: Additional file 1: — All phylogenies used in analyses. R script for data extraction and analyses. Detailed results/raw output from SLOUCH. SLOUCH input data. Likelihood plots for all half-life estimations. (ZIP 2442 kb) [file 12862_2016_778_MOESM1_ESM.zip › Additional file 1/Results Bergman's rule - body mass/Bovidae_BM_temp.pdf]

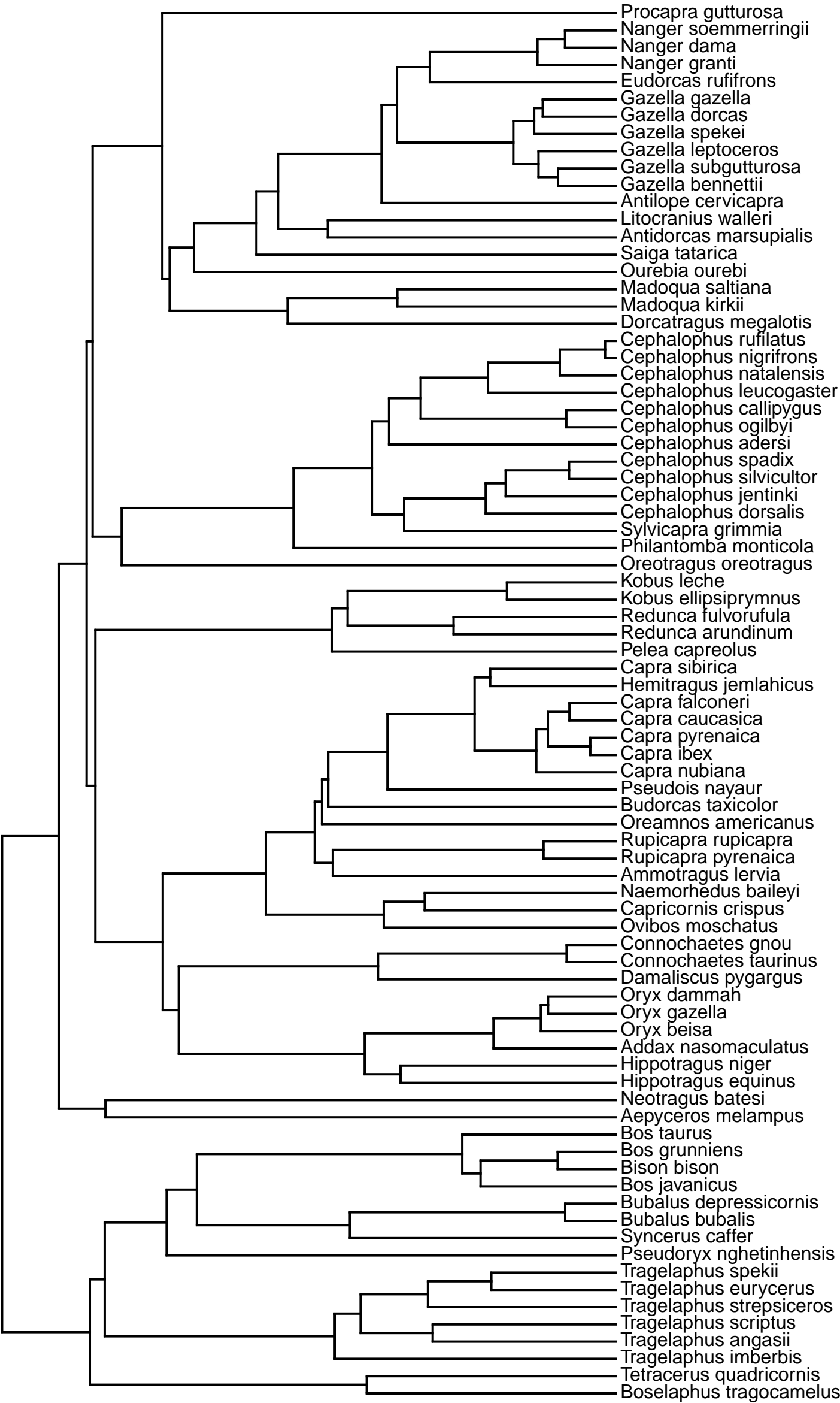

Supplement: Additional file 1: — All phylogenies used in analyses. R script for data extraction and analyses. Detailed results/raw output from SLOUCH. SLOUCH input data. Likelihood plots for all half-life estimations. (ZIP 2442 kb) [file 12862_2016_778_MOESM1_ESM.zip › Additional file 1/Results Bergman's rule - body mass/Bovidae_tree.pdf]

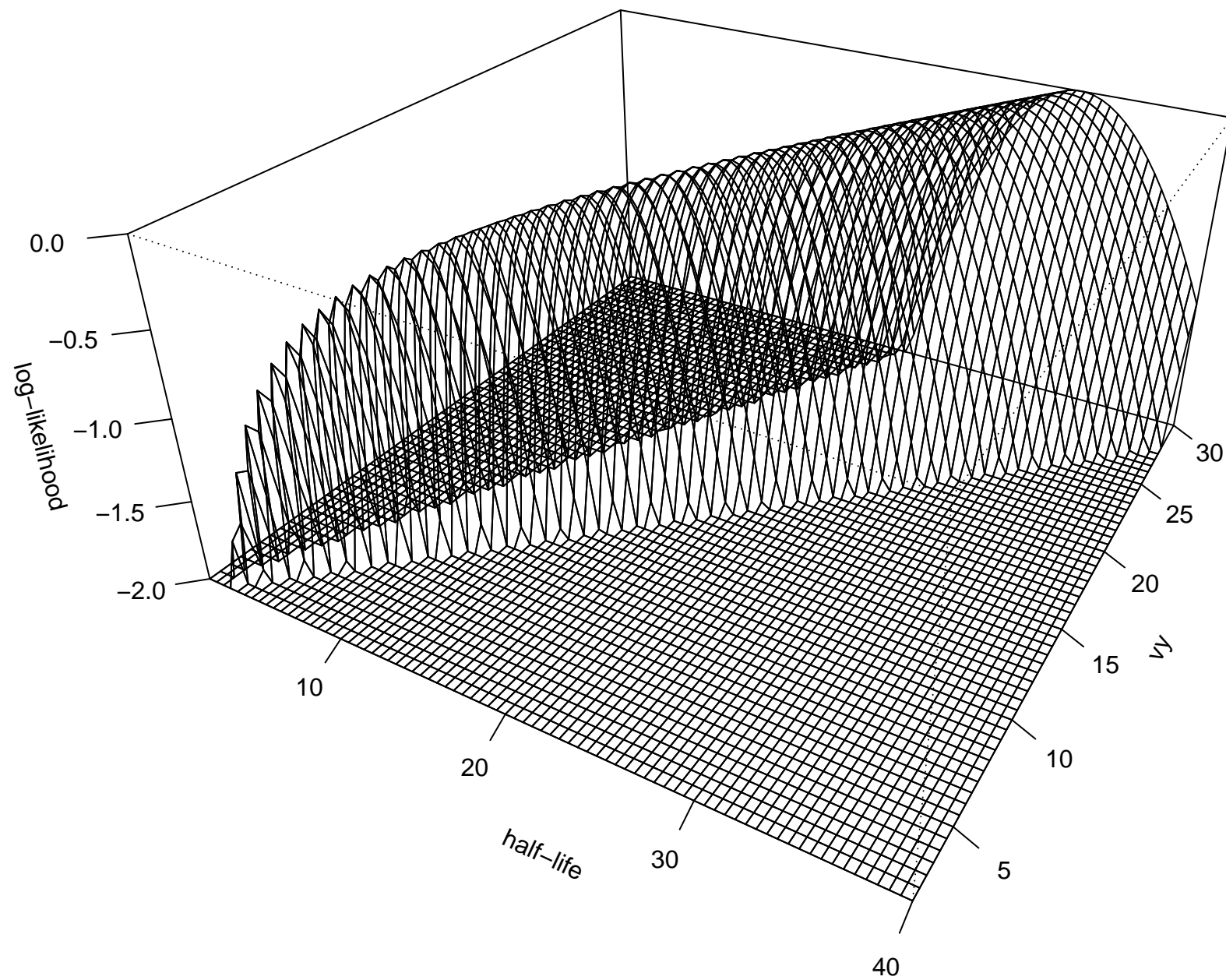

Supplement: Additional file 1: — All phylogenies used in analyses. R script for data extraction and analyses. Detailed results/raw output from SLOUCH. SLOUCH input data. Likelihood plots for all half-life estimations. (ZIP 2442 kb) [file 12862_2016_778_MOESM1_ESM.zip › Additional file 1/Results Bergman's rule - body mass/bovida_PhySig_BM.pdf]

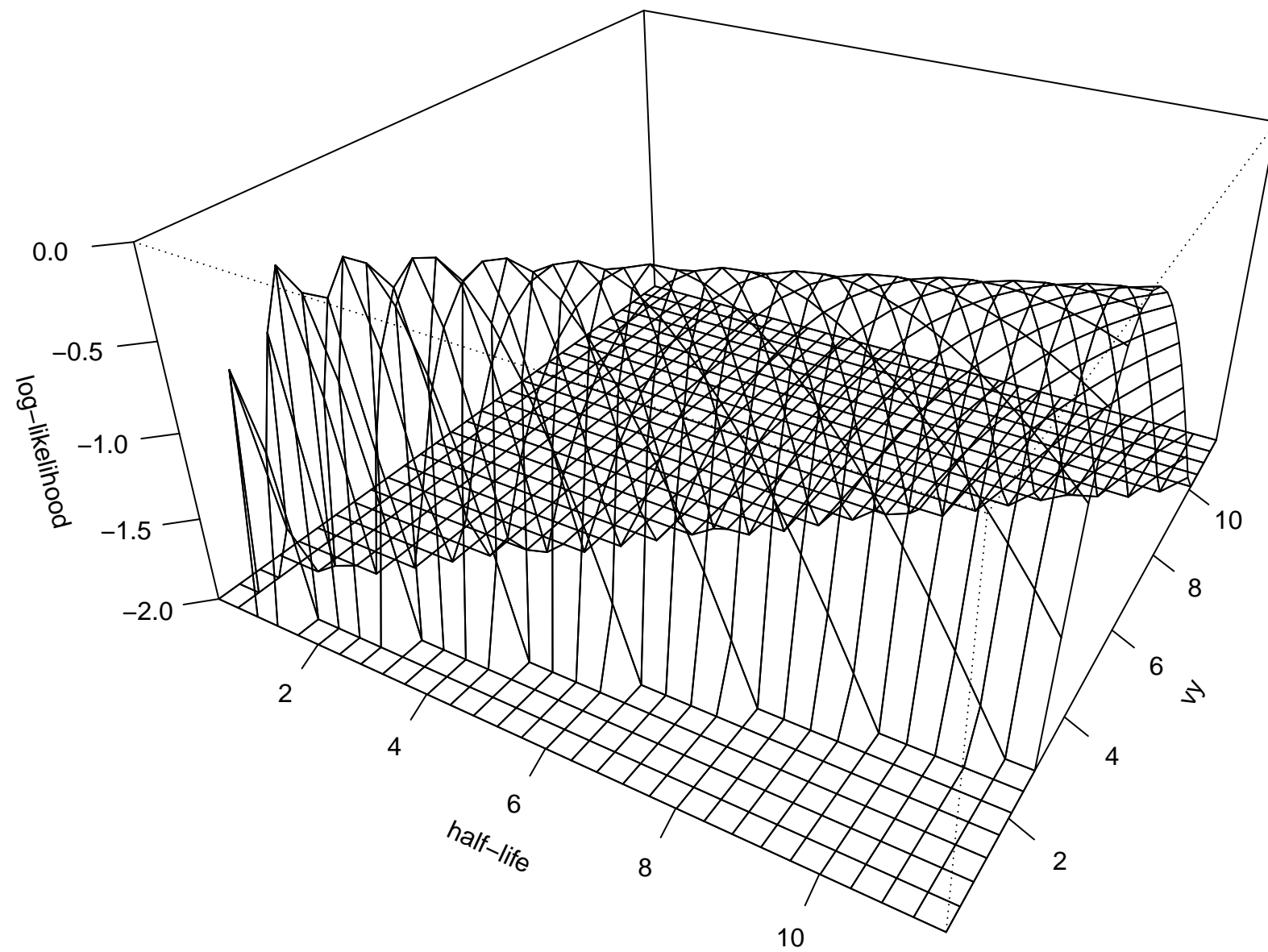

Supplement: Additional file 1: — All phylogenies used in analyses. R script for data extraction and analyses. Detailed results/raw output from SLOUCH. SLOUCH input data. Likelihood plots for all half-life estimations. (ZIP 2442 kb) [file 12862_2016_778_MOESM1_ESM.zip › Additional file 1/Results Bergman's rule - body mass/Canidae_BM_maxlat.pdf]

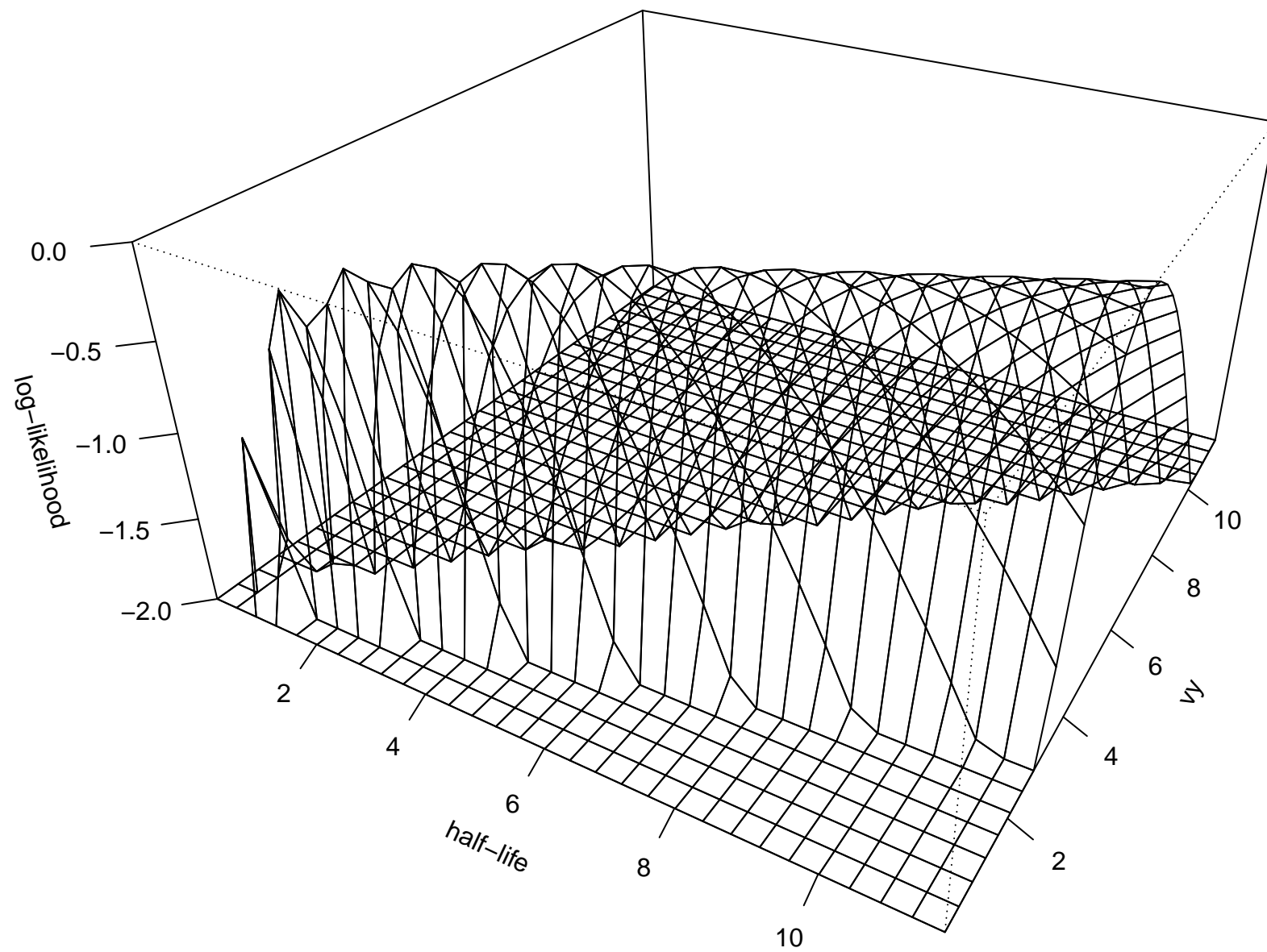

Supplement: Additional file 1: — All phylogenies used in analyses. R script for data extraction and analyses. Detailed results/raw output from SLOUCH. SLOUCH input data. Likelihood plots for all half-life estimations. (ZIP 2442 kb) [file 12862_2016_778_MOESM1_ESM.zip › Additional file 1/Results Bergman's rule - body mass/Canidae_BM_midlat.pdf]

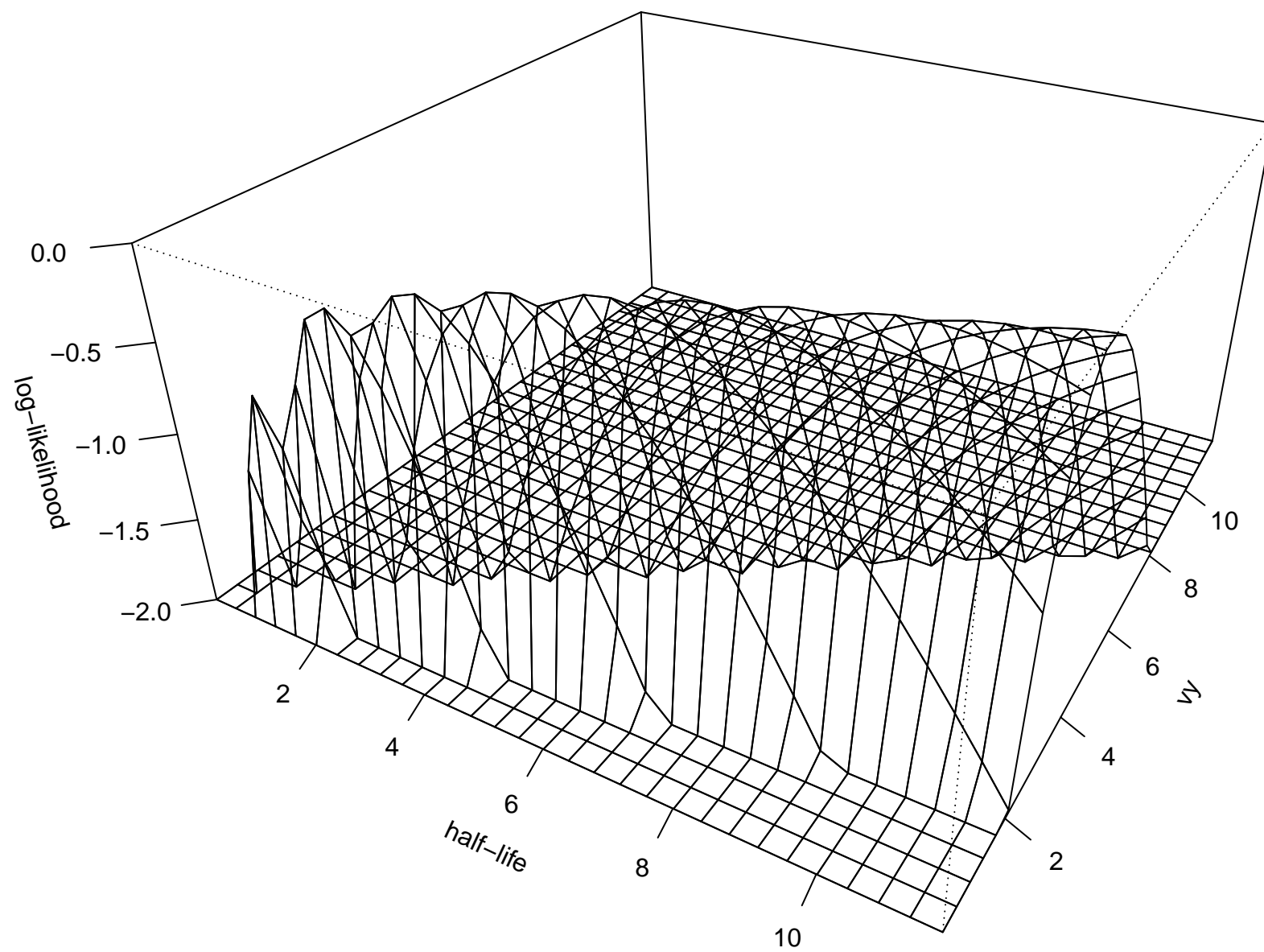

Supplement: Additional file 1: — All phylogenies used in analyses. R script for data extraction and analyses. Detailed results/raw output from SLOUCH. SLOUCH input data. Likelihood plots for all half-life estimations. (ZIP 2442 kb) [file 12862_2016_778_MOESM1_ESM.zip › Additional file 1/Results Bergman's rule - body mass/Canidae_BM_temp.pdf]

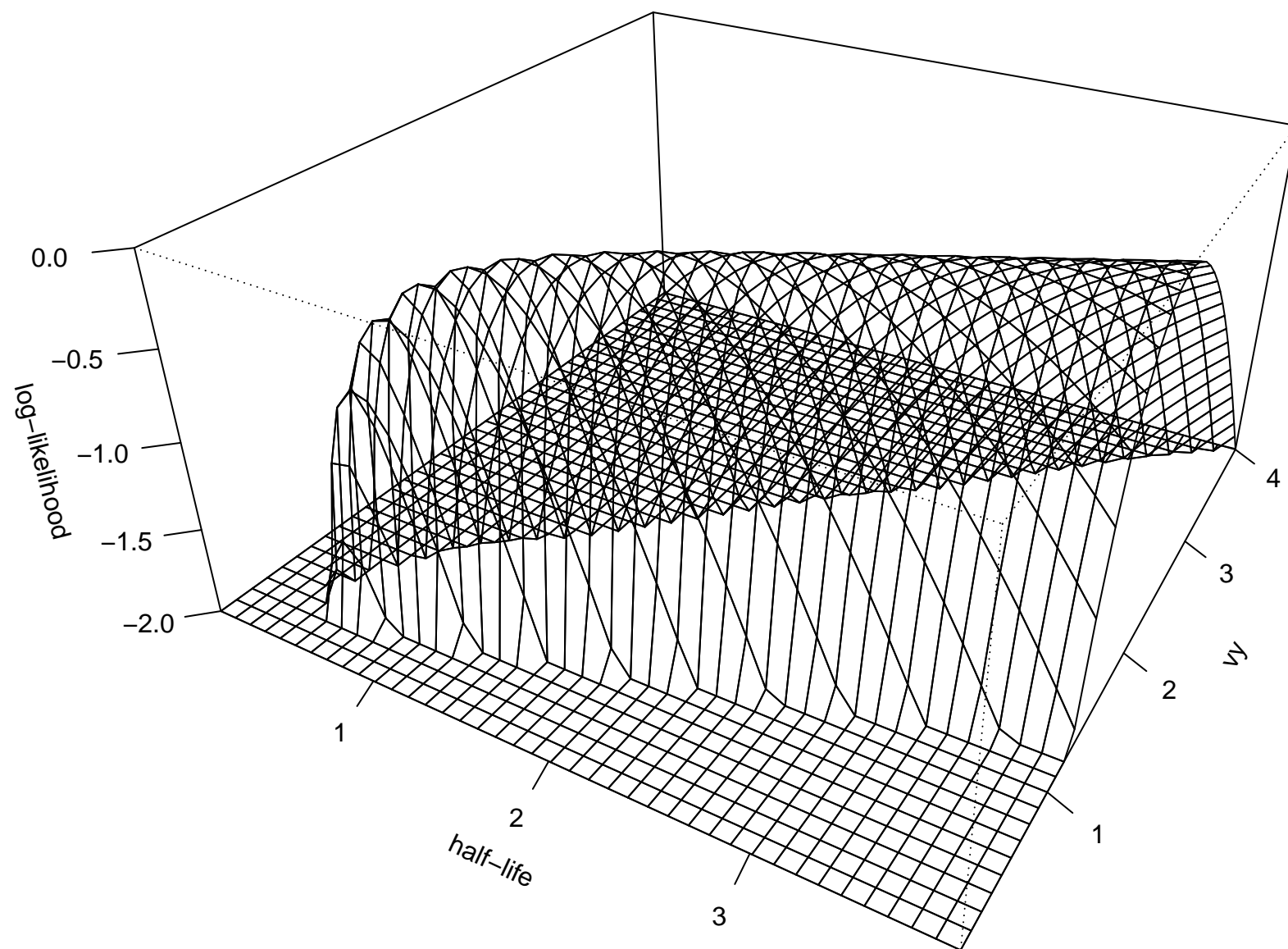

Supplement: Additional file 1: — All phylogenies used in analyses. R script for data extraction and analyses. Detailed results/raw output from SLOUCH. SLOUCH input data. Likelihood plots for all half-life estimations. (ZIP 2442 kb) [file 12862_2016_778_MOESM1_ESM.zip › Additional file 1/Results Bergman's rule - body mass/Canidae_PhySig.pdf]

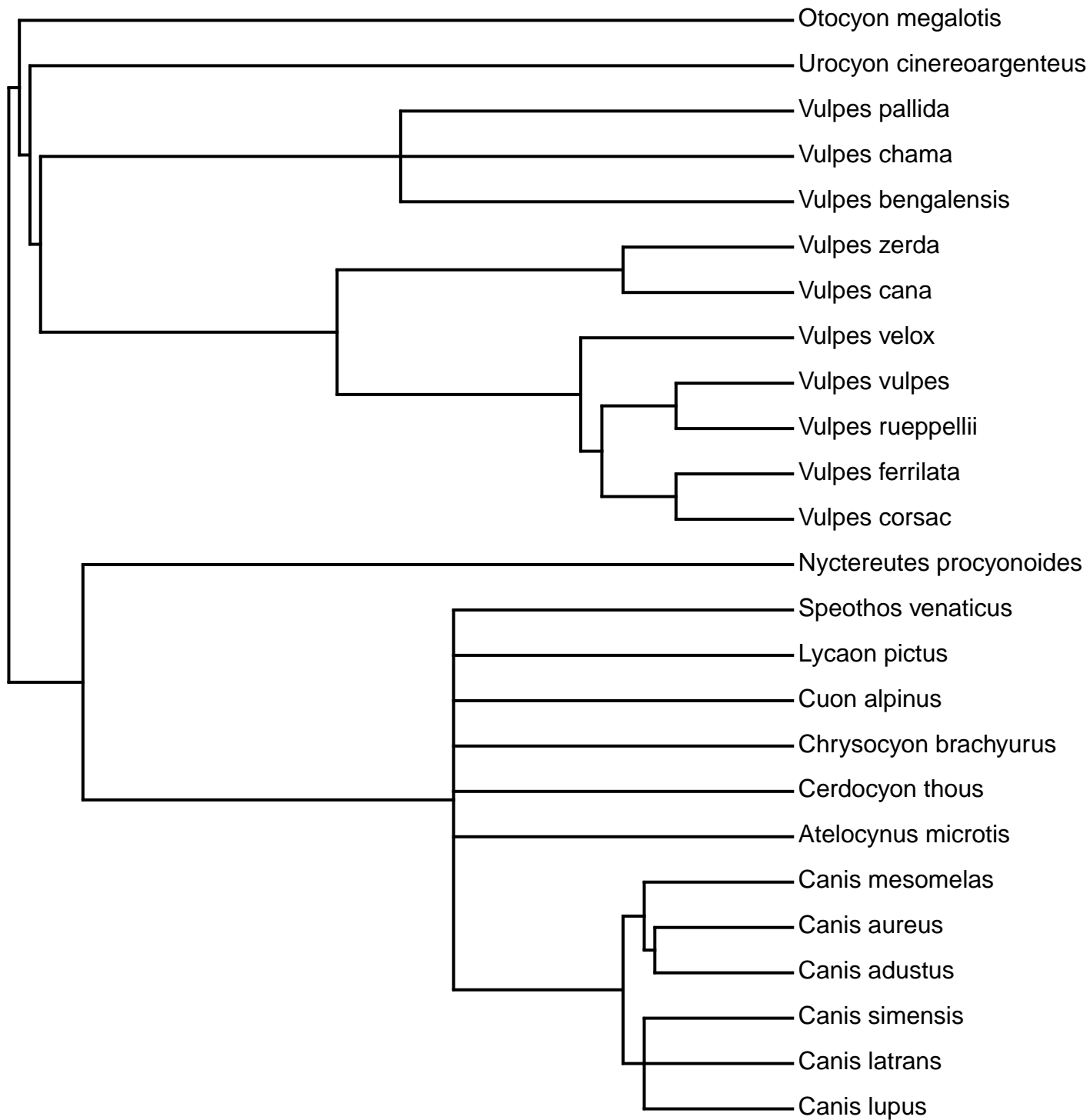

Supplement: Additional file 1: — All phylogenies used in analyses. R script for data extraction and analyses. Detailed results/raw output from SLOUCH. SLOUCH input data. Likelihood plots for all half-life estimations. (ZIP 2442 kb) [file 12862_2016_778_MOESM1_ESM.zip › Additional file 1/Results Bergman's rule - body mass/Canidae_tree.pdf]

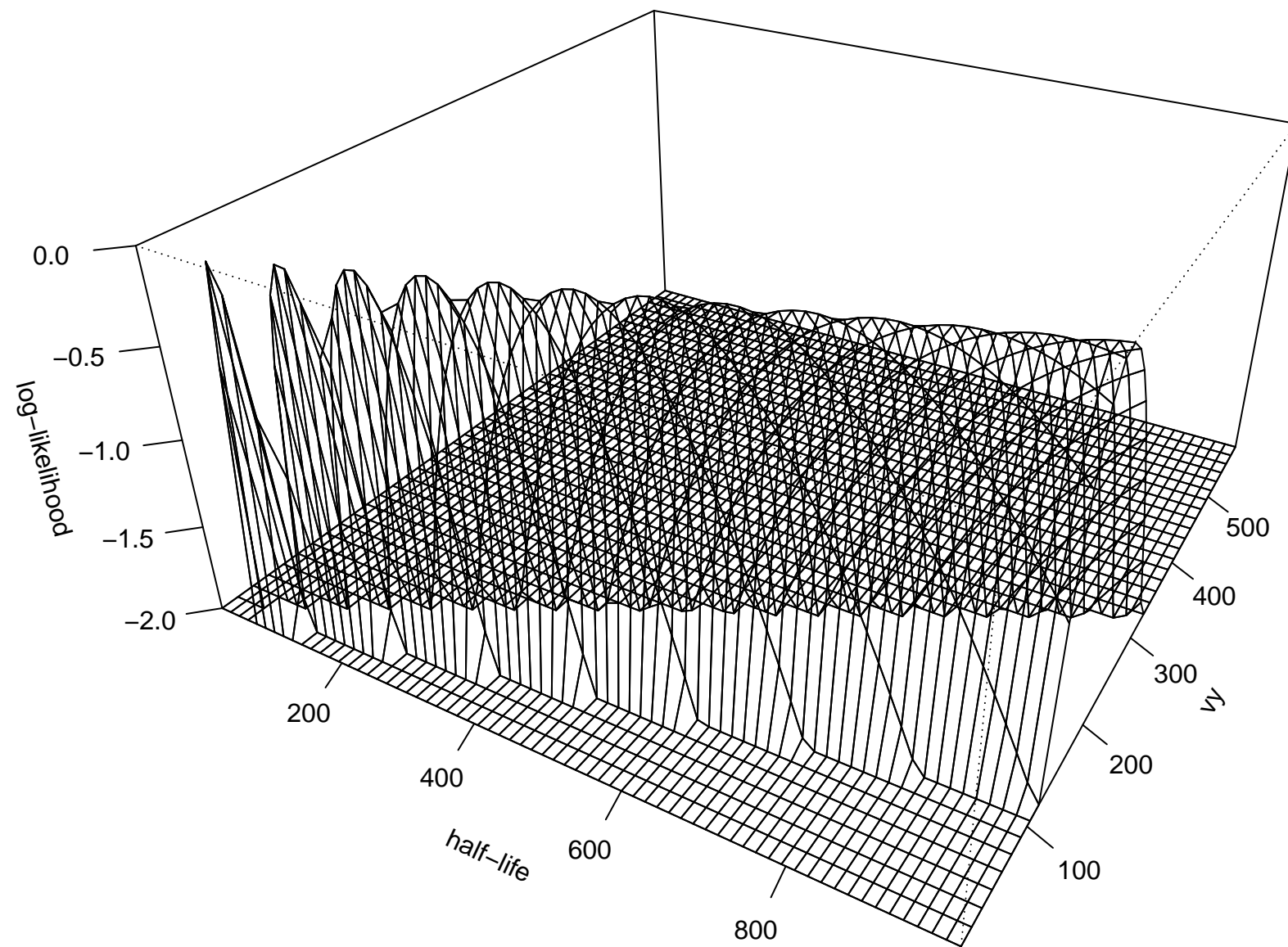

Supplement: Additional file 1: — All phylogenies used in analyses. R script for data extraction and analyses. Detailed results/raw output from SLOUCH. SLOUCH input data. Likelihood plots for all half-life estimations. (ZIP 2442 kb) [file 12862_2016_778_MOESM1_ESM.zip › Additional file 1/Results Bergman's rule - body mass/Cebidae_BM_maxlat.pdf]

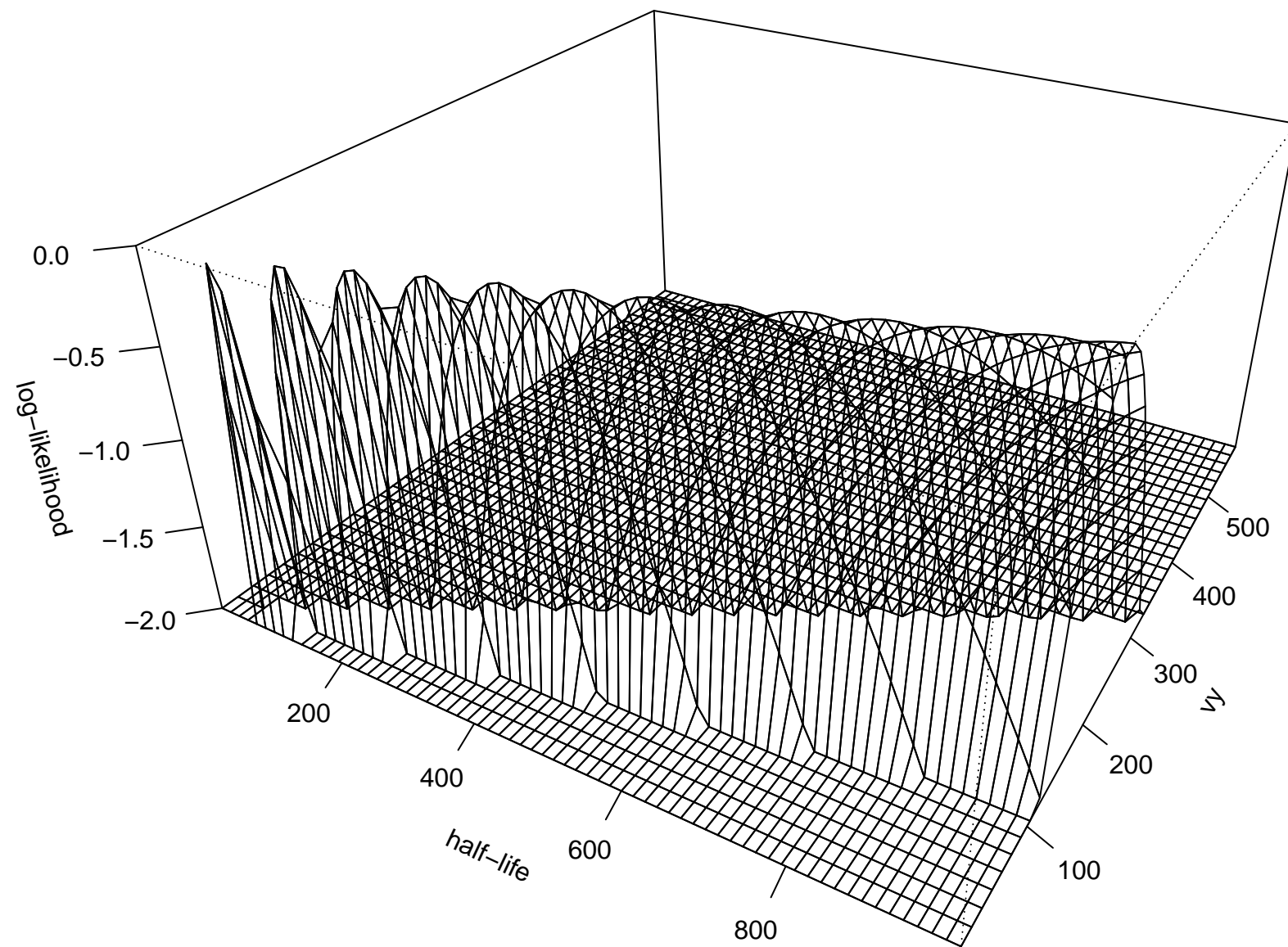

Supplement: Additional file 1: — All phylogenies used in analyses. R script for data extraction and analyses. Detailed results/raw output from SLOUCH. SLOUCH input data. Likelihood plots for all half-life estimations. (ZIP 2442 kb) [file 12862_2016_778_MOESM1_ESM.zip › Additional file 1/Results Bergman's rule - body mass/Cebidae_BM_midlat.pdf]

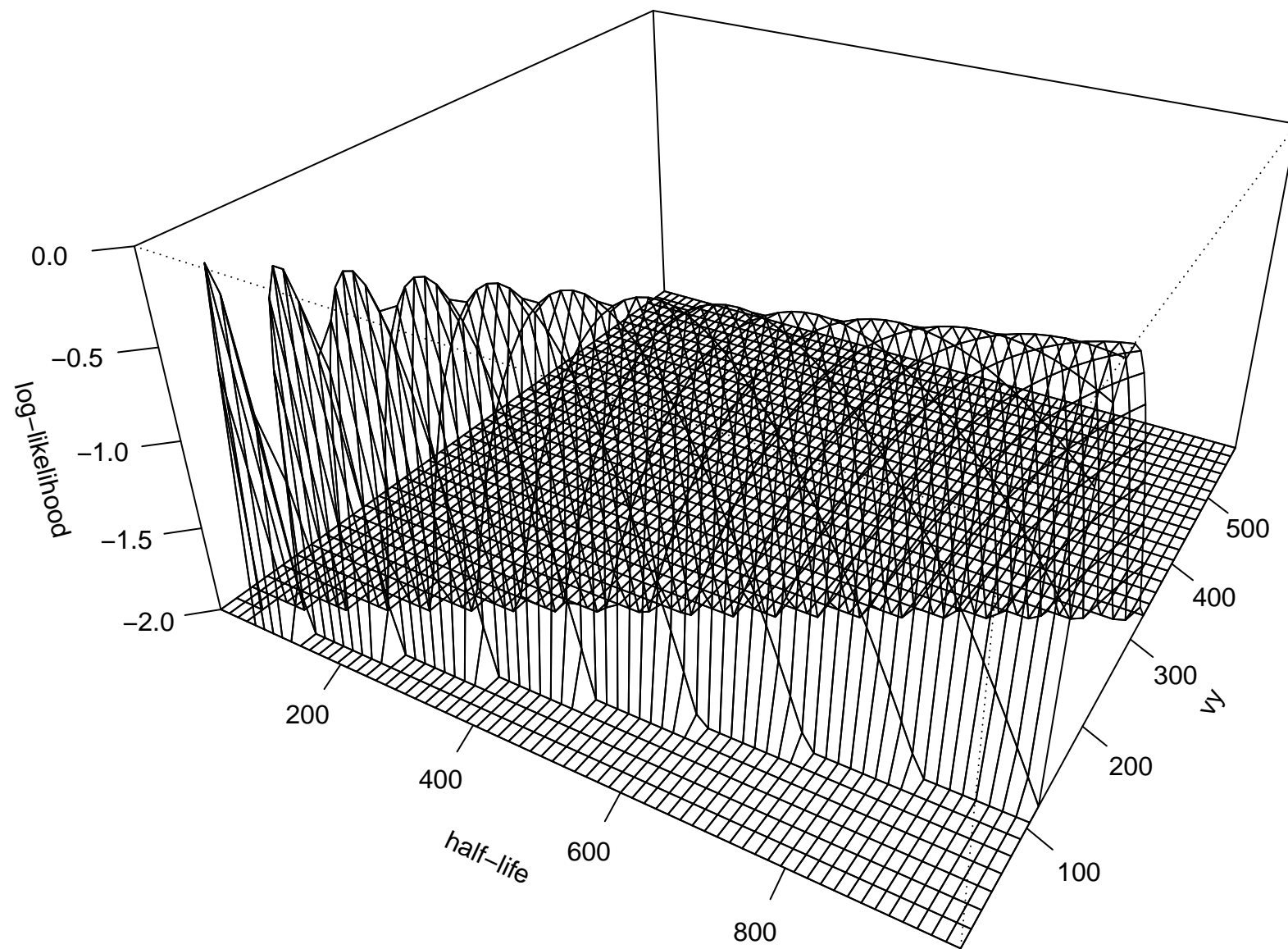

Supplement: Additional file 1: — All phylogenies used in analyses. R script for data extraction and analyses. Detailed results/raw output from SLOUCH. SLOUCH input data. Likelihood plots for all half-life estimations. (ZIP 2442 kb) [file 12862_2016_778_MOESM1_ESM.zip › Additional file 1/Results Bergman's rule - body mass/Cebidae_BM_temp.pdf]

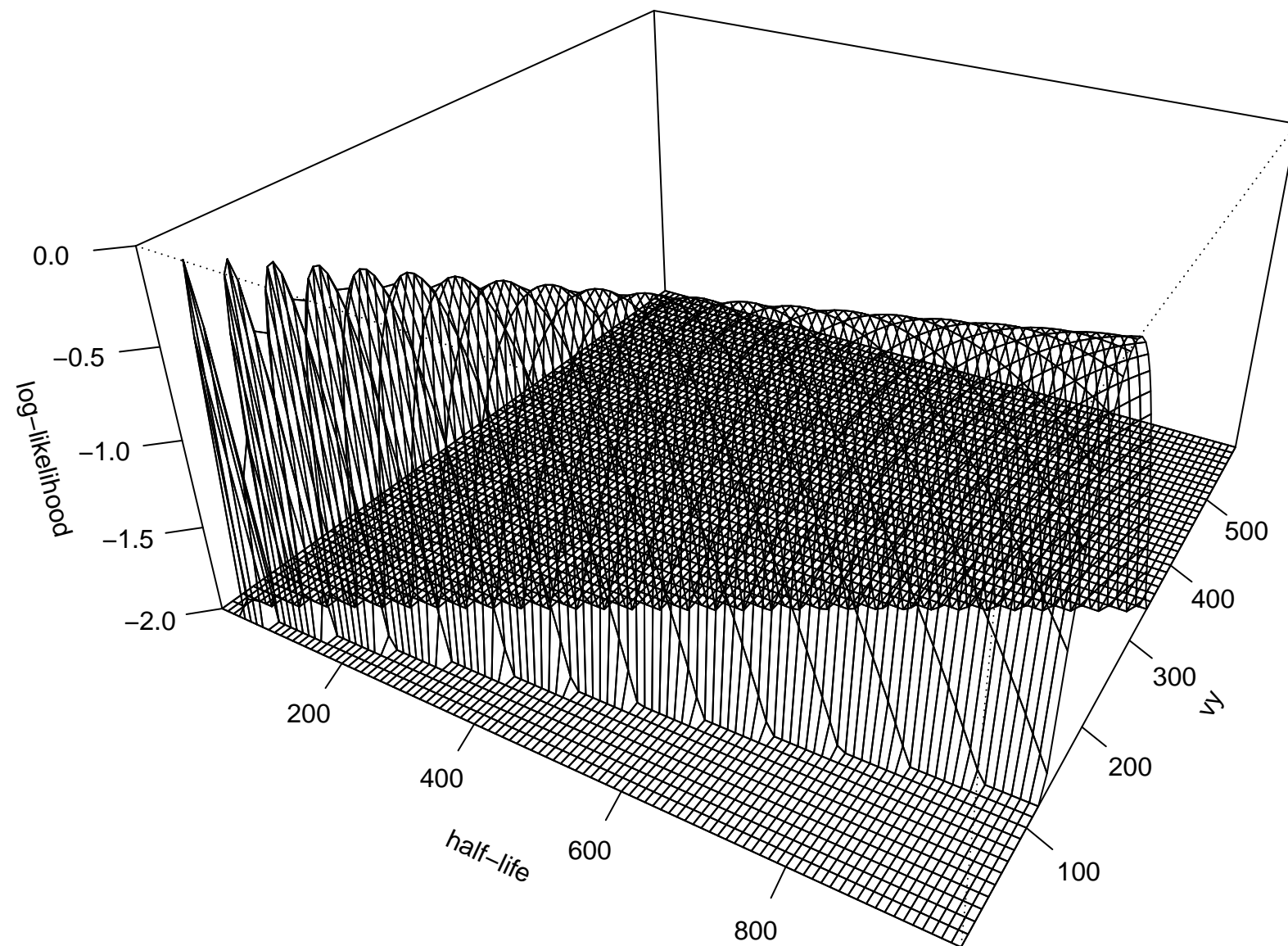

Supplement: Additional file 1: — All phylogenies used in analyses. R script for data extraction and analyses. Detailed results/raw output from SLOUCH. SLOUCH input data. Likelihood plots for all half-life estimations. (ZIP 2442 kb) [file 12862_2016_778_MOESM1_ESM.zip › Additional file 1/Results Bergman's rule - body mass/Cebidae_phySig.pdf]

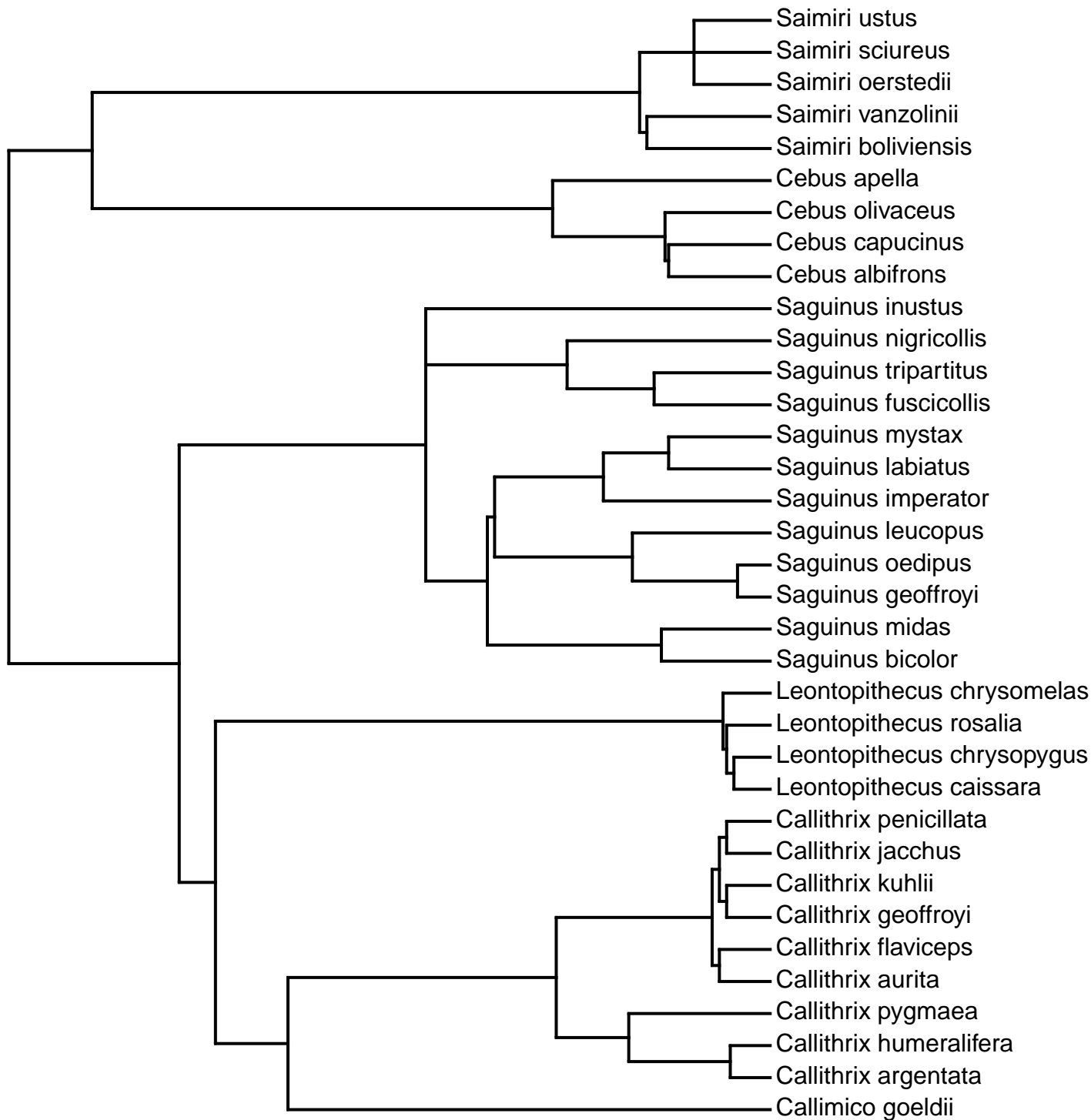

Supplement: Additional file 1: — All phylogenies used in analyses. R script for data extraction and analyses. Detailed results/raw output from SLOUCH. SLOUCH input data. Likelihood plots for all half-life estimations. (ZIP 2442 kb) [file 12862_2016_778_MOESM1_ESM.zip › Additional file 1/Results Bergman's rule - body mass/Cebidae_tree.pdf]

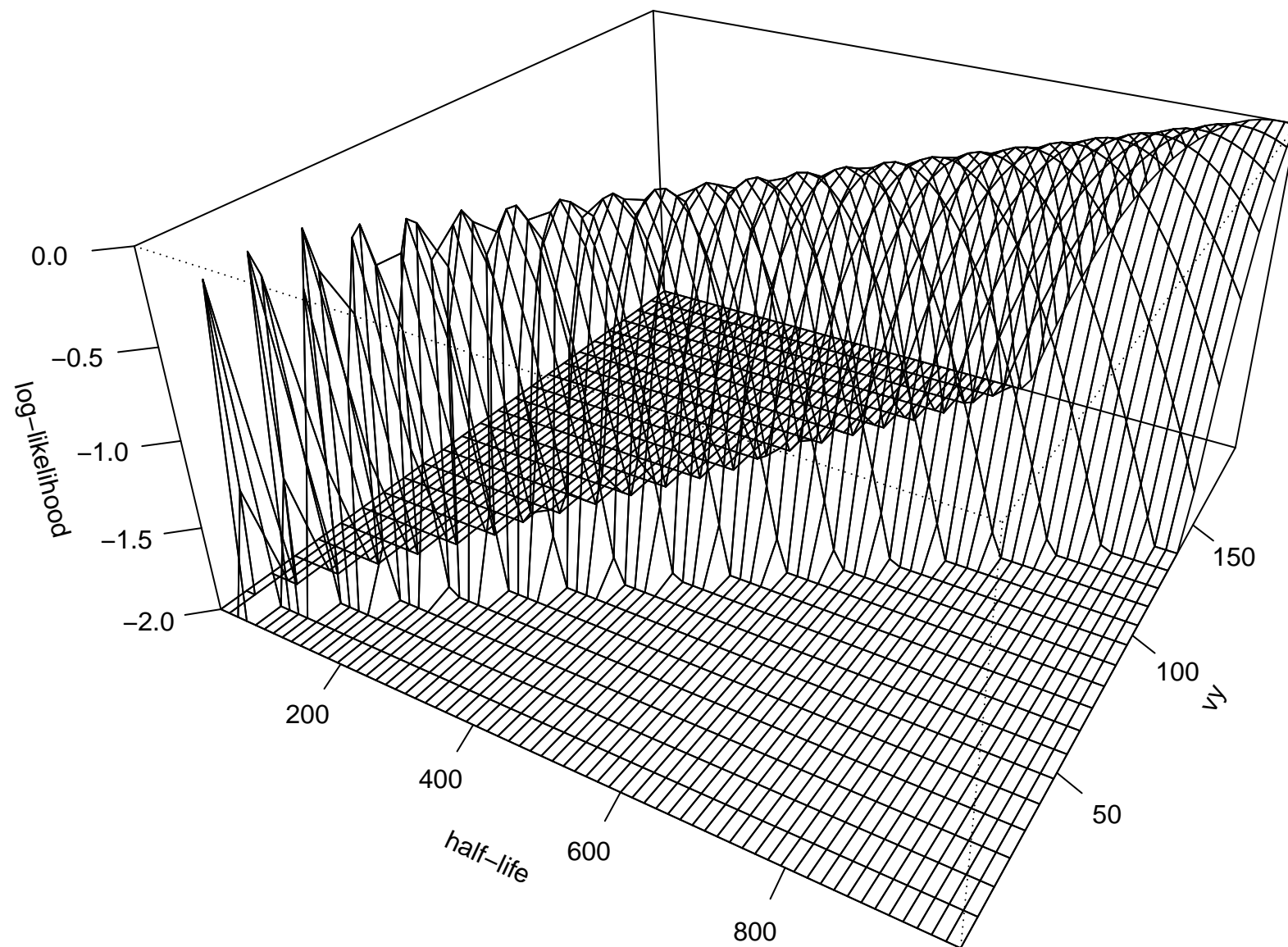

Supplement: Additional file 1: — All phylogenies used in analyses. R script for data extraction and analyses. Detailed results/raw output from SLOUCH. SLOUCH input data. Likelihood plots for all half-life estimations. (ZIP 2442 kb) [file 12862_2016_778_MOESM1_ESM.zip › Additional file 1/Results Bergman's rule - body mass/Cercopithecidae_BM_maxlat.pdf]

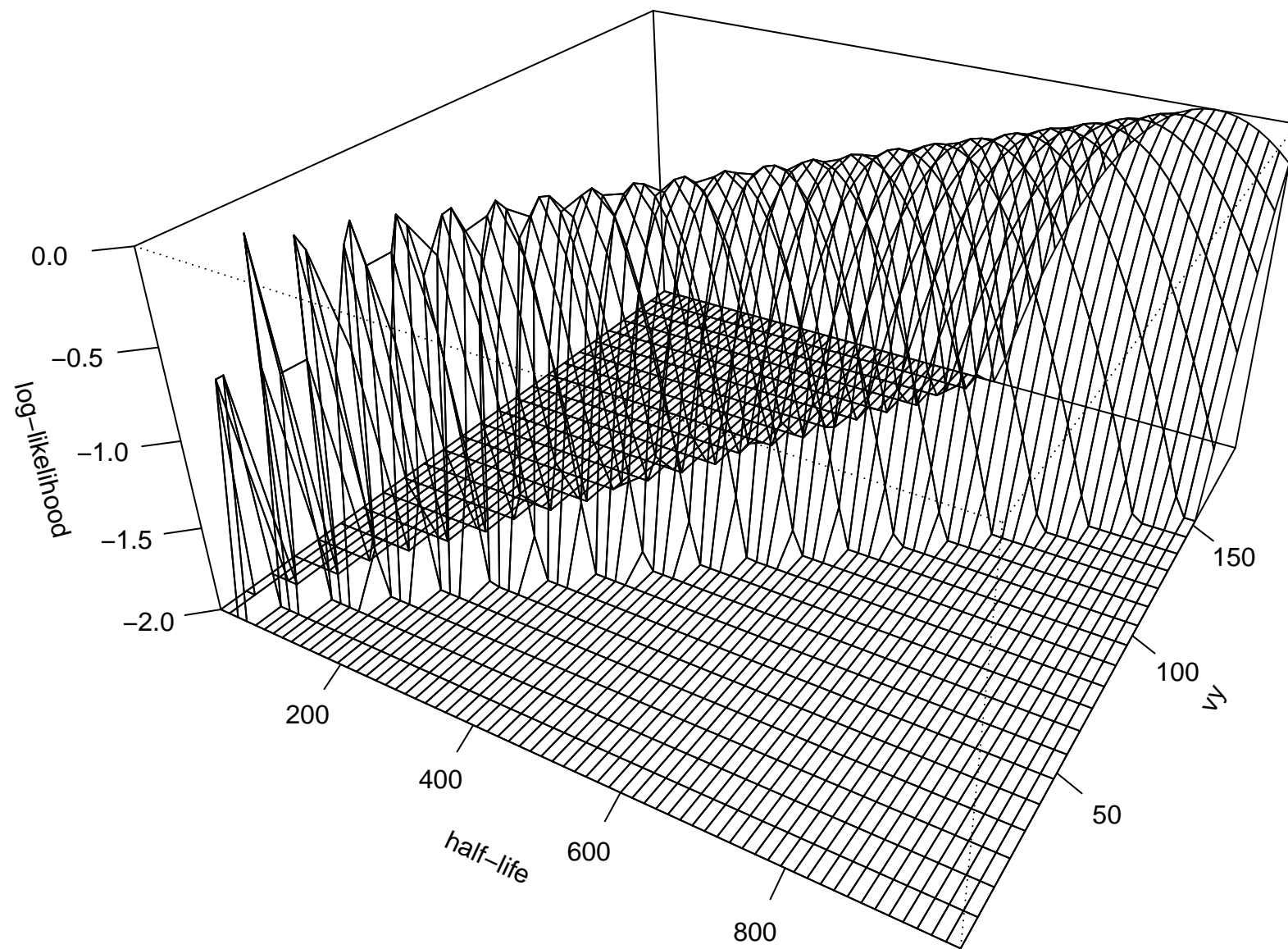

Supplement: Additional file 1: — All phylogenies used in analyses. R script for data extraction and analyses. Detailed results/raw output from SLOUCH. SLOUCH input data. Likelihood plots for all half-life estimations. (ZIP 2442 kb) [file 12862_2016_778_MOESM1_ESM.zip › Additional file 1/Results Bergman's rule - body mass/Cercopithecidae_BM_midlat.pdf]

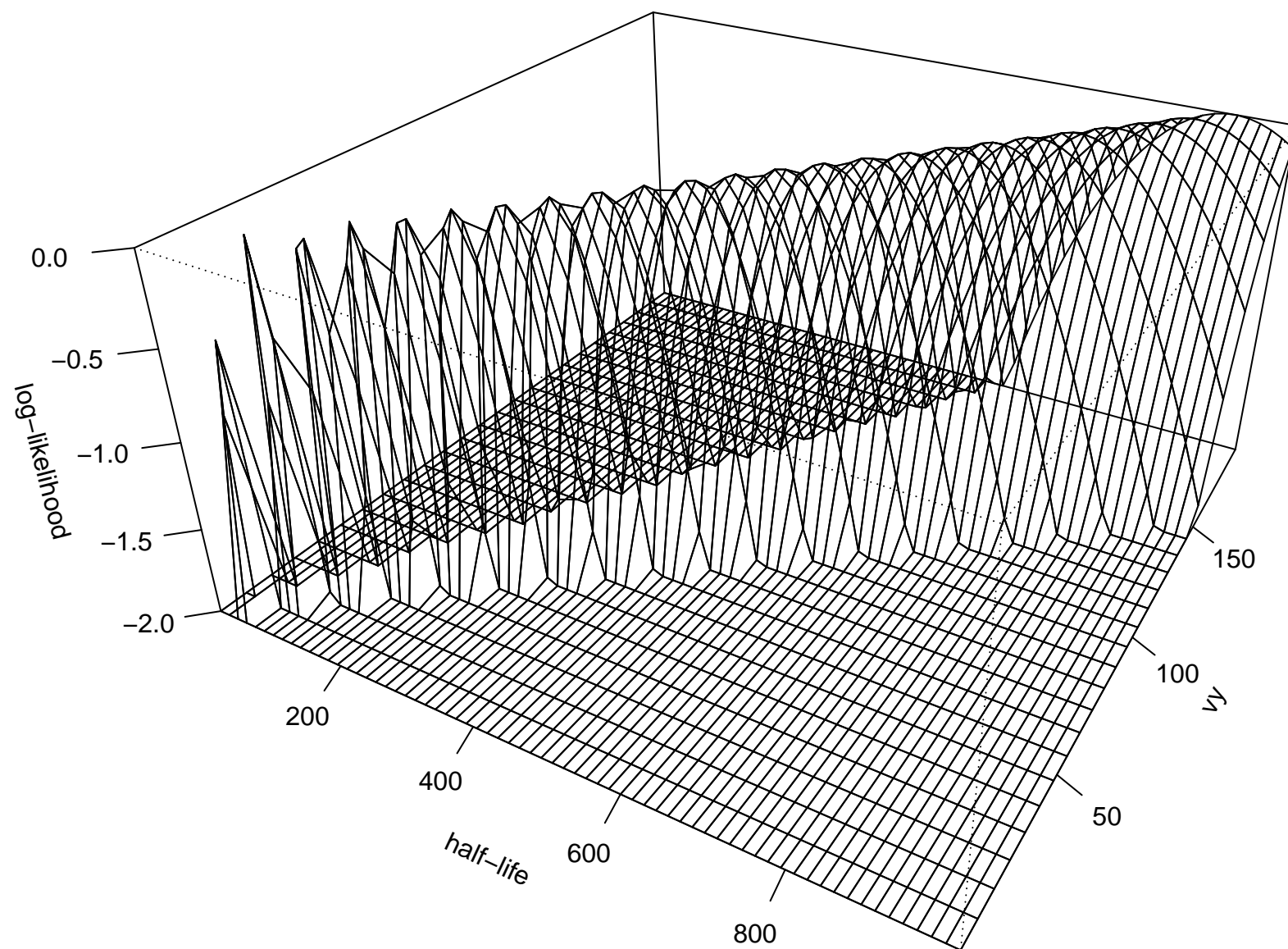

Supplement: Additional file 1: — All phylogenies used in analyses. R script for data extraction and analyses. Detailed results/raw output from SLOUCH. SLOUCH input data. Likelihood plots for all half-life estimations. (ZIP 2442 kb) [file 12862_2016_778_MOESM1_ESM.zip › Additional file 1/Results Bergman's rule - body mass/Cercopithecidae_BM_temp.pdf]

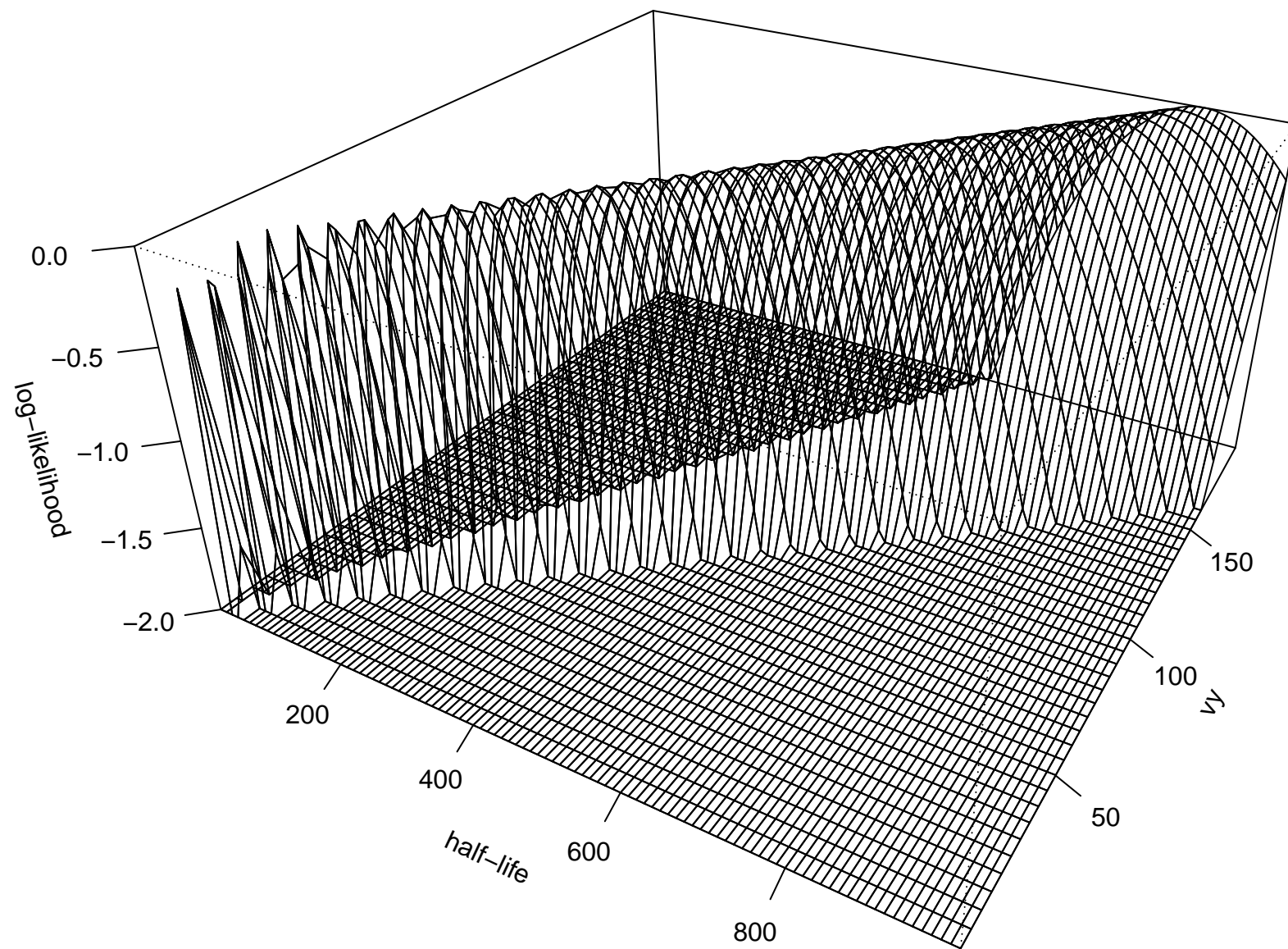

Supplement: Additional file 1: — All phylogenies used in analyses. R script for data extraction and analyses. Detailed results/raw output from SLOUCH. SLOUCH input data. Likelihood plots for all half-life estimations. (ZIP 2442 kb) [file 12862_2016_778_MOESM1_ESM.zip › Additional file 1/Results Bergman's rule - body mass/Cercopithecidae_phySig.pdf]

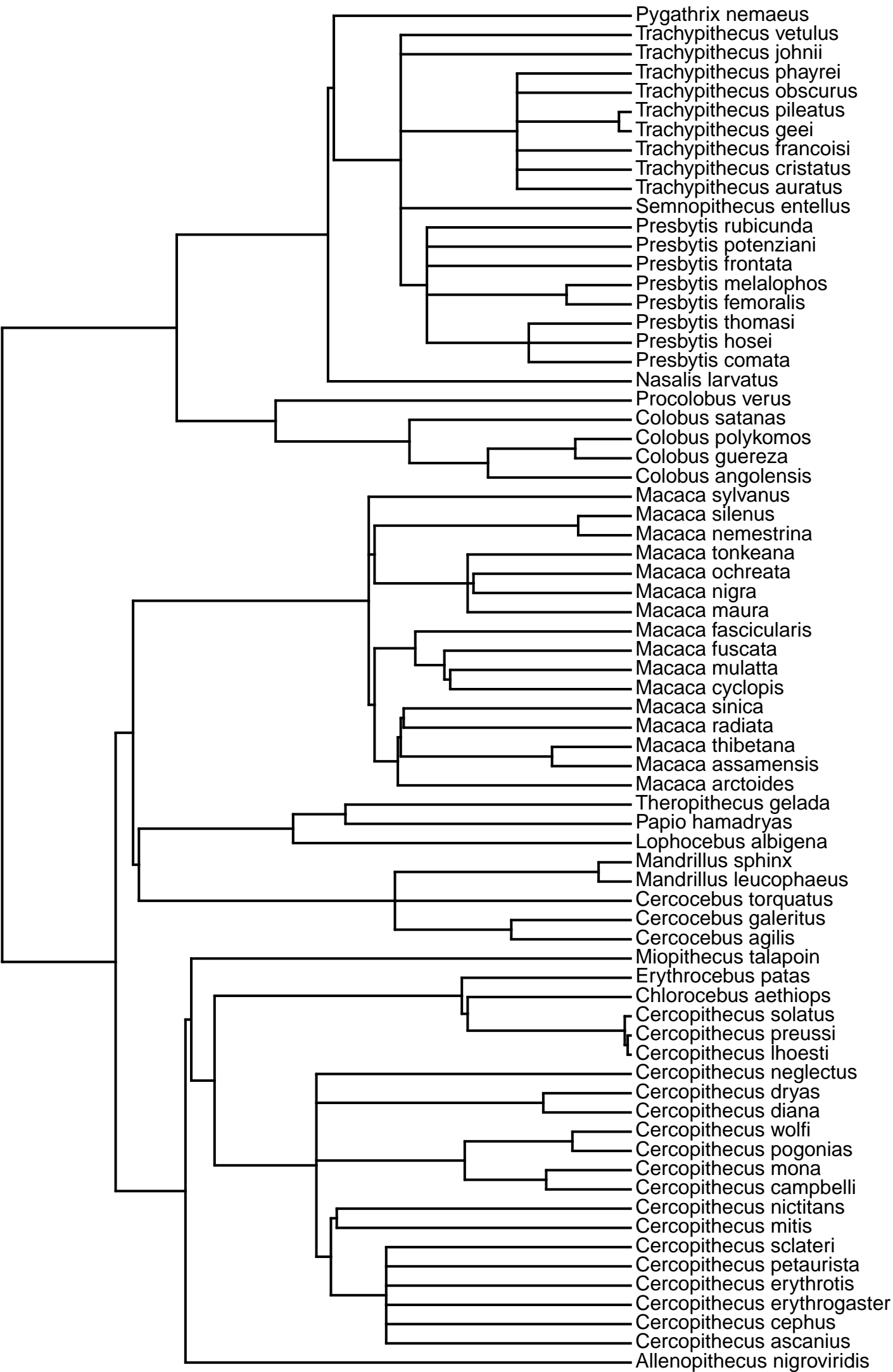

Supplement: Additional file 1: — All phylogenies used in analyses. R script for data extraction and analyses. Detailed results/raw output from SLOUCH. SLOUCH input data. Likelihood plots for all half-life estimations. (ZIP 2442 kb) [file 12862_2016_778_MOESM1_ESM.zip › Additional file 1/Results Bergman's rule - body mass/Cercopithecidae_tree.pdf]

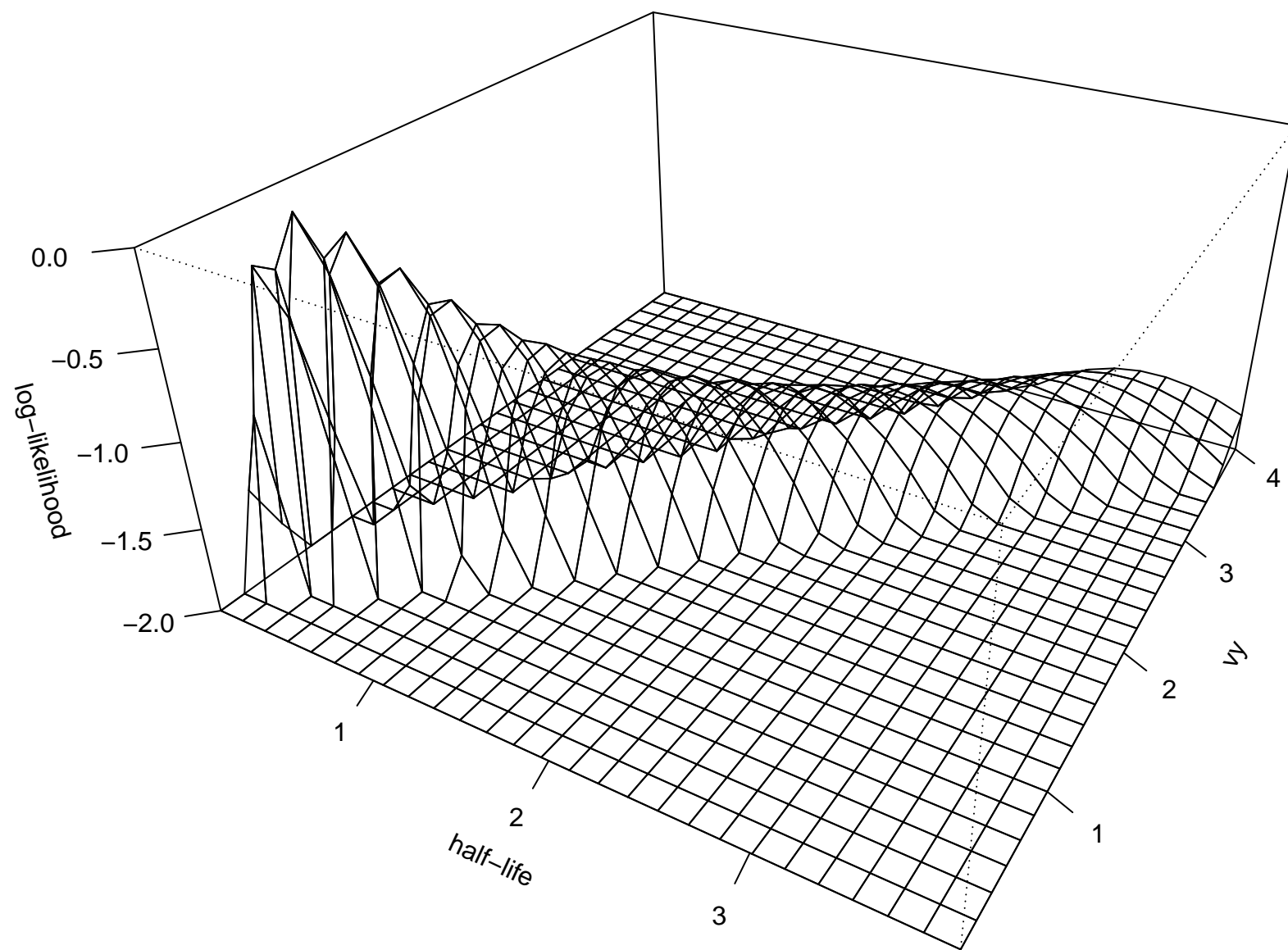

Supplement: Additional file 1: — All phylogenies used in analyses. R script for data extraction and analyses. Detailed results/raw output from SLOUCH. SLOUCH input data. Likelihood plots for all half-life estimations. (ZIP 2442 kb) [file 12862_2016_778_MOESM1_ESM.zip › Additional file 1/Results Bergman's rule - body mass/Cervidae_BM_maxlat.pdf]

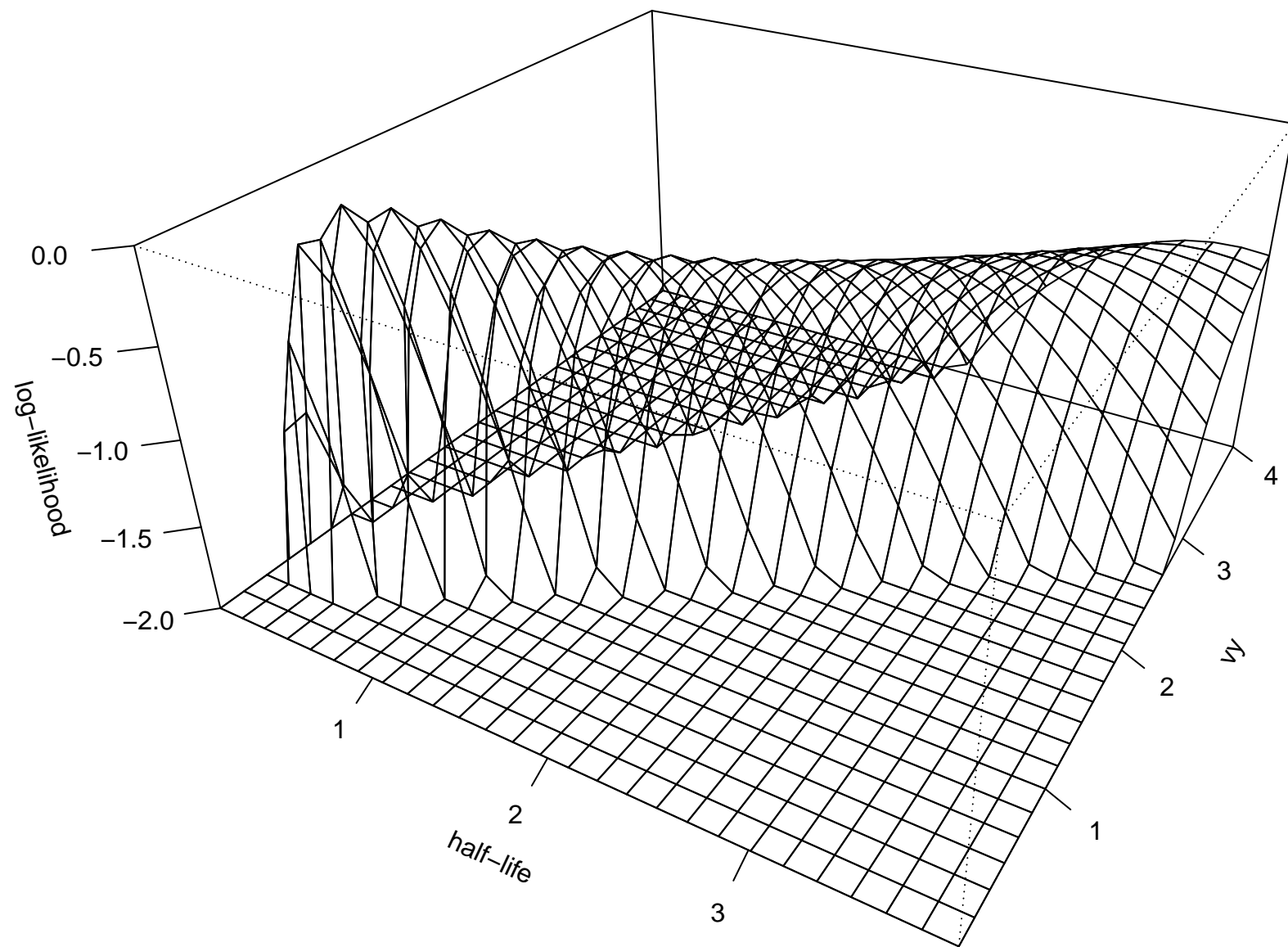

Supplement: Additional file 1: — All phylogenies used in analyses. R script for data extraction and analyses. Detailed results/raw output from SLOUCH. SLOUCH input data. Likelihood plots for all half-life estimations. (ZIP 2442 kb) [file 12862_2016_778_MOESM1_ESM.zip › Additional file 1/Results Bergman's rule - body mass/Cervidae_BM_midlat.pdf]

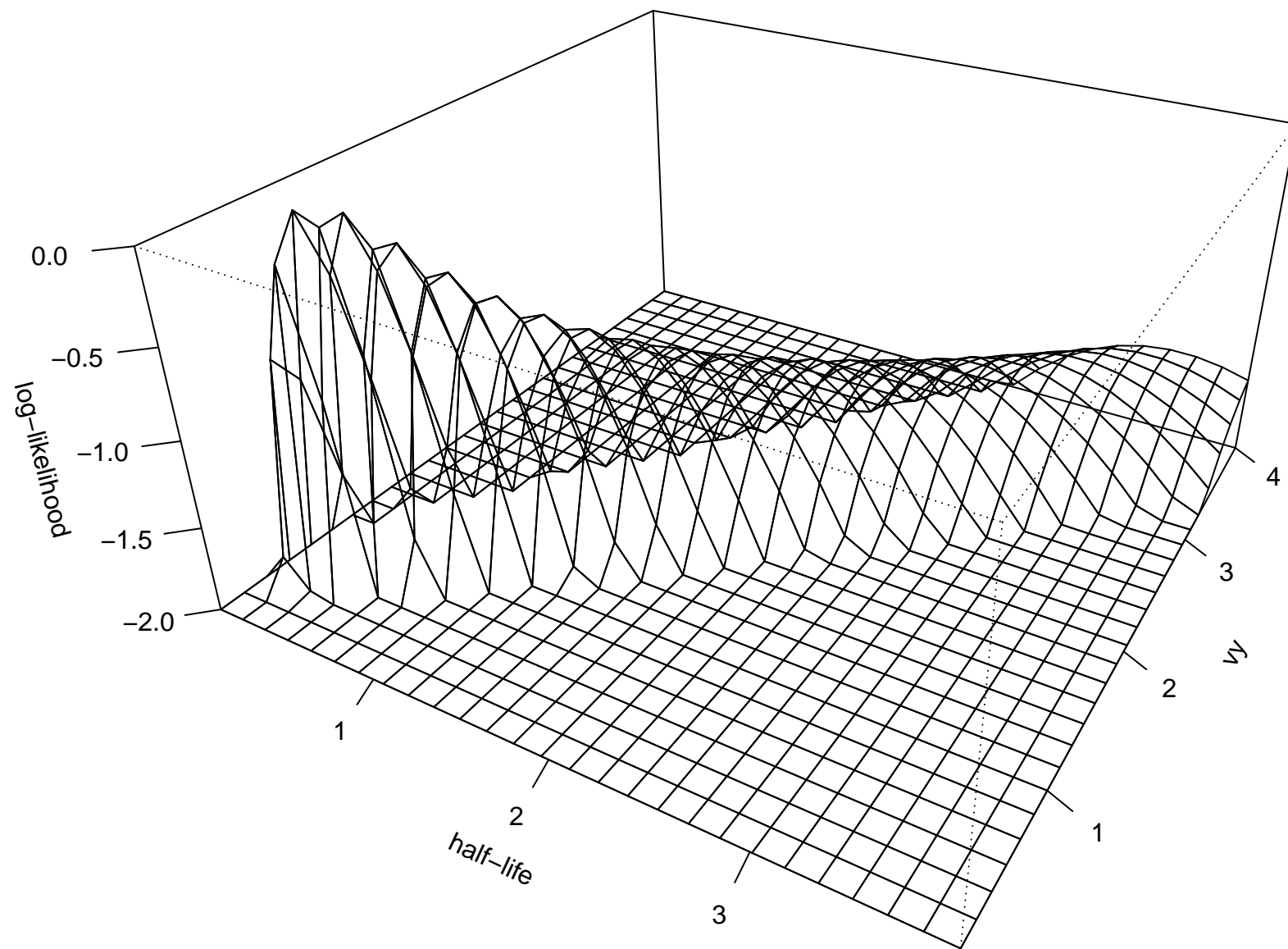

Supplement: Additional file 1: — All phylogenies used in analyses. R script for data extraction and analyses. Detailed results/raw output from SLOUCH. SLOUCH input data. Likelihood plots for all half-life estimations. (ZIP 2442 kb) [file 12862_2016_778_MOESM1_ESM.zip › Additional file 1/Results Bergman's rule - body mass/Cervidae_BM_temp.pdf]

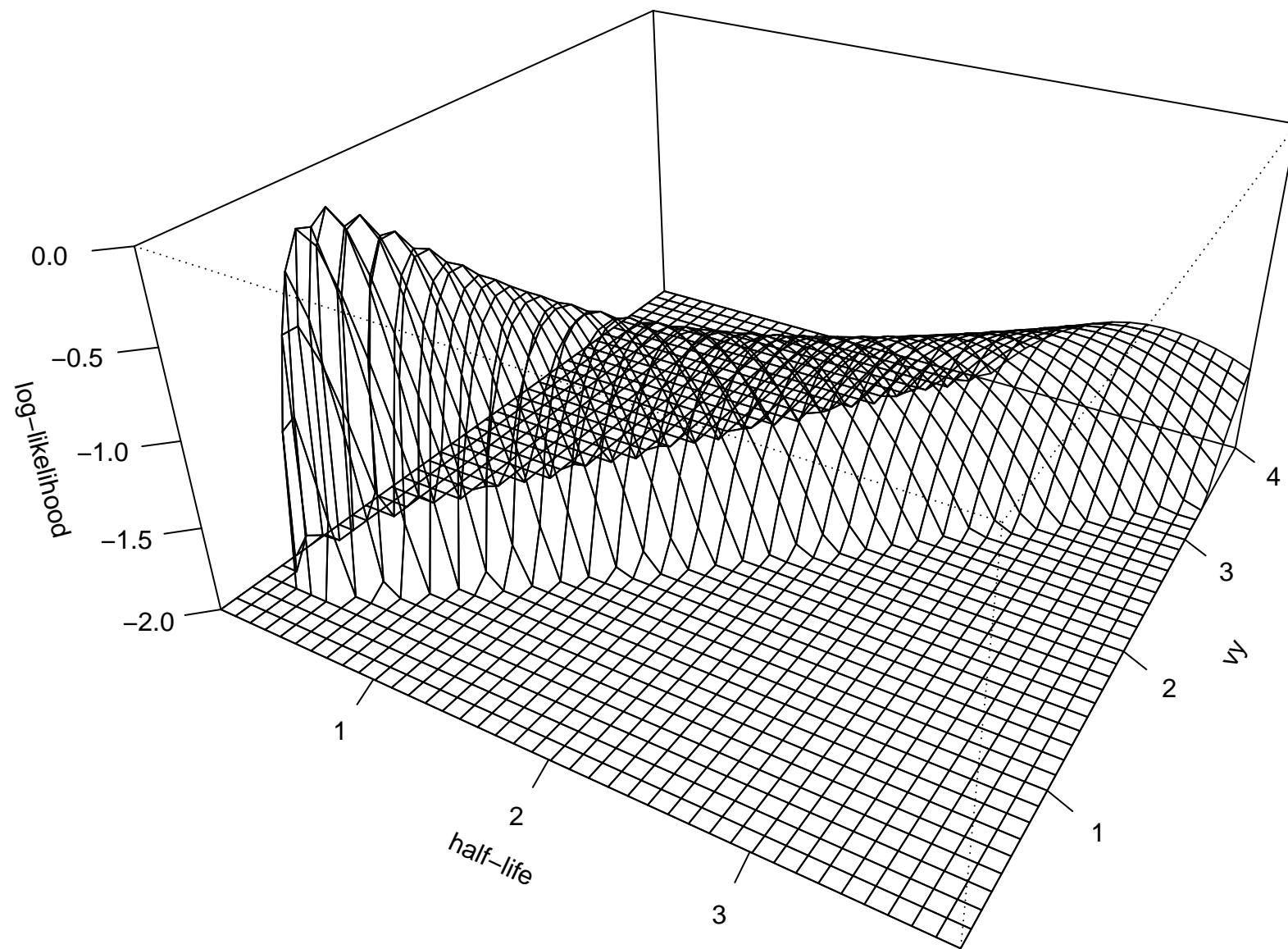

Supplement: Additional file 1: — All phylogenies used in analyses. R script for data extraction and analyses. Detailed results/raw output from SLOUCH. SLOUCH input data. Likelihood plots for all half-life estimations. (ZIP 2442 kb) [file 12862_2016_778_MOESM1_ESM.zip › Additional file 1/Results Bergman's rule - body mass/Cervidae_phySig.pdf]

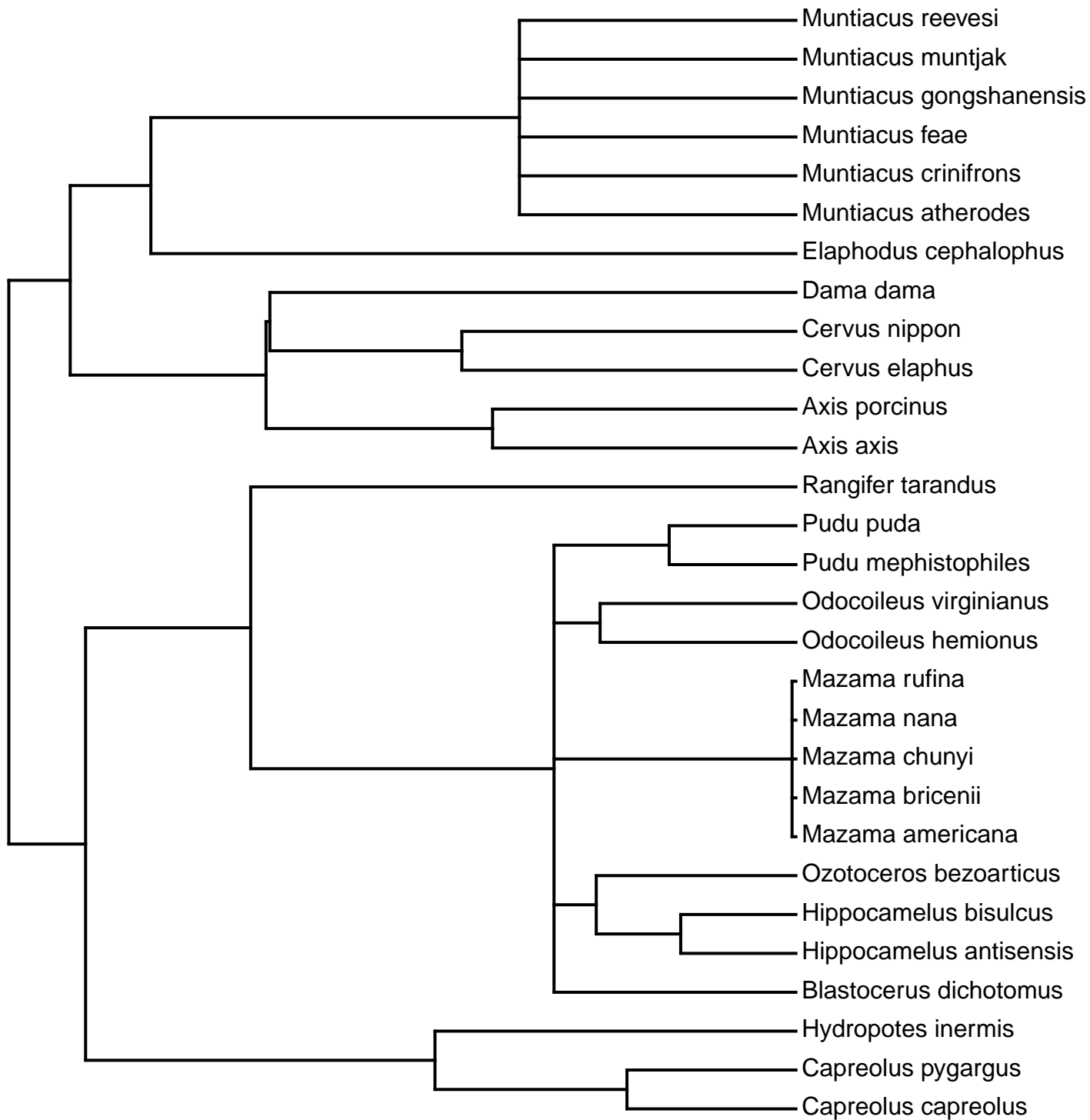

Supplement: Additional file 1: — All phylogenies used in analyses. R script for data extraction and analyses. Detailed results/raw output from SLOUCH. SLOUCH input data. Likelihood plots for all half-life estimations. (ZIP 2442 kb) [file 12862_2016_778_MOESM1_ESM.zip › Additional file 1/Results Bergman's rule - body mass/Cervidae_tree.pdf]

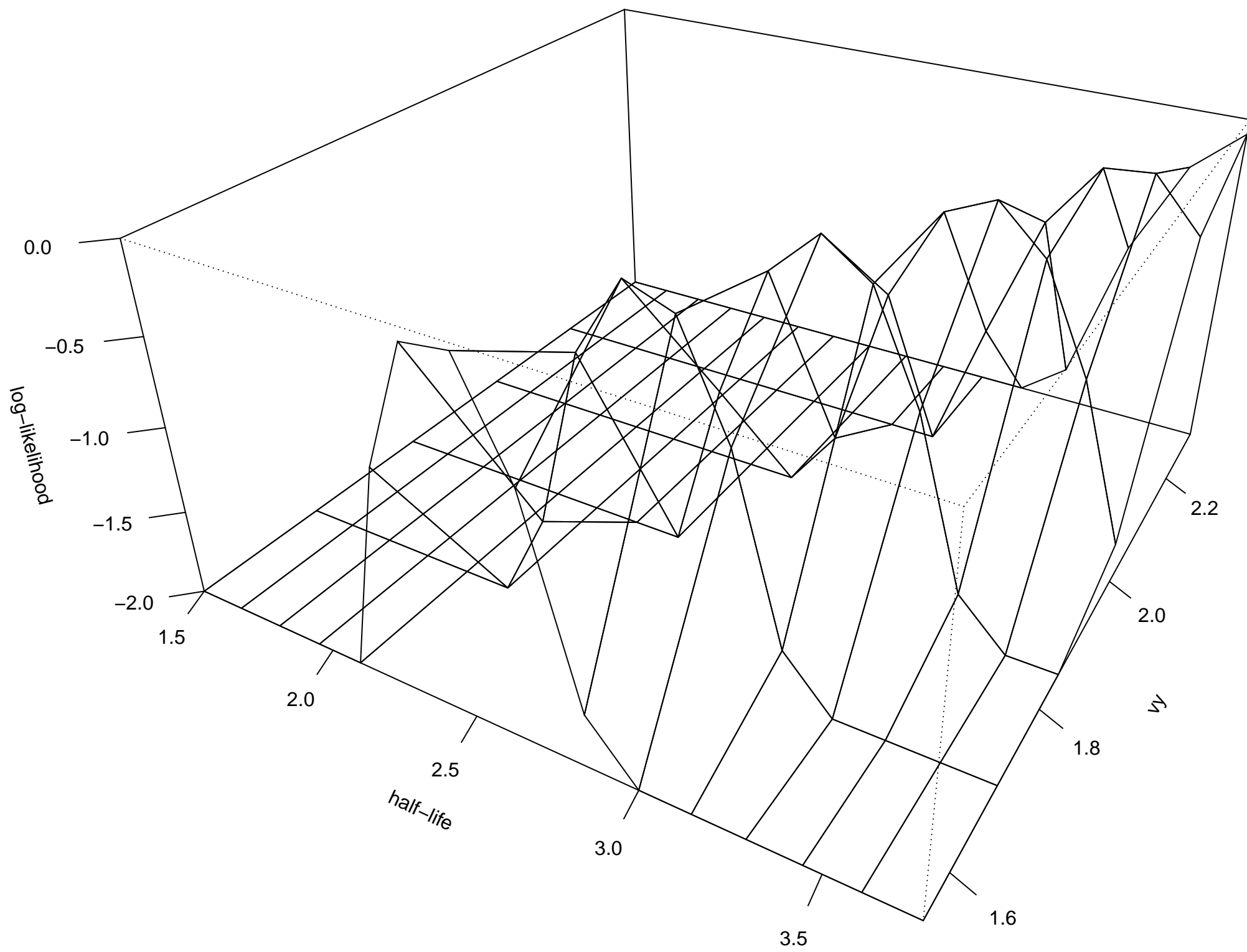

Supplement: Additional file 1: — All phylogenies used in analyses. R script for data extraction and analyses. Detailed results/raw output from SLOUCH. SLOUCH input data. Likelihood plots for all half-life estimations. (ZIP 2442 kb) [file 12862_2016_778_MOESM1_ESM.zip › Additional file 1/Results Bergman's rule - body mass/Cricetidae_BM_maxlat.pdf]

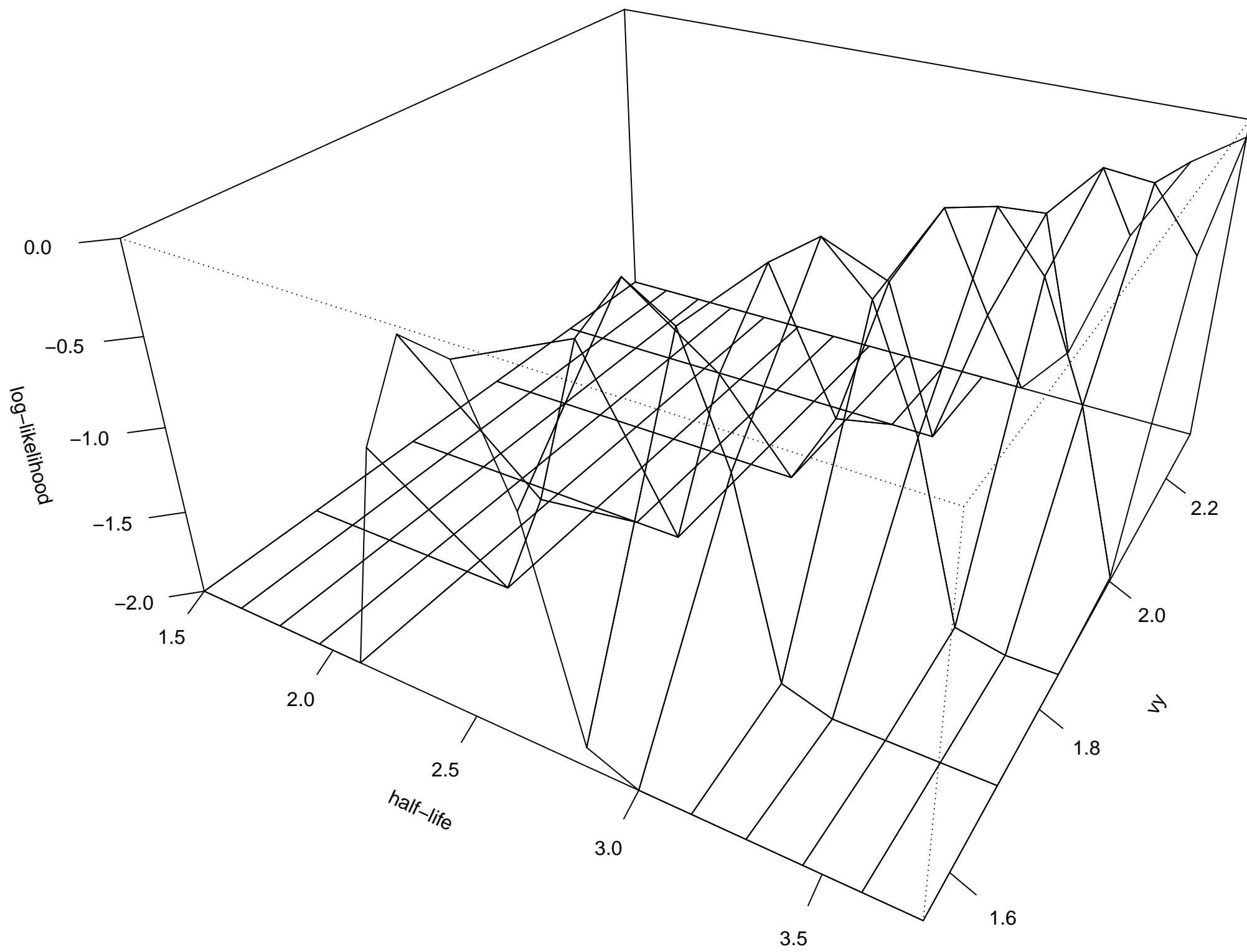

Supplement: Additional file 1: — All phylogenies used in analyses. R script for data extraction and analyses. Detailed results/raw output from SLOUCH. SLOUCH input data. Likelihood plots for all half-life estimations. (ZIP 2442 kb) [file 12862_2016_778_MOESM1_ESM.zip › Additional file 1/Results Bergman's rule - body mass/Cricetidae_BM_midlat.pdf]

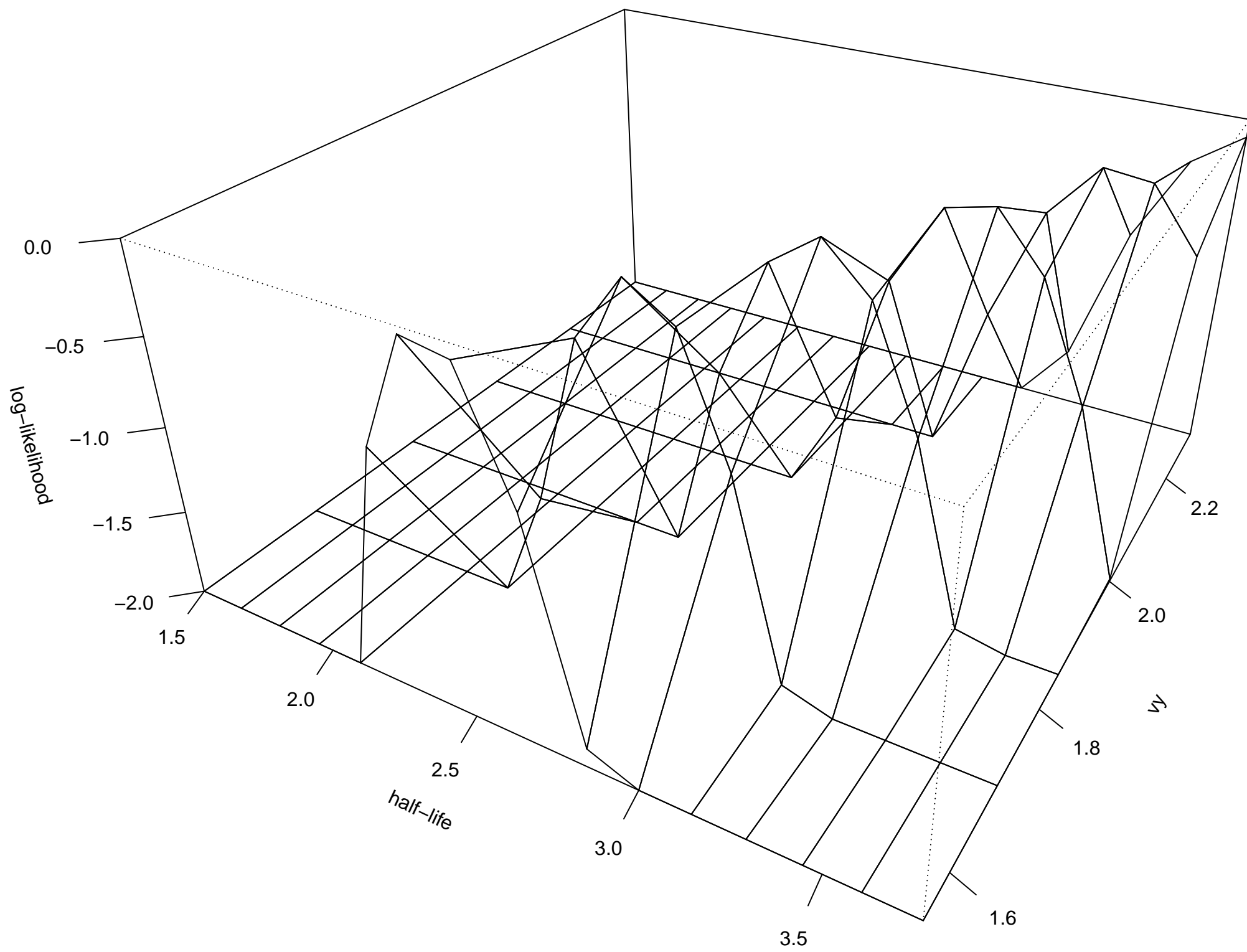

Supplement: Additional file 1: — All phylogenies used in analyses. R script for data extraction and analyses. Detailed results/raw output from SLOUCH. SLOUCH input data. Likelihood plots for all half-life estimations. (ZIP 2442 kb) [file 12862_2016_778_MOESM1_ESM.zip › Additional file 1/Results Bergman's rule - body mass/Cricetidae_BM_temp.pdf]

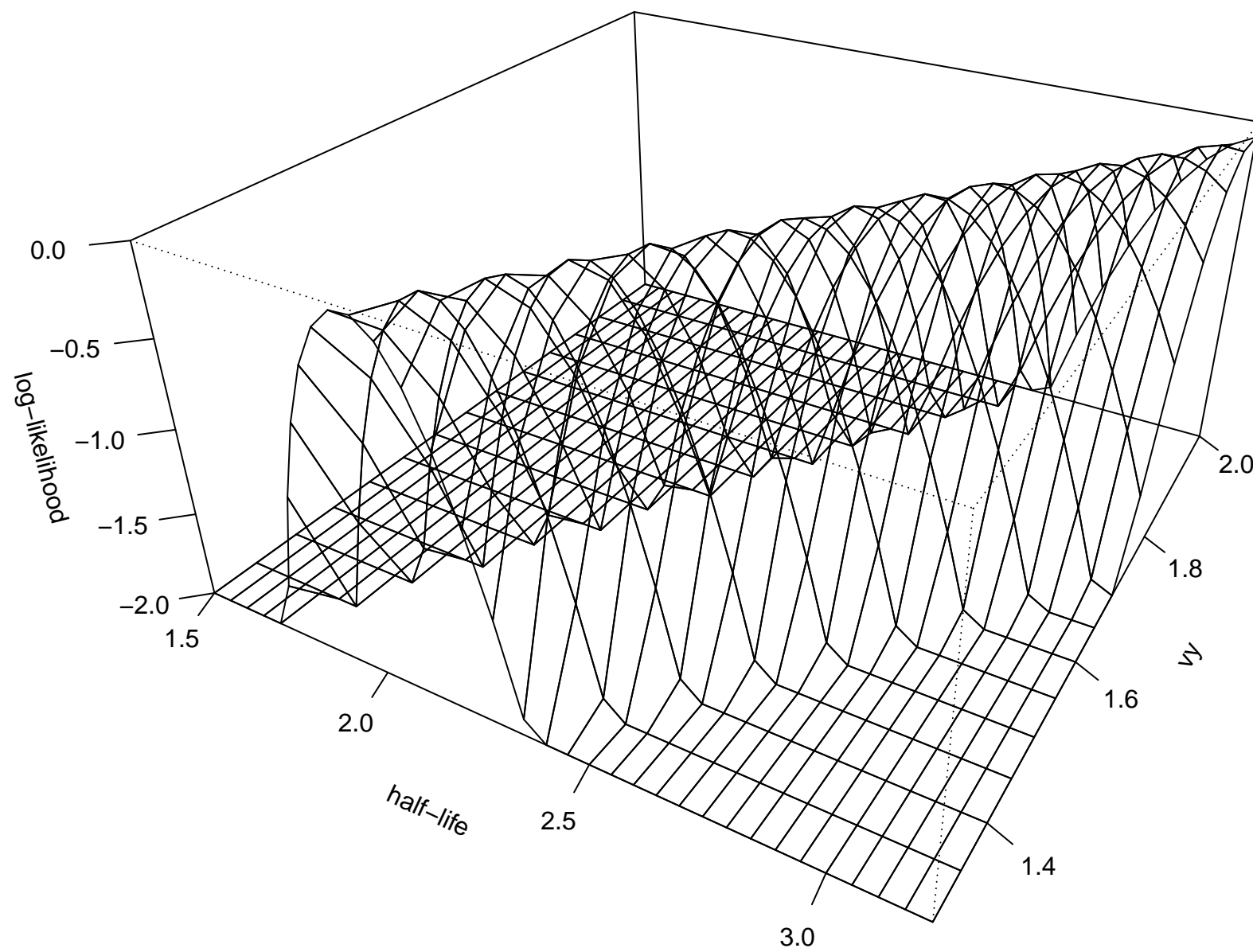

Supplement: Additional file 1: — All phylogenies used in analyses. R script for data extraction and analyses. Detailed results/raw output from SLOUCH. SLOUCH input data. Likelihood plots for all half-life estimations. (ZIP 2442 kb) [file 12862_2016_778_MOESM1_ESM.zip › Additional file 1/Results Bergman's rule - body mass/Cricetidae_phySig.pdf]

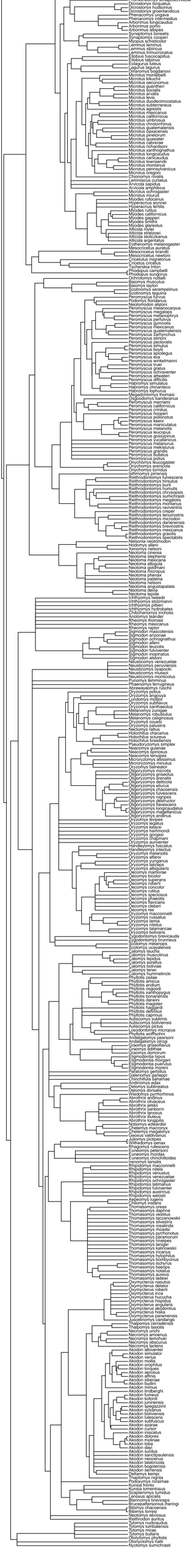

Supplement: Additional file 1: — All phylogenies used in analyses. R script for data extraction and analyses. Detailed results/raw output from SLOUCH. SLOUCH input data. Likelihood plots for all half-life estimations. (ZIP 2442 kb) [file 12862_2016_778_MOESM1_ESM.zip › Additional file 1/Results Bergman's rule - body mass/Cricetidae_tree.pdf]

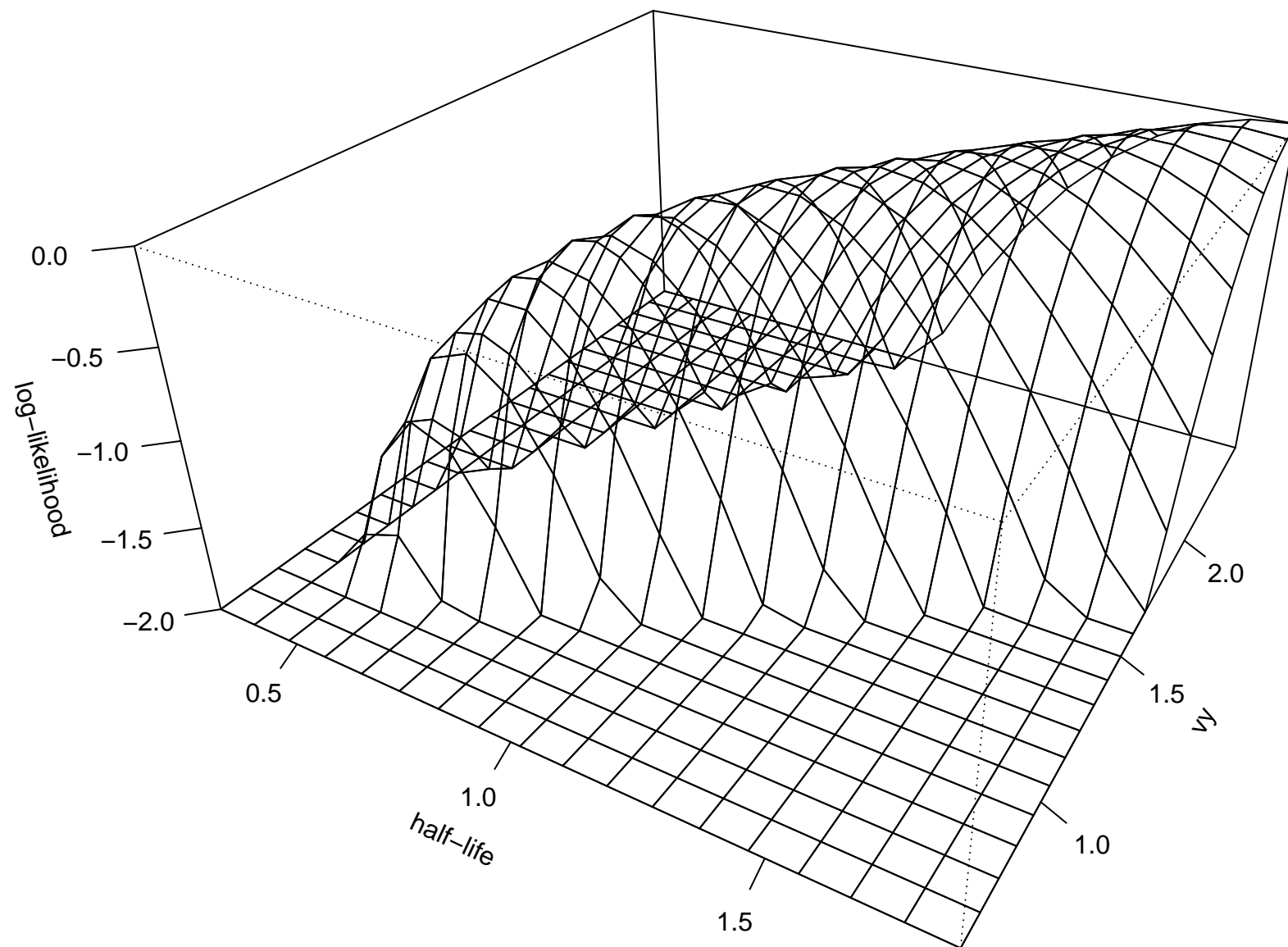

Supplement: Additional file 1: — All phylogenies used in analyses. R script for data extraction and analyses. Detailed results/raw output from SLOUCH. SLOUCH input data. Likelihood plots for all half-life estimations. (ZIP 2442 kb) [file 12862_2016_778_MOESM1_ESM.zip › Additional file 1/Results Bergman's rule - body mass/Dasyuridae_BM_maxlat.pdf]

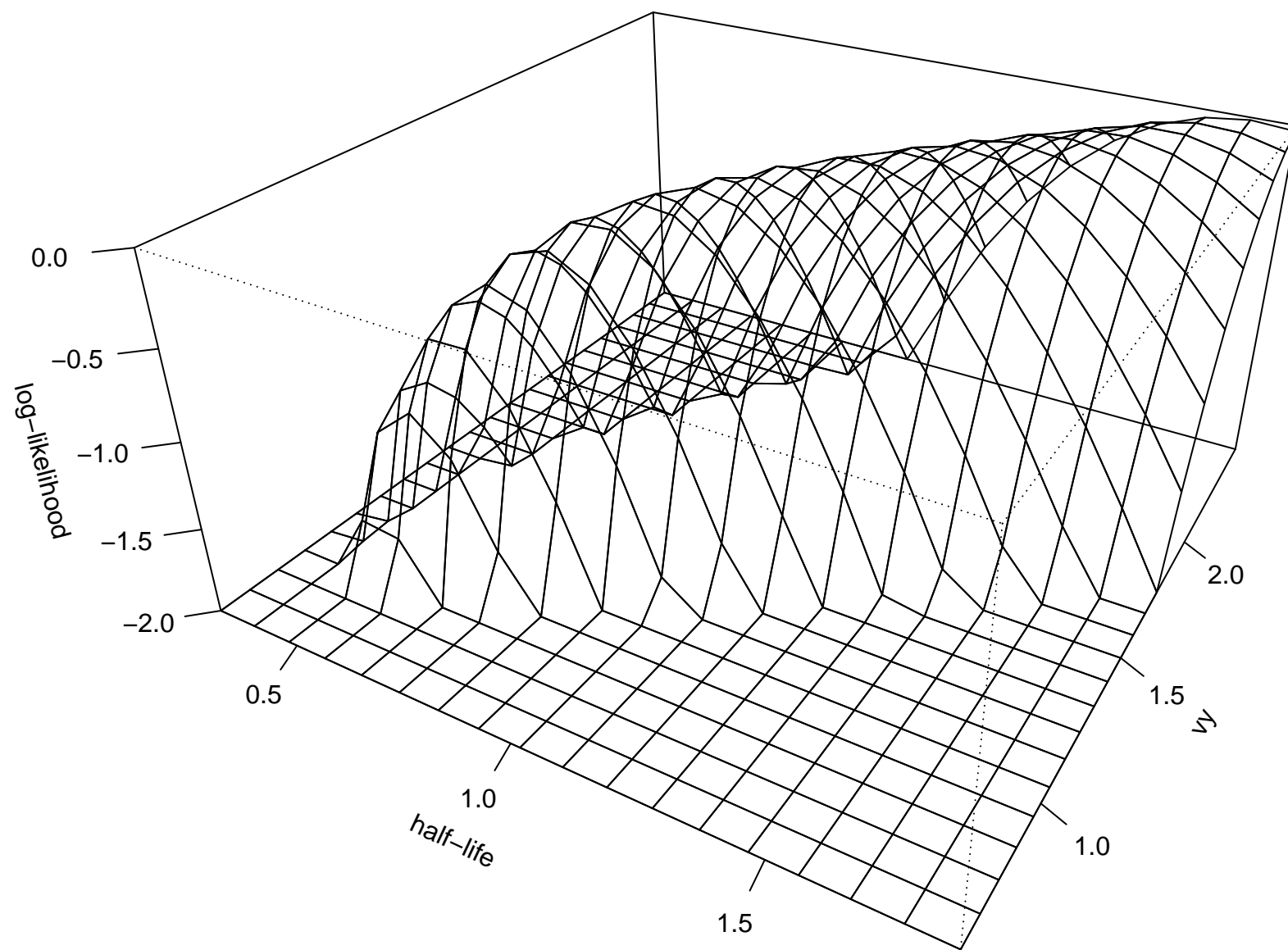

Supplement: Additional file 1: — All phylogenies used in analyses. R script for data extraction and analyses. Detailed results/raw output from SLOUCH. SLOUCH input data. Likelihood plots for all half-life estimations. (ZIP 2442 kb) [file 12862_2016_778_MOESM1_ESM.zip › Additional file 1/Results Bergman's rule - body mass/Dasyuridae_BM_midlat.pdf]

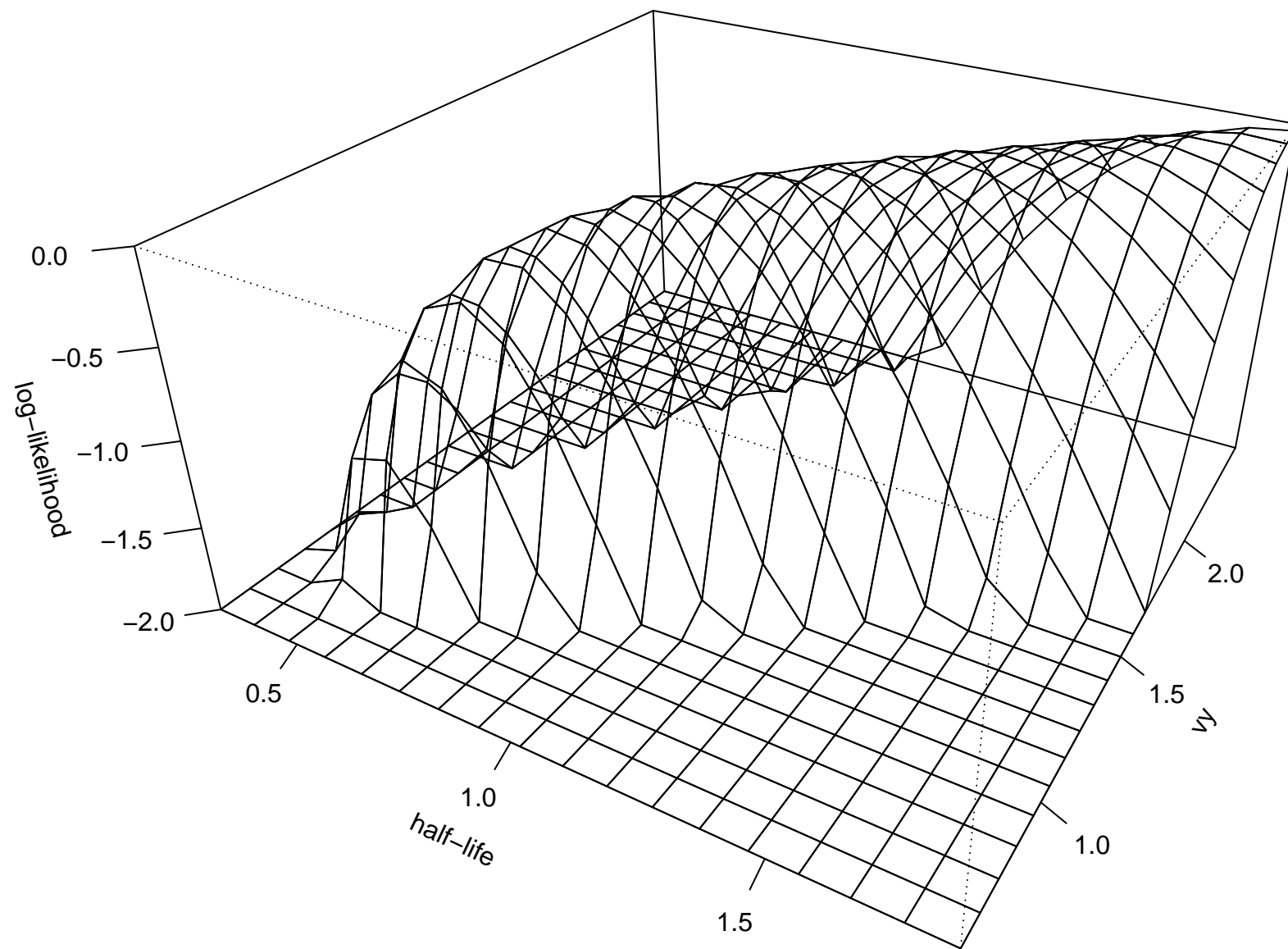

Supplement: Additional file 1: — All phylogenies used in analyses. R script for data extraction and analyses. Detailed results/raw output from SLOUCH. SLOUCH input data. Likelihood plots for all half-life estimations. (ZIP 2442 kb) [file 12862_2016_778_MOESM1_ESM.zip › Additional file 1/Results Bergman's rule - body mass/Dasyuridae_BM_temp.pdf]

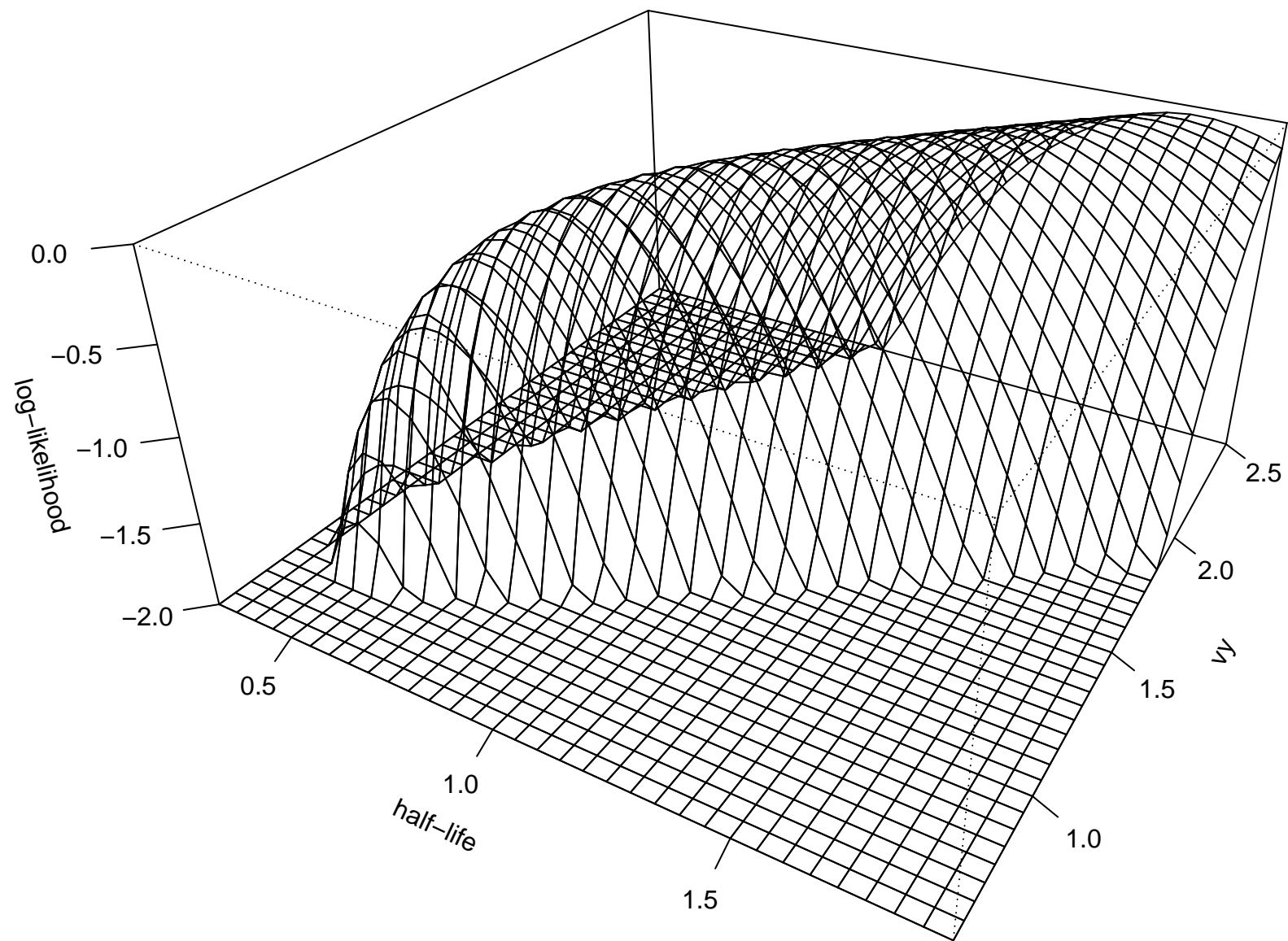

Supplement: Additional file 1: — All phylogenies used in analyses. R script for data extraction and analyses. Detailed results/raw output from SLOUCH. SLOUCH input data. Likelihood plots for all half-life estimations. (ZIP 2442 kb) [file 12862_2016_778_MOESM1_ESM.zip › Additional file 1/Results Bergman's rule - body mass/Dasyuridae_phySig.pdf]

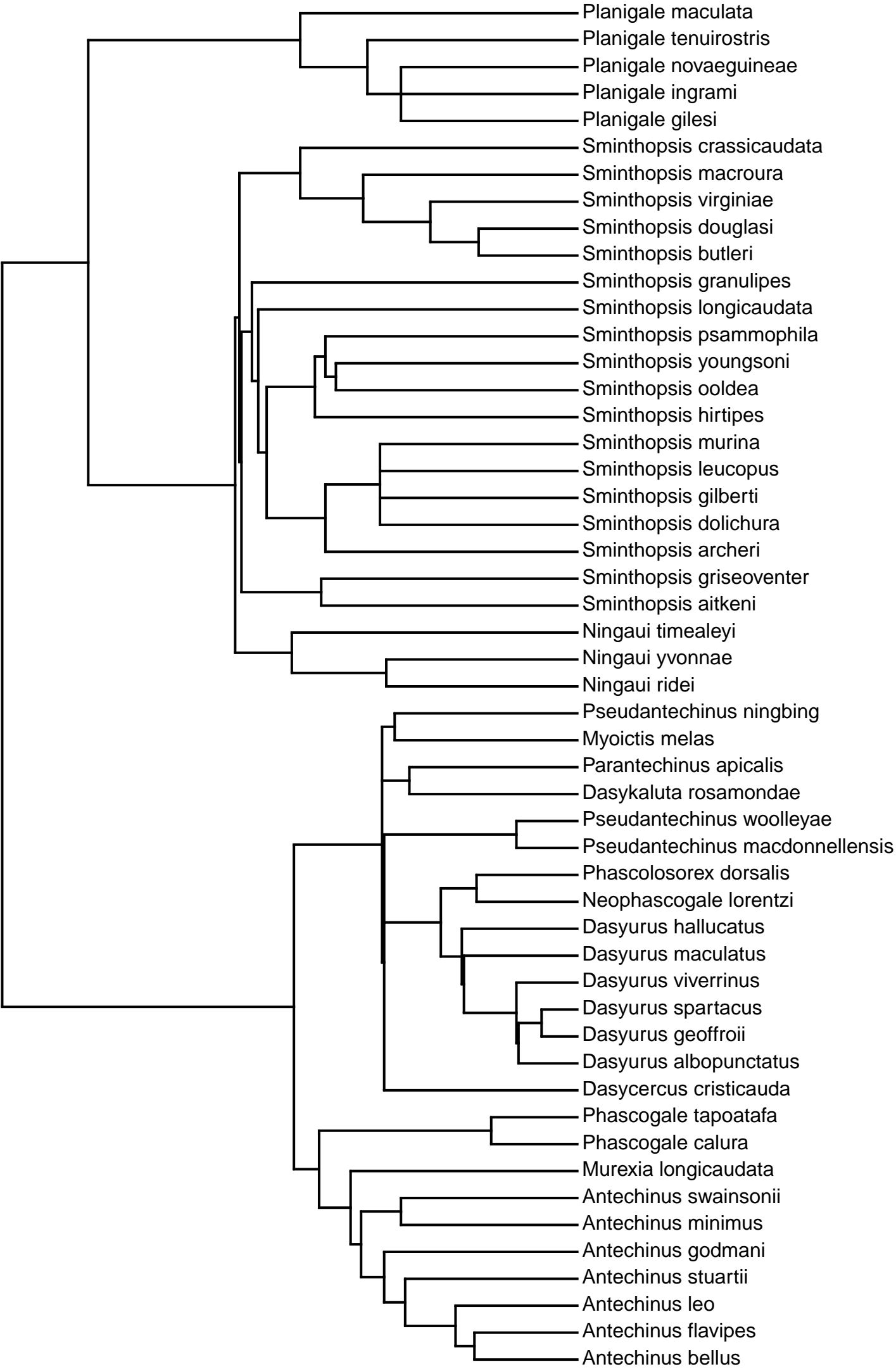

Supplement: Additional file 1: — All phylogenies used in analyses. R script for data extraction and analyses. Detailed results/raw output from SLOUCH. SLOUCH input data. Likelihood plots for all half-life estimations. (ZIP 2442 kb) [file 12862_2016_778_MOESM1_ESM.zip › Additional file 1/Results Bergman's rule - body mass/Dasyuridae_tree.pdf]

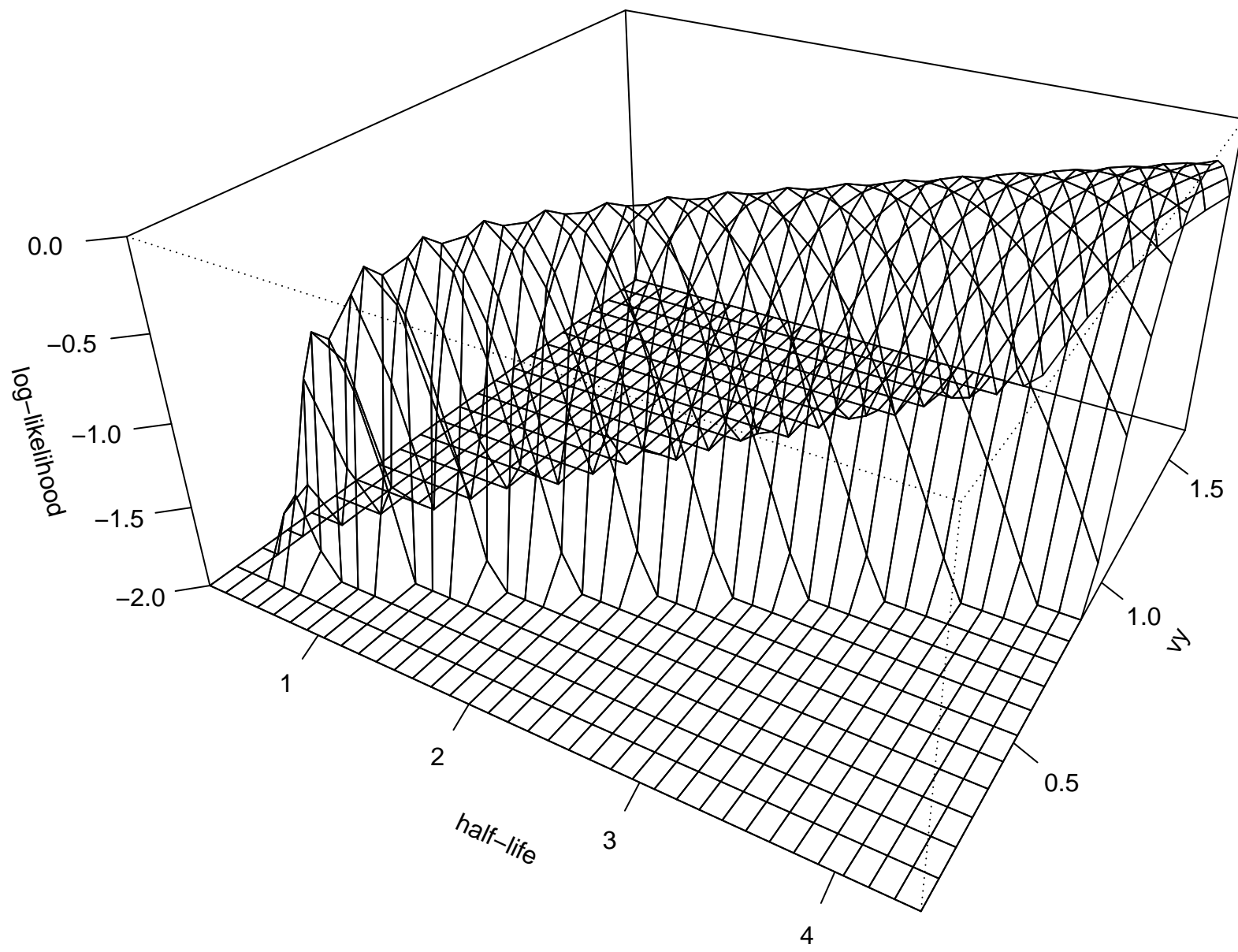

Supplement: Additional file 1: — All phylogenies used in analyses. R script for data extraction and analyses. Detailed results/raw output from SLOUCH. SLOUCH input data. Likelihood plots for all half-life estimations. (ZIP 2442 kb) [file 12862_2016_778_MOESM1_ESM.zip › Additional file 1/Results Bergman's rule - body mass/Echimyidae_BM_maxlat.pdf]

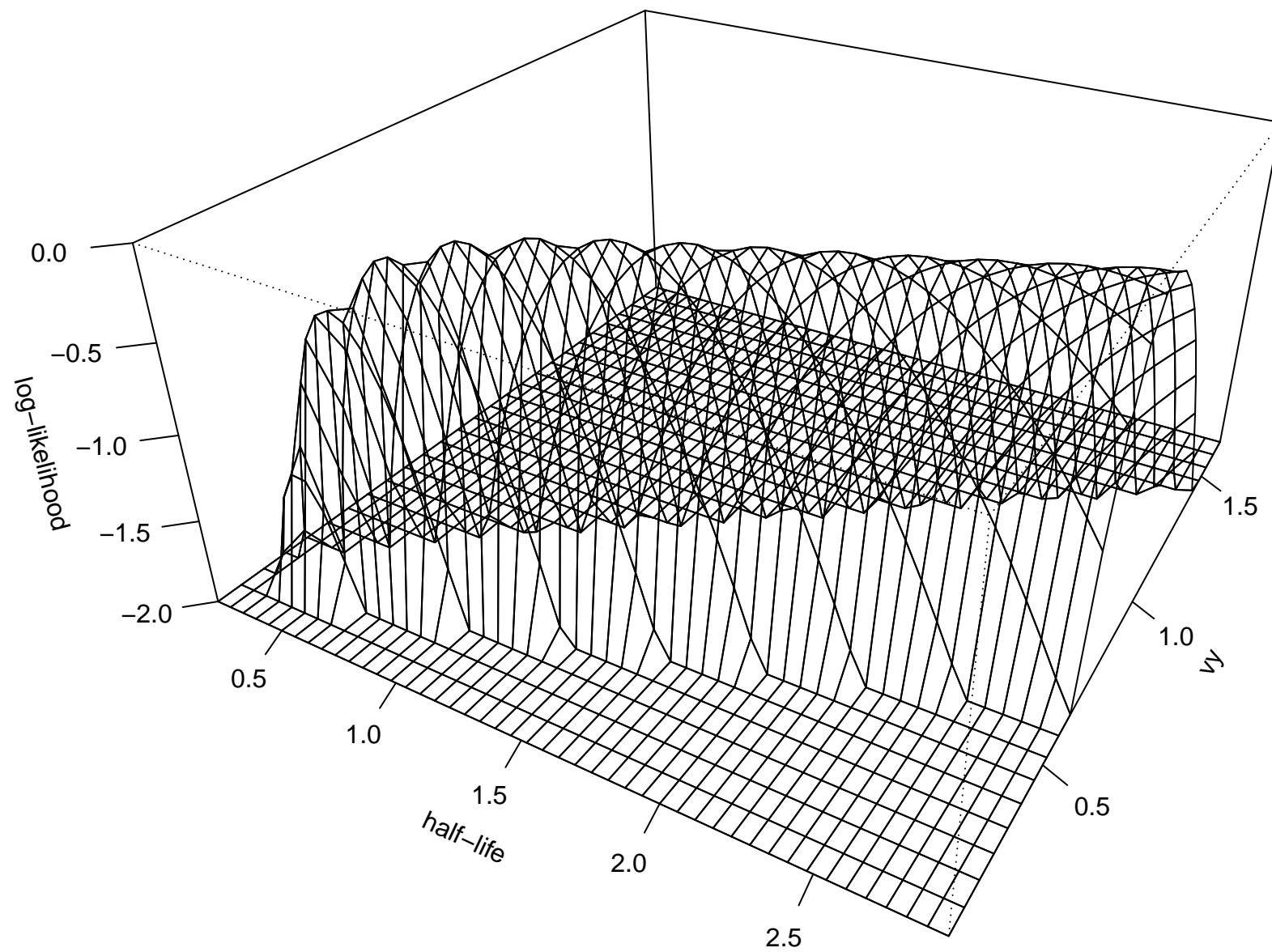

Supplement: Additional file 1: — All phylogenies used in analyses. R script for data extraction and analyses. Detailed results/raw output from SLOUCH. SLOUCH input data. Likelihood plots for all half-life estimations. (ZIP 2442 kb) [file 12862_2016_778_MOESM1_ESM.zip › Additional file 1/Results Bergman's rule - body mass/Echimyidae_BM_midlat.pdf]

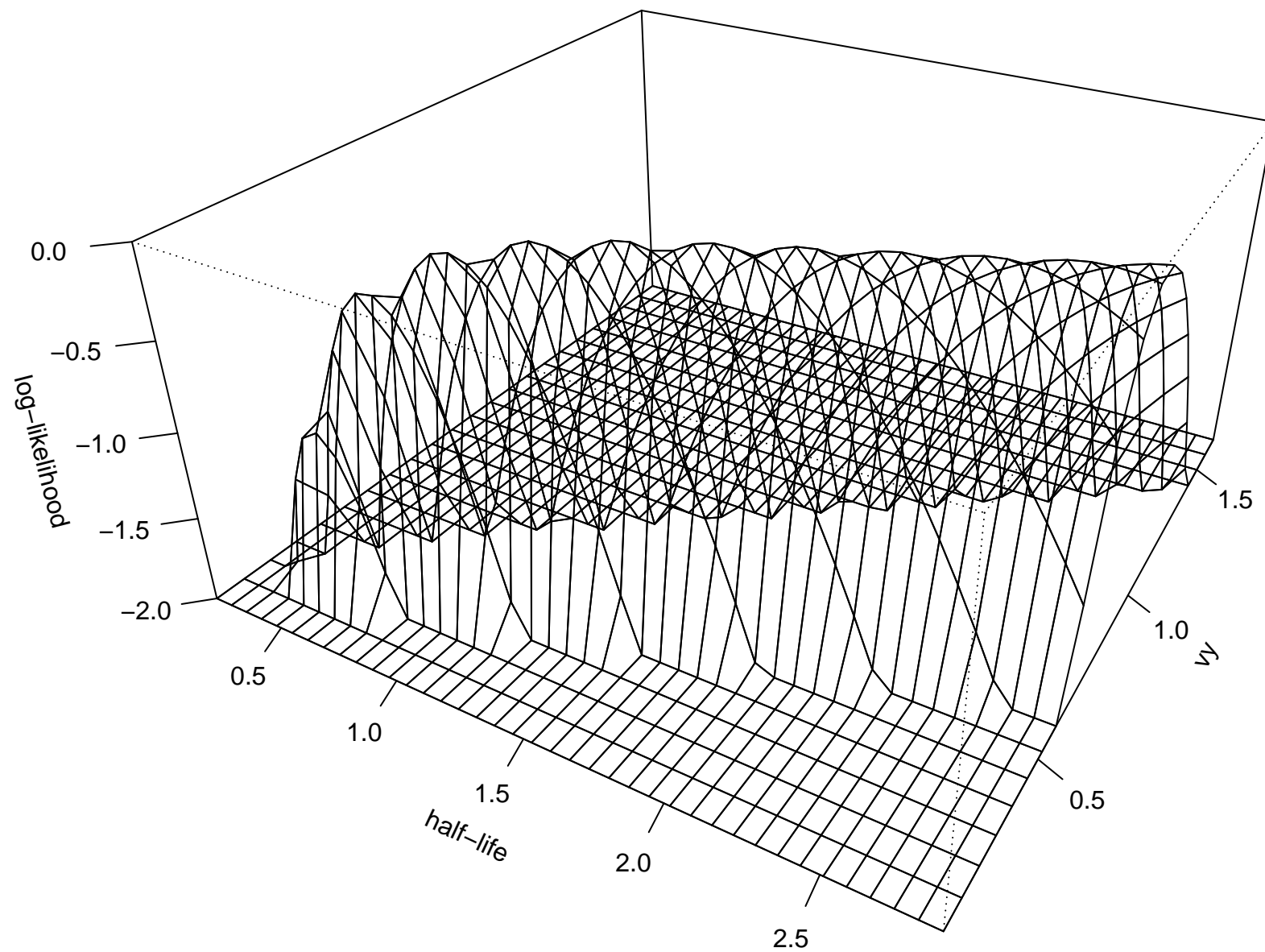

Supplement: Additional file 1: — All phylogenies used in analyses. R script for data extraction and analyses. Detailed results/raw output from SLOUCH. SLOUCH input data. Likelihood plots for all half-life estimations. (ZIP 2442 kb) [file 12862_2016_778_MOESM1_ESM.zip › Additional file 1/Results Bergman's rule - body mass/Echimyidae_BM_temp.pdf]

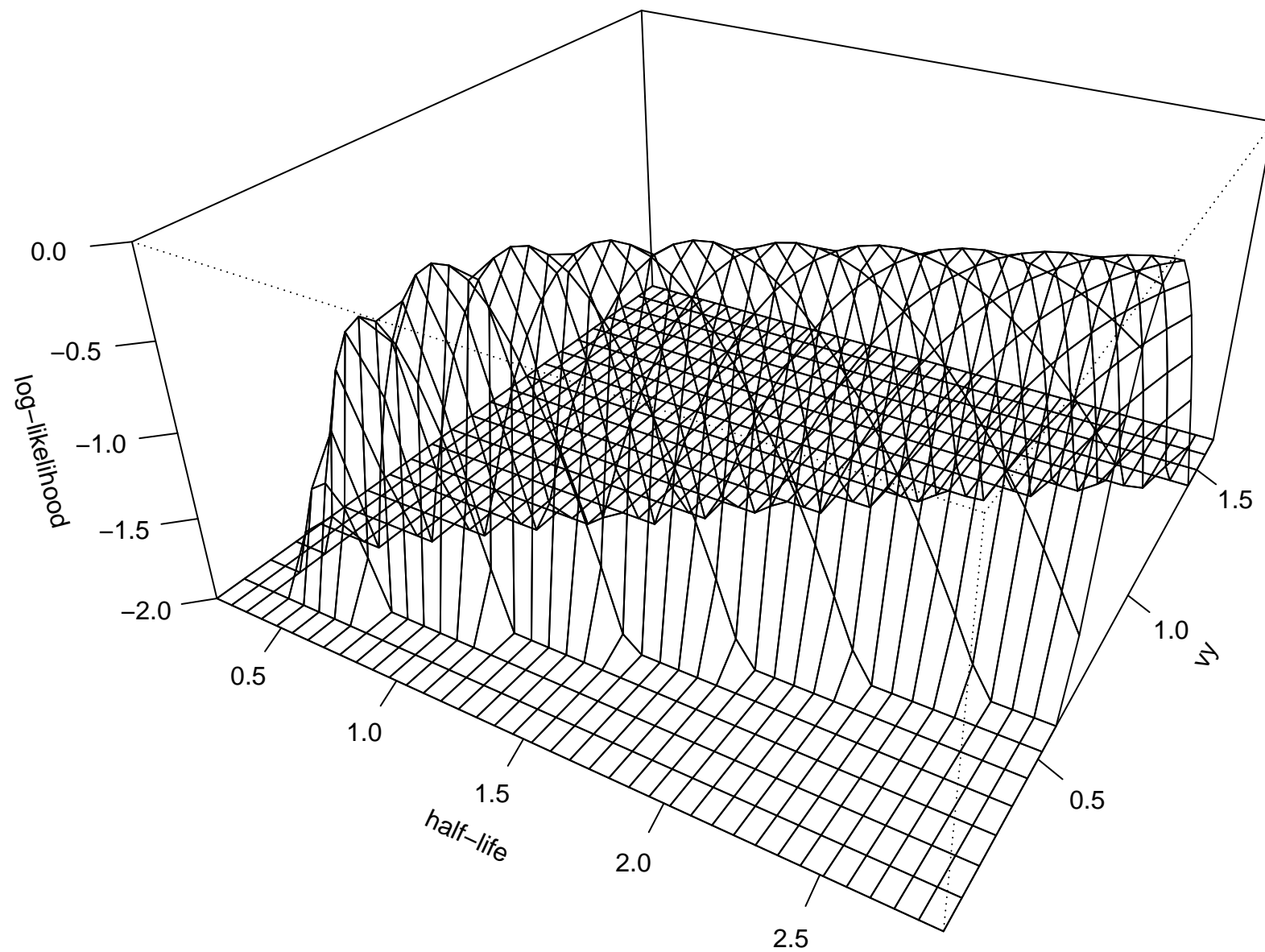

Supplement: Additional file 1: — All phylogenies used in analyses. R script for data extraction and analyses. Detailed results/raw output from SLOUCH. SLOUCH input data. Likelihood plots for all half-life estimations. (ZIP 2442 kb) [file 12862_2016_778_MOESM1_ESM.zip › Additional file 1/Results Bergman's rule - body mass/Echimyidae_phySig.pdf]

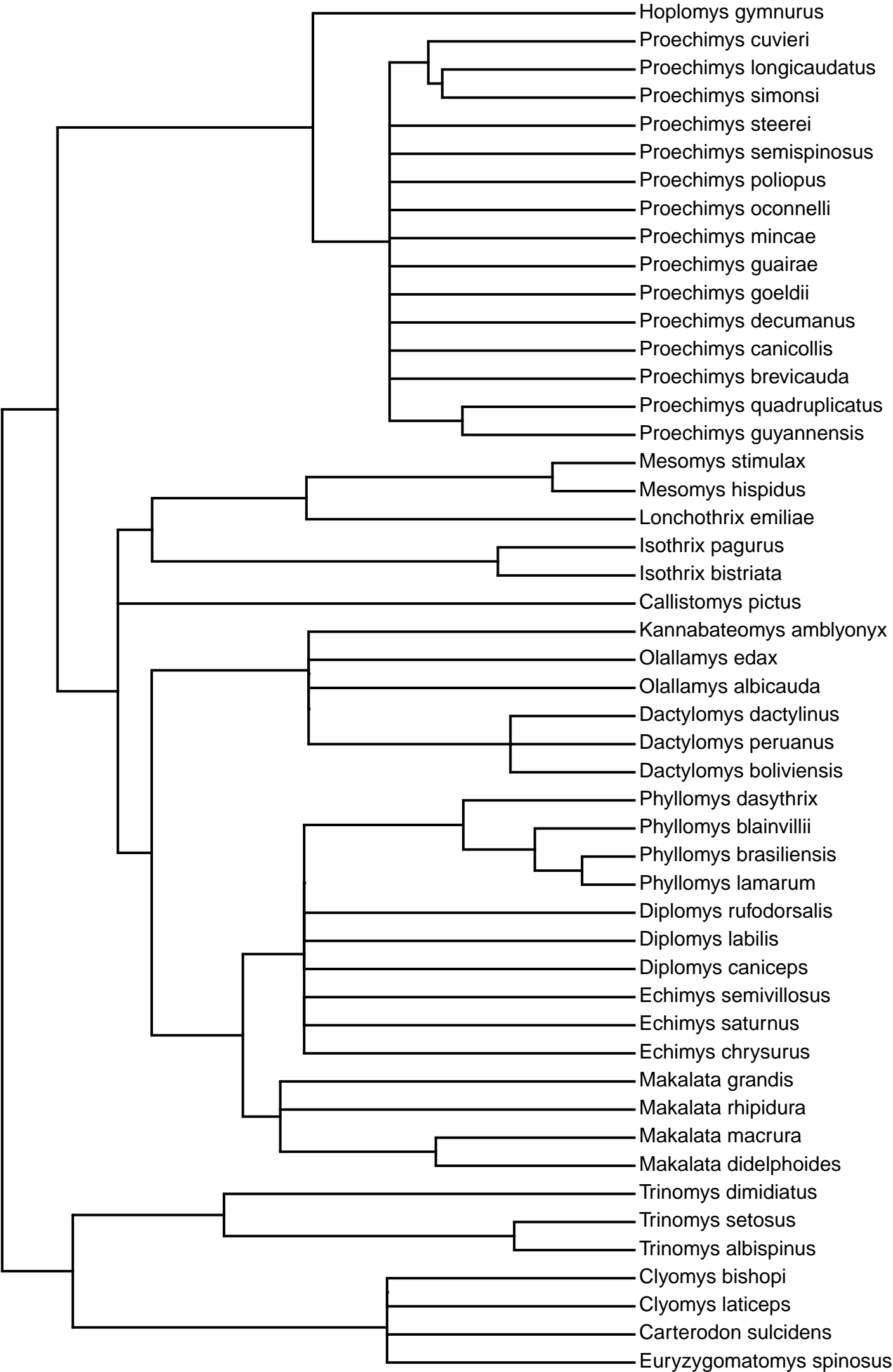

Supplement: Additional file 1: — All phylogenies used in analyses. R script for data extraction and analyses. Detailed results/raw output from SLOUCH. SLOUCH input data. Likelihood plots for all half-life estimations. (ZIP 2442 kb) [file 12862_2016_778_MOESM1_ESM.zip › Additional file 1/Results Bergman's rule - body mass/Echimyidae_tree.pdf]

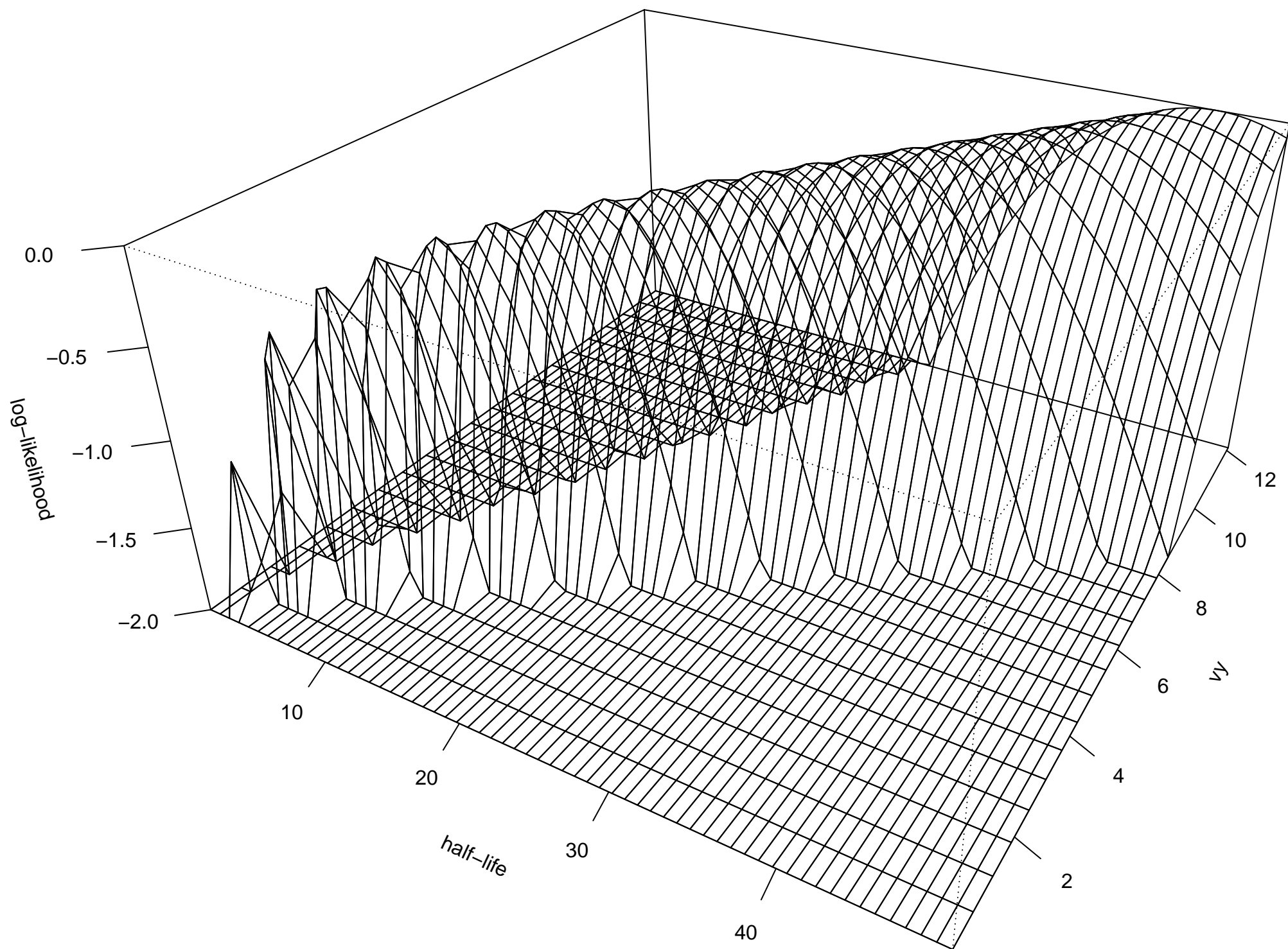

Supplement: Additional file 1: — All phylogenies used in analyses. R script for data extraction and analyses. Detailed results/raw output from SLOUCH. SLOUCH input data. Likelihood plots for all half-life estimations. (ZIP 2442 kb) [file 12862_2016_778_MOESM1_ESM.zip › Additional file 1/Results Bergman's rule - body mass/Emballonuridae_BM_maxlat.pdf]

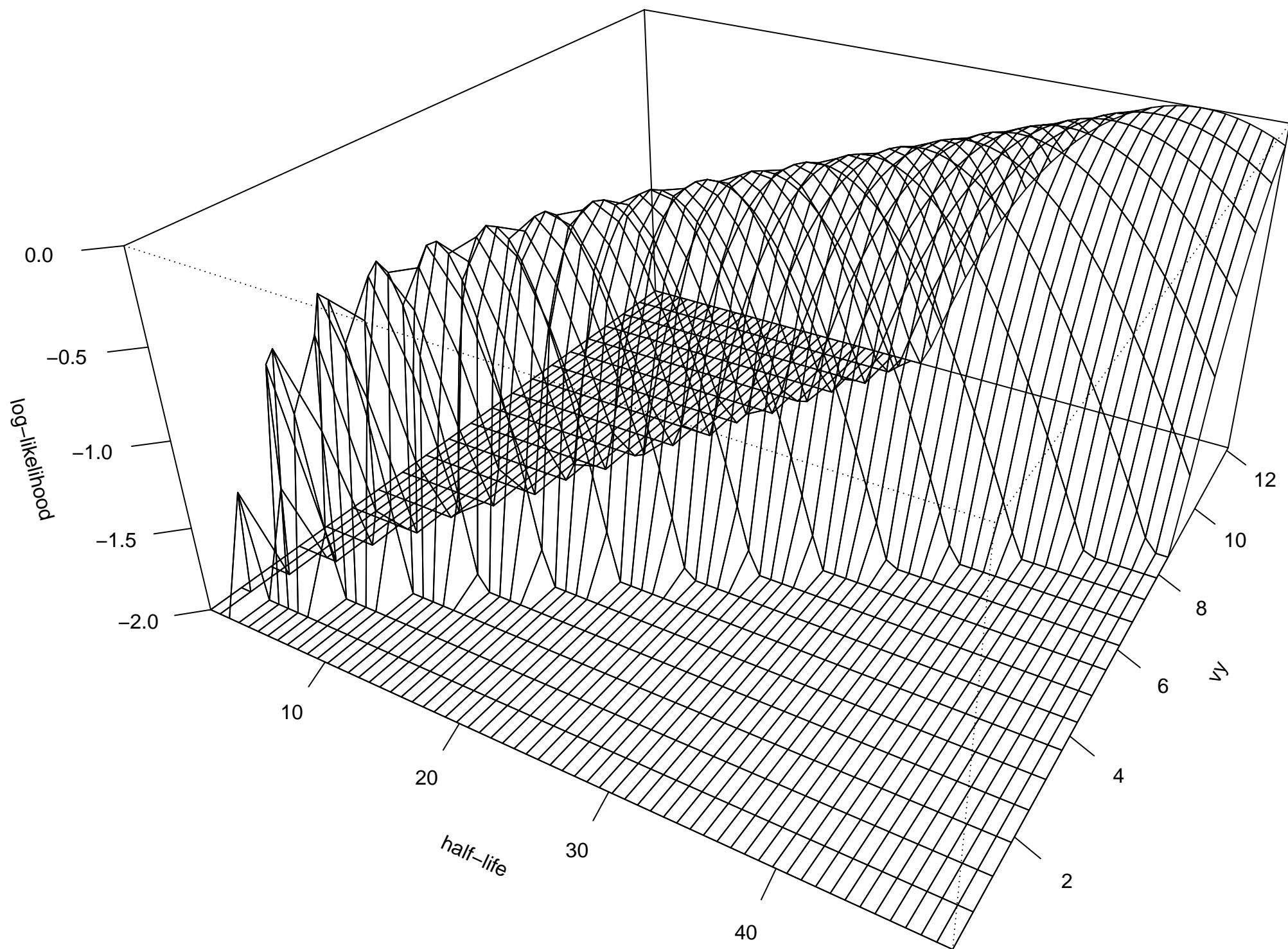

Supplement: Additional file 1: — All phylogenies used in analyses. R script for data extraction and analyses. Detailed results/raw output from SLOUCH. SLOUCH input data. Likelihood plots for all half-life estimations. (ZIP 2442 kb) [file 12862_2016_778_MOESM1_ESM.zip › Additional file 1/Results Bergman's rule - body mass/Emballonuridae_BM_midlat.pdf]

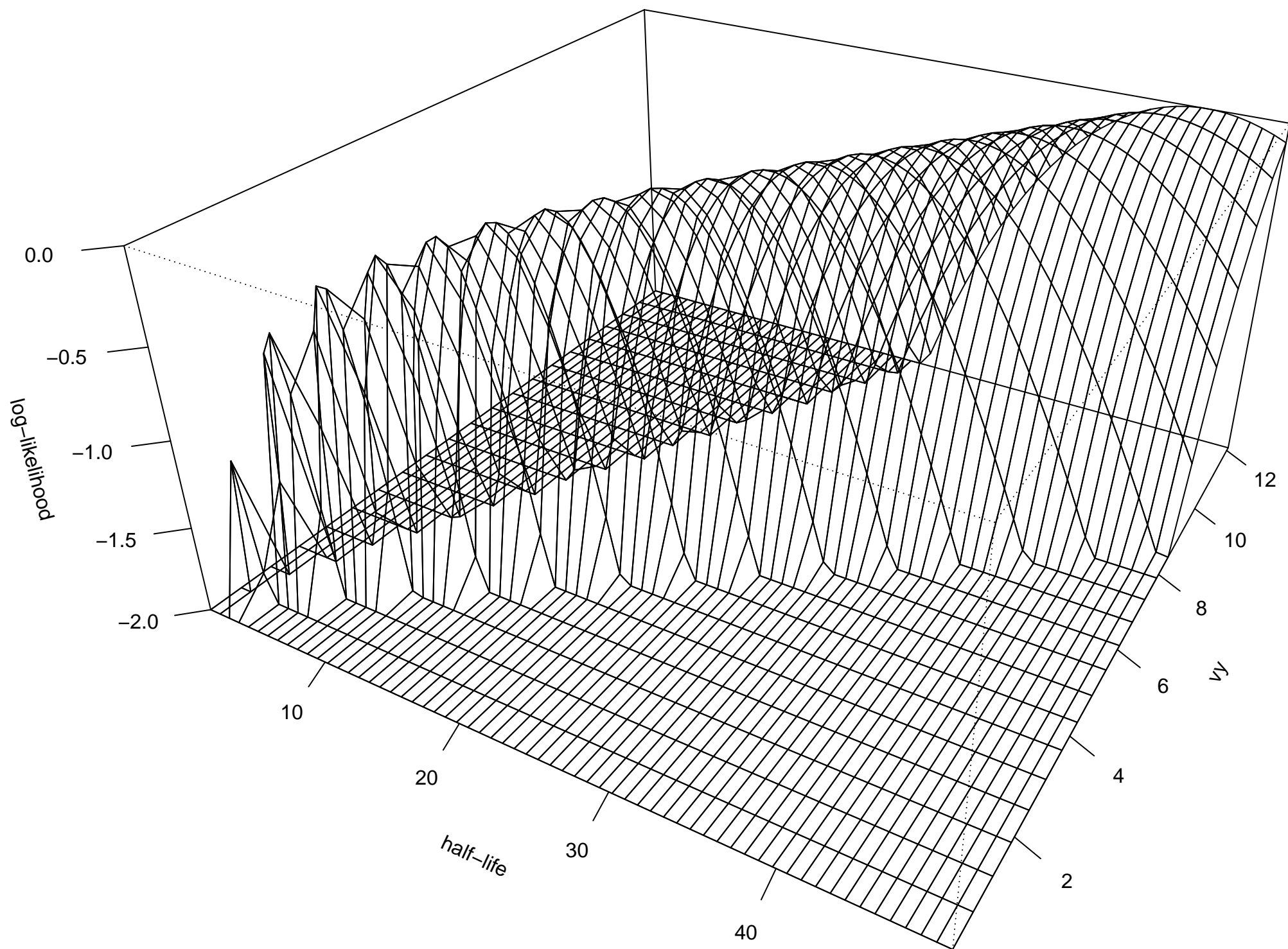

Supplement: Additional file 1: — All phylogenies used in analyses. R script for data extraction and analyses. Detailed results/raw output from SLOUCH. SLOUCH input data. Likelihood plots for all half-life estimations. (ZIP 2442 kb) [file 12862_2016_778_MOESM1_ESM.zip › Additional file 1/Results Bergman's rule - body mass/Emballonuridae_BM_temp.pdf]

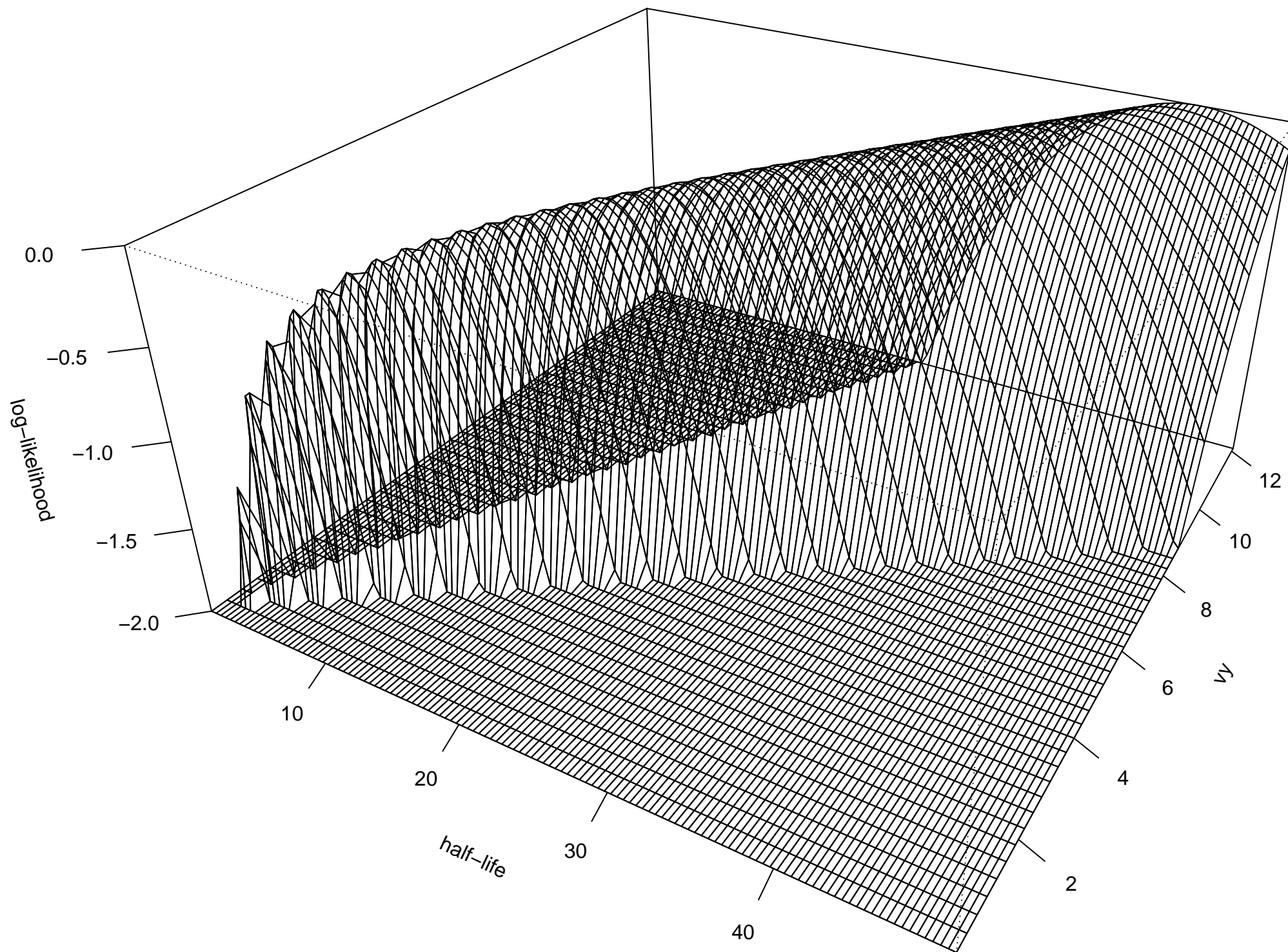

Supplement: Additional file 1: — All phylogenies used in analyses. R script for data extraction and analyses. Detailed results/raw output from SLOUCH. SLOUCH input data. Likelihood plots for all half-life estimations. (ZIP 2442 kb) [file 12862_2016_778_MOESM1_ESM.zip › Additional file 1/Results Bergman's rule - body mass/Emballonuridae_phySig.pdf]

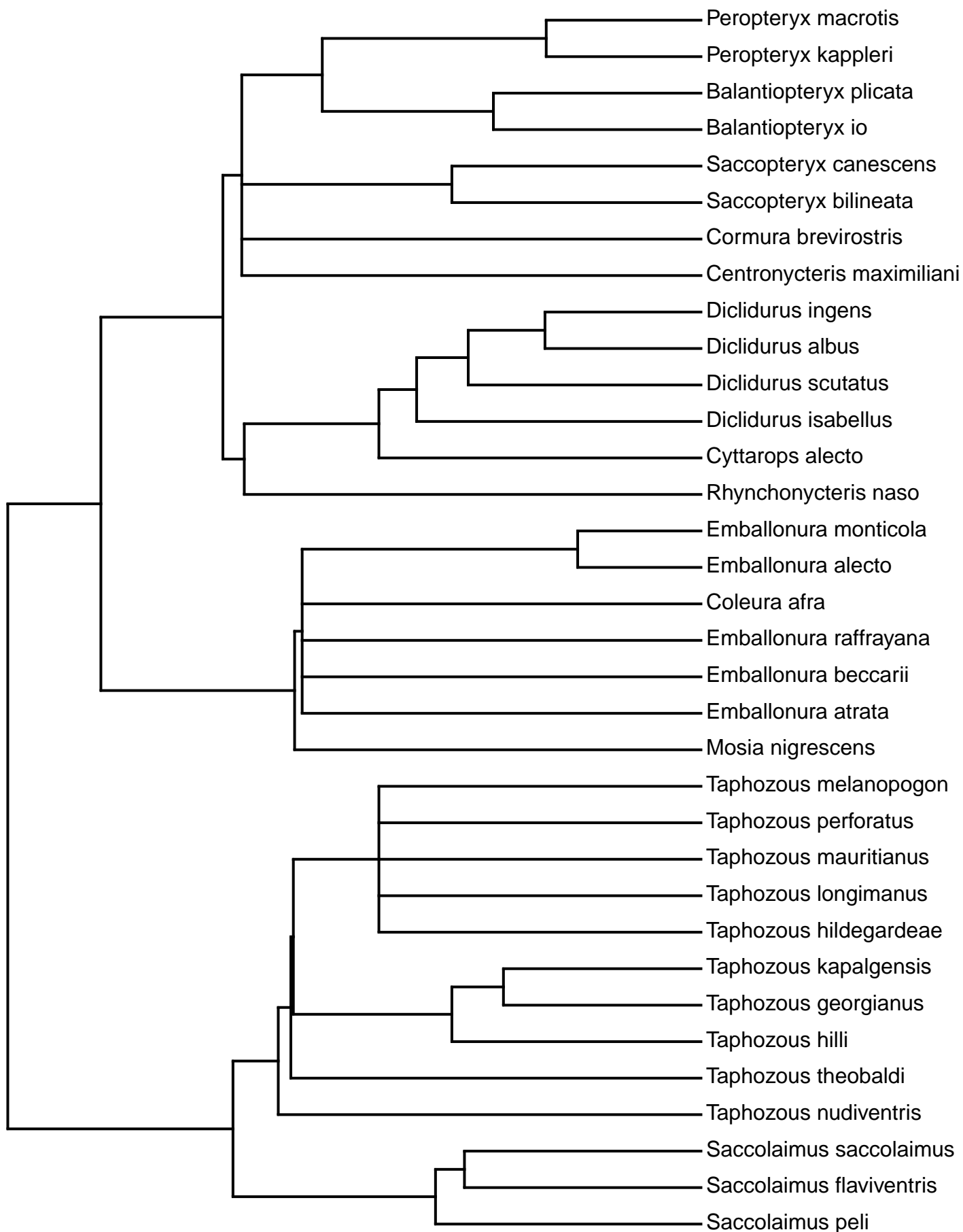

Supplement: Additional file 1: — All phylogenies used in analyses. R script for data extraction and analyses. Detailed results/raw output from SLOUCH. SLOUCH input data. Likelihood plots for all half-life estimations. (ZIP 2442 kb) [file 12862_2016_778_MOESM1_ESM.zip › Additional file 1/Results Bergman's rule - body mass/Emballonuridae_tree.pdf]

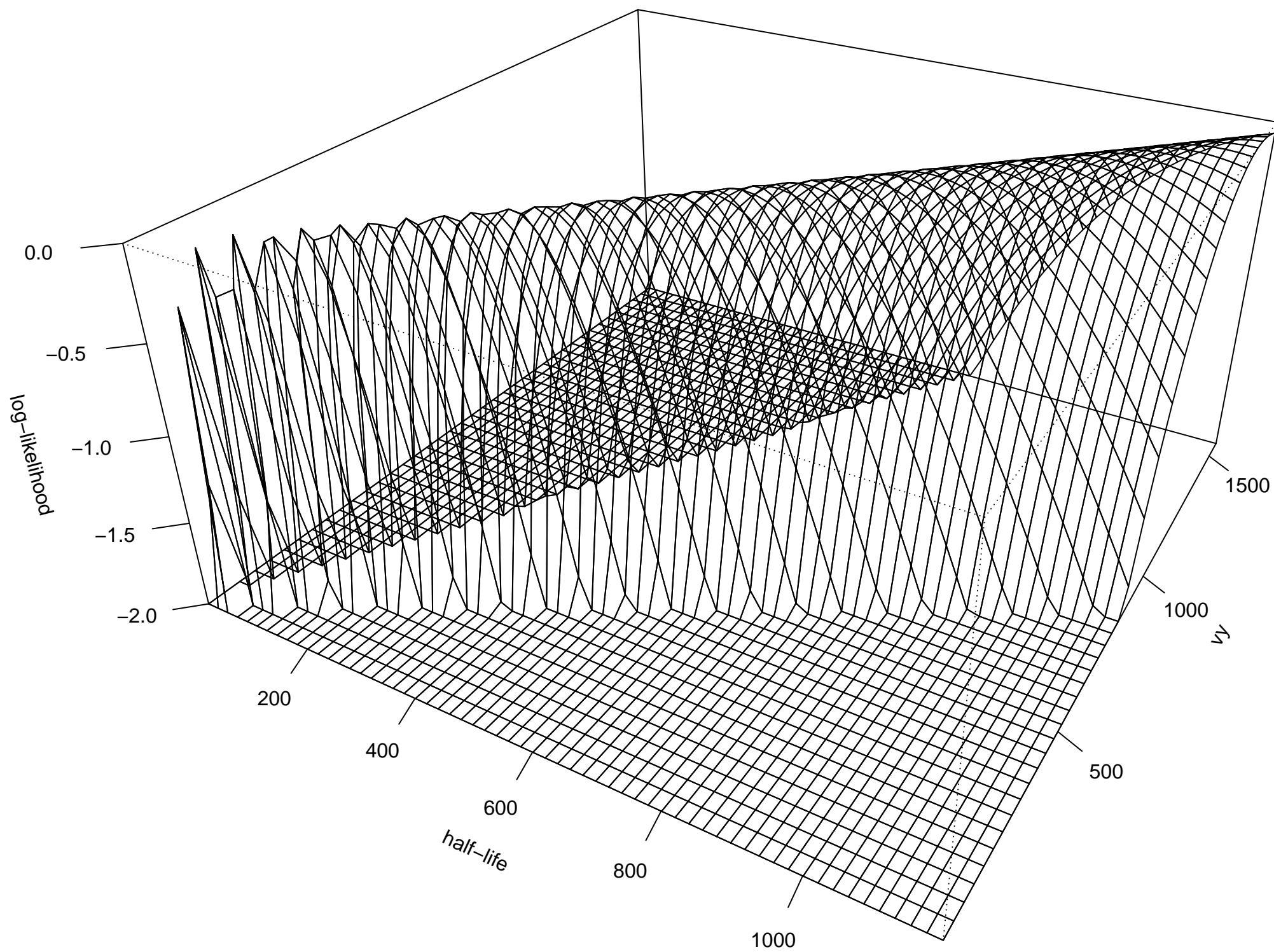

Supplement: Additional file 1: — All phylogenies used in analyses. R script for data extraction and analyses. Detailed results/raw output from SLOUCH. SLOUCH input data. Likelihood plots for all half-life estimations. (ZIP 2442 kb) [file 12862_2016_778_MOESM1_ESM.zip › Additional file 1/Results Bergman's rule - body mass/Felidae_BM_maxlat.pdf]

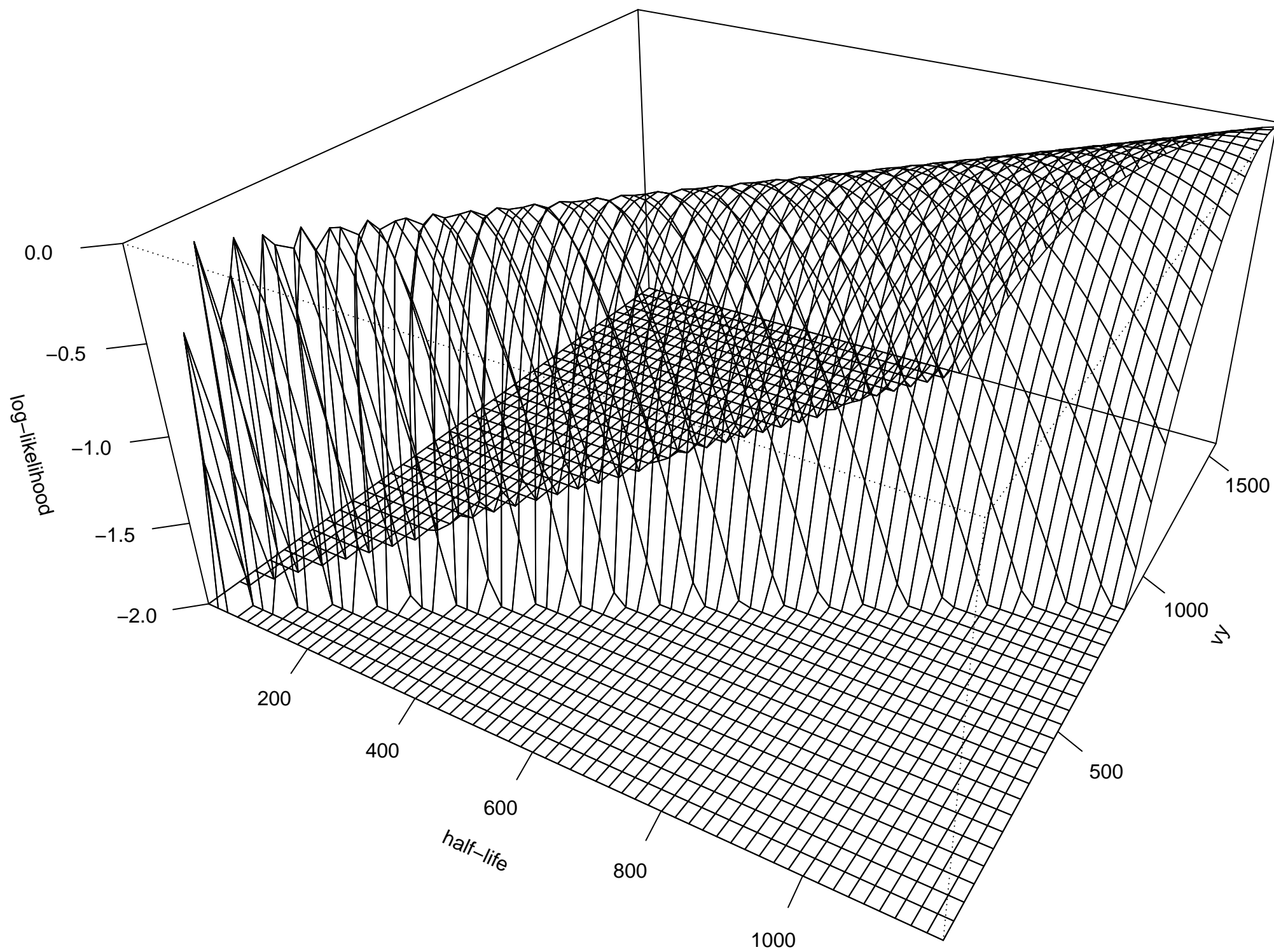

Supplement: Additional file 1: — All phylogenies used in analyses. R script for data extraction and analyses. Detailed results/raw output from SLOUCH. SLOUCH input data. Likelihood plots for all half-life estimations. (ZIP 2442 kb) [file 12862_2016_778_MOESM1_ESM.zip › Additional file 1/Results Bergman's rule - body mass/Felidae_BM_midlat.pdf]

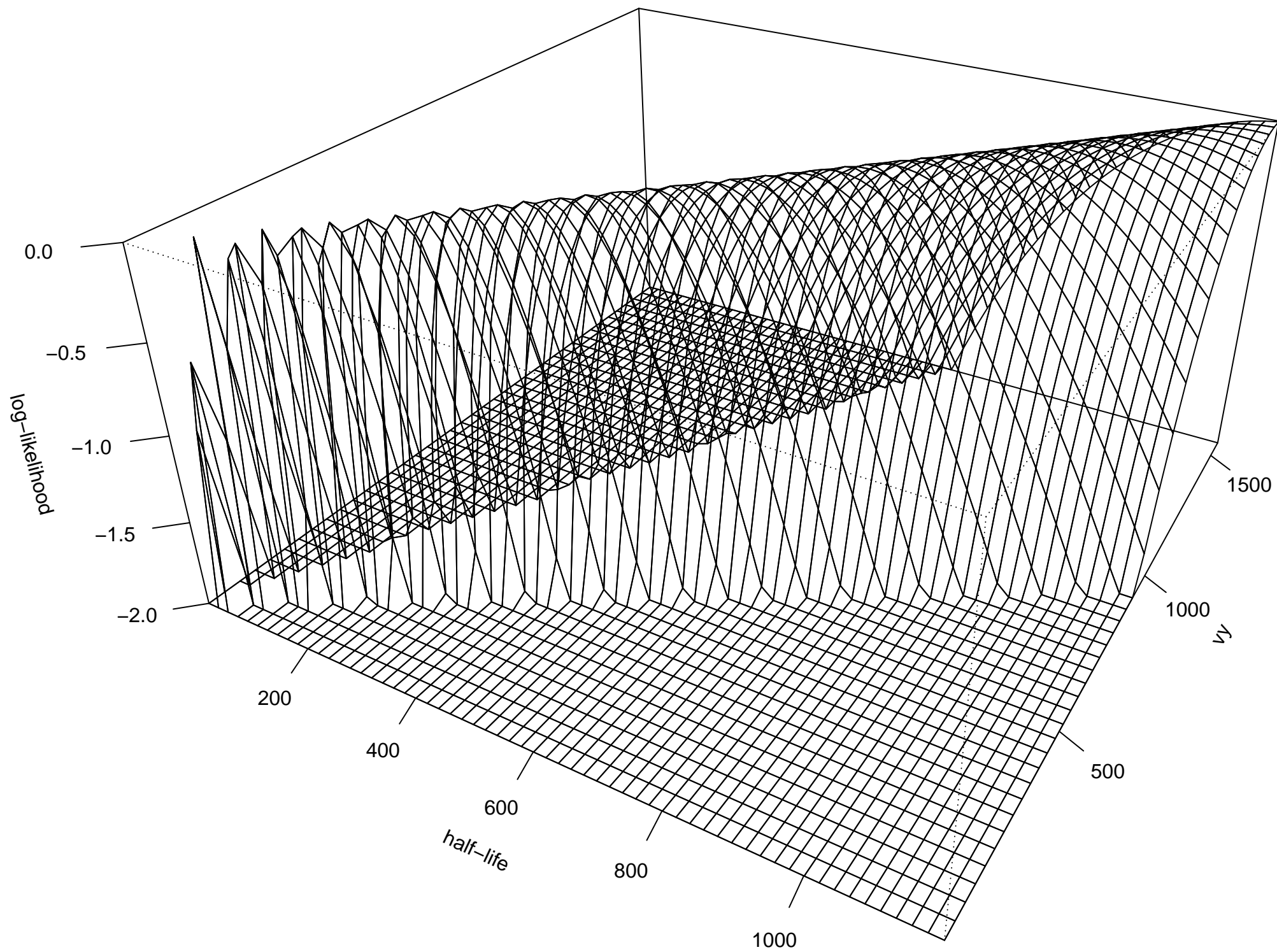

Supplement: Additional file 1: — All phylogenies used in analyses. R script for data extraction and analyses. Detailed results/raw output from SLOUCH. SLOUCH input data. Likelihood plots for all half-life estimations. (ZIP 2442 kb) [file 12862_2016_778_MOESM1_ESM.zip › Additional file 1/Results Bergman's rule - body mass/Felidae_BM_temp.pdf]

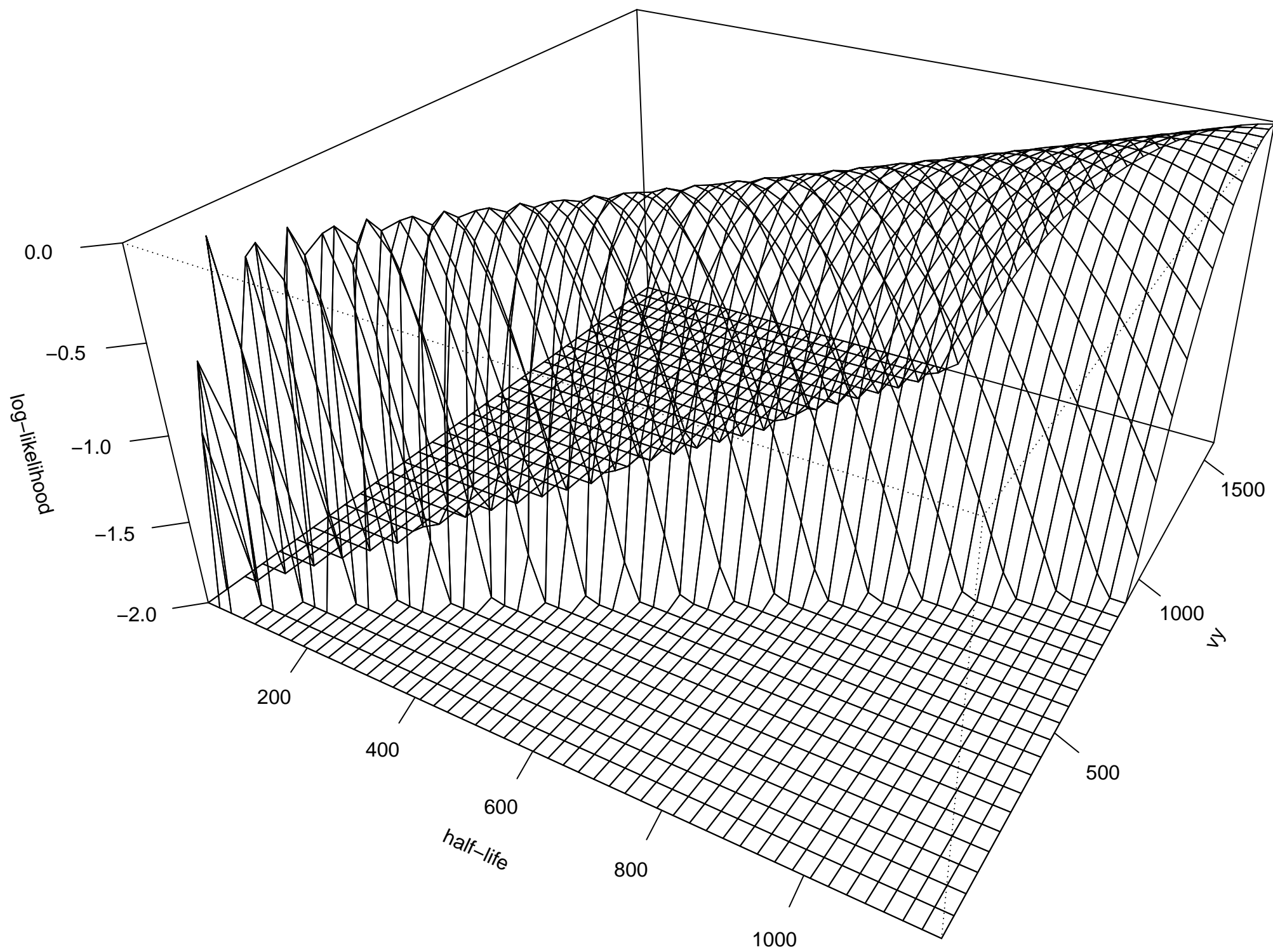

Supplement: Additional file 1: — All phylogenies used in analyses. R script for data extraction and analyses. Detailed results/raw output from SLOUCH. SLOUCH input data. Likelihood plots for all half-life estimations. (ZIP 2442 kb) [file 12862_2016_778_MOESM1_ESM.zip › Additional file 1/Results Bergman's rule - body mass/Felidae_phySig.pdf]

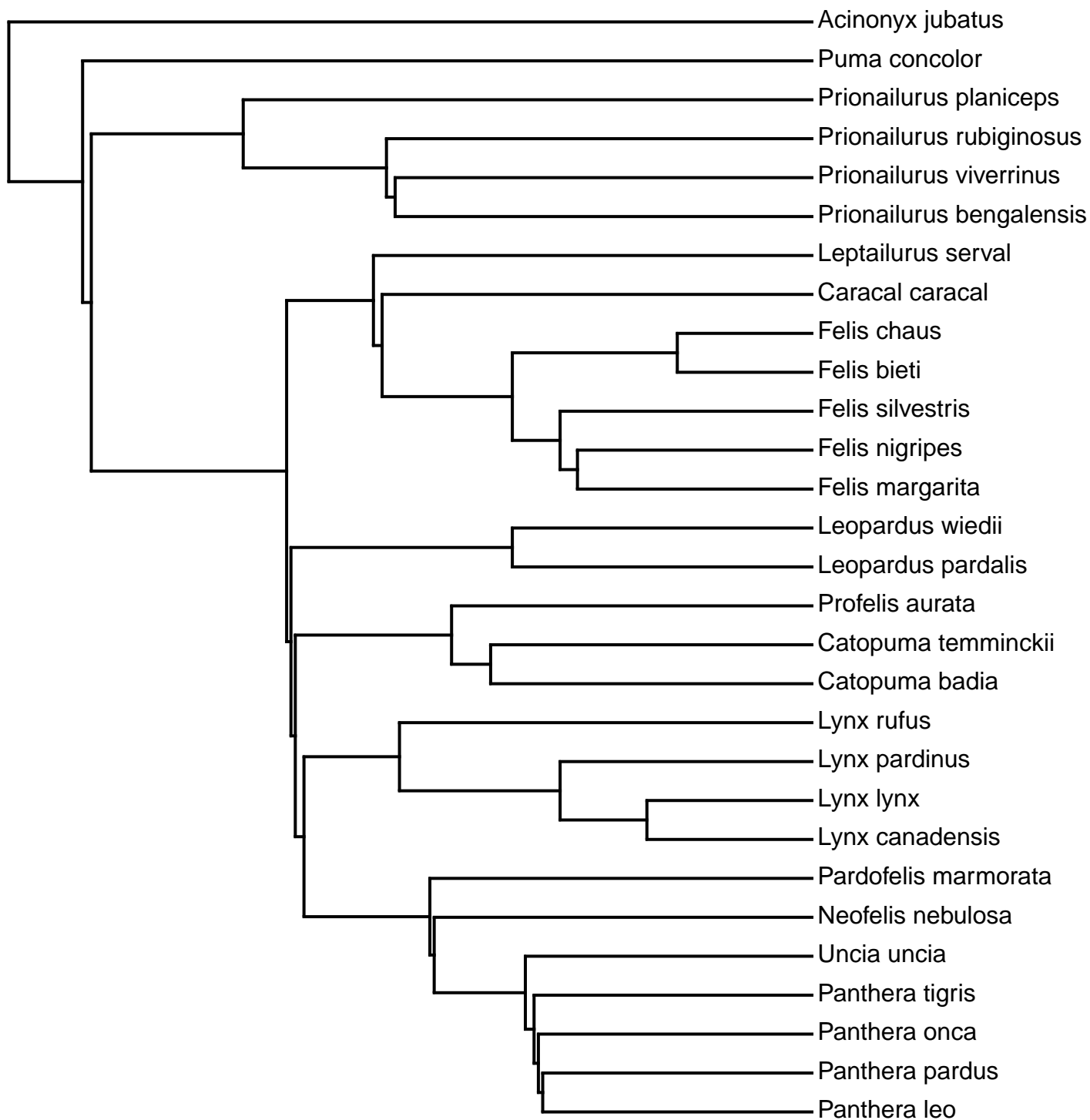

Supplement: Additional file 1: — All phylogenies used in analyses. R script for data extraction and analyses. Detailed results/raw output from SLOUCH. SLOUCH input data. Likelihood plots for all half-life estimations. (ZIP 2442 kb) [file 12862_2016_778_MOESM1_ESM.zip › Additional file 1/Results Bergman's rule - body mass/Felidae_tree.pdf]

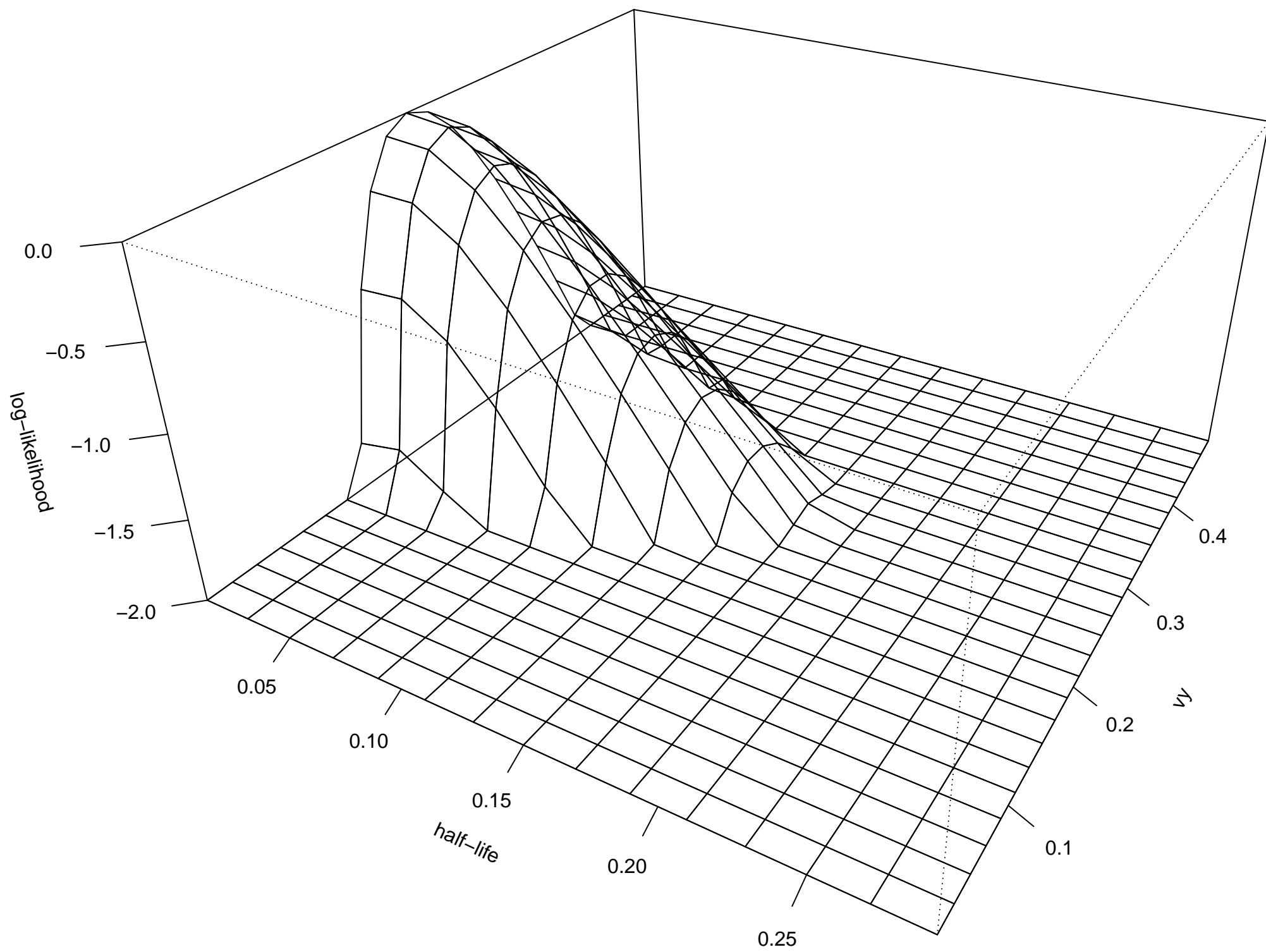

Supplement: Additional file 1: — All phylogenies used in analyses. R script for data extraction and analyses. Detailed results/raw output from SLOUCH. SLOUCH input data. Likelihood plots for all half-life estimations. (ZIP 2442 kb) [file 12862_2016_778_MOESM1_ESM.zip › Additional file 1/Results Bergman's rule - body mass/Geomyidae_BM_maxlat.pdf]

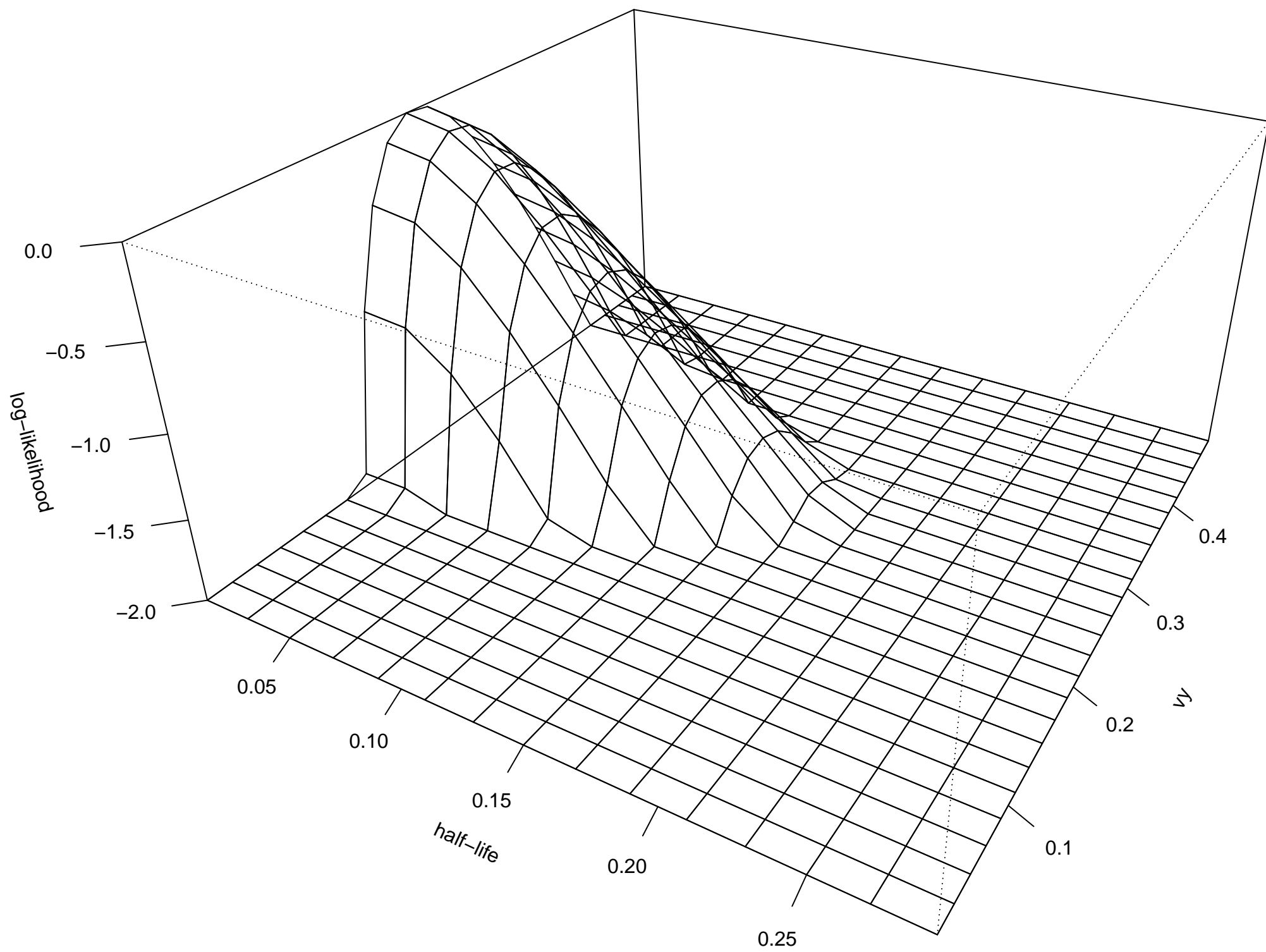

Supplement: Additional file 1: — All phylogenies used in analyses. R script for data extraction and analyses. Detailed results/raw output from SLOUCH. SLOUCH input data. Likelihood plots for all half-life estimations. (ZIP 2442 kb) [file 12862_2016_778_MOESM1_ESM.zip › Additional file 1/Results Bergman's rule - body mass/Geomyidae_BM_midlat.pdf]

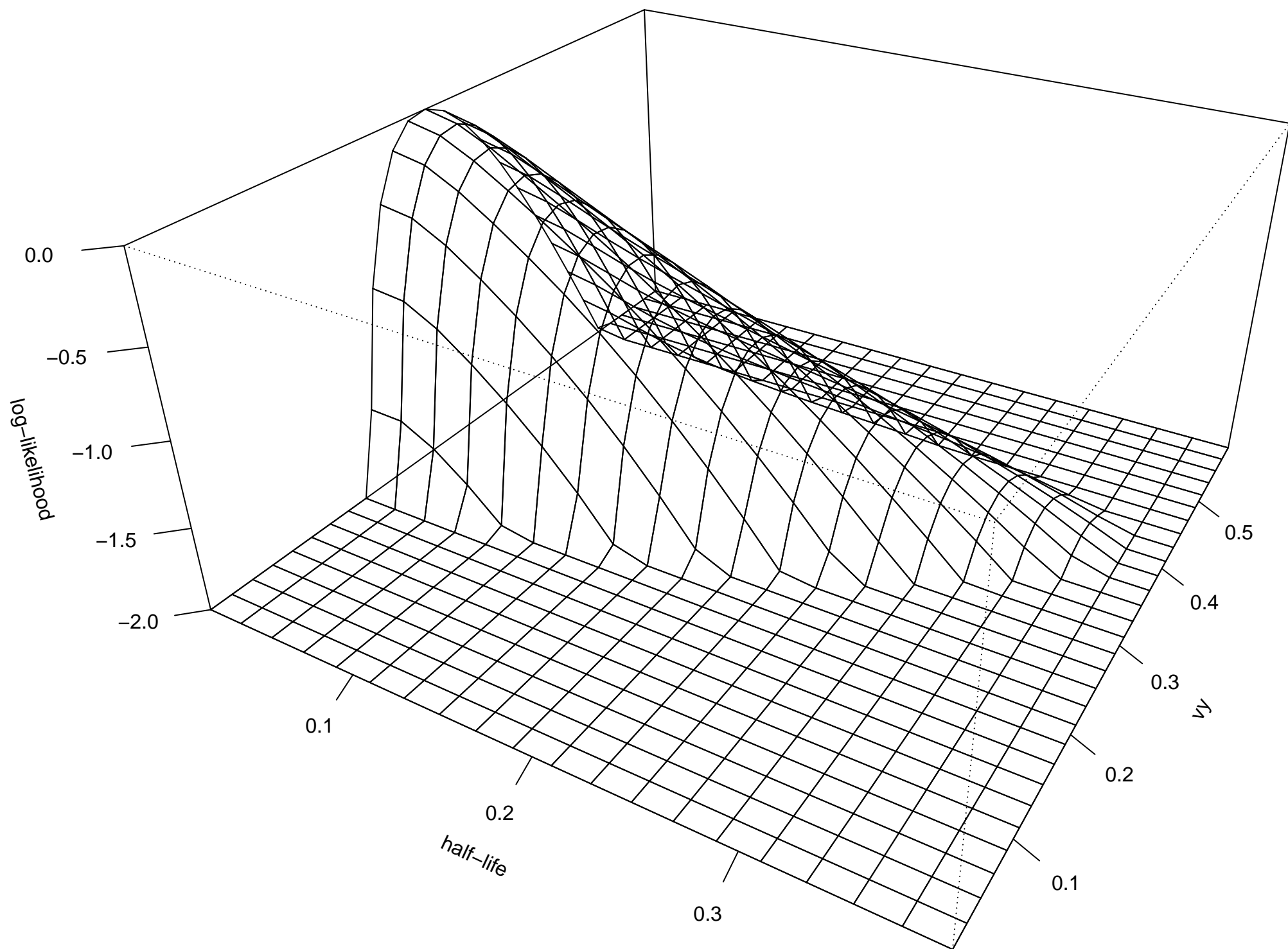

Supplement: Additional file 1: — All phylogenies used in analyses. R script for data extraction and analyses. Detailed results/raw output from SLOUCH. SLOUCH input data. Likelihood plots for all half-life estimations. (ZIP 2442 kb) [file 12862_2016_778_MOESM1_ESM.zip › Additional file 1/Results Bergman's rule - body mass/Geomyidae_BM_temp.pdf]

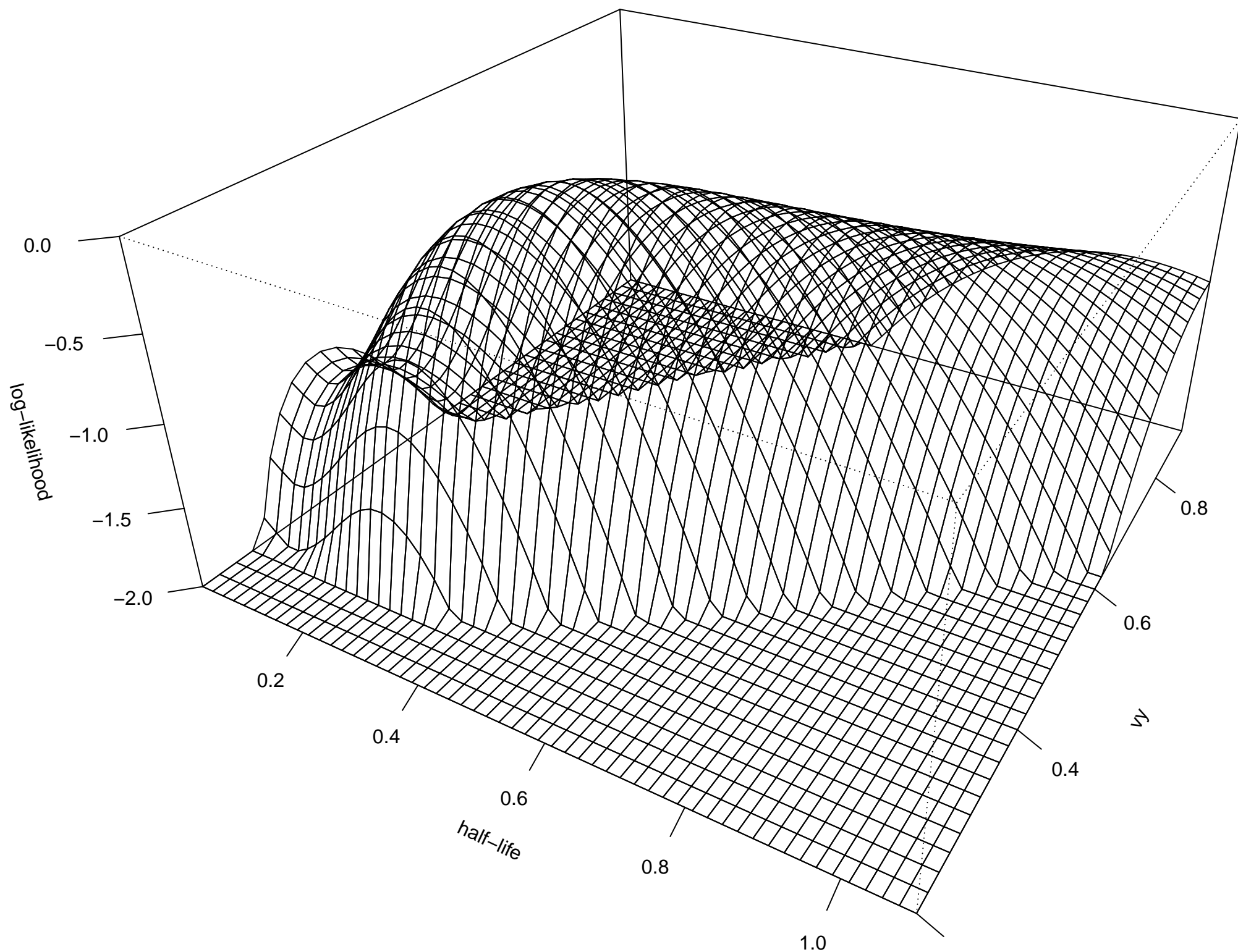

Supplement: Additional file 1: — All phylogenies used in analyses. R script for data extraction and analyses. Detailed results/raw output from SLOUCH. SLOUCH input data. Likelihood plots for all half-life estimations. (ZIP 2442 kb) [file 12862_2016_778_MOESM1_ESM.zip › Additional file 1/Results Bergman's rule - body mass/Geomyidae_phySig.pdf]

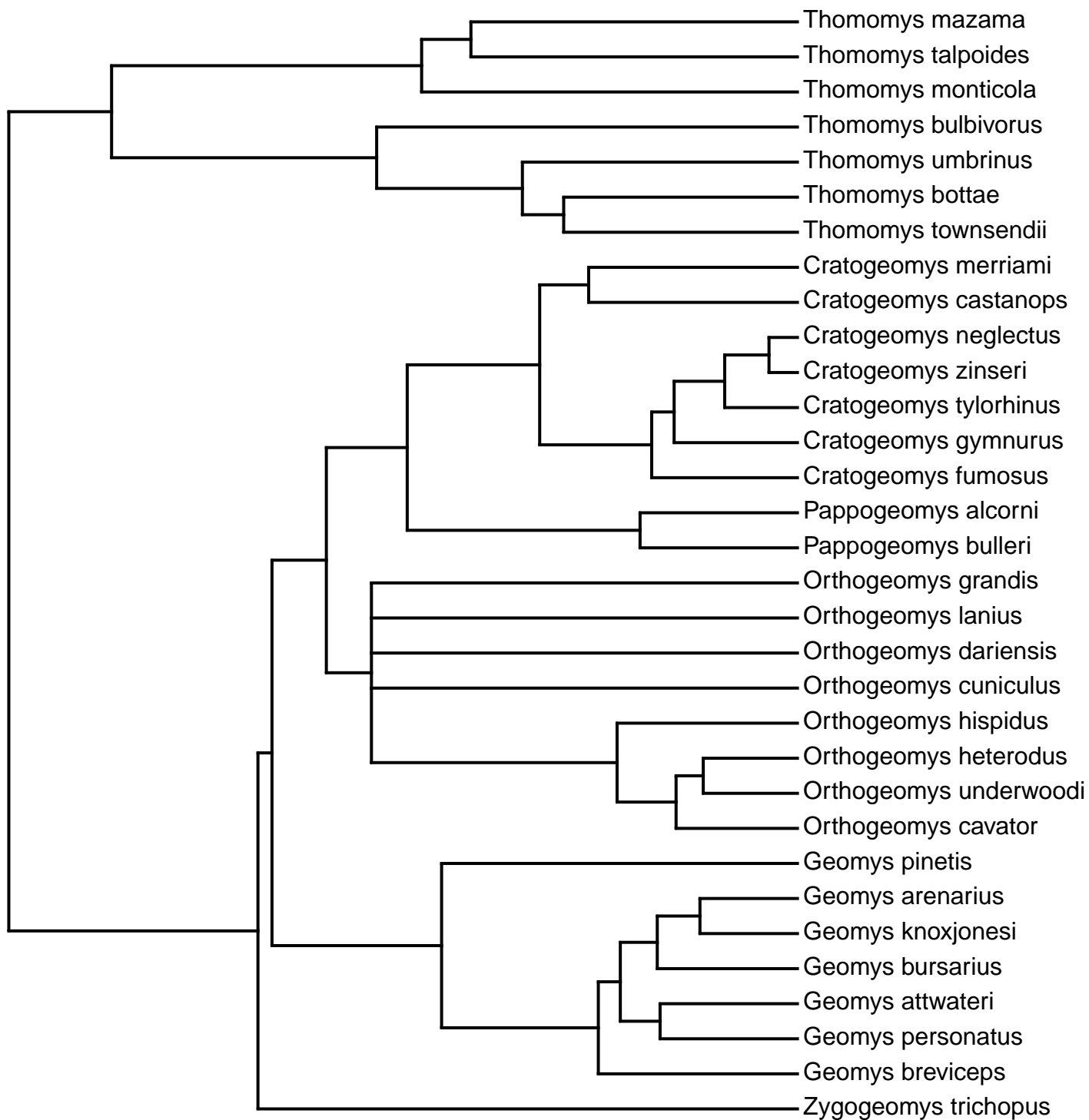

Supplement: Additional file 1: — All phylogenies used in analyses. R script for data extraction and analyses. Detailed results/raw output from SLOUCH. SLOUCH input data. Likelihood plots for all half-life estimations. (ZIP 2442 kb) [file 12862_2016_778_MOESM1_ESM.zip › Additional file 1/Results Bergman's rule - body mass/Geomyidae_tree.pdf]

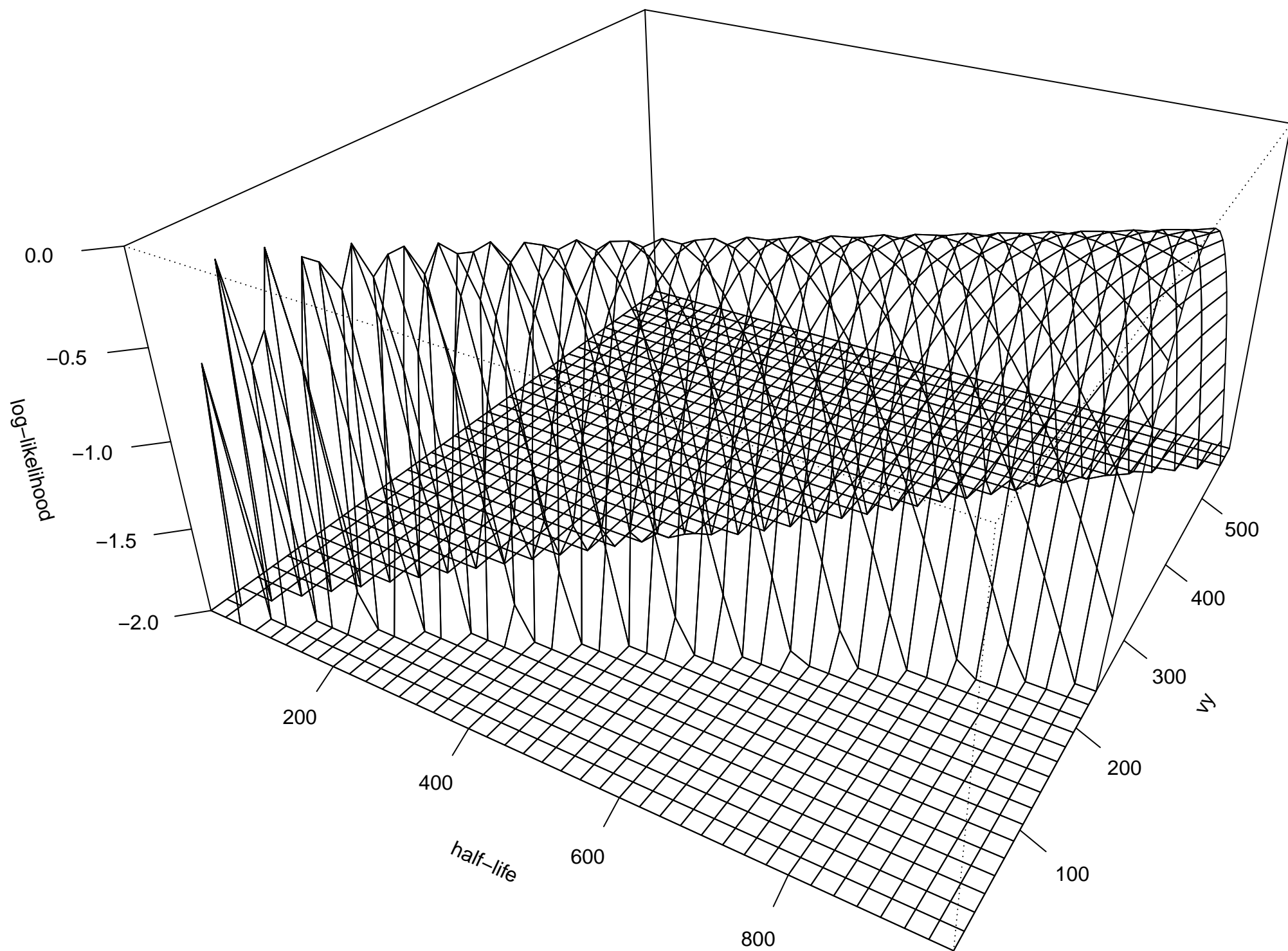

Supplement: Additional file 1: — All phylogenies used in analyses. R script for data extraction and analyses. Detailed results/raw output from SLOUCH. SLOUCH input data. Likelihood plots for all half-life estimations. (ZIP 2442 kb) [file 12862_2016_778_MOESM1_ESM.zip › Additional file 1/Results Bergman's rule - body mass/Heteromyidae_BM_maxlat.pdf]

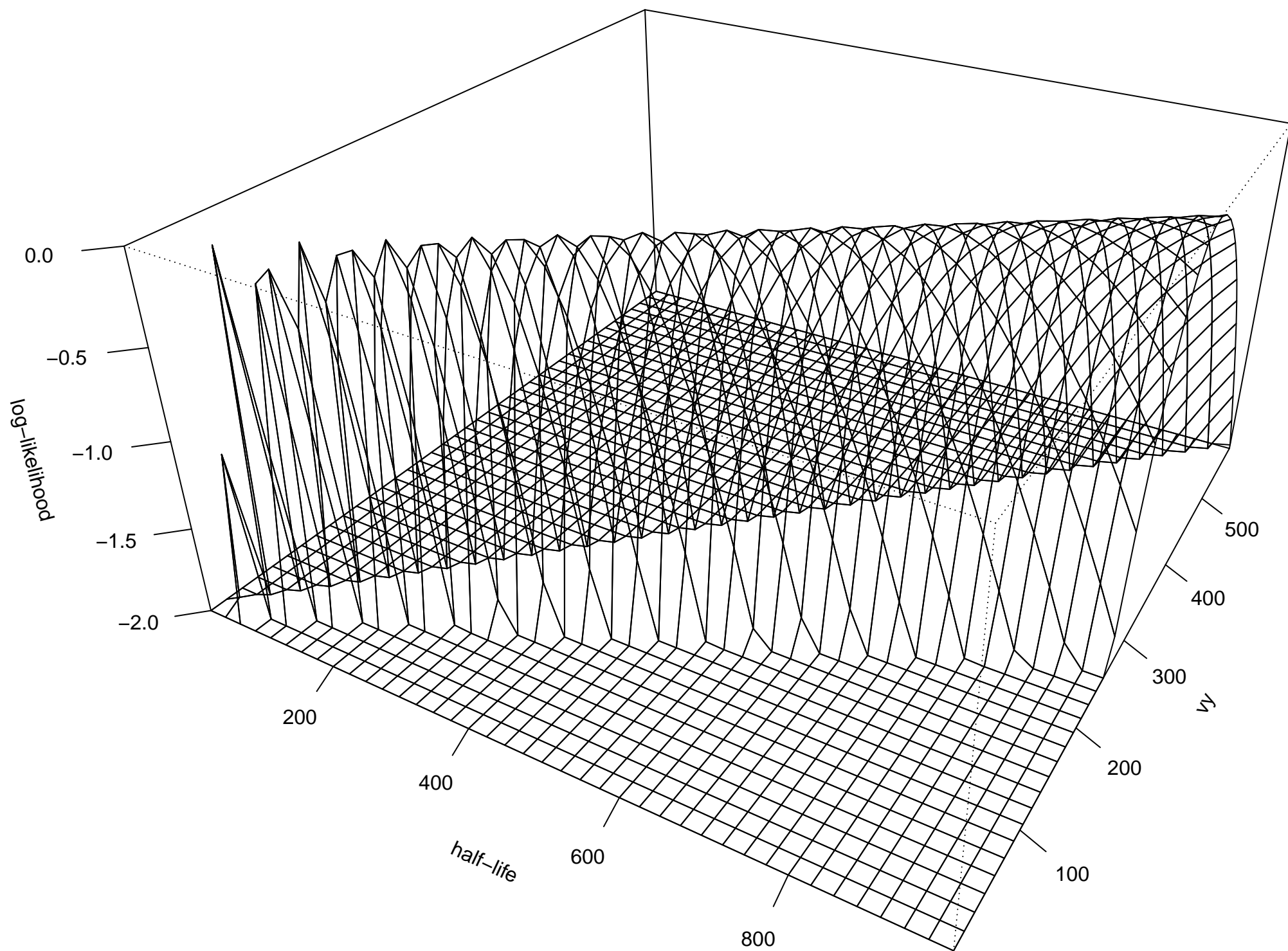

Supplement: Additional file 1: — All phylogenies used in analyses. R script for data extraction and analyses. Detailed results/raw output from SLOUCH. SLOUCH input data. Likelihood plots for all half-life estimations. (ZIP 2442 kb) [file 12862_2016_778_MOESM1_ESM.zip › Additional file 1/Results Bergman's rule - body mass/Heteromyidae_BM_midlat.pdf]

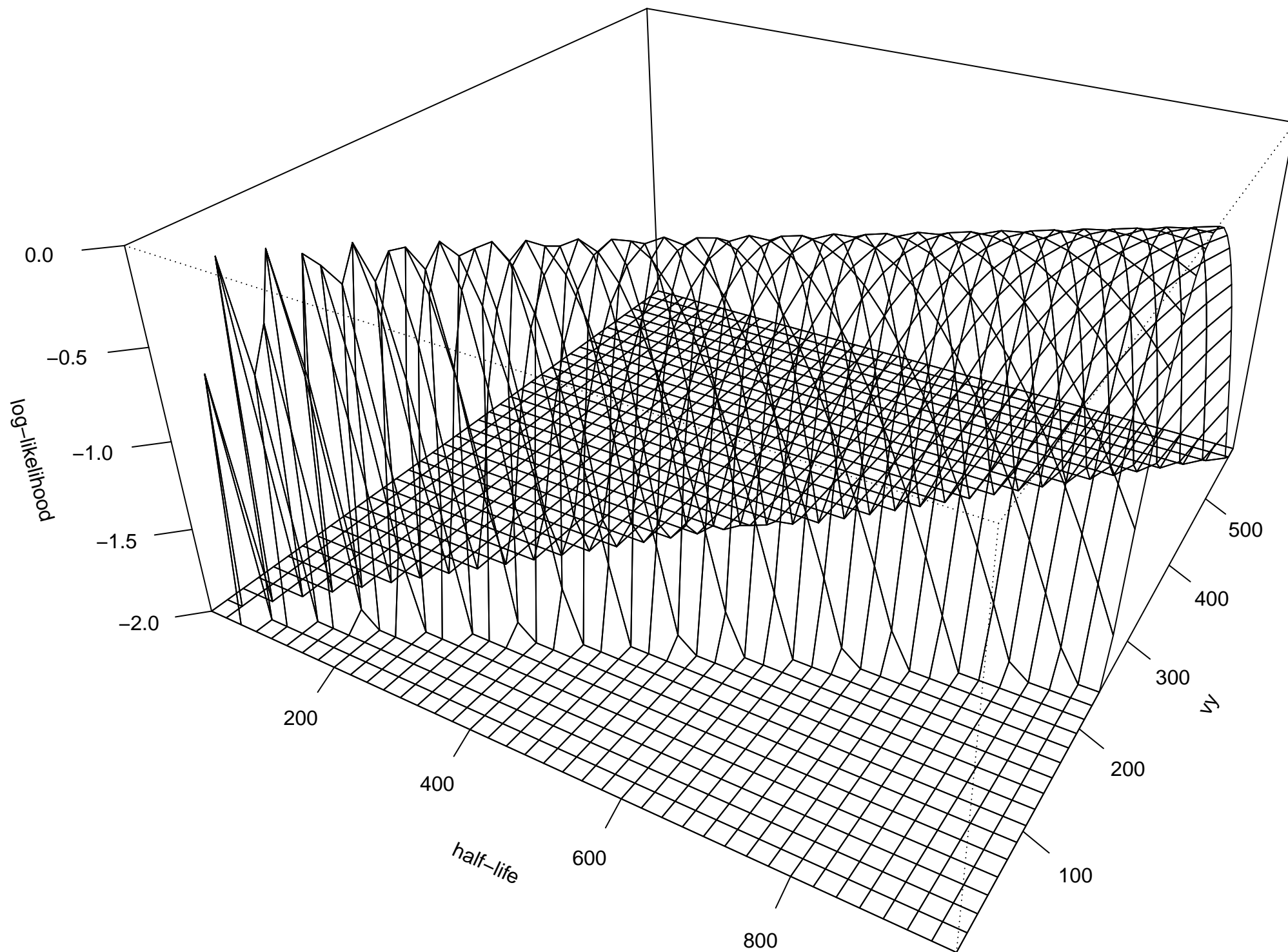

Supplement: Additional file 1: — All phylogenies used in analyses. R script for data extraction and analyses. Detailed results/raw output from SLOUCH. SLOUCH input data. Likelihood plots for all half-life estimations. (ZIP 2442 kb) [file 12862_2016_778_MOESM1_ESM.zip › Additional file 1/Results Bergman's rule - body mass/Heteromyidae_BM_temp.pdf]

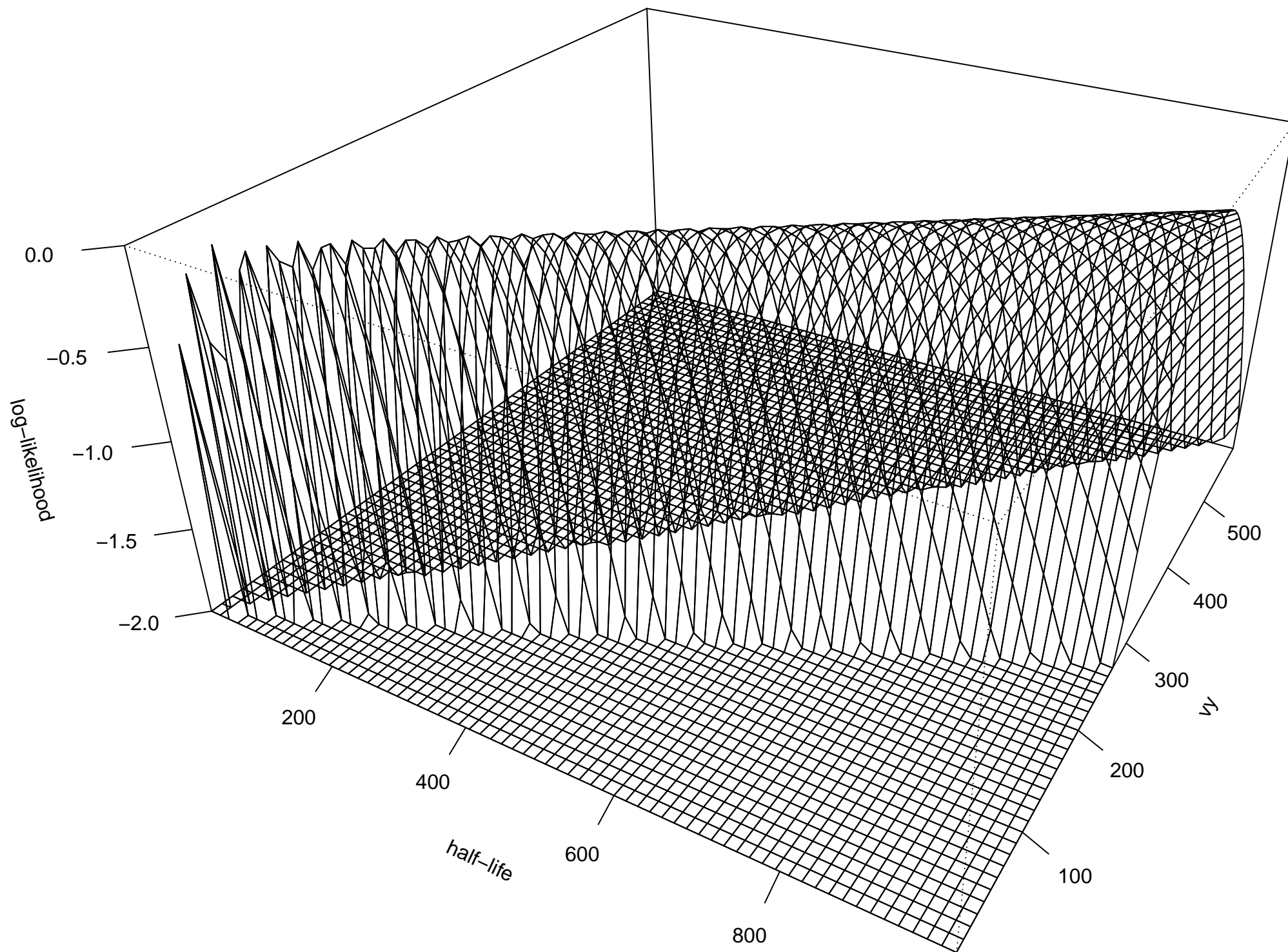

Supplement: Additional file 1: — All phylogenies used in analyses. R script for data extraction and analyses. Detailed results/raw output from SLOUCH. SLOUCH input data. Likelihood plots for all half-life estimations. (ZIP 2442 kb) [file 12862_2016_778_MOESM1_ESM.zip › Additional file 1/Results Bergman's rule - body mass/Heteromyidae_phySig.pdf]

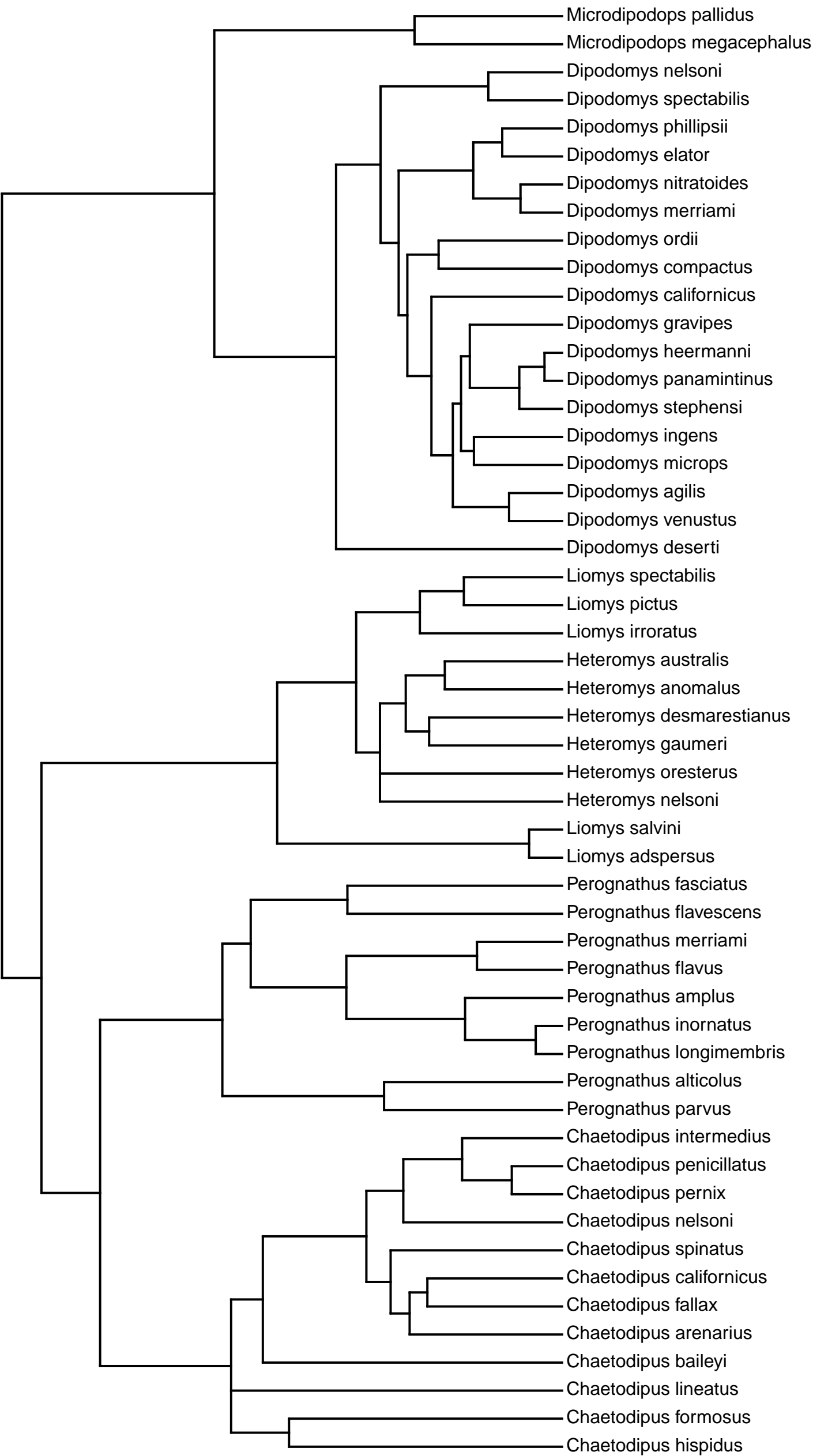

Supplement: Additional file 1: — All phylogenies used in analyses. R script for data extraction and analyses. Detailed results/raw output from SLOUCH. SLOUCH input data. Likelihood plots for all half-life estimations. (ZIP 2442 kb) [file 12862_2016_778_MOESM1_ESM.zip › Additional file 1/Results Bergman's rule - body mass/Heteromyidae_tree.pdf]

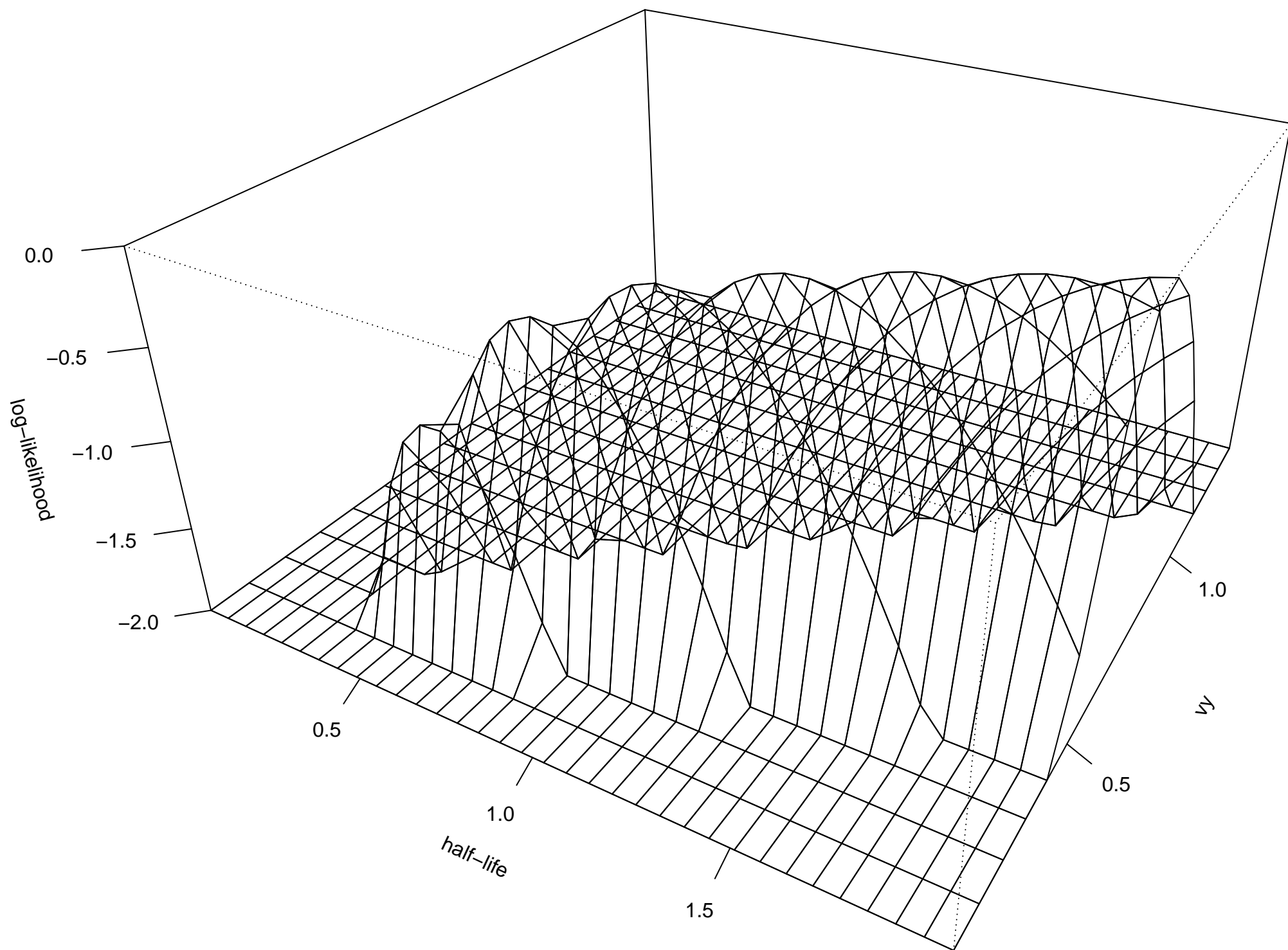

Supplement: Additional file 1: — All phylogenies used in analyses. R script for data extraction and analyses. Detailed results/raw output from SLOUCH. SLOUCH input data. Likelihood plots for all half-life estimations. (ZIP 2442 kb) [file 12862_2016_778_MOESM1_ESM.zip › Additional file 1/Results Bergman's rule - body mass/Leporidae_BM_maxlat.pdf]

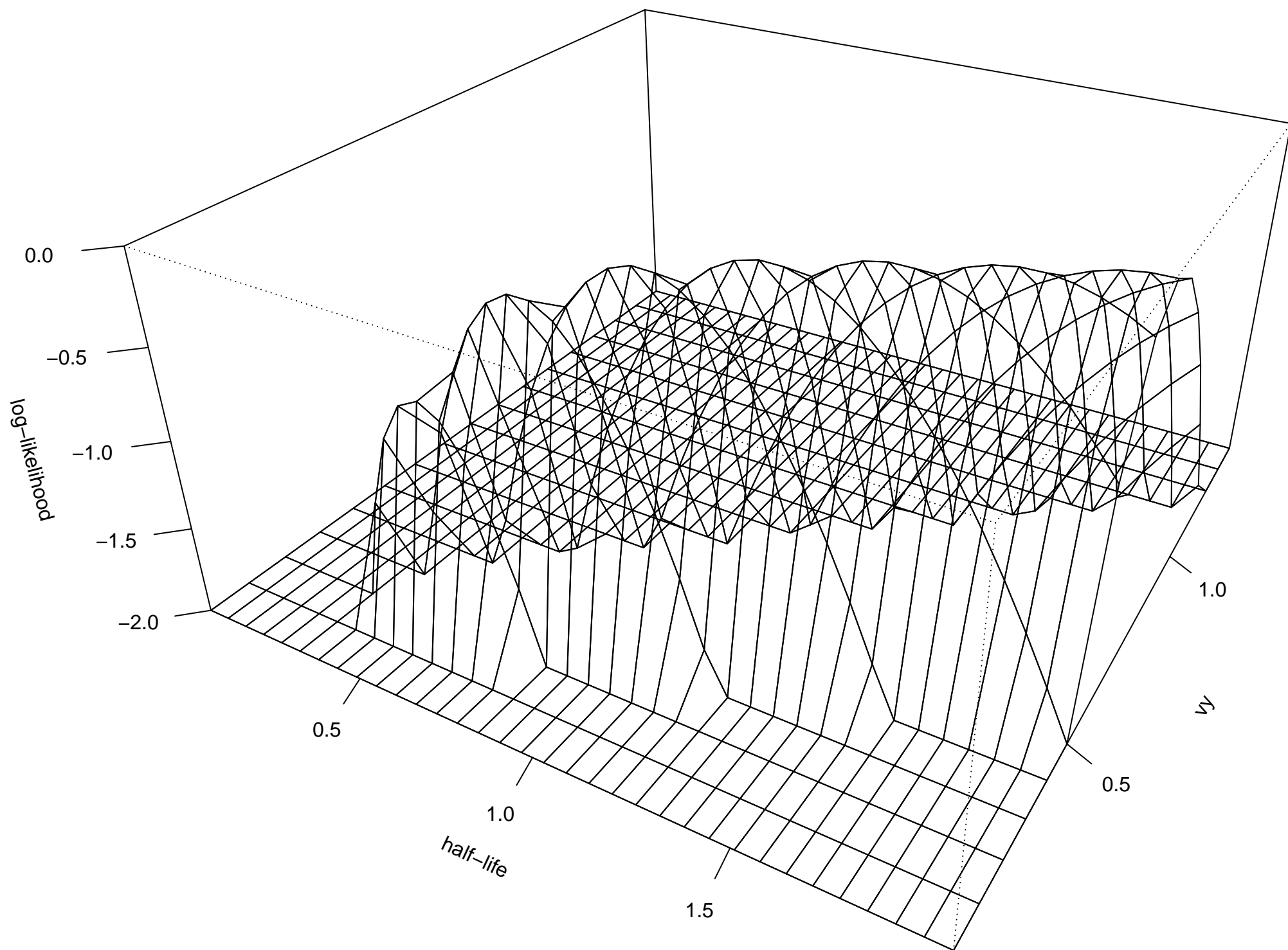

Supplement: Additional file 1: — All phylogenies used in analyses. R script for data extraction and analyses. Detailed results/raw output from SLOUCH. SLOUCH input data. Likelihood plots for all half-life estimations. (ZIP 2442 kb) [file 12862_2016_778_MOESM1_ESM.zip › Additional file 1/Results Bergman's rule - body mass/Leporidae_BM_midlat.pdf]

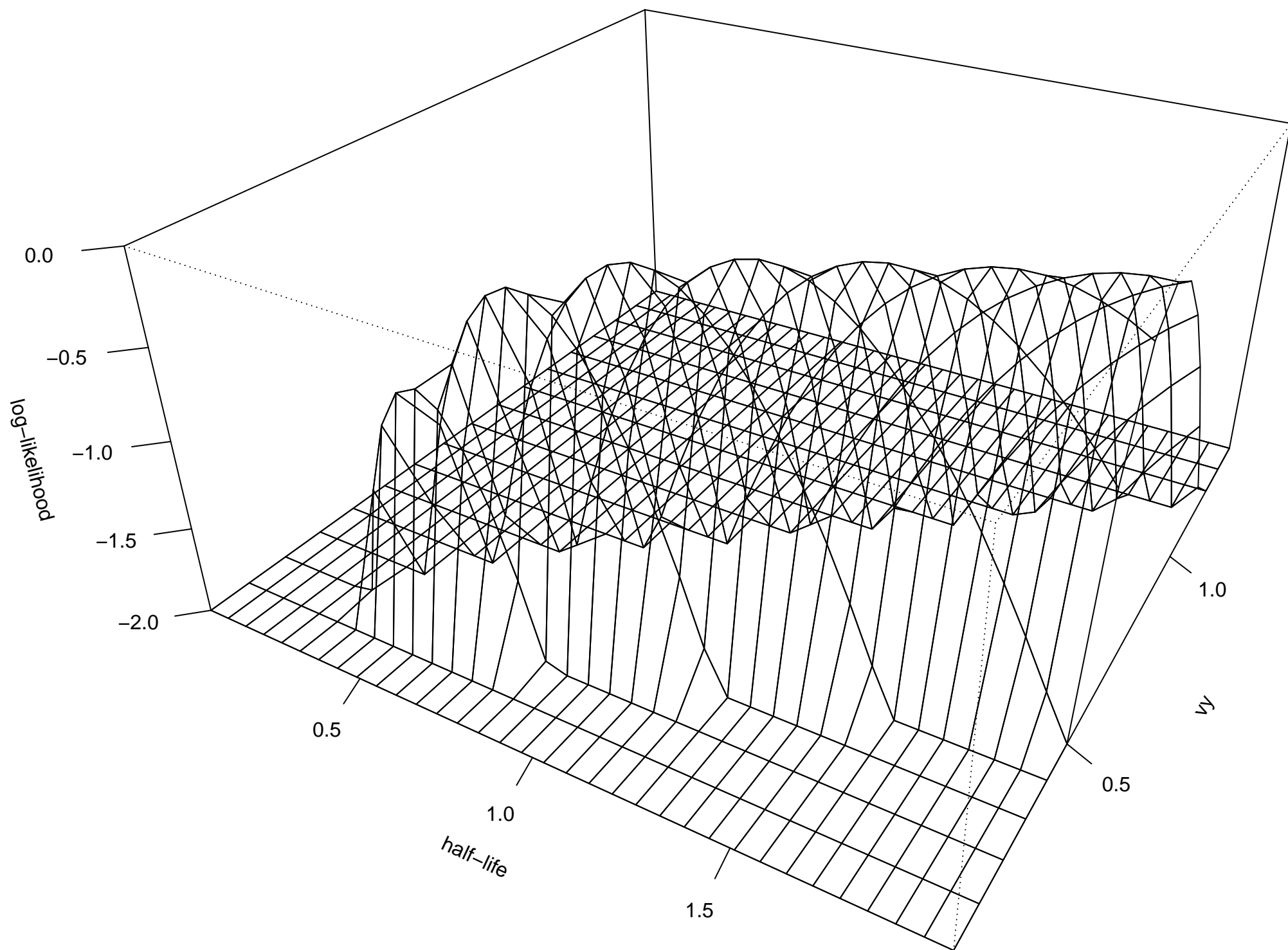

Supplement: Additional file 1: — All phylogenies used in analyses. R script for data extraction and analyses. Detailed results/raw output from SLOUCH. SLOUCH input data. Likelihood plots for all half-life estimations. (ZIP 2442 kb) [file 12862_2016_778_MOESM1_ESM.zip › Additional file 1/Results Bergman's rule - body mass/Leporidae_BM_temp.pdf]

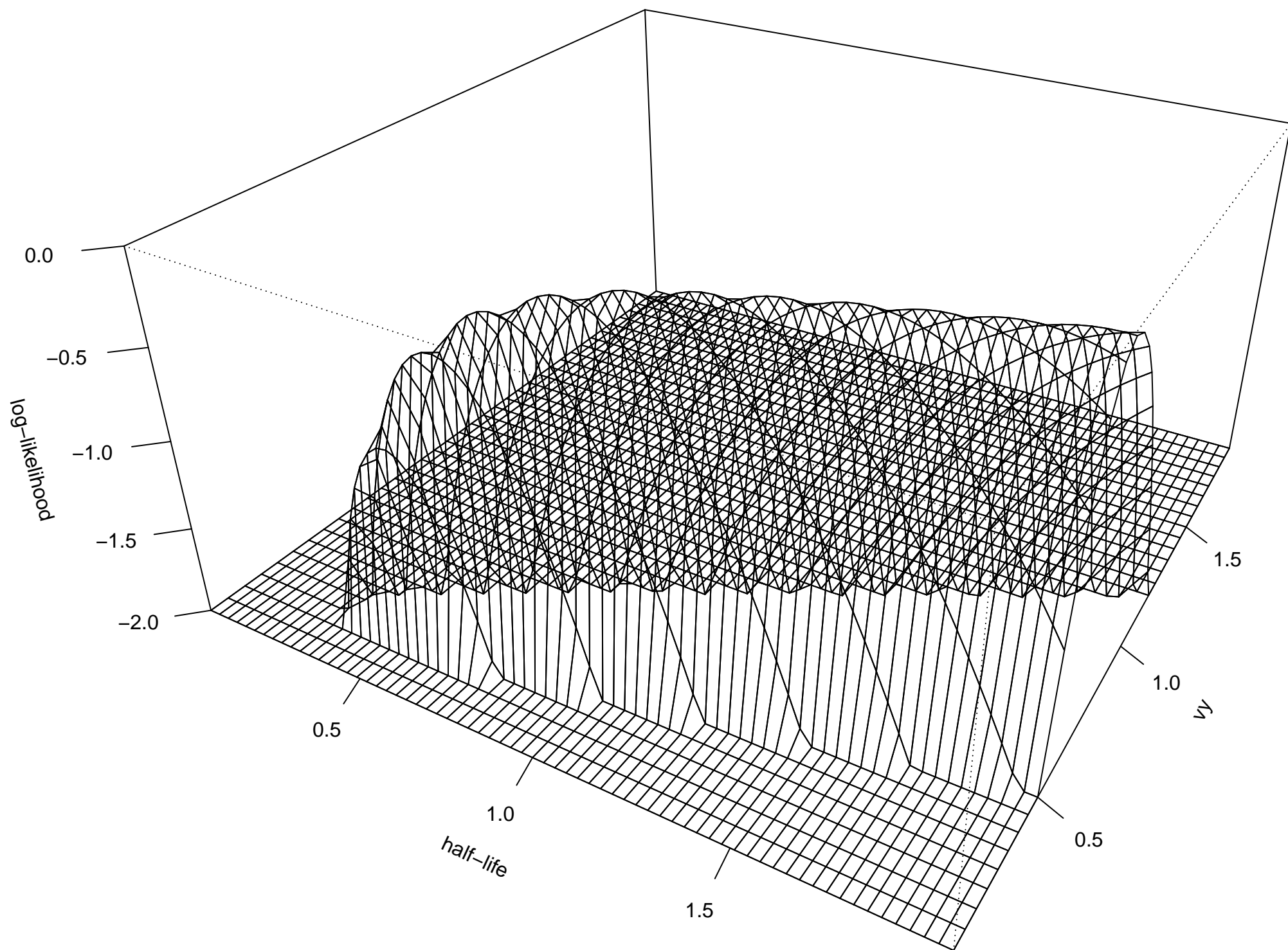

Supplement: Additional file 1: — All phylogenies used in analyses. R script for data extraction and analyses. Detailed results/raw output from SLOUCH. SLOUCH input data. Likelihood plots for all half-life estimations. (ZIP 2442 kb) [file 12862_2016_778_MOESM1_ESM.zip › Additional file 1/Results Bergman's rule - body mass/Leporidae_phySig.pdf]

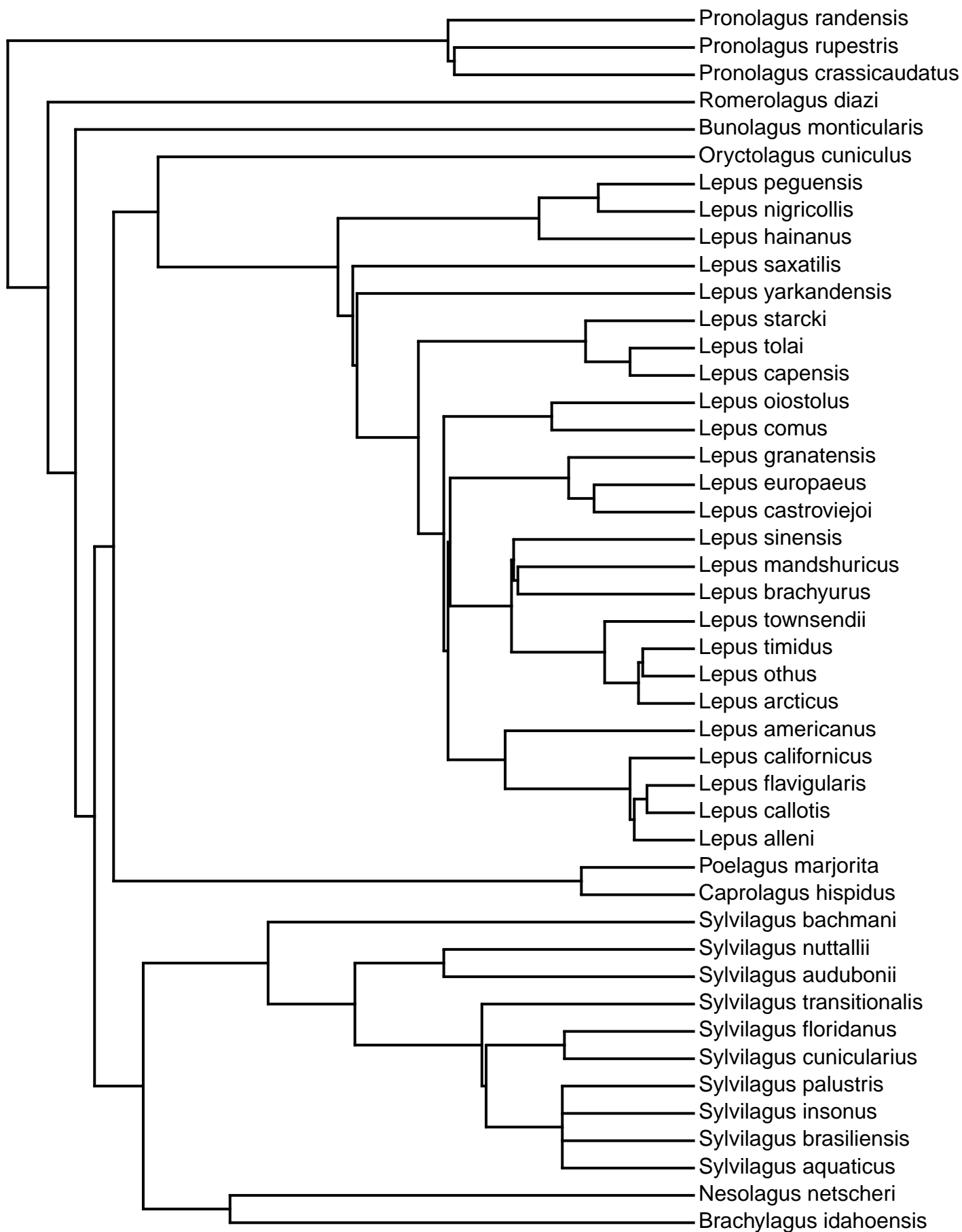

Supplement: Additional file 1: — All phylogenies used in analyses. R script for data extraction and analyses. Detailed results/raw output from SLOUCH. SLOUCH input data. Likelihood plots for all half-life estimations. (ZIP 2442 kb) [file 12862_2016_778_MOESM1_ESM.zip › Additional file 1/Results Bergman's rule - body mass/Leporidae_tree.pdf]

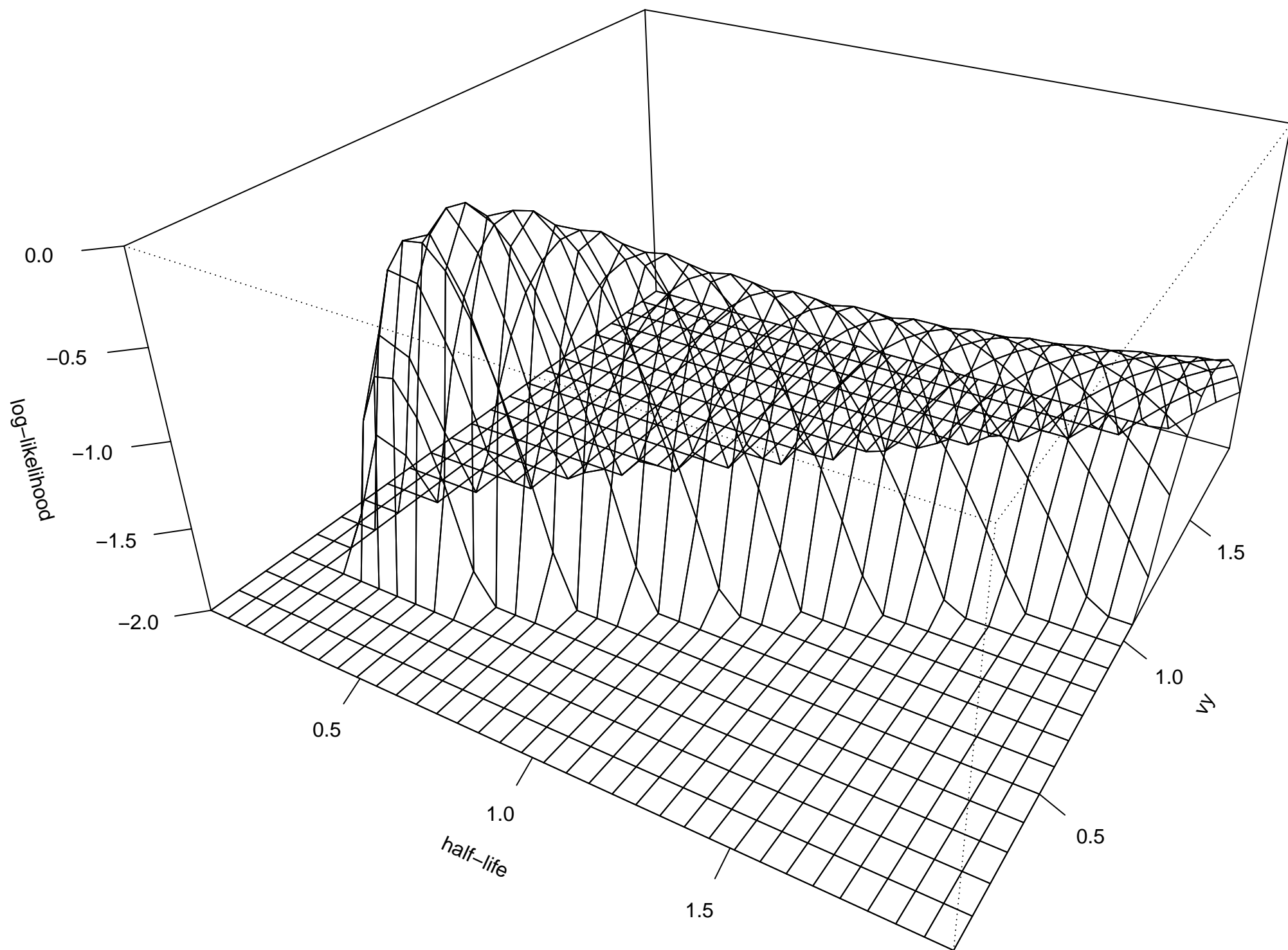

Supplement: Additional file 1: — All phylogenies used in analyses. R script for data extraction and analyses. Detailed results/raw output from SLOUCH. SLOUCH input data. Likelihood plots for all half-life estimations. (ZIP 2442 kb) [file 12862_2016_778_MOESM1_ESM.zip › Additional file 1/Results Bergman's rule - body mass/Macropodidae_BM_maxlat.pdf]

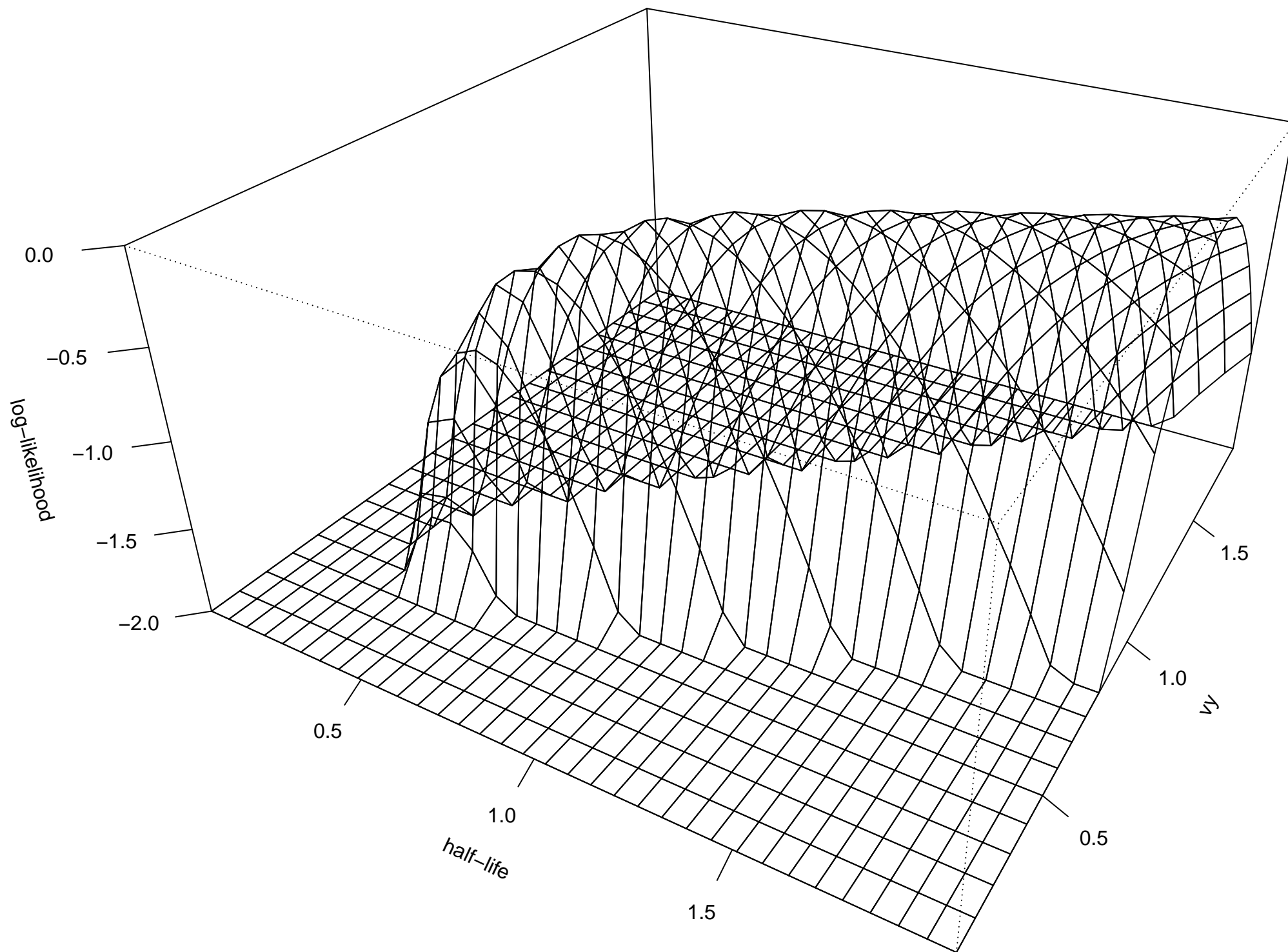

Supplement: Additional file 1: — All phylogenies used in analyses. R script for data extraction and analyses. Detailed results/raw output from SLOUCH. SLOUCH input data. Likelihood plots for all half-life estimations. (ZIP 2442 kb) [file 12862_2016_778_MOESM1_ESM.zip › Additional file 1/Results Bergman's rule - body mass/Macropodidae_BM_midlat.pdf]

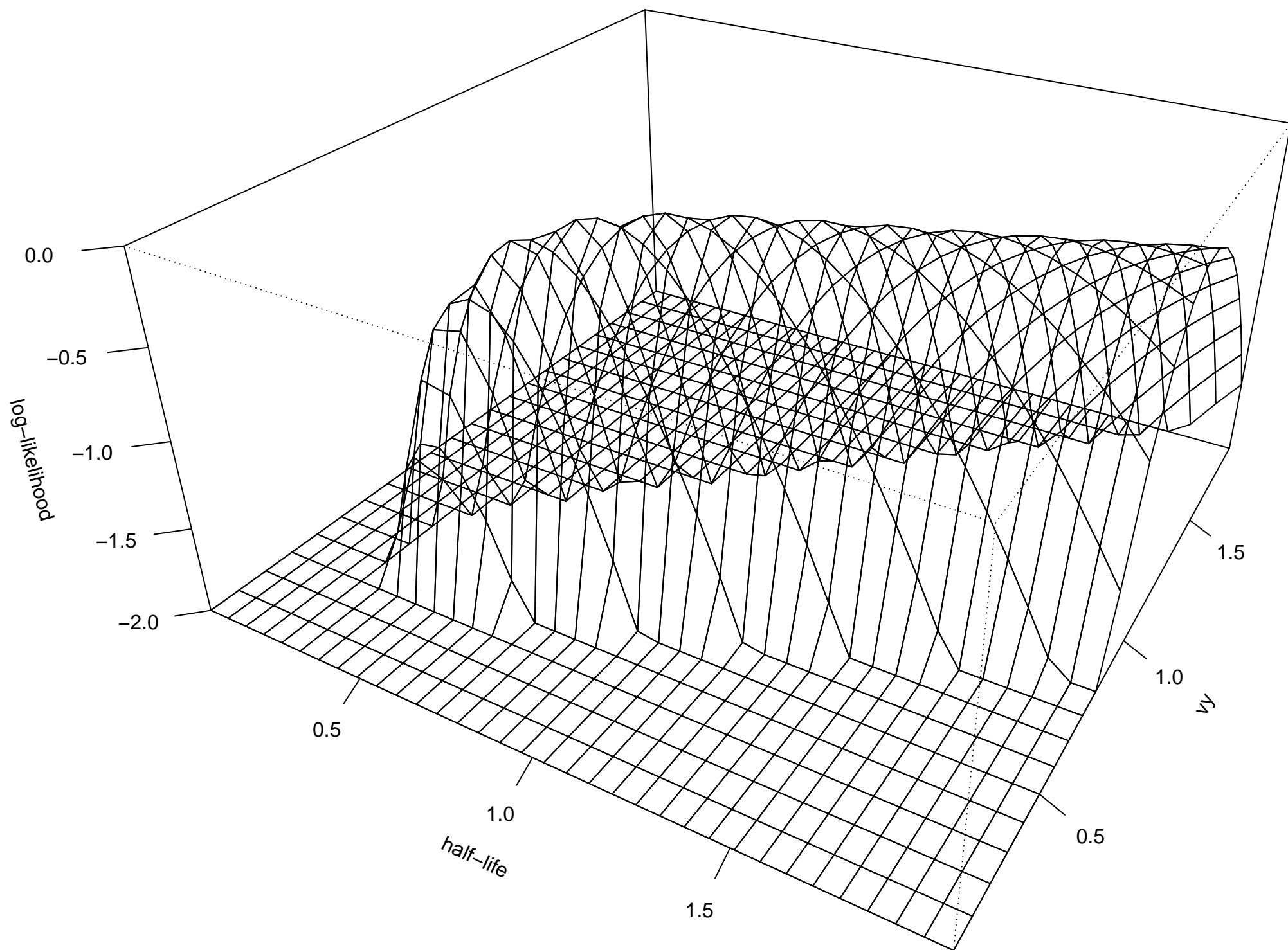

Supplement: Additional file 1: — All phylogenies used in analyses. R script for data extraction and analyses. Detailed results/raw output from SLOUCH. SLOUCH input data. Likelihood plots for all half-life estimations. (ZIP 2442 kb) [file 12862_2016_778_MOESM1_ESM.zip › Additional file 1/Results Bergman's rule - body mass/Macropodidae_BM_temp.pdf]

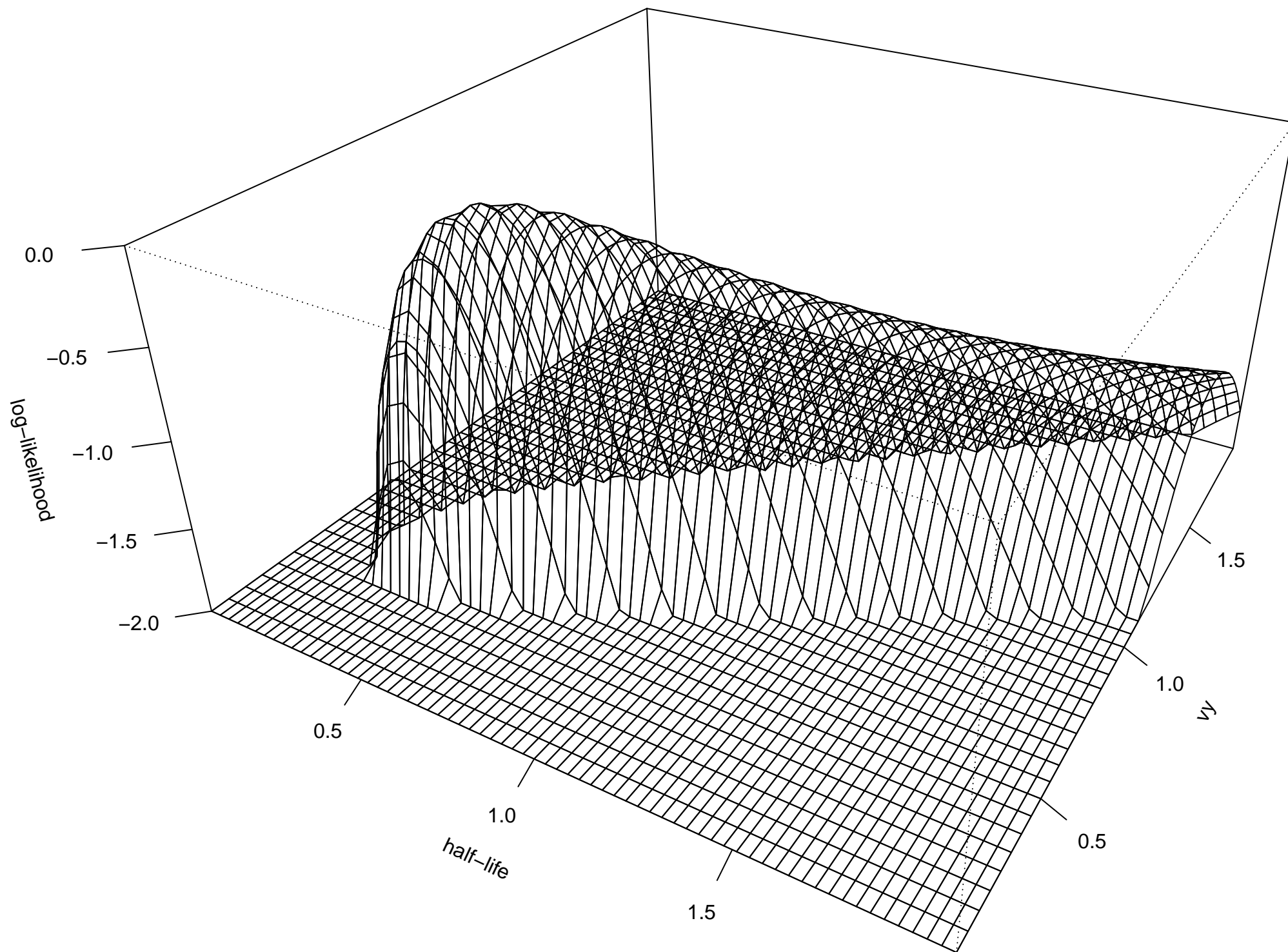

Supplement: Additional file 1: — All phylogenies used in analyses. R script for data extraction and analyses. Detailed results/raw output from SLOUCH. SLOUCH input data. Likelihood plots for all half-life estimations. (ZIP 2442 kb) [file 12862_2016_778_MOESM1_ESM.zip › Additional file 1/Results Bergman's rule - body mass/Macropodidae_phySig.pdf]

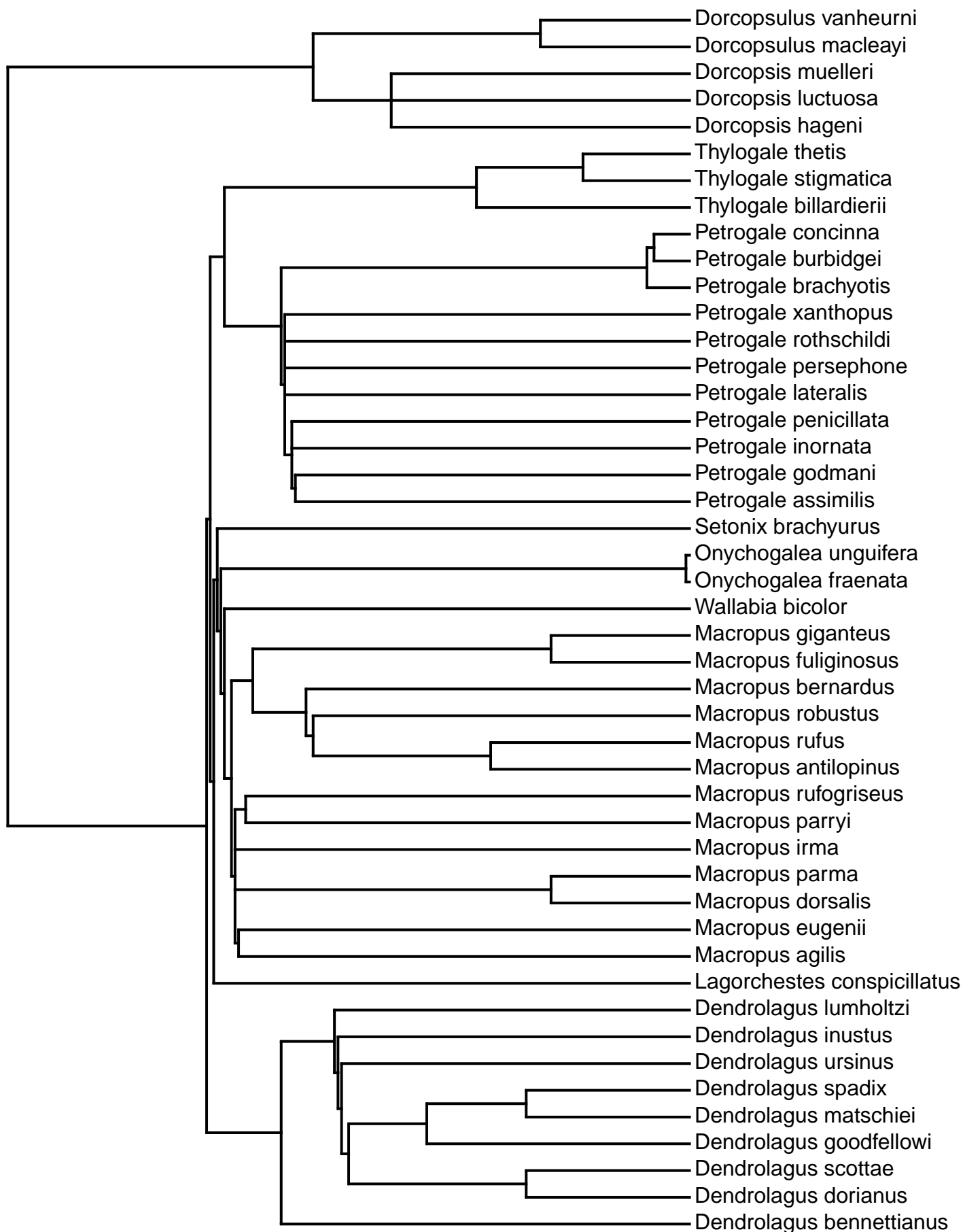

Supplement: Additional file 1: — All phylogenies used in analyses. R script for data extraction and analyses. Detailed results/raw output from SLOUCH. SLOUCH input data. Likelihood plots for all half-life estimations. (ZIP 2442 kb) [file 12862_2016_778_MOESM1_ESM.zip › Additional file 1/Results Bergman's rule - body mass/Macropodidae_tree.pdf]

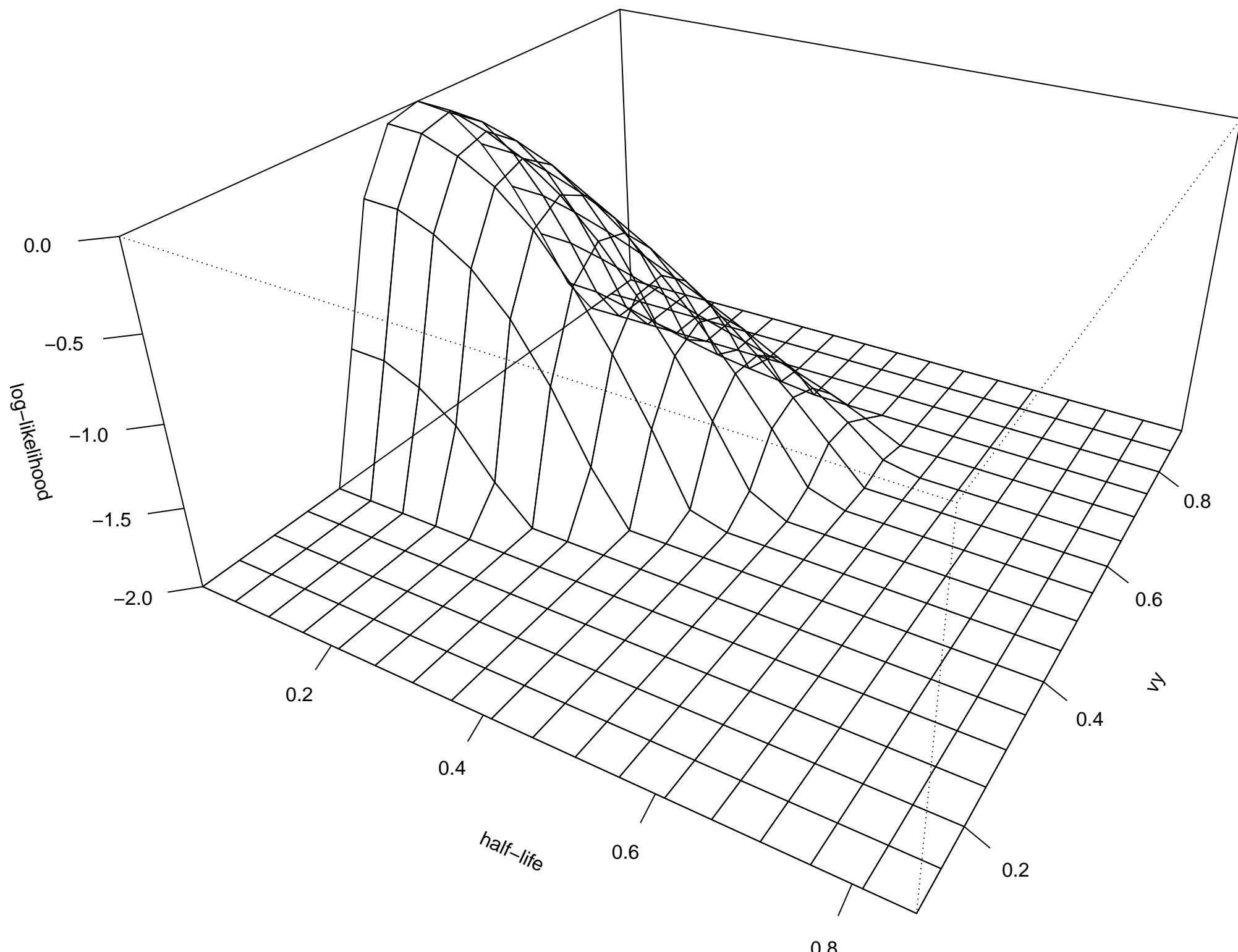

Supplement: Additional file 1: — All phylogenies used in analyses. R script for data extraction and analyses. Detailed results/raw output from SLOUCH. SLOUCH input data. Likelihood plots for all half-life estimations. (ZIP 2442 kb) [file 12862_2016_778_MOESM1_ESM.zip › Additional file 1/Results Bergman's rule - body mass/Molossidae_BM_maxlat.pdf]

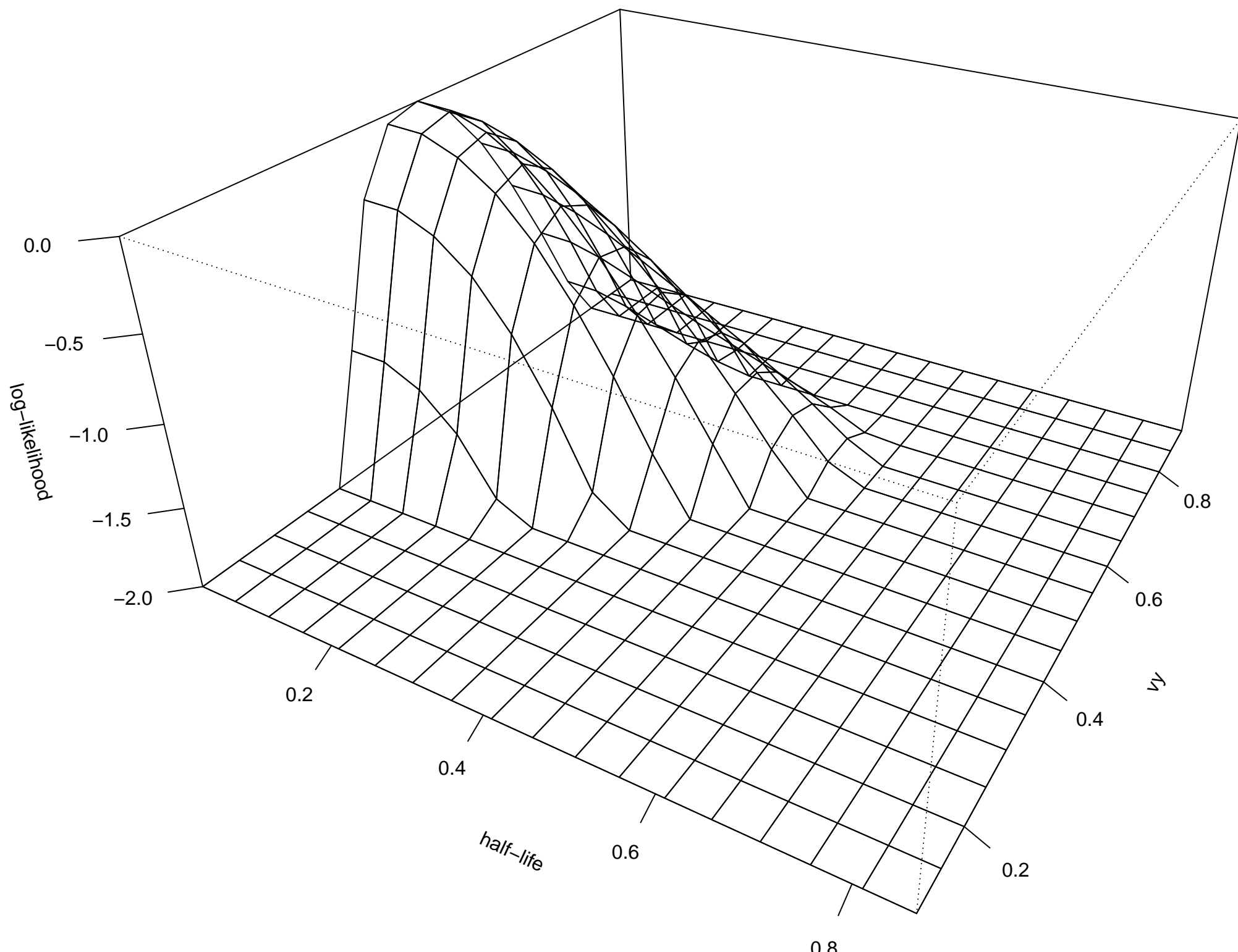

Supplement: Additional file 1: — All phylogenies used in analyses. R script for data extraction and analyses. Detailed results/raw output from SLOUCH. SLOUCH input data. Likelihood plots for all half-life estimations. (ZIP 2442 kb) [file 12862_2016_778_MOESM1_ESM.zip › Additional file 1/Results Bergman's rule - body mass/Molossidae_BM_midlat.pdf]

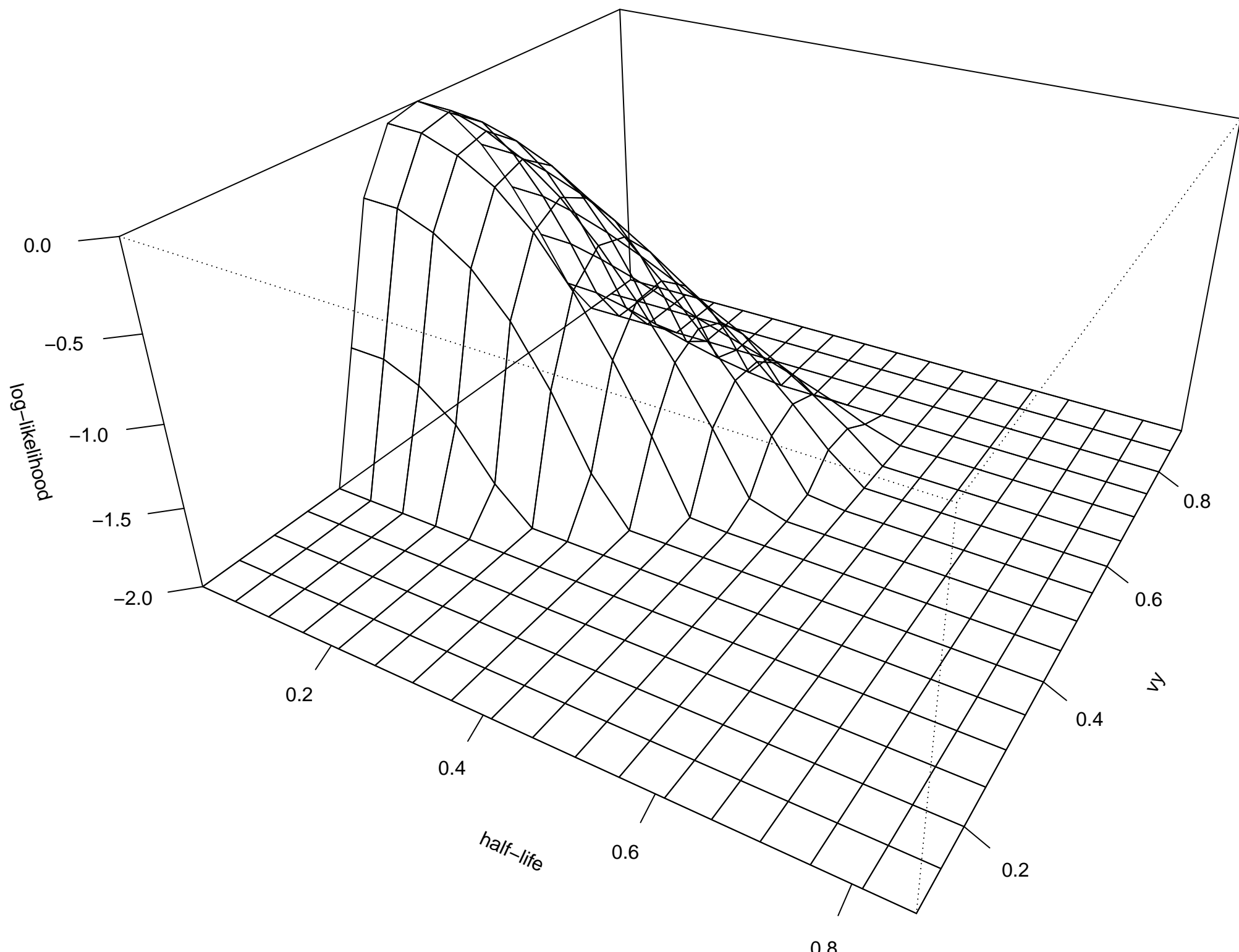

Supplement: Additional file 1: — All phylogenies used in analyses. R script for data extraction and analyses. Detailed results/raw output from SLOUCH. SLOUCH input data. Likelihood plots for all half-life estimations. (ZIP 2442 kb) [file 12862_2016_778_MOESM1_ESM.zip › Additional file 1/Results Bergman's rule - body mass/Molossidae_BM_temp.pdf]

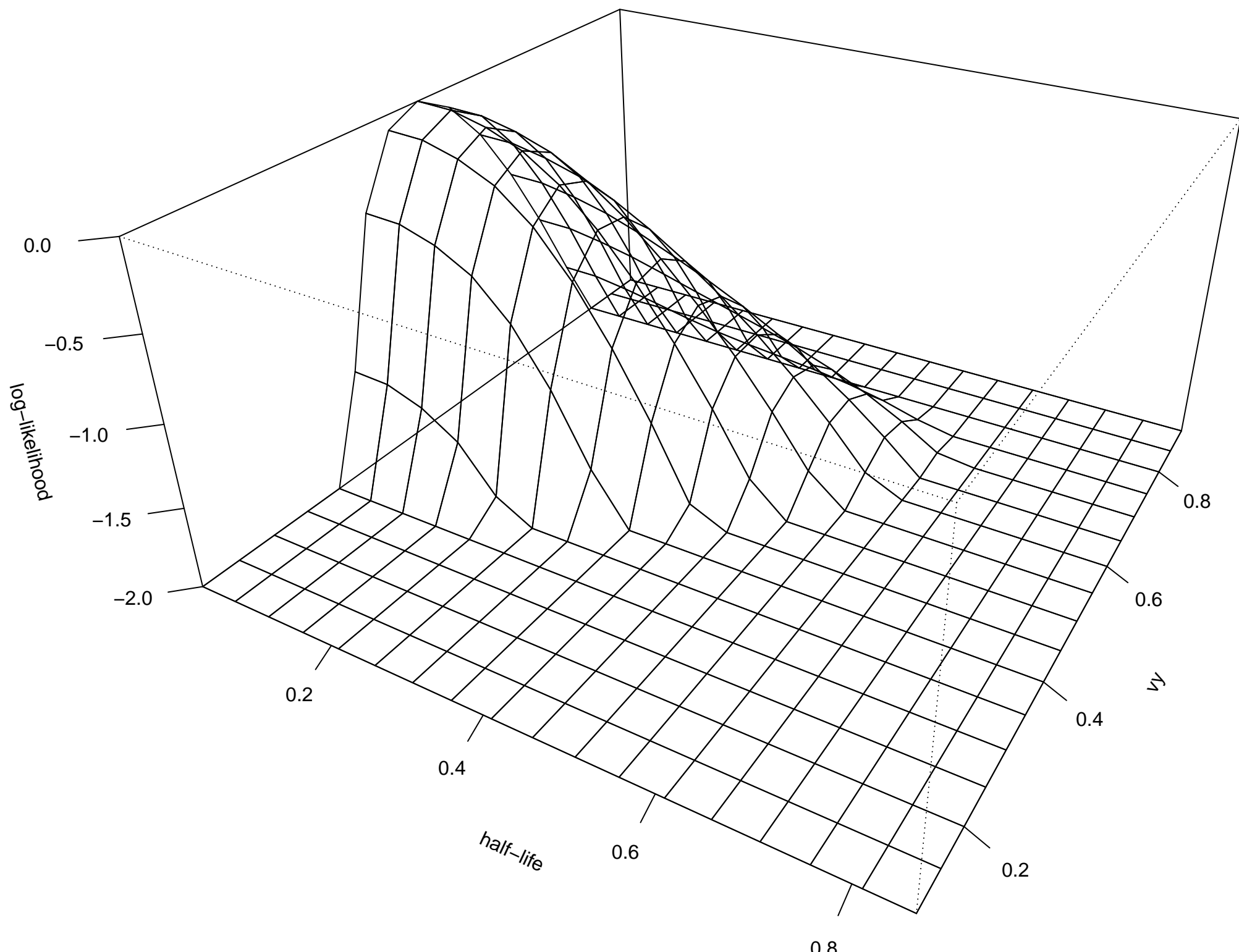

Supplement: Additional file 1: — All phylogenies used in analyses. R script for data extraction and analyses. Detailed results/raw output from SLOUCH. SLOUCH input data. Likelihood plots for all half-life estimations. (ZIP 2442 kb) [file 12862_2016_778_MOESM1_ESM.zip › Additional file 1/Results Bergman's rule - body mass/Molossidae_phySig.pdf]

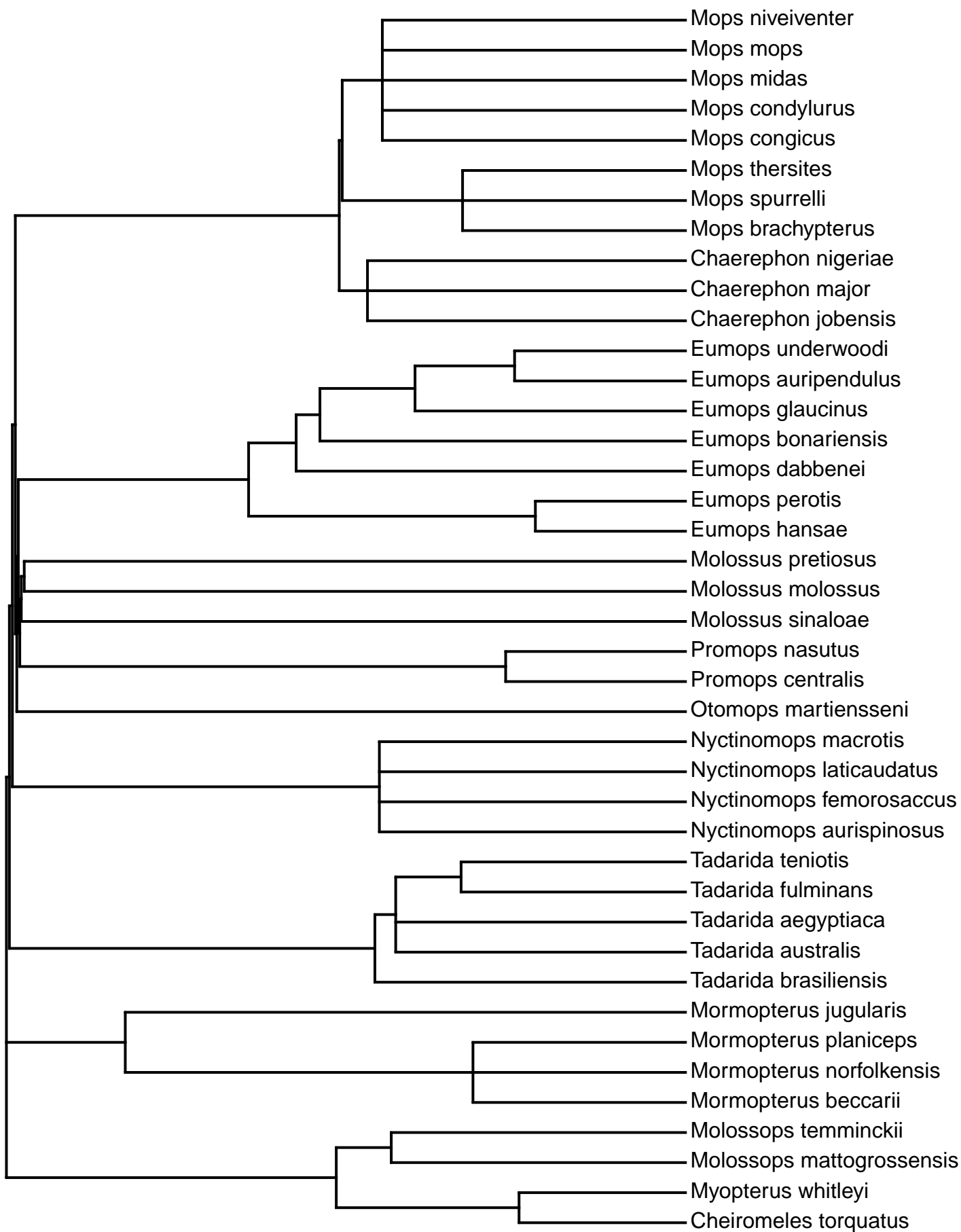

Supplement: Additional file 1: — All phylogenies used in analyses. R script for data extraction and analyses. Detailed results/raw output from SLOUCH. SLOUCH input data. Likelihood plots for all half-life estimations. (ZIP 2442 kb) [file 12862_2016_778_MOESM1_ESM.zip › Additional file 1/Results Bergman's rule - body mass/Molossidae_tree.pdf]
